# Supplementary material for: In Silico Analysis of Nanoplastics’ and β-amyloid Fibrils’ Interactions
Source: Molecules. 2023 Jan 2;28(1):388. doi: 10.3390/molecules28010388 (PMC9824275; doi:10.3390/molecules28010388)
Supplement: Supplementary file 1 [file molecules-28-00388-s001.zip › molecules-2074866-supplementary.pdf]

Supporting Information

In silico analysis of nanoplastics and β-amyloid fibrils interactions

Silvia Gabbrielli, Luca Colnaghi, Gemma Mazzuoli-Weber, Alberto Redaelli, Alfonso Gautieri

1.1 Polystyrene NP Coordinates

```
POLYSTYRENE
 1800
    1STYR     B    1  14.833   8.322   5.673 -0.0485  0.1544  0.2538
    1STYR    R1    2  15.019   8.390   5.864 -0.0636  0.1444  0.2689
    1STYR    R2    3  15.285   8.357   5.897 -0.0619  0.1759  0.2879
    1STYR    R3    4  15.114   8.284   6.093 -0.0661  0.1716  0.2826
    2STYR     B    5  14.639   8.252   5.787 -0.0593  0.1367  0.2189
    2STYR    R1    6  14.722   8.070   5.635 -0.0170  0.1345  0.2431
    2STYR    R2    7  14.762   7.805   5.671 -0.0062  0.1363  0.2440
    2STYR    R3    8  14.638   7.893   5.448  0.0061  0.1318  0.2354
    3STYR     B    9  14.433   8.145   5.845 -0.0443  0.1333  0.2108
    3STYR    R1   10  14.659   8.128   6.012 -0.0856  0.1439  0.2521
    3STYR    R2   11  14.838   7.958   6.122 -0.0685  0.1764  0.2751
    3STYR    R3   12  14.741   8.164   6.266 -0.1172  0.1649  0.2594
    4STYR     B   13  14.205   8.105   5.904 -0.0541  0.1067  0.1630
    4STYR    R1   14  14.295   7.941   5.724 -0.0283  0.1099  0.2062
    4STYR    R2   15  14.321   7.676   5.681  0.0068  0.1100  0.2259
    4STYR    R3   16  14.200   7.831   5.496  0.0096  0.0845  0.2026
    5STYR     B   17  13.970   8.173   5.918 -0.0532  0.0974  0.1599
    5STYR    R1   18  14.007   7.927   5.975 -0.0488  0.1013  0.1505
    5STYR    R2   19  13.911   7.756   6.160 -0.0629  0.1027  0.1445
    5STYR    R3   20  13.925   7.681   5.901 -0.0146  0.0898  0.1507
    6STYR     B   21  13.906   8.419   5.877 -0.0498  0.0933  0.1364
    6STYR    R1   22  13.725   8.234   5.803 -0.0425  0.0380  0.1247
    6STYR    R2   23  13.577   8.165   5.588 -0.0353  0.0892  0.1032
    6STYR    R3   24  13.463   8.178   5.833 -0.0493  0.0557  0.0985
    7STYR     B   25  14.074   8.551   5.780 -0.0589  0.1011  0.1270
    7STYR    R1   26  13.873   8.687   5.869 -0.0849  0.0822  0.1266
    7STYR    R2   27  13.827   8.905   6.021 -0.1179  0.0910  0.1042
    7STYR    R3   28  13.713   8.885   5.777 -0.0931  0.0612  0.0950
    8STYR     B   29  14.289   8.540   5.663 -0.0468  0.1024  0.1719
    8STYR    R1   30  14.060   8.423   5.551 -0.0403  0.0777  0.1570
    8STYR    R2   31  13.974   8.191   5.444 -0.0009  0.0724  0.1367
    8STYR    R3   32  13.896   8.426   5.337 -0.0078  0.0678  0.1317
    9STYR     B   33  14.493   8.645   5.720 -0.0690  0.1282  0.1991
    9STYR    R1   34  14.467   8.649   5.476 -0.0461  0.1028  0.1917
    9STYR    R2   35  14.642   8.600   5.276 -0.0247  0.0981  0.2115
    9STYR    R3   36  14.465   8.803   5.254 -0.0220  0.0979  0.1881
   10STYR     B   37  14.668   8.764   5.785 -0.0689  0.1321  0.2132
   10STYR    R1   38  14.408   8.822   5.853 -0.0845  0.1142  0.1774
   10STYR    R2   39  14.246   8.878   6.062 -0.1219  0.1182  0.1472
   10STYR    R3   40  14.194   8.982   5.818 -0.0856  0.1029  0.1328
   11STYR     B   41  14.876   8.871   5.788 -0.0879  0.1340  0.2320
   11STYR    R1   42  14.788   8.766   6.014 -0.0915  0.1402  0.2326
   11STYR    R2   43  14.879   8.608   6.213 -0.1197  0.1515  0.2544
   11STYR    R3   44  14.719   8.818   6.269 -0.1327  0.1510  0.2193
   12STYR     B   45  15.009   9.041   5.684 -0.0641  0.1582  0.2353
   12STYR    R1   46  15.062   8.789   5.618 -0.0583  0.1436  0.2464
   12STYR    R2   47  15.078   8.620   5.408 -0.0262  0.1113  0.2746
   12STYR    R3   48  15.280   8.632   5.586 -0.0428  0.1562  0.2904
   13STYR     B   49  14.989   9.277   5.668 -0.1183  0.1289  0.2298
   13STYR    R1   50  14.977   9.163   5.913 -0.1156  0.1616  0.2205
   13STYR    R2   51  14.814   9.186   6.127 -0.1454  0.1655  0.1974
   13STYR    R3   52  15.081   9.168   6.162 -0.1489  0.1833  0.2340
   14STYR     B   53  14.843   9.415   5.527 -0.0925  0.1384  0.1963
```

14STYR	R1	54	14.929	9.187	5.421	-0.0585	0.1380	0.2135
14STYR	R2	55	14.840	8.973	5.282	-0.0309	0.1337	0.2022
14STYR	R3	56	15.043	9.122	5.185	-0.0230	0.1409	0.2297
15STYR	B	57	14.670	9.575	5.494	-0.1203	0.1226	0.1653
15STYR	R1	58	14.844	9.524	5.287	-0.0572	0.1080	0.1881
15STYR	R2	59	14.843	9.466	5.023	-0.0064	0.1014	0.1893
15STYR	R3	60	15.031	9.631	5.125	-0.0567	0.1448	0.2129
16STYR	B	61	14.445	9.638	5.573	-0.1091	0.1060	0.1232
16STYR	R1	62	14.536	9.380	5.603	-0.1001	0.1204	0.1693
16STYR	R2	63	14.558	9.203	5.805	-0.0763	0.1176	0.1643
16STYR	R3	64	14.501	9.116	5.556	-0.0776	0.1183	0.1644
17STYR	B	65	14.312	9.842	5.532	-0.1071	0.1126	0.0963
17STYR	R1	66	14.385	9.689	5.323	-0.0774	0.1083	0.1355
17STYR	R2	67	14.501	9.719	5.081	-0.0436	0.0867	0.1489
17STYR	R3	68	14.274	9.575	5.104	-0.0373	0.0740	0.1330
18STYR	B	69	14.223	10.053	5.613	-0.1260	0.0895	0.0979
18STYR	R1	70	14.271	10.026	5.351	-0.1011	0.0967	0.0936
18STYR	R2	71	14.162	10.033	5.104	-0.0714	0.0658	0.0795
18STYR	R3	72	14.407	10.138	5.146	-0.0846	0.0882	0.0999
19STYR	B	73	14.014	10.130	5.717	-0.1589	0.0890	0.0838
19STYR	R1	74	14.232	10.263	5.795	-0.1675	0.1026	0.0758
19STYR	R2	75	14.358	10.349	6.019	-0.2067	0.1219	0.0906
19STYR	R3	76	14.275	10.527	5.833	-0.1880	0.1071	0.0680
20STYR	B	77	13.794	10.053	5.737	-0.1478	0.0686	0.0349
20STYR	R1	78	13.837	10.263	5.875	-0.1648	0.0950	0.0443
20STYR	R2	79	13.738	10.510	5.918	-0.1850	0.0929	0.0108
20STYR	R3	80	13.818	10.359	6.127	-0.2165	0.1095	0.0351
21STYR	B	81	13.637	9.930	5.870	-0.1487	0.0732	0.0463
21STYR	R1	82	13.883	9.817	5.813	-0.1320	0.0759	0.0649
21STYR	R2	83	14.108	9.711	5.918	-0.1350	0.0888	0.0845
21STYR	R3	84	13.992	9.596	5.703	-0.1187	0.0728	0.0843
22STYR	B	85	13.659	9.877	6.108	-0.1969	0.0945	0.0214
22STYR	R1	86	13.470	10.043	6.066	-0.1841	0.0802	0.0089
22STYR	R2	87	13.379	10.279	6.159	-0.2062	0.0834	-0.0204
22STYR	R3	88	13.206	10.079	6.106	-0.1913	0.0732	-0.0309
23STYR	B	89	13.752	9.718	6.245	-0.1993	0.1008	0.0574
23STYR	R1	90	13.894	9.939	6.233	-0.1976	0.1125	0.0675
23STYR	R2	91	14.022	10.106	6.403	-0.2235	0.1247	0.0752
23STYR	R3	92	14.136	10.039	6.167	-0.2003	0.1307	0.0847
24STYR	B	93	13.953	9.632	6.325	-0.1939	0.1145	0.0941
24STYR	R1	94	13.787	9.478	6.173	-0.1492	0.0985	0.0686
24STYR	R2	95	13.635	9.255	6.174	-0.1384	0.0911	0.0644
24STYR	R3	96	13.774	9.322	5.952	-0.1134	0.0827	0.0775
25STYR	B	97	14.055	9.458	6.448	-0.1936	0.1382	0.0967
25STYR	R1	98	14.204	9.670	6.395	-0.1992	0.1213	0.1106
25STYR	R2	99	14.435	9.751	6.280	-0.1922	0.1388	0.1369
25STYR	R3	100	14.358	9.856	6.516	-0.2296	0.1366	0.1257
26STYR	B	101	14.033	9.240	6.512	-0.1689	0.1394	0.1285
26STYR	R1	102	14.181	9.306	6.305	-0.1748	0.1005	0.1199
26STYR	R2	103	14.433	9.299	6.207	-0.1642	0.1404	0.1437
26STYR	R3	104	14.224	9.326	6.039	-0.1532	0.1009	0.1233
27STYR	B	105	13.911	9.047	6.561	-0.1890	0.1301	0.1071
27STYR	R1	106	14.103	9.123	6.741	-0.2236	0.1512	0.1259
27STYR	R2	107	14.313	9.036	6.886	-0.2333	0.1724	0.1530
27STYR	R3	108	14.138	9.209	6.995	-0.2621	0.1586	0.1287
28STYR	B	109	13.819	8.853	6.457	-0.1628	0.1060	0.1048
28STYR	R1	110	13.643	9.015	6.564	-0.1828	0.0950	0.0720
28STYR	R2	111	13.436	8.998	6.737	-0.2119	0.0951	0.0375
28STYR	R3	112	13.406	9.130	6.504	-0.1751	0.0887	0.0291
29STYR	B	113	13.880	8.649	6.376	-0.1375	0.1126	0.1241
29STYR	R1	114	14.070	8.779	6.484	-0.1702	0.1432	0.1515
29STYR	R2	115	14.254	8.698	6.664	-0.1830	0.1294	0.1585
29STYR	R3	116	14.325	8.864	6.464	-0.1669	0.1378	0.1713
30STYR	B	117	14.012	8.473	6.307	-0.1222	0.1292	0.1476
30STYR	R1	118	13.899	8.438	6.539	-0.1432	0.1143	0.1295
30STYR	R2	119	13.741	8.273	6.683	-0.1522	0.1198	0.1260
30STYR	R3	120	13.976	8.359	6.786	-0.1639	0.1327	0.1419
31STYR	B	121	14.067	8.249	6.342	-0.1119	0.1136	0.1593
31STYR	R1	122	14.264	8.417	6.299	-0.1243	0.1463	0.1782
31STYR	R2	123	14.466	8.490	6.136	-0.1046	0.1495	0.2040
31STYR	R3	124	14.506	8.477	6.403	-0.1355	0.1399	0.2082
32STYR	B	125	14.246	8.091	6.337	-0.0929	0.1566	0.2036
32STYR	R1	126	14.052	8.029	6.502	-0.1246	0.1346	0.1664

32STYR	R2	127	13.858	7.853	6.566	-0.1146	0.1210	0.1591
32STYR	R3	128	14.032	7.938	6.755	-0.1450	0.1548	0.1720
33STYR	B	129	14.406	8.016	6.506	-0.1074	0.1737	0.1895
33STYR	R1	130	14.362	7.863	6.278	-0.0880	0.1403	0.2189
33STYR	R2	131	14.301	7.607	6.221	-0.0735	0.1361	0.2227
33STYR	R3	132	14.484	7.738	6.073	-0.0509	0.1313	0.2461
34STYR	B	133	14.466	8.137	6.716	-0.1267	0.1948	0.1923
34STYR	R1	134	14.623	7.928	6.632	-0.1260	0.1624	0.2318
34STYR	R2	135	14.727	7.697	6.725	-0.1174	0.1807	0.2682
34STYR	R3	136	14.875	7.857	6.566	-0.1154	0.1721	0.2615
35STYR	B	137	14.454	8.342	6.831	-0.1704	0.1607	0.1937
35STYR	R1	138	14.372	8.147	6.975	-0.1847	0.1801	0.1965
35STYR	R2	139	14.160	8.090	7.132	-0.2021	0.1683	0.1687
35STYR	R3	140	14.408	8.020	7.211	-0.2085	0.1819	0.2011
36STYR	B	141	14.376	8.471	7.024	-0.1981	0.1945	0.1705
36STYR	R1	142	14.611	8.537	6.903	-0.1847	0.1785	0.2235
36STYR	R2	143	14.871	8.586	6.959	-0.1899	0.1863	0.2409
36STYR	R3	144	14.724	8.770	6.828	-0.1859	0.1805	0.2280
37STYR	B	145	14.186	8.610	7.098	-0.2065	0.1891	0.1620
37STYR	R1	146	14.315	8.488	7.300	-0.2225	0.1802	0.1684
37STYR	R2	147	14.302	8.309	7.502	-0.2397	0.1913	0.1772
37STYR	R3	148	14.410	8.553	7.545	-0.2867	0.2106	0.1855
38STYR	B	149	14.023	8.591	7.277	-0.2460	0.1637	0.1414
38STYR	R1	150	13.941	8.716	7.056	-0.2063	0.1534	0.1089
38STYR	R2	151	13.751	8.704	6.865	-0.2027	0.1254	0.1071
38STYR	R3	152	13.805	8.939	6.987	-0.2324	0.1352	0.1015
39STYR	B	153	13.896	8.622	7.480	-0.2509	0.1721	0.1209
39STYR	R1	154	13.889	8.381	7.371	-0.2294	0.1668	0.1349
39STYR	R2	155	13.890	8.128	7.466	-0.2308	0.1663	0.1335
39STYR	R3	156	13.755	8.187	7.240	-0.2109	0.1635	0.1209
40STYR	B	157	13.937	8.730	7.686	-0.2942	0.1766	0.1178
40STYR	R1	158	14.044	8.837	7.480	-0.2754	0.1741	0.1369
40STYR	R2	159	14.053	9.083	7.369	-0.2793	0.1644	0.1148
40STYR	R3	160	14.280	8.938	7.396	-0.2737	0.1790	0.1476
41STYR	B	161	13.891	8.859	7.880	-0.3289	0.1837	0.0910
41STYR	R1	162	14.145	8.810	7.839	-0.3110	0.2096	0.1489
41STYR	R2	163	14.389	8.923	7.811	-0.3102	0.2116	0.1638
41STYR	R3	164	14.352	8.725	7.990	-0.3228	0.2210	0.1717
42STYR	B	165	13.974	9.072	7.947	-0.3406	0.1651	0.1042
42STYR	R1	166	13.847	8.950	8.136	-0.3784	0.2098	0.1047
42STYR	R2	167	13.648	8.982	8.316	-0.3966	0.1765	0.0908
42STYR	R3	168	13.889	8.890	8.396	-0.3849	0.2222	0.1086
43STYR	B	169	13.914	9.280	8.053	-0.3766	0.1821	0.1071
43STYR	R1	170	14.127	9.276	7.883	-0.3576	0.1958	0.1256
43STYR	R2	171	14.216	9.365	7.644	-0.3385	0.1917	0.1312
43STYR	R3	172	14.387	9.340	7.851	-0.3579	0.2093	0.1494
44STYR	B	173	13.666	9.360	8.079	-0.3955	0.1586	0.0388
44STYR	R1	174	13.850	9.379	8.294	-0.4022	0.2091	0.0747
44STYR	R2	175	13.942	9.551	8.482	-0.4428	0.2332	0.0726
44STYR	R3	176	13.853	9.306	8.554	-0.4060	0.2214	0.0782
45STYR	B	177	13.494	9.225	7.964	-0.3670	0.1772	0.0450
45STYR	R1	178	13.394	9.403	8.118	-0.3746	0.1638	0.0391
45STYR	R2	179	13.207	9.397	8.313	-0.4211	0.1639	-0.0050
45STYR	R3	180	13.196	9.587	8.121	-0.3842	0.1546	-0.0165
46STYR	B	181	13.495	9.033	7.837	-0.3347	0.1727	0.0663
46STYR	R1	182	13.301	9.045	8.001	-0.3543	0.1552	0.0279
46STYR	R2	183	13.031	9.039	8.020	-0.3560	0.1364	-0.0022
46STYR	R3	184	13.182	8.902	8.197	-0.3659	0.1575	0.0225
47STYR	B	185	13.410	8.821	7.796	-0.2966	0.1507	0.0613
47STYR	R1	186	13.488	8.963	7.588	-0.2929	0.1346	0.0531
47STYR	R2	187	13.389	9.060	7.357	-0.2829	0.1363	0.0496
47STYR	R3	188	13.644	8.971	7.368	-0.2826	0.1385	0.0605
48STYR	B	189	13.487	8.611	7.701	-0.2838	0.1488	0.0703
48STYR	R1	190	13.479	8.631	7.972	-0.3286	0.1485	0.0618
48STYR	R2	191	13.620	8.585	8.198	-0.3552	0.2133	0.0919
48STYR	R3	192	13.366	8.495	8.177	-0.3361	0.1642	0.0681
49STYR	B	193	13.624	8.433	7.776	-0.2962	0.1429	0.1026
49STYR	R1	194	13.430	8.357	7.624	-0.2640	0.1312	0.0925
49STYR	R2	195	13.380	8.199	7.411	-0.2304	0.1224	0.0913
49STYR	R3	196	13.235	8.171	7.637	-0.2637	0.1295	0.0707
50STYR	B	197	13.638	8.209	7.855	-0.2857	0.1811	0.1331
50STYR	R1	198	13.851	8.359	7.900	-0.3037	0.1794	0.1229
50STYR	R2	199	14.120	8.360	7.869	-0.2979	0.1960	0.1731

50STYR	R3	200	14.010	8.455	8.096	-0.3470	0.1993	0.1482
1STYR	B	201	11.951	9.076	5.659	-0.0810	-0.0266	-0.1316
1STYR	R1	202	11.715	9.123	5.545	-0.0642	-0.0459	-0.1588
1STYR	R2	203	11.497	9.280	5.573	-0.0770	-0.0566	-0.1979
1STYR	R3	204	11.468	9.018	5.516	-0.0547	-0.0650	-0.1704
2STYR	B	205	12.042	9.266	5.769	-0.0920	-0.0143	-0.1291
2STYR	R1	206	11.976	9.059	5.929	-0.1139	-0.0123	-0.1246
2STYR	R2	207	12.068	8.888	6.115	-0.1220	0.0017	-0.1077
2STYR	R3	208	11.832	9.012	6.152	-0.1261	0.0005	-0.1298
3STYR	B	209	12.197	9.460	5.755	-0.1158	-0.0152	-0.1119
3STYR	R1	210	11.939	9.505	5.834	-0.1388	-0.0397	-0.1435
3STYR	R2	211	11.713	9.640	5.773	-0.1322	-0.0444	-0.1785
3STYR	R3	212	11.808	9.642	6.026	-0.1670	-0.0359	-0.1655
4STYR	B	213	12.268	9.607	5.936	-0.1390	0.0123	-0.1155
4STYR	R1	214	12.475	9.522	5.808	-0.1254	-0.0116	-0.1040
4STYR	R2	215	12.700	9.635	5.710	-0.1216	0.0137	-0.0663
4STYR	R3	216	12.713	9.399	5.841	-0.1140	0.0261	-0.0445
5STYR	B	217	12.436	9.648	6.092	-0.1811	0.0137	-0.1062
5STYR	R1	218	12.232	9.493	6.168	-0.1568	0.0072	-0.1130
5STYR	R2	219	12.034	9.477	6.351	-0.1827	0.0008	-0.1414
5STYR	R3	220	12.177	9.256	6.286	-0.1678	0.0049	-0.1227
6STYR	B	221	12.503	9.725	6.302	-0.1910	0.0335	-0.0903
6STYR	R1	222	12.348	9.884	6.156	-0.1798	0.0147	-0.1030
6STYR	R2	223	12.109	10.009	6.152	-0.1898	-0.0057	-0.1610
6STYR	R3	224	12.334	10.144	6.087	-0.1887	0.0065	-0.1324
7STYR	B	225	12.660	9.742	6.482	-0.2405	0.0485	-0.0797
7STYR	R1	226	12.426	9.633	6.536	-0.2081	0.0509	-0.0910
7STYR	R2	227	12.340	9.409	6.661	-0.2051	0.0429	-0.1032
7STYR	R3	228	12.220	9.646	6.710	-0.2278	0.0335	-0.1129
8STYR	B	229	12.862	9.838	6.541	-0.2449	0.0464	-0.0592
8STYR	R1	230	12.804	9.847	6.284	-0.2005	0.0461	-0.0650
8STYR	R2	231	12.771	10.021	6.080	-0.1925	0.0358	-0.0751
8STYR	R3	232	12.903	9.789	6.040	-0.1615	0.0490	-0.0499
9STYR	B	233	12.964	10.039	6.597	-0.2536	0.0600	-0.0494
9STYR	R1	234	12.695	10.023	6.614	-0.2491	0.0573	-0.0707
9STYR	R2	235	12.479	10.151	6.515	-0.2472	0.0315	-0.1087
9STYR	R3	236	12.480	10.068	6.771	-0.2805	0.0355	-0.1072
10STYR	B	237	13.155	10.167	6.664	-0.2592	0.0922	-0.0532
10STYR	R1	238	12.974	10.270	6.488	-0.2549	0.0629	-0.0680
10STYR	R2	239	12.801	10.476	6.464	-0.2625	0.0525	-0.1022
10STYR	R3	240	12.943	10.393	6.249	-0.2190	0.0574	-0.0756
11STYR	B	241	13.295	10.210	6.861	-0.2994	0.0686	-0.0047
11STYR	R1	242	13.424	10.158	6.638	-0.2548	0.1092	-0.0208
11STYR	R2	243	13.628	10.001	6.556	-0.2277	0.1091	0.0455
11STYR	R3	244	13.631	10.266	6.504	-0.2354	0.1008	0.0021
12STYR	B	245	13.316	10.203	7.096	-0.3226	0.1314	-0.0134
12STYR	R1	246	13.067	10.220	6.994	-0.3082	0.1030	-0.0321
12STYR	R2	247	12.840	10.365	6.967	-0.3152	0.0848	-0.0703
12STYR	R3	248	12.827	10.103	7.031	-0.3047	0.0861	-0.0629
13STYR	B	249	13.277	10.049	7.275	-0.3188	0.1308	-0.0334
13STYR	R1	250	13.459	10.241	7.322	-0.3368	0.1344	-0.0004
13STYR	R2	251	13.519	10.418	7.517	-0.3703	0.1569	-0.0105
13STYR	R3	252	13.716	10.277	7.398	-0.3501	0.1398	0.0425
14STYR	B	253	13.374	9.850	7.380	-0.3175	0.1537	0.0026
14STYR	R1	254	13.132	9.933	7.466	-0.3323	0.1085	-0.0186
14STYR	R2	255	12.876	9.861	7.514	-0.3373	0.0978	-0.0607
14STYR	R3	256	13.022	9.979	7.708	-0.3823	0.1275	-0.0447
15STYR	B	257	13.635	9.832	7.381	-0.3339	0.1623	0.0346
15STYR	R1	258	13.497	9.888	7.613	-0.3687	0.1484	0.0019
15STYR	R2	259	13.472	10.073	7.808	-0.4219	0.1570	-0.0128
15STYR	R3	260	13.451	9.810	7.867	-0.3567	0.1559	0.0064
16STYR	B	261	13.734	9.894	7.163	-0.3221	0.1423	0.0496
16STYR	R1	262	13.817	9.665	7.261	-0.3047	0.1450	0.0603
16STYR	R2	263	13.834	9.400	7.212	-0.2828	0.1446	0.0697
16STYR	R3	264	14.051	9.536	7.296	-0.3019	0.1594	0.0954
17STYR	B	265	13.617	9.844	6.960	-0.2747	0.1403	0.0264
17STYR	R1	266	13.757	10.077	6.974	-0.2951	0.1301	0.0430
17STYR	R2	267	13.721	10.343	6.941	-0.3266	0.1258	0.0421
17STYR	R3	268	13.939	10.221	6.836	-0.2981	0.1560	0.0661
18STYR	B	269	13.412	9.763	6.891	-0.2722	0.1003	0.0192
18STYR	R1	270	13.606	9.608	6.827	-0.2746	0.0984	0.0458
18STYR	R2	271	13.691	9.352	6.831	-0.2297	0.1134	0.0663
18STYR	R3	272	13.646	9.475	6.595	-0.2082	0.1008	0.0555

19STYR	B	273	13.177	9.781	6.912	-0.2603	0.0891	-0.0149
19STYR	R1	274	13.281	9.721	6.669	-0.2265	0.0892	-0.0060
19STYR	R2	275	13.292	9.790	6.409	-0.2176	0.0779	-0.0087
19STYR	R3	276	13.252	9.532	6.479	-0.2065	0.0788	0.0012
20STYR	B	277	13.031	9.719	7.090	-0.2885	0.0984	-0.0373
20STYR	R1	278	13.232	9.561	7.054	-0.2762	0.0965	0.0018
20STYR	R2	279	13.424	9.442	7.201	-0.2742	0.1270	0.0240
20STYR	R3	280	13.315	9.312	6.992	-0.2559	0.0951	0.0341
21STYR	B	281	12.977	9.516	7.210	-0.2961	0.0929	-0.0127
21STYR	R1	282	12.783	9.678	7.109	-0.2860	0.1007	-0.0496
21STYR	R2	283	12.547	9.791	7.175	-0.3046	0.0709	-0.0646
21STYR	R3	284	12.580	9.681	6.931	-0.2800	0.0622	-0.0574
22STYR	B	285	12.801	9.442	7.366	-0.3097	0.0990	-0.0411
22STYR	R1	286	13.046	9.449	7.466	-0.3259	0.1109	-0.0108
22STYR	R2	287	13.180	9.287	7.635	-0.3325	0.1254	0.0085
22STYR	R3	288	13.175	9.553	7.680	-0.3438	0.1280	-0.0082
23STYR	B	289	12.751	9.216	7.447	-0.2953	0.0974	-0.0472
23STYR	R1	290	12.641	9.296	7.212	-0.2758	0.0939	-0.0613
23STYR	R2	291	12.624	9.234	6.950	-0.2433	0.0725	-0.0584
23STYR	R3	292	12.404	9.318	7.084	-0.2666	0.0545	-0.0851
24STYR	B	293	12.799	9.027	7.591	-0.3107	0.1042	-0.0211
24STYR	R1	294	12.740	9.264	7.709	-0.3224	0.0952	-0.0289
24STYR	R2	295	12.836	9.428	7.901	-0.3654	0.1352	-0.0414
24STYR	R3	296	12.573	9.369	7.894	-0.3585	0.1077	-0.0685
25STYR	B	297	12.991	8.878	7.605	-0.3039	0.1192	0.0004
25STYR	R1	298	12.920	8.962	7.357	-0.2871	0.0964	0.0002
25STYR	R2	299	12.845	8.864	7.117	-0.2313	0.0748	-0.0086
25STYR	R3	300	13.027	9.063	7.131	-0.2617	0.1012	0.0142
26STYR	B	301	13.110	8.726	7.475	-0.2589	0.1252	0.0166
26STYR	R1	302	12.998	8.616	7.674	-0.3011	0.1287	0.0030
26STYR	R2	303	12.837	8.409	7.738	-0.3018	0.1318	0.0111
26STYR	R3	304	13.052	8.464	7.891	-0.2986	0.1337	0.0058
27STYR	B	305	13.069	8.515	7.371	-0.2501	0.1090	0.0264
27STYR	R1	306	13.275	8.640	7.289	-0.2361	0.1077	0.0643
27STYR	R2	307	13.532	8.584	7.225	-0.2319	0.1168	0.0730
27STYR	R3	308	13.388	8.752	7.070	-0.2283	0.1225	0.0758
28STYR	B	309	13.026	8.461	7.144	-0.2117	0.0892	0.0342
28STYR	R1	310	12.972	8.267	7.310	-0.2316	0.1143	0.0336
28STYR	R2	311	13.007	7.999	7.312	-0.1999	0.1185	0.0557
28STYR	R3	312	12.768	8.104	7.383	-0.2215	0.0949	0.0185
29STYR	B	313	12.890	8.348	6.981	-0.1900	0.0931	0.0093
29STYR	R1	314	13.168	8.375	6.953	-0.1930	0.1001	0.0400
29STYR	R2	315	13.408	8.253	6.956	-0.1839	0.1190	0.0831
29STYR	R3	316	13.360	8.449	6.778	-0.1738	0.1022	0.0618
30STYR	B	317	12.694	8.439	6.869	-0.1983	0.0602	-0.0047
30STYR	R1	318	12.667	8.454	7.122	-0.2203	0.0729	-0.0123
30STYR	R2	319	12.460	8.391	7.284	-0.2323	0.0622	-0.0317
30STYR	R3	320	12.632	8.587	7.355	-0.2553	0.0786	-0.0206
31STYR	B	321	12.556	8.625	6.850	-0.1933	0.0618	-0.0330
31STYR	R1	322	12.449	8.391	6.794	-0.1880	0.0519	-0.0353
31STYR	R2	323	12.311	8.247	6.612	-0.1494	0.0226	-0.0419
31STYR	R3	324	12.240	8.240	6.872	-0.1788	0.0315	-0.0496
32STYR	B	325	12.572	8.844	6.763	-0.1799	0.0501	-0.0607
32STYR	R1	326	12.436	8.822	6.989	-0.2141	0.0704	-0.0627
32STYR	R2	327	12.423	8.923	7.239	-0.2643	0.0693	-0.0648
32STYR	R3	328	12.198	8.893	7.093	-0.2389	0.0404	-0.0985
33STYR	B	329	12.646	8.974	6.591	-0.1730	0.0353	-0.0406
33STYR	R1	330	12.821	8.807	6.676	-0.1859	0.0741	-0.0191
33STYR	R2	331	12.980	8.609	6.584	-0.1827	0.0625	0.0118
33STYR	R3	332	13.074	8.784	6.767	-0.1957	0.0732	0.0083
34STYR	B	333	12.584	9.107	6.411	-0.1566	0.0416	-0.0579
34STYR	R1	334	12.392	8.967	6.547	-0.1839	0.0185	-0.0678
34STYR	R2	335	12.154	9.012	6.666	-0.2050	0.0145	-0.1078
34STYR	R3	336	12.191	8.788	6.520	-0.1730	0.0098	-0.0927
35STYR	B	337	12.654	9.316	6.324	-0.1612	0.0303	-0.0693
35STYR	R1	338	12.581	9.127	6.154	-0.1502	0.0232	-0.0548
35STYR	R2	339	12.651	8.994	5.930	-0.1076	0.0311	-0.0463
35STYR	R3	340	12.406	9.106	5.949	-0.1229	0.0030	-0.0760
36STYR	B	341	12.880	9.395	6.250	-0.1708	0.0490	-0.0390
36STYR	R1	342	12.866	9.341	6.511	-0.1966	0.0600	-0.0352
36STYR	R2	343	13.008	9.209	6.699	-0.2135	0.0761	-0.0111
36STYR	R3	344	12.914	9.457	6.750	-0.2420	0.0695	-0.0307
37STYR	B	345	13.052	9.209	6.251	-0.1459	0.0629	0.0001

37STYR	R1	346	13.104	9.414	6.089	-0.1353	0.0494	-0.0011
37STYR	R2	347	13.160	9.461	5.829	-0.1271	0.0554	0.0017
37STYR	R3	348	13.312	9.572	6.022	-0.1512	0.0750	0.0095
38STYR	B	349	13.012	8.976	6.291	-0.1779	0.0764	-0.0037
38STYR	R1	350	13.086	9.058	6.050	-0.1291	0.0541	0.0060
38STYR	R2	351	13.032	9.048	5.786	-0.0928	0.0347	-0.0007
38STYR	R3	352	13.288	9.036	5.873	-0.1038	0.0533	0.0346
39STYR	B	353	12.964	8.785	6.170	-0.1098	0.0626	0.0121
39STYR	R1	354	13.202	8.803	6.279	-0.1451	0.0593	0.0336
39STYR	R2	355	13.461	8.798	6.201	-0.1392	0.0876	0.0513
39STYR	R3	356	13.386	8.672	6.427	-0.1386	0.0902	0.0529
40STYR	B	357	12.965	8.562	6.115	-0.1118	0.0321	0.0181
40STYR	R1	358	12.737	8.663	6.210	-0.1197	0.0383	-0.0164
40STYR	R2	359	12.474	8.697	6.163	-0.1177	0.0204	-0.0408
40STYR	R3	360	12.556	8.591	6.397	-0.1314	0.0402	-0.0271
41STYR	B	361	13.045	8.347	6.046	-0.0880	0.0621	0.0446
41STYR	R1	362	13.143	8.557	5.911	-0.0615	0.0533	0.0424
41STYR	R2	363	13.178	8.670	5.669	-0.0633	0.0322	0.0321
41STYR	R3	364	13.392	8.611	5.822	-0.0630	0.0625	0.0436
42STYR	B	365	13.150	8.181	6.197	-0.0921	0.0458	0.0636
42STYR	R1	366	13.048	8.084	5.967	-0.0706	0.0498	0.0456
42STYR	R2	367	12.880	7.889	5.883	-0.0320	0.0222	0.0322
42STYR	R3	368	13.117	7.926	5.759	-0.0176	0.0452	0.0666
43STYR	B	369	13.157	8.215	6.453	-0.1212	0.0807	0.0517
43STYR	R1	370	13.390	8.227	6.285	-0.1010	0.0746	0.0699
43STYR	R2	371	13.640	8.128	6.260	-0.0908	0.0860	0.1258
43STYR	R3	372	13.588	8.384	6.192	-0.0881	0.0824	0.1103
44STYR	B	373	12.922	8.185	6.555	-0.1409	0.0673	0.0127
44STYR	R1	374	13.100	8.022	6.653	-0.1365	0.0694	0.0479
44STYR	R2	375	13.131	7.758	6.695	-0.1413	0.0729	0.0728
44STYR	R3	376	13.144	7.930	6.902	-0.1370	0.0923	0.0564
45STYR	B	377	12.713	8.188	6.437	-0.1241	0.0535	0.0002
45STYR	R1	378	12.725	8.044	6.667	-0.1402	0.0634	0.0130
45STYR	R2	379	12.615	7.997	6.909	-0.1492	0.0613	0.0085
45STYR	R3	380	12.685	7.790	6.750	-0.1264	0.0626	0.0170
46STYR	B	381	12.649	8.201	6.203	-0.1121	0.0194	0.0056
46STYR	R1	382	12.754	7.969	6.301	-0.0952	0.0581	0.0067
46STYR	R2	383	12.953	7.786	6.291	-0.0831	0.0687	0.0511
46STYR	R3	384	12.696	7.705	6.307	-0.0802	0.0553	0.0306
47STYR	B	385	12.588	8.277	5.979	-0.0708	0.0324	-0.0111
47STYR	R1	386	12.384	8.262	6.158	-0.0919	0.0196	-0.0630
47STYR	R2	387	12.140	8.149	6.182	-0.0794	-0.0025	-0.0409
47STYR	R3	388	12.175	8.410	6.243	-0.1096	0.0150	-0.0982
48STYR	B	389	12.389	8.332	5.851	-0.0696	-0.0040	-0.0458
48STYR	R1	390	12.527	8.121	5.769	-0.0453	0.0150	-0.0221
48STYR	R2	391	12.499	7.861	5.703	-0.0346	0.0100	-0.0074
48STYR	R3	392	12.636	8.028	5.541	-0.0201	0.0011	-0.0046
49STYR	B	393	12.245	8.521	5.808	-0.0610	-0.0203	-0.0643
49STYR	R1	394	12.491	8.522	5.708	-0.0529	0.0059	-0.0366
49STYR	R2	395	12.617	8.544	5.471	-0.0323	0.0085	-0.0253
49STYR	R3	396	12.727	8.652	5.693	-0.0563	0.0145	-0.0163
50STYR	B	397	12.262	8.745	5.745	-0.0844	0.0197	-0.0876
50STYR	R1	398	12.014	8.639	5.749	-0.0694	-0.0236	-0.0917
50STYR	R2	399	11.793	8.627	5.596	-0.0476	-0.0649	-0.1199
50STYR	R3	400	11.771	8.672	5.861	-0.0873	-0.0286	-0.1292
1STYR	B	401	16.281	9.986	5.866	-0.1660	0.2289	0.3381
1STYR	R1	402	16.125	10.124	5.706	-0.1625	0.2187	0.3087
1STYR	R2	403	16.080	10.384	5.650	-0.1681	0.2146	0.2942
1STYR	R3	404	16.139	10.211	5.451	-0.1311	0.2114	0.3080
2STYR	B	405	16.055	9.926	5.862	-0.1540	0.2082	0.2891
2STYR	R1	406	16.172	9.914	6.095	-0.1847	0.2318	0.3196
2STYR	R2	407	16.134	9.995	6.349	-0.2192	0.2545	0.3073
2STYR	R3	408	16.237	9.750	6.300	-0.1981	0.2558	0.3431
3STYR	B	409	15.865	9.789	5.858	-0.1398	0.1908	0.2980
3STYR	R1	410	16.108	9.690	5.775	-0.1231	0.2222	0.3259
3STYR	R2	411	16.292	9.497	5.817	-0.1146	0.2377	0.3587
3STYR	R3	412	16.234	9.575	5.565	-0.0850	0.2242	0.3477
4STYR	B	413	15.628	9.735	5.861	-0.1388	0.1808	0.2708
4STYR	R1	414	15.741	9.883	5.656	-0.1323	0.1884	0.2882
4STYR	R2	415	15.720	10.112	5.515	-0.1203	0.1798	0.2724
4STYR	R3	416	15.802	9.886	5.393	-0.0999	0.1748	0.2955
5STYR	B	417	15.540	9.519	5.937	-0.1366	0.2081	0.2744
5STYR	R1	418	15.761	9.588	6.053	-0.1596	0.2062	0.2903

5STYR	R2	419	15.997	9.478	6.124	-0.1624	0.2249	0.3289
5STYR	R3	420	15.835	9.584	6.313	-0.1916	0.2317	0.3000
6STYR	B	421	15.419	9.322	5.897	-0.1378	0.1729	0.2817
6STYR	R1	422	15.419	9.418	6.152	-0.1679	0.2008	0.2726
6STYR	R2	423	15.461	9.331	6.404	-0.2121	0.1909	0.2767
6STYR	R3	424	15.257	9.500	6.352	-0.2107	0.1831	0.2454
7STYR	B	425	15.441	9.122	5.751	-0.0966	0.1813	0.2924
7STYR	R1	426	15.662	9.273	5.765	-0.1065	0.1890	0.3093
7STYR	R2	427	15.929	9.241	5.794	-0.1068	0.2097	0.3342
7STYR	R3	428	15.832	9.393	5.592	-0.1001	0.1917	0.3175
8STYR	B	429	15.606	8.950	5.725	-0.0885	0.1739	0.3072
8STYR	R1	430	15.493	9.025	5.503	-0.0606	0.1894	0.2870
8STYR	R2	431	15.578	9.104	5.260	-0.0345	0.1549	0.2848
8STYR	R3	432	15.389	8.913	5.281	-0.0298	0.1511	0.2917
9STYR	B	433	15.739	8.793	5.845	-0.0764	0.2100	0.3259
9STYR	R1	434	15.492	8.854	5.934	-0.1014	0.1890	0.2793
9STYR	R2	435	15.404	8.933	6.177	-0.1256	0.1885	0.2708
9STYR	R3	436	15.252	8.769	6.025	-0.1037	0.1765	0.2618
10STYR	B	437	15.754	8.664	6.049	-0.0867	0.2230	0.3465
10STYR	R1	438	15.829	8.920	6.070	-0.1320	0.2331	0.3241
10STYR	R2	439	15.765	9.149	6.198	-0.1461	0.2308	0.3211
10STYR	R3	440	16.021	9.063	6.194	-0.1448	0.2339	0.3430
11STYR	B	441	15.882	8.640	6.252	-0.1167	0.2473	0.3473
11STYR	R1	442	15.629	8.554	6.253	-0.1220	0.1880	0.3187
11STYR	R2	443	15.488	8.328	6.299	-0.1367	0.1997	0.3318
11STYR	R3	444	15.391	8.567	6.379	-0.1334	0.2137	0.2947
12STYR	B	445	16.107	8.641	6.324	-0.1265	0.2565	0.3576
12STYR	R1	446	16.070	8.553	6.083	-0.0877	0.2245	0.3783
12STYR	R2	447	16.132	8.337	5.934	-0.0564	0.2239	0.3920
12STYR	R3	448	16.156	8.584	5.829	-0.0825	0.2212	0.3797
13STYR	B	449	16.315	8.735	6.365	-0.1427	0.2650	0.3990
13STYR	R1	450	16.286	8.473	6.409	-0.1307	0.2650	0.4185
13STYR	R2	451	16.390	8.234	6.337	-0.1185	0.2673	0.4284
13STYR	R3	452	16.327	8.281	6.595	-0.1344	0.2697	0.4241
14STYR	B	453	16.481	8.899	6.392	-0.1667	0.2754	0.4217
14STYR	R1	454	16.403	8.855	6.144	-0.1337	0.2249	0.4039
14STYR	R2	455	16.530	8.809	5.910	-0.1019	0.2416	0.4178
14STYR	R3	456	16.321	8.980	5.919	-0.1113	0.2312	0.3992
15STYR	B	457	16.601	9.033	6.547	-0.1597	0.2604	0.4218
15STYR	R1	458	16.754	8.918	6.372	-0.1427	0.2792	0.4434
15STYR	R2	459	16.977	8.767	6.345	-0.1294	0.2943	0.4678
15STYR	R3	460	16.948	8.992	6.198	-0.1391	0.2800	0.4477
16STYR	B	461	16.734	9.208	6.599	-0.2128	0.2991	0.4239
16STYR	R1	462	16.583	9.217	6.373	-0.1686	0.2770	0.4030
16STYR	R2	463	16.396	9.352	6.233	-0.1696	0.2542	0.3823
16STYR	R3	464	16.608	9.244	6.105	-0.1525	0.2631	0.4030
17STYR	B	465	16.922	9.305	6.715	-0.2001	0.3085	0.4603
17STYR	R1	466	16.870	9.046	6.768	-0.2103	0.3008	0.4540
17STYR	R2	467	16.993	8.805	6.770	-0.2012	0.3056	0.4777
17STYR	R3	468	16.864	8.893	6.991	-0.2389	0.3049	0.4562
18STYR	B	469	16.960	9.564	6.719	-0.2125	0.3054	0.4188
18STYR	R1	470	17.006	9.417	6.483	-0.2022	0.3228	0.4446
18STYR	R2	471	16.966	9.431	6.216	-0.1586	0.2996	0.4367
18STYR	R3	472	17.208	9.360	6.313	-0.1782	0.2950	0.4824
19STYR	B	473	16.807	9.679	6.544	-0.2184	0.2798	0.4145
19STYR	R1	474	16.814	9.810	6.770	-0.2494	0.3199	0.4062
19STYR	R2	475	16.856	10.065	6.848	-0.2813	0.3260	0.4038
19STYR	R3	476	16.643	9.929	6.942	-0.2823	0.3110	0.3797
20STYR	B	477	16.557	9.641	6.578	-0.2395	0.2769	0.3862
20STYR	R1	478	16.653	9.733	6.332	-0.2036	0.2872	0.3992
20STYR	R2	479	16.621	9.685	6.068	-0.1753	0.2737	0.3981
20STYR	R3	480	16.622	9.939	6.161	-0.1959	0.2779	0.3867
21STYR	B	481	16.474	9.506	6.765	-0.2407	0.2898	0.3770
21STYR	R1	482	16.327	9.722	6.718	-0.2315	0.2815	0.3568
21STYR	R2	483	16.212	9.924	6.855	-0.2665	0.2712	0.3430
21STYR	R3	484	16.062	9.768	6.694	-0.2335	0.2551	0.3277
22STYR	B	485	16.339	9.474	6.943	-0.2513	0.2787	0.3732
22STYR	R1	486	16.596	9.472	7.000	-0.2639	0.3078	0.4000
22STYR	R2	487	16.778	9.323	7.132	-0.2763	0.3136	0.4236
22STYR	R3	488	16.772	9.591	7.168	-0.2896	0.3133	0.4230
23STYR	B	489	16.111	9.449	7.004	-0.2566	0.2709	0.3648
23STYR	R1	490	16.190	9.333	6.774	-0.2195	0.2817	0.3533
23STYR	R2	491	16.162	9.106	6.631	-0.2072	0.2690	0.3710

23STYR	R3	492	16.107	9.345	6.518	-0.1863	0.2598	0.3413
24STYR	B	493	15.951	9.540	7.138	-0.2809	0.2768	0.3316
24STYR	R1	494	16.211	9.594	7.192	-0.2978	0.2908	0.3419
24STYR	R2	495	16.367	9.807	7.248	-0.3112	0.2987	0.3495
24STYR	R3	496	16.387	9.582	7.396	-0.3300	0.3111	0.3712
25STYR	B	497	15.720	9.511	7.197	-0.2837	0.2500	0.2997
25STYR	R1	498	15.788	9.475	6.937	-0.2604	0.2497	0.3074
25STYR	R2	499	15.682	9.563	6.704	-0.2290	0.2371	0.2882
25STYR	R3	500	15.747	9.303	6.733	-0.2207	0.2412	0.3066
26STYR	B	501	15.555	9.651	7.284	-0.3123	0.2659	0.2819
26STYR	R1	502	15.800	9.699	7.360	-0.3255	0.2760	0.2973
26STYR	R2	503	15.971	9.706	7.570	-0.3357	0.2805	0.3054
26STYR	R3	504	15.966	9.910	7.393	-0.3328	0.2804	0.3052
27STYR	B	505	15.486	9.872	7.337	-0.3334	0.2461	0.2603
27STYR	R1	506	15.384	9.759	7.117	-0.3025	0.2433	0.2551
27STYR	R2	507	15.355	9.788	6.850	-0.2551	0.2265	0.2480
27STYR	R3	508	15.149	9.698	6.999	-0.2750	0.2158	0.2142
28STYR	B	509	15.326	10.026	7.251	-0.3178	0.2412	0.2560
28STYR	R1	510	15.522	10.140	7.375	-0.3366	0.2514	0.2471
28STYR	R2	511	15.543	10.334	7.561	-0.3696	0.2702	0.2315
28STYR	R3	512	15.723	10.320	7.360	-0.3440	0.2603	0.2551
29STYR	B	513	15.345	10.232	7.136	-0.3253	0.2296	0.2047
29STYR	R1	514	15.118	10.121	7.098	-0.3059	0.2186	0.1795
29STYR	R2	515	14.948	10.053	6.899	-0.2870	0.1929	0.1721
29STYR	R3	516	14.855	10.174	7.122	-0.3105	0.2076	0.1542
30STYR	B	517	15.422	10.299	6.929	-0.3086	0.2326	0.2187
30STYR	R1	518	15.377	10.488	7.108	-0.3296	0.2421	0.1953
30STYR	R2	519	15.251	10.726	7.124	-0.3562	0.2289	0.1834
30STYR	R3	520	15.483	10.688	7.256	-0.3782	0.2446	0.2270
31STYR	B	521	15.438	10.219	6.709	-0.2548	0.2268	0.2323
31STYR	R1	522	15.624	10.141	6.903	-0.3047	0.2448	0.2674
31STYR	R2	523	15.874	10.146	7.005	-0.3119	0.2586	0.2845
31STYR	R3	524	15.749	9.910	6.965	-0.3013	0.2523	0.2885
32STYR	B	525	15.253	10.111	6.570	-0.2516	0.1912	0.2232
32STYR	R1	526	15.191	10.348	6.686	-0.2670	0.2005	0.1970
32STYR	R2	527	15.066	10.570	6.595	-0.2784	0.1864	0.1782
32STYR	R3	528	15.009	10.475	6.841	-0.2959	0.1871	0.1744
33STYR	B	529	15.258	9.993	6.366	-0.2243	0.1972	0.2134
33STYR	R1	530	15.075	9.905	6.521	-0.2408	0.1971	0.1970
33STYR	R2	531	14.809	9.860	6.535	-0.2359	0.1633	0.1825
33STYR	R3	532	14.982	9.672	6.621	-0.2167	0.1913	0.2057
34STYR	B	533	15.242	9.904	6.172	-0.2134	0.1934	0.2332
34STYR	R1	534	15.485	9.890	6.294	-0.2021	0.1917	0.2441
34STYR	R2	535	15.668	9.920	6.491	-0.2447	0.2401	0.2766
34STYR	R3	536	15.730	9.989	6.237	-0.2054	0.2212	0.2810
35STYR	B	537	15.182	9.760	5.997	-0.1511	0.1598	0.2308
35STYR	R1	538	15.225	10.001	5.926	-0.1589	0.1936	0.2339
35STYR	R2	539	15.102	10.184	5.770	-0.1656	0.1606	0.2003
35STYR	R3	540	15.369	10.150	5.752	-0.1612	0.1829	0.2227
36STYR	B	541	15.137	9.710	5.784	-0.1218	0.1493	0.2071
36STYR	R1	542	14.988	9.591	5.980	-0.1558	0.1388	0.1958
36STYR	R2	543	14.719	9.569	5.961	-0.1559	0.1569	0.1744
36STYR	R3	544	14.836	9.599	6.203	-0.1730	0.1477	0.1838
37STYR	B	545	15.081	9.795	5.569	-0.1016	0.1353	0.1933
37STYR	R1	546	15.260	9.617	5.559	-0.0989	0.1653	0.2373
37STYR	R2	547	15.308	9.403	5.401	-0.0636	0.1598	0.2553
37STYR	R3	548	15.501	9.588	5.439	-0.0818	0.1759	0.2691
38STYR	B	549	14.936	9.928	5.436	-0.1082	0.1415	0.1986
38STYR	R1	550	15.188	9.958	5.387	-0.0954	0.1441	0.2190
38STYR	R2	551	15.341	10.174	5.333	-0.0976	0.1478	0.2273
38STYR	R3	552	15.375	9.946	5.193	-0.0756	0.1447	0.2379
39STYR	B	553	14.718	10.020	5.485	-0.1278	0.1235	0.1621
39STYR	R1	554	14.867	10.175	5.339	-0.1128	0.1250	0.1640
39STYR	R2	555	14.972	10.423	5.317	-0.1228	0.1294	0.1664
39STYR	R3	556	14.841	10.312	5.108	-0.0970	0.1165	0.1572
40STYR	B	557	14.661	10.228	5.566	-0.1369	0.1122	0.1330
40STYR	R1	558	14.674	10.047	5.743	-0.1530	0.1376	0.1522
40STYR	R2	559	14.516	9.951	5.940	-0.1648	0.1226	0.1356
40STYR	R3	560	14.780	9.988	5.984	-0.1741	0.1544	0.1656
41STYR	B	561	14.694	10.417	5.701	-0.1718	0.1225	0.1407
41STYR	R1	562	14.520	10.421	5.485	-0.1474	0.1047	0.1091
41STYR	R2	563	14.268	10.471	5.403	-0.1435	0.0973	0.0928
41STYR	R3	564	14.478	10.559	5.257	-0.1367	0.0934	0.1003

42STYR	B	565	14.754	10.504	5.921	-0.1870	0.1502	0.1448
42STYR	R1	566	14.924	10.570	5.729	-0.1844	0.1623	0.1495
42STYR	R2	567	15.191	10.578	5.688	-0.1802	0.1623	0.1763
42STYR	R3	568	15.036	10.784	5.607	-0.1693	0.1721	0.1803
43STYR	B	569	14.888	10.409	6.098	-0.2275	0.1569	0.1625
43STYR	R1	570	14.725	10.587	6.182	-0.2291	0.1615	0.1182
43STYR	R2	571	14.738	10.797	6.352	-0.2471	0.1709	0.1079
43STYR	R3	572	14.537	10.618	6.374	-0.2362	0.1590	0.1117
44STYR	B	573	15.078	10.350	6.180	-0.2200	0.1363	0.1909
44STYR	R1	574	14.868	10.251	6.305	-0.2403	0.1870	0.1585
44STYR	R2	575	14.611	10.177	6.342	-0.2255	0.1300	0.1490
44STYR	R3	576	14.756	10.271	6.549	-0.2464	0.1382	0.1599
45STYR	B	577	15.319	10.381	6.152	-0.2203	0.2040	0.2086
45STYR	R1	578	15.170	10.604	6.165	-0.2521	0.1686	0.1878
45STYR	R2	579	15.178	10.840	6.296	-0.2598	0.1758	0.1754
45STYR	R3	580	15.112	10.833	6.034	-0.2237	0.1638	0.1666
46STYR	B	581	15.484	10.412	6.312	-0.2385	0.2079	0.2110
46STYR	R1	582	15.572	10.388	6.071	-0.2186	0.2153	0.2261
46STYR	R2	583	15.749	10.253	5.919	-0.1842	0.2068	0.2732
46STYR	R3	584	15.740	10.522	5.907	-0.1898	0.2056	0.2473
47STYR	B	585	15.700	10.405	6.394	-0.2591	0.2209	0.2543
47STYR	R1	586	15.543	10.597	6.490	-0.2746	0.2149	0.2225
47STYR	R2	587	15.551	10.864	6.525	-0.2944	0.2167	0.2129
47STYR	R3	588	15.455	10.705	6.721	-0.3076	0.2188	0.2082
48STYR	B	589	15.855	10.315	6.544	-0.2598	0.2420	0.2597
48STYR	R1	590	15.957	10.409	6.321	-0.2324	0.2315	0.2801
48STYR	R2	591	16.120	10.352	6.113	-0.2068	0.2445	0.2964
48STYR	R3	592	16.150	10.584	6.248	-0.2447	0.2509	0.2941
49STYR	B	593	16.063	10.306	6.647	-0.2536	0.2706	0.2870
49STYR	R1	594	15.895	10.488	6.740	-0.2785	0.2567	0.2771
49STYR	R2	595	15.793	10.573	6.975	-0.3167	0.2510	0.2625
49STYR	R3	596	15.884	10.752	6.795	-0.3246	0.2574	0.2649
50STYR	B	597	16.213	10.417	6.795	-0.2925	0.2529	0.3152
50STYR	R1	598	16.324	10.281	6.597	-0.2318	0.2646	0.3356
50STYR	R2	599	16.485	10.297	6.381	-0.2340	0.2576	0.3335
50STYR	R3	600	16.511	10.092	6.555	-0.2324	0.2631	0.3399
1STYR	B	601	10.576	7.265	2.070	0.4410	-0.2797	-0.2093
1STYR	R1	602	10.317	7.306	2.012	0.4549	-0.3082	-0.2437
1STYR	R2	603	10.068	7.219	2.069	0.4531	-0.3136	-0.2598
1STYR	R3	604	10.136	7.278	1.814	0.4683	-0.3123	-0.2554
2STYR	B	605	10.533	7.470	1.946	0.4408	-0.2946	-0.1989
2STYR	R1	606	10.773	7.463	2.072	0.4223	-0.2776	-0.1813
2STYR	R2	607	11.038	7.487	2.025	0.4211	-0.2565	-0.1772
2STYR	R3	608	10.929	7.599	2.246	0.4074	-0.2587	-0.1828
3STYR	B	609	10.616	7.691	1.910	0.4345	-0.2932	-0.2141
3STYR	R1	610	10.429	7.622	1.739	0.4463	-0.3301	-0.2441
3STYR	R2	611	10.381	7.615	1.474	0.4770	-0.3165	-0.2501
3STYR	R3	612	10.186	7.691	1.644	0.4614	-0.3122	-0.2698
4STYR	B	613	10.579	7.904	1.816	0.4477	-0.2937	-0.2342
4STYR	R1	614	10.828	7.848	1.864	0.4297	-0.2730	-0.1831
4STYR	R2	615	11.067	7.874	1.740	0.4333	-0.2709	-0.1758
4STYR	R3	616	11.045	7.927	2.004	0.4181	-0.2413	-0.1829
5STYR	B	617	10.654	8.127	1.788	0.4224	-0.2974	-0.2412
5STYR	R1	618	10.426	8.058	1.666	0.4593	-0.3086	-0.2568
5STYR	R2	619	10.315	8.059	1.419	0.4741	-0.3160	-0.2635
5STYR	R3	620	10.167	8.126	1.635	0.4548	-0.3324	-0.2717
6STYR	B	621	10.838	8.288	1.834	0.4125	-0.2977	-0.2208
6STYR	R1	622	10.781	8.208	1.576	0.4635	-0.2988	-0.2333
6STYR	R2	623	10.874	8.076	1.360	0.4810	-0.3067	-0.2209
6STYR	R3	624	10.733	8.304	1.328	0.4622	-0.3213	-0.2417
7STYR	B	625	10.874	8.385	2.056	0.3970	-0.2593	-0.2195
7STYR	R1	626	11.099	8.347	1.921	0.4020	-0.2497	-0.2023
7STYR	R2	627	11.326	8.482	1.863	0.4102	-0.2552	-0.1829
7STYR	R3	628	11.338	8.223	1.941	0.3986	-0.2506	-0.1660
8STYR	B	629	10.719	8.454	2.225	0.3442	-0.2521	-0.2333
8STYR	R1	630	10.792	8.209	2.237	0.3614	-0.2731	-0.2329
8STYR	R2	631	10.665	7.972	2.262	0.3721	-0.2762	-0.2084
8STYR	R3	632	10.890	8.021	2.403	0.3507	-0.2383	-0.1871
9STYR	B	633	10.597	8.682	2.216	0.3383	-0.2737	-0.2756
9STYR	R1	634	10.874	8.668	2.220	0.3447	-0.2643	-0.2403
9STYR	R2	635	11.095	8.767	2.102	0.3561	-0.2565	-0.2124
9STYR	R3	636	11.075	8.765	2.371	0.3111	-0.2327	-0.2155
10STYR	B	637	10.652	8.802	2.022	0.3525	-0.2809	-0.2511

10STYR	R1	638	10.456	8.632	2.010	0.3832	-0.2878	-0.2772
10STYR	R2	639	10.355	8.394	1.933	0.4016	-0.2978	-0.2702
10STYR	R3	640	10.207	8.618	1.905	0.4048	-0.3026	-0.3268
11STYR	B	641	10.685	8.759	1.799	0.4002	-0.3030	-0.2666
11STYR	R1	642	10.760	8.992	1.917	0.3532	-0.2647	-0.2435
11STYR	R2	643	10.976	9.145	1.968	0.3534	-0.2678	-0.2348
11STYR	R3	644	10.736	9.261	1.928	0.3645	-0.2619	-0.2849
12STYR	B	645	10.859	8.675	1.655	0.4185	-0.2772	-0.2366
12STYR	R1	646	10.597	8.651	1.580	0.4238	-0.3056	-0.2607
12STYR	R2	647	10.449	8.686	1.357	0.4373	-0.3165	-0.2714
12STYR	R3	648	10.399	8.478	1.522	0.4378	-0.3177	-0.2728
13STYR	B	649	11.083	8.782	1.638	0.4207	-0.2617	-0.2329
13STYR	R1	650	11.067	8.553	1.513	0.4271	-0.2722	-0.2018
13STYR	R2	651	11.202	8.320	1.491	0.4568	-0.2576	-0.1765
13STYR	R3	652	11.135	8.459	1.269	0.4696	-0.2728	-0.1899
14STYR	B	653	11.113	9.023	1.572	0.3986	-0.2873	-0.2332
14STYR	R1	654	11.310	8.890	1.719	0.3825	-0.2552	-0.2080
14STYR	R2	655	11.568	8.813	1.713	0.3848	-0.2506	-0.1706
14STYR	R3	656	11.461	8.906	1.942	0.3536	-0.2399	-0.1895
15STYR	B	657	10.909	9.154	1.532	0.3989	-0.2889	-0.2539
15STYR	R1	658	10.960	8.979	1.345	0.4211	-0.2930	-0.2408
15STYR	R2	659	10.968	8.978	1.075	0.4632	-0.3065	-0.2395
15STYR	R3	660	10.839	8.780	1.207	0.4518	-0.3056	-0.2494
16STYR	B	661	10.681	9.122	1.496	0.4005	-0.3188	-0.2808
16STYR	R1	662	10.779	9.370	1.516	0.3847	-0.2909	-0.2802
16STYR	R2	663	10.801	9.610	1.392	0.3895	-0.2900	-0.2775
16STYR	R3	664	10.729	9.598	1.652	0.3598	-0.2930	-0.2858
17STYR	B	665	10.446	9.088	1.564	0.4058	-0.3242	-0.2927
17STYR	R1	666	10.520	9.119	1.296	0.4213	-0.3143	-0.2943
17STYR	R2	667	10.471	9.273	1.080	0.4423	-0.3219	-0.3044
17STYR	R3	668	10.490	9.005	1.053	0.4587	-0.3217	-0.2954
18STYR	B	669	10.343	9.045	1.779	0.3974	-0.2989	-0.3213
18STYR	R1	670	10.196	8.963	1.595	0.4008	-0.3336	-0.3245
18STYR	R2	671	9.939	8.996	1.519	0.4136	-0.3443	-0.3730
18STYR	R3	672	10.047	8.749	1.525	0.4171	-0.3419	-0.3339
19STYR	B	673	10.320	9.091	2.004	0.3340	-0.2989	-0.3109
19STYR	R1	674	10.275	9.284	1.830	0.3597	-0.3163	-0.3387
19STYR	R2	675	10.332	9.541	1.768	0.3472	-0.3154	-0.3463
19STYR	R3	676	10.076	9.457	1.773	0.3526	-0.3333	-0.3656
20STYR	B	677	10.283	9.022	2.223	0.3175	-0.2667	-0.3199
20STYR	R1	678	10.077	9.022	2.051	0.3452	-0.3141	-0.3368
20STYR	R2	679	9.878	8.877	1.941	0.3626	-0.3162	-0.3656
20STYR	R3	680	9.839	9.138	1.997	0.3482	-0.3193	-0.3610
21STYR	B	681	10.328	9.083	2.449	0.2864	-0.2726	-0.3113
21STYR	R1	682	10.334	9.284	2.269	0.3039	-0.2812	-0.3311
21STYR	R2	683	10.473	9.494	2.173	0.3001	-0.2766	-0.3264
21STYR	R3	684	10.210	9.520	2.227	0.2941	-0.2894	-0.3488
22STYR	B	685	10.349	9.272	2.589	0.2563	-0.2525	-0.3052
22STYR	R1	686	10.104	9.189	2.520	0.2740	-0.2802	-0.3585
22STYR	R2	687	9.866	9.154	2.642	0.2707	-0.2896	-0.3676
22STYR	R3	688	9.878	9.277	2.402	0.2731	-0.3058	-0.3758
23STYR	B	689	10.341	9.314	2.827	0.2380	-0.2623	-0.3197
23STYR	R1	690	10.231	9.495	2.678	0.2427	-0.2625	-0.3454
23STYR	R2	691	10.219	9.763	2.649	0.2208	-0.2652	-0.3618
23STYR	R3	692	9.991	9.619	2.661	0.2330	-0.2867	-0.3871
24STYR	B	693	10.405	9.178	3.009	0.2251	-0.2463	-0.3278
24STYR	R1	694	10.172	9.289	3.039	0.1996	-0.2460	-0.3639
24STYR	R2	695	10.039	9.426	3.230	0.1874	-0.2582	-0.3636
24STYR	R3	696	9.915	9.229	3.094	0.2039	-0.2667	-0.3663
25STYR	B	697	10.543	9.008	3.073	0.2041	-0.2414	-0.2839
25STYR	R1	698	10.665	9.220	3.011	0.2063	-0.2196	-0.2777
25STYR	R2	699	10.861	9.376	3.112	0.1907	-0.2021	-0.2741
25STYR	R3	700	10.884	9.276	2.862	0.2224	-0.2330	-0.2589
26STYR	B	701	10.563	8.790	3.082	0.2485	-0.2266	-0.2721
26STYR	R1	702	10.324	8.880	3.014	0.2277	-0.2671	-0.3045
26STYR	R2	703	10.137	8.881	2.820	0.2535	-0.2834	-0.3296
26STYR	R3	704	10.065	8.835	3.076	0.2201	-0.2673	-0.3360
27STYR	B	705	10.650	8.586	3.057	0.2641	-0.2407	-0.2541
27STYR	R1	706	10.586	8.724	2.846	0.2820	-0.2450	-0.2774
27STYR	R2	707	10.445	8.690	2.618	0.2962	-0.2611	-0.2838
27STYR	R3	708	10.674	8.834	2.615	0.2806	-0.2361	-0.2737
28STYR	B	709	10.531	8.370	3.051	0.2608	-0.2312	-0.2808
28STYR	R1	710	10.483	8.540	3.259	0.2376	-0.2374	-0.2781

28STYR	R2	711	10.276	8.652	3.392	0.2106	-0.2412	-0.3164
28STYR	R3	712	10.483	8.545	3.529	0.2004	-0.2129	-0.2785
29STYR	B	713	10.307	8.357	2.969	0.2573	-0.2546	-0.2735
29STYR	R1	714	10.476	8.151	2.919	0.2854	-0.2427	-0.2597
29STYR	R2	715	10.502	7.883	2.937	0.2939	-0.2406	-0.2412
29STYR	R3	716	10.581	8.012	2.713	0.3264	-0.2490	-0.2348
30STYR	B	717	10.145	8.445	2.820	0.2925	-0.2694	-0.3247
30STYR	R1	718	10.056	8.383	3.068	0.2479	-0.2657	-0.3285
30STYR	R2	719	9.917	8.488	3.274	0.2439	-0.2543	-0.3371
30STYR	R3	720	9.881	8.230	3.205	0.2480	-0.2591	-0.3211
31STYR	B	721	9.929	8.391	2.753	0.3251	-0.2727	-0.3326
31STYR	R1	722	10.041	8.536	2.574	0.3037	-0.2847	-0.3271
31STYR	R2	723	10.094	8.555	2.310	0.3511	-0.2967	-0.3184
31STYR	R3	724	9.978	8.761	2.439	0.3148	-0.2963	-0.3516
32STYR	B	725	9.749	8.329	2.608	0.3178	-0.2743	-0.3481
32STYR	R1	726	9.733	8.533	2.802	0.2905	-0.2942	-0.3629
32STYR	R2	727	9.596	8.625	3.015	0.2510	-0.2899	-0.3900
32STYR	R3	728	9.691	8.799	2.831	0.2569	-0.2961	-0.3928
33STYR	B	729	9.777	8.156	2.425	0.3450	-0.3129	-0.3300
33STYR	R1	730	9.666	8.386	2.353	0.3359	-0.3156	-0.3576
33STYR	R2	731	9.676	8.590	2.178	0.3629	-0.3276	-0.3702
33STYR	R3	732	9.451	8.442	2.200	0.3562	-0.3204	-0.3880
34STYR	B	733	9.889	8.145	2.215	0.3844	-0.3121	-0.3288
34STYR	R1	734	9.665	7.999	2.240	0.3701	-0.3219	-0.3471
34STYR	R2	735	9.549	7.756	2.215	0.4033	-0.3396	-0.3308
34STYR	R3	736	9.434	7.973	2.102	0.3934	-0.3690	-0.3778
35STYR	B	737	10.069	8.101	2.066	0.4103	-0.2960	-0.3181
35STYR	R1	738	9.871	8.241	1.964	0.3970	-0.3290	-0.3401
35STYR	R2	739	9.682	8.236	1.771	0.4125	-0.3448	-0.3551
35STYR	R3	740	9.833	8.457	1.806	0.3896	-0.3311	-0.3412
36STYR	B	741	10.246	7.925	2.085	0.4068	-0.3009	-0.2930
36STYR	R1	742	10.229	8.115	2.276	0.3635	-0.2867	-0.2832
36STYR	R2	743	10.384	8.280	2.423	0.3425	-0.2717	-0.2776
36STYR	R3	744	10.208	8.115	2.545	0.3532	-0.2878	-0.2839
37STYR	B	745	10.159	7.691	2.094	0.4140	-0.3156	-0.2620
37STYR	R1	746	10.321	7.732	2.282	0.4050	-0.2694	-0.2593
37STYR	R2	747	10.531	7.606	2.397	0.3917	-0.2671	-0.2328
37STYR	R3	748	10.342	7.724	2.551	0.3702	-0.2704	-0.2565
38STYR	B	749	9.990	7.634	2.253	0.4226	-0.2924	-0.2913
38STYR	R1	750	9.919	7.672	1.999	0.4301	-0.3207	-0.3130
38STYR	R2	751	9.778	7.550	1.803	0.4359	-0.3395	-0.3055
38STYR	R3	752	9.768	7.819	1.830	0.4353	-0.3369	-0.3317
39STYR	B	753	9.918	7.706	2.477	0.3733	-0.3069	-0.2997
39STYR	R1	754	9.840	7.460	2.396	0.3846	-0.3036	-0.3011
39STYR	R2	755	9.606	7.326	2.373	0.3988	-0.3262	-0.3162
39STYR	R3	756	9.827	7.197	2.459	0.4033	-0.3023	-0.2917
40STYR	B	757	9.914	7.569	2.671	0.3653	-0.2973	-0.3001
40STYR	R1	758	9.930	7.811	2.727	0.3193	-0.2984	-0.2935
40STYR	R2	759	9.792	7.978	2.889	0.3083	-0.2826	-0.3191
40STYR	R3	760	10.062	7.973	2.900	0.3076	-0.2807	-0.3010
41STYR	B	761	10.054	7.512	2.850	0.3359	-0.2817	-0.2750
41STYR	R1	762	9.807	7.433	2.868	0.3488	-0.2893	-0.2993
41STYR	R2	763	9.603	7.434	3.045	0.3240	-0.2979	-0.3277
41STYR	R3	764	9.661	7.209	2.909	0.3614	-0.2993	-0.3095
42STYR	B	765	10.277	7.488	2.911	0.3283	-0.2551	-0.2535
42STYR	R1	766	10.200	7.350	2.705	0.3716	-0.2632	-0.2450
42STYR	R2	767	10.214	7.091	2.628	0.3720	-0.2690	-0.2255
42STYR	R3	768	10.265	7.288	2.450	0.4056	-0.2832	-0.2315
43STYR	B	769	10.496	7.435	2.856	0.3507	-0.2392	-0.2137
43STYR	R1	770	10.387	7.303	3.053	0.3166	-0.2386	-0.2270
43STYR	R2	771	10.342	7.046	3.121	0.3457	-0.2442	-0.2288
43STYR	R3	772	10.424	7.223	3.308	0.2980	-0.2296	-0.2214
44STYR	B	773	10.707	7.364	2.956	0.3208	-0.2167	-0.1954
44STYR	R1	774	10.655	7.280	2.707	0.3684	-0.2580	-0.1816
44STYR	R2	775	10.778	7.265	2.468	0.3941	-0.2525	-0.1688
44STYR	R3	776	10.649	7.050	2.566	0.4052	-0.2625	-0.1759
45STYR	B	777	10.828	7.446	3.149	0.2936	-0.2097	-0.1834
45STYR	R1	778	10.831	7.178	3.096	0.3454	-0.2164	-0.1839
45STYR	R2	779	11.005	6.972	3.088	0.3589	-0.2074	-0.1258
45STYR	R3	780	10.751	6.931	3.171	0.3455	-0.2118	-0.1685
46STYR	B	781	10.807	7.698	3.226	0.2958	-0.2131	-0.2144
46STYR	R1	782	10.893	7.624	2.959	0.3362	-0.2029	-0.1690
46STYR	R2	783	10.855	7.684	2.699	0.3410	-0.2329	-0.1767

46STYR	R3	784	11.104	7.635	2.791	0.3411	-0.2076	-0.1632
47STYR	B	785	10.584	7.721	3.354	0.2589	-0.2104	-0.2015
47STYR	R1	786	10.750	7.924	3.399	0.2480	-0.2082	-0.2008
47STYR	R2	787	10.880	8.058	3.594	0.2251	-0.2003	-0.1910
47STYR	R3	788	10.695	8.184	3.444	0.2456	-0.2052	-0.2206
48STYR	B	789	10.362	7.691	3.303	0.2868	-0.2401	-0.2556
48STYR	R1	790	10.436	7.607	3.540	0.2553	-0.2053	-0.2323
48STYR	R2	791	10.417	7.395	3.706	0.2489	-0.2029	-0.2299
48STYR	R3	792	10.369	7.643	3.799	0.2090	-0.2051	-0.2442
49STYR	B	793	10.138	7.731	3.256	0.2856	-0.2509	-0.2807
49STYR	R1	794	10.264	7.920	3.389	0.2520	-0.2328	-0.2739
49STYR	R2	795	10.273	8.056	3.622	0.2316	-0.2305	-0.2744
49STYR	R3	796	10.277	8.190	3.388	0.2362	-0.2320	-0.2753
50STYR	B	797	9.905	7.744	3.273	0.2692	-0.2628	-0.2968
50STYR	R1	798	10.019	7.506	3.314	0.2889	-0.2526	-0.2748
50STYR	R2	799	10.033	7.303	3.492	0.2854	-0.2530	-0.2750
50STYR	R3	800	9.981	7.251	3.232	0.3088	-0.2540	-0.2795
1STYR	B	801	11.274	7.369	3.163	0.3186	-0.1944	-0.1071
1STYR	R1	802	11.282	7.258	2.928	0.3558	-0.1870	-0.1251
1STYR	R2	803	11.351	7.311	2.672	0.3883	-0.2053	-0.1201
1STYR	R3	804	11.107	7.210	2.727	0.3848	-0.2105	-0.1449
2STYR	B	805	11.243	7.397	3.391	0.2847	-0.1755	-0.1343
2STYR	R1	806	11.451	7.253	3.312	0.2880	-0.1750	-0.1055
2STYR	R2	807	11.552	7.004	3.337	0.3159	-0.1611	-0.0789
2STYR	R3	808	11.718	7.216	3.325	0.2906	-0.1404	-0.0626
3STYR	B	809	11.235	7.374	3.623	0.2508	-0.1752	-0.1252
3STYR	R1	810	11.049	7.254	3.507	0.2827	-0.1804	-0.1381
3STYR	R2	811	10.783	7.217	3.538	0.2819	-0.2024	-0.1708
3STYR	R3	812	10.949	7.004	3.531	0.3010	-0.1884	-0.1457
4STYR	B	813	11.250	7.449	3.847	0.2327	-0.1556	-0.1538
4STYR	R1	814	11.158	7.214	3.831	0.2307	-0.1610	-0.1321
4STYR	R2	815	10.960	7.079	3.954	0.2343	-0.1721	-0.1387
4STYR	R3	816	11.201	6.963	3.920	0.2399	-0.1608	-0.1360
5STYR	B	817	11.258	7.627	4.003	0.2028	-0.1513	-0.1615
5STYR	R1	818	11.360	7.390	4.079	0.2068	-0.1381	-0.1296
5STYR	R2	819	11.337	7.202	4.272	0.1991	-0.1347	-0.1272
5STYR	R3	820	11.578	7.251	4.160	0.2082	-0.1193	-0.1009
6STYR	B	821	11.129	7.816	4.065	0.1771	-0.1530	-0.1715
6STYR	R1	822	11.006	7.636	3.913	0.2033	-0.1619	-0.1644
6STYR	R2	823	10.853	7.621	3.691	0.2294	-0.1706	-0.1819
6STYR	R3	824	10.791	7.473	3.908	0.2237	-0.1880	-0.1954
7STYR	B	825	11.077	7.960	4.243	0.1567	-0.1407	-0.1723
7STYR	R1	826	11.057	7.695	4.287	0.1608	-0.1484	-0.1732
7STYR	R2	827	10.884	7.497	4.349	0.1562	-0.1412	-0.1628
7STYR	R3	828	11.123	7.512	4.474	0.1540	-0.1364	-0.1591
8STYR	B	829	10.939	8.126	4.332	0.1394	-0.1580	-0.1891
8STYR	R1	830	10.828	7.955	4.159	0.1563	-0.1676	-0.2204
8STYR	R2	831	10.625	7.778	4.144	0.1562	-0.1670	-0.2256
8STYR	R3	832	10.673	7.945	3.938	0.1806	-0.1876	-0.2367
9STYR	B	833	10.839	8.333	4.318	0.1233	-0.1798	-0.2210
9STYR	R1	834	11.062	8.288	4.172	0.1331	-0.1504	-0.1858
9STYR	R2	835	11.298	8.407	4.116	0.1323	-0.1384	-0.1642
9STYR	R3	836	11.164	8.275	3.922	0.1690	-0.1668	-0.1704
10STYR	B	837	10.664	8.486	4.335	0.1097	-0.1660	-0.2534
10STYR	R1	838	10.585	8.243	4.347	0.1121	-0.1840	-0.2552
10STYR	R2	839	10.459	8.048	4.486	0.1150	-0.1959	-0.2692
10STYR	R3	840	10.383	8.104	4.233	0.1299	-0.1949	-0.2735
11STYR	B	841	10.601	8.686	4.231	0.1165	-0.1666	-0.2560
11STYR	R1	842	10.634	8.482	4.073	0.1367	-0.1826	-0.2497
11STYR	R2	843	10.496	8.337	3.891	0.1788	-0.2093	-0.2604
11STYR	R3	844	10.758	8.391	3.851	0.1751	-0.1705	-0.2333
12STYR	B	845	10.437	8.764	4.081	0.1231	-0.1946	-0.2778
12STYR	R1	846	10.354	8.642	4.309	0.1053	-0.1989	-0.3019
12STYR	R2	847	10.188	8.675	4.520	0.0746	-0.1828	-0.3283
12STYR	R3	848	10.175	8.450	4.372	0.1043	-0.1993	-0.3060
13STYR	B	849	10.417	8.916	3.906	0.1294	-0.2032	-0.3077
13STYR	R1	850	10.240	8.727	3.901	0.1541	-0.2274	-0.2979
13STYR	R2	851	9.971	8.724	3.877	0.1574	-0.2349	-0.3356
13STYR	R3	852	10.119	8.530	3.761	0.1780	-0.2400	-0.3009
14STYR	B	853	10.354	8.983	3.693	0.1488	-0.2027	-0.3087
14STYR	R1	854	10.307	9.147	3.903	0.1186	-0.2111	-0.3221
14STYR	R2	855	10.094	9.299	3.968	0.0959	-0.2294	-0.3539
14STYR	R3	856	10.344	9.389	4.017	0.0812	-0.2027	-0.3280

15STYR	B	857	10.408	9.083	3.472	0.1711	-0.2422	-0.3001
15STYR	R1	858	10.146	9.036	3.540	0.1716	-0.2241	-0.3308
15STYR	R2	859	9.918	8.913	3.463	0.1937	-0.2506	-0.3542
15STYR	R3	860	9.909	9.164	3.563	0.1559	-0.2458	-0.3692
16STYR	B	861	10.631	9.226	3.474	0.1650	-0.1983	-0.2941
16STYR	R1	862	10.660	8.959	3.519	0.1820	-0.1939	-0.2796
16STYR	R2	863	10.835	8.773	3.430	0.1857	-0.2084	-0.2424
16STYR	R3	864	10.777	8.789	3.693	0.1636	-0.1755	-0.2492
17STYR	B	865	10.703	9.261	3.716	0.1297	-0.1910	-0.2746
17STYR	R1	866	10.504	9.416	3.608	0.1361	-0.1965	-0.3063
17STYR	R2	867	10.247	9.499	3.615	0.1271	-0.2203	-0.3540
17STYR	R3	868	10.447	9.680	3.621	0.1089	-0.2002	-0.3469
18STYR	B	869	10.889	9.191	3.843	0.1173	-0.1910	-0.2513
18STYR	R1	870	10.783	9.422	3.916	0.1085	-0.1777	-0.2784
18STYR	R2	871	10.792	9.692	3.923	0.0779	-0.1765	-0.2858
18STYR	R3	872	10.715	9.553	4.141	0.0559	-0.1776	-0.2943
19STYR	B	873	10.900	9.119	4.069	0.0961	-0.1560	-0.2466
19STYR	R1	874	11.072	9.001	3.914	0.1276	-0.1632	-0.2343
19STYR	R2	875	11.163	8.752	3.863	0.1397	-0.1614	-0.2219
19STYR	R3	876	11.337	8.957	3.893	0.1324	-0.1608	-0.1824
20STYR	B	877	10.789	9.115	4.274	0.0806	-0.1551	-0.2399
20STYR	R1	878	11.025	9.240	4.260	0.0719	-0.1225	-0.2393
20STYR	R2	879	11.117	9.473	4.360	0.0591	-0.1225	-0.2275
20STYR	R3	880	11.276	9.255	4.356	0.0639	-0.1190	-0.2189
21STYR	B	881	10.544	9.129	4.330	0.0517	-0.1710	-0.3061
21STYR	R1	882	10.699	9.273	4.491	0.0634	-0.1628	-0.2867
21STYR	R2	883	10.730	9.527	4.577	0.0319	-0.1598	-0.2840
21STYR	R3	884	10.727	9.324	4.754	-0.0295	-0.1540	-0.2780
22STYR	B	885	10.481	9.009	4.544	0.0575	-0.1784	-0.2995
22STYR	R1	886	10.281	9.082	4.371	0.0613	-0.1881	-0.3088
22STYR	R2	887	10.065	8.979	4.245	0.0911	-0.1981	-0.3519
22STYR	R3	888	10.042	9.207	4.387	0.0509	-0.2018	-0.3523
23STYR	B	889	10.613	8.838	4.654	0.0457	-0.1558	-0.2710
23STYR	R1	890	10.458	8.976	4.811	0.0180	-0.1695	-0.2980
23STYR	R2	891	10.464	9.114	5.043	-0.0193	-0.1451	-0.3114
23STYR	R3	892	10.276	8.924	5.004	-0.0064	-0.1566	-0.3175
24STYR	B	893	10.788	8.696	4.688	0.0492	-0.1515	-0.2449
24STYR	R1	894	10.564	8.601	4.760	0.0716	-0.1614	-0.2729
24STYR	R2	895	10.404	8.384	4.745	0.0773	-0.1662	-0.2650
24STYR	R3	896	10.452	8.500	4.984	0.0379	-0.1289	-0.2749
25STYR	B	897	11.004	8.635	4.744	0.0594	-0.1175	-0.2214
25STYR	R1	898	10.872	8.793	4.911	0.0106	-0.1265	-0.2384
25STYR	R2	899	10.779	8.813	5.164	-0.0120	-0.1279	-0.2466
25STYR	R3	900	10.898	9.025	5.047	-0.0123	-0.1236	-0.2390
26STYR	B	901	11.187	8.508	4.716	0.0585	-0.1148	-0.1817
26STYR	R1	902	10.958	8.398	4.800	0.0611	-0.1341	-0.2192
26STYR	R2	903	10.786	8.196	4.753	0.0743	-0.1430	-0.2293
26STYR	R3	904	10.837	8.273	5.006	0.0303	-0.1186	-0.2277
27STYR	B	905	11.287	8.354	4.555	0.0758	-0.1197	-0.1802
27STYR	R1	906	11.199	8.612	4.472	0.0977	-0.1414	-0.2090
27STYR	R2	907	11.042	8.732	4.288	0.1047	-0.1358	-0.2113
27STYR	R3	908	11.294	8.820	4.329	0.0961	-0.1237	-0.1845
28STYR	B	909	11.462	8.197	4.549	0.0922	-0.1149	-0.1692
28STYR	R1	910	11.252	8.114	4.675	0.0693	-0.1319	-0.1792
28STYR	R2	911	11.176	8.020	4.917	0.0590	-0.1183	-0.1771
28STYR	R3	912	11.094	7.896	4.691	0.0862	-0.1436	-0.1731
29STYR	B	913	11.671	8.104	4.583	0.0975	-0.0878	-0.1303
29STYR	R1	914	11.589	8.146	4.328	0.1156	-0.1044	-0.1224
29STYR	R2	915	11.700	8.245	4.103	0.1364	-0.1207	-0.1192
29STYR	R3	916	11.531	8.035	4.089	0.1713	-0.1491	-0.1154
30STYR	B	917	11.841	7.929	4.548	0.1149	-0.0949	-0.0831
30STYR	R1	918	11.649	7.906	4.750	0.0957	-0.0958	-0.0998
30STYR	R2	919	11.609	7.873	5.015	0.0666	-0.0799	-0.1022
30STYR	R3	920	11.474	7.720	4.838	0.1063	-0.1094	-0.1073
31STYR	B	921	11.939	7.862	4.332	0.1376	-0.1109	-0.0742
31STYR	R1	922	12.098	7.828	4.531	0.1214	-0.0719	-0.0568
31STYR	R2	923	12.264	7.628	4.603	0.1262	-0.0612	-0.0377
31STYR	R3	924	12.345	7.885	4.624	0.1025	-0.0553	-0.0162
32STYR	B	925	11.885	7.763	4.130	0.1611	-0.1148	-0.0623
32STYR	R1	926	11.715	7.723	4.328	0.1505	-0.0987	-0.0778
32STYR	R2	927	11.594	7.531	4.474	0.1434	-0.1072	-0.0950
32STYR	R3	928	11.455	7.748	4.395	0.1404	-0.1149	-0.1109
33STYR	B	929	11.743	7.631	4.004	0.1926	-0.1352	-0.0967

33STYR	R1	930	11.936	7.518	4.156	0.1895	-0.0864	-0.0558
33STYR	R2	931	11.983	7.351	4.363	0.1592	-0.0773	-0.0416
33STYR	R3	932	12.140	7.341	4.144	0.1831	-0.0961	-0.0237
34STYR	B	933	11.648	7.615	3.793	0.2314	-0.1532	-0.0956
34STYR	R1	934	11.905	7.620	3.800	0.2274	-0.1211	-0.0663
34STYR	R2	935	12.123	7.747	3.704	0.2292	-0.1113	-0.0490
34STYR	R3	936	12.118	7.477	3.716	0.2554	-0.1103	-0.0138
35STYR	B	937	11.667	7.587	3.562	0.2637	-0.1370	-0.0817
35STYR	R1	938	11.681	7.369	3.728	0.2468	-0.1346	-0.0909
35STYR	R2	939	11.579	7.121	3.754	0.2618	-0.1403	-0.0868
35STYR	R3	940	11.841	7.164	3.800	0.2515	-0.1169	-0.0507
36STYR	B	941	11.673	7.741	3.378	0.2633	-0.1565	-0.0913
36STYR	R1	942	11.477	7.778	3.562	0.2391	-0.1614	-0.1181
36STYR	R2	943	11.374	7.975	3.714	0.2057	-0.1662	-0.1345
36STYR	R3	944	11.210	7.813	3.573	0.2363	-0.1649	-0.1718
37STYR	B	945	11.838	7.873	3.252	0.2685	-0.1316	-0.0896
37STYR	R1	946	11.676	7.682	3.120	0.3083	-0.1375	-0.1012
37STYR	R2	947	11.700	7.464	2.963	0.3200	-0.1668	-0.0591
37STYR	R3	948	11.527	7.660	2.896	0.3325	-0.1742	-0.1139
38STYR	B	949	12.045	7.996	3.293	0.2517	-0.1355	-0.0719
38STYR	R1	950	11.848	8.026	3.475	0.2341	-0.1289	-0.0848
38STYR	R2	951	11.815	8.031	3.743	0.2200	-0.1218	-0.0867
38STYR	R3	952	11.692	8.214	3.588	0.2063	-0.1425	-0.1004
39STYR	B	953	12.287	7.950	3.362	0.2405	-0.1160	-0.0516
39STYR	R1	954	12.127	7.747	3.245	0.2935	-0.1297	-0.0603
39STYR	R2	955	12.061	7.489	3.288	0.2836	-0.1215	-0.0256
39STYR	R3	956	12.124	7.573	3.039	0.3126	-0.1631	-0.0324
40STYR	B	957	12.416	8.059	3.554	0.2353	-0.0936	-0.0257
40STYR	R1	958	12.281	8.218	3.368	0.2413	-0.1279	-0.0501
40STYR	R2	959	12.115	8.431	3.361	0.2440	-0.1258	-0.0490
40STYR	R3	960	12.325	8.417	3.192	0.2376	-0.1326	-0.0563
41STYR	B	961	12.451	8.282	3.650	0.2012	-0.0872	-0.0409
41STYR	R1	962	12.363	8.098	3.818	0.1886	-0.0798	-0.0318
41STYR	R2	963	12.167	8.071	4.002	0.1706	-0.0822	-0.0513
41STYR	R3	964	12.424	8.023	4.070	0.1661	-0.0643	-0.0217
42STYR	B	965	12.527	8.509	3.620	0.2007	-0.1020	-0.0388
42STYR	R1	966	12.485	8.433	3.870	0.1664	-0.0944	-0.0355
42STYR	R2	967	12.600	8.421	4.114	0.1405	-0.0788	-0.0224
42STYR	R3	968	12.352	8.525	4.087	0.1392	-0.0902	-0.0539
43STYR	B	969	12.594	8.675	3.459	0.1947	-0.0998	-0.0595
43STYR	R1	970	12.694	8.423	3.440	0.2286	-0.0787	-0.0056
43STYR	R2	971	12.716	8.232	3.251	0.2428	-0.0886	0.0059
43STYR	R3	972	12.926	8.287	3.412	0.2257	-0.0912	0.0294
44STYR	B	973	12.706	8.886	3.448	0.1881	-0.0667	-0.0355
44STYR	R1	974	12.648	8.800	3.215	0.2204	-0.0851	-0.0261
44STYR	R2	975	12.769	8.744	2.981	0.2415	-0.1120	-0.0089
44STYR	R3	976	12.560	8.915	2.987	0.2541	-0.0952	-0.0442
45STYR	B	977	12.649	9.117	3.498	0.1795	-0.0815	-0.0412
45STYR	R1	978	12.860	9.077	3.342	0.1942	-0.0778	-0.0144
45STYR	R2	979	12.948	9.162	3.101	0.2169	-0.0888	-0.0100
45STYR	R3	980	13.124	9.108	3.299	0.1971	-0.0633	0.0145
46STYR	B	981	12.435	9.173	3.597	0.1907	-0.0891	-0.0913
46STYR	R1	982	12.597	9.055	3.751	0.1265	-0.0831	-0.0425
46STYR	R2	983	12.694	9.080	4.001	0.1099	-0.0514	-0.0392
46STYR	R3	984	12.604	8.841	3.915	0.1533	-0.0718	-0.0287
47STYR	B	985	12.268	9.027	3.679	0.1601	-0.1132	-0.0853
47STYR	R1	986	12.222	9.284	3.691	0.1436	-0.1053	-0.0940
47STYR	R2	987	12.036	9.465	3.616	0.1414	-0.1234	-0.1322
47STYR	R3	988	12.134	9.468	3.868	0.0945	-0.1096	-0.1139
48STYR	B	989	12.208	8.808	3.714	0.1605	-0.1147	-0.0880
48STYR	R1	990	12.165	8.886	3.477	0.1820	-0.1164	-0.0949
48STYR	R2	991	11.961	8.886	3.300	0.1936	-0.1457	-0.1082
48STYR	R3	992	12.212	8.829	3.217	0.2035	-0.1316	-0.0876
49STYR	B	993	12.031	8.651	3.730	0.1594	-0.1126	-0.0893
49STYR	R1	994	11.993	8.910	3.810	0.1488	-0.1228	-0.1049
49STYR	R2	995	11.819	9.115	3.783	0.1502	-0.1260	-0.1383
49STYR	R3	996	11.914	9.050	4.027	0.1152	-0.1153	-0.1218
50STYR	B	997	12.019	8.430	3.806	0.1807	-0.1037	-0.0913
50STYR	R1	998	11.800	8.574	3.825	0.1662	-0.1046	-0.1181
50STYR	R2	999	11.535	8.549	3.782	0.1746	-0.1386	-0.1514
50STYR	R3	1000	11.625	8.683	3.998	0.1303	-0.1374	-0.1336
1STYR	B	1001	13.871	9.699	7.894	-0.3801	0.1807	0.0938
1STYR	R1	1002	13.784	9.792	8.133	-0.4215	0.2019	0.0543

1STYR	R2	1003	13.637	9.997	8.229	-0.4461	0.1946	0.0323
1STYR	R3	1004	13.586	9.745	8.312	-0.4189	0.1968	0.0559
2STYR	B	1005	13.959	9.764	7.693	-0.3537	0.1834	0.0721
2STYR	R1	1006	13.801	9.549	7.694	-0.3530	0.1832	0.0805
2STYR	R2	1007	13.573	9.449	7.589	-0.3353	0.1592	0.0649
2STYR	R3	1008	13.791	9.291	7.615	-0.3220	0.1807	0.0844
3STYR	B	1009	14.032	9.970	7.591	-0.3557	0.1750	0.0767
3STYR	R1	1010	14.218	9.791	7.609	-0.3479	0.2069	0.1157
3STYR	R2	1011	14.460	9.729	7.711	-0.3598	0.2114	0.1469
3STYR	R3	1012	14.413	9.696	7.448	-0.3249	0.2116	0.1405
4STYR	B	1013	14.109	10.039	7.381	-0.3231	0.1602	0.0813
4STYR	R1	1014	14.116	10.221	7.574	-0.3856	0.1889	0.0661
4STYR	R2	1015	14.029	10.472	7.621	-0.3862	0.1897	0.0609
4STYR	R3	1016	14.282	10.403	7.684	-0.3891	0.1886	0.0720
5STYR	B	1017	14.182	9.974	7.168	-0.3340	0.1698	0.0928
5STYR	R1	1018	14.172	10.235	7.225	-0.3307	0.1762	0.0638
5STYR	R2	1019	14.322	10.459	7.250	-0.3446	0.1829	0.0873
5STYR	R3	1020	14.068	10.476	7.160	-0.3348	0.1728	0.0580
6STYR	B	1021	14.366	9.905	7.046	-0.3105	0.1702	0.1313
6STYR	R1	1022	14.142	9.826	6.951	-0.2748	0.1599	0.0988
6STYR	R2	1023	14.029	9.594	6.870	-0.2399	0.1428	0.0989
6STYR	R3	1024	13.991	9.820	6.727	-0.2371	0.1275	0.0742
7STYR	B	1025	14.576	9.835	6.966	-0.2884	0.1978	0.1547
7STYR	R1	1026	14.469	10.085	6.900	-0.2728	0.1695	0.1058
7STYR	R2	1027	14.524	10.349	6.896	-0.2897	0.1734	0.1254
7STYR	R3	1028	14.360	10.243	6.710	-0.2672	0.1732	0.1056
8STYR	B	1029	14.715	9.645	7.041	-0.2836	0.1879	0.1810
8STYR	R1	1030	14.571	9.610	6.819	-0.2410	0.1680	0.1544
8STYR	R2	1031	14.389	9.431	6.730	-0.2251	0.1595	0.1389
8STYR	R3	1032	14.590	9.512	6.568	-0.2133	0.1665	0.1569
9STYR	B	1033	14.793	9.426	6.977	-0.2465	0.2024	0.1964
9STYR	R1	1034	14.657	9.431	7.199	-0.2866	0.2150	0.1638
9STYR	R2	1035	14.436	9.301	7.283	-0.2899	0.2048	0.1395
9STYR	R3	1036	14.641	9.341	7.453	-0.3156	0.2255	0.1658
10STYR	B	1037	14.843	9.205	7.059	-0.2588	0.1958	0.2091
10STYR	R1	1038	14.790	9.220	6.795	-0.2043	0.1857	0.1910
10STYR	R2	1039	14.889	9.206	6.545	-0.1930	0.1756	0.1960
10STYR	R3	1040	14.645	9.104	6.599	-0.1967	0.1801	0.1885
11STYR	B	1041	15.019	9.060	7.138	-0.2415	0.1976	0.2202
11STYR	R1	1042	14.789	8.992	7.226	-0.2590	0.2023	0.2157
11STYR	R2	1043	14.692	8.922	7.468	-0.2893	0.1975	0.2022
11STYR	R3	1044	14.636	8.771	7.251	-0.2487	0.1928	0.1948
12STYR	B	1045	15.167	8.918	7.047	-0.2256	0.2067	0.2510
12STYR	R1	1046	15.188	9.166	6.969	-0.2481	0.2282	0.2491
12STYR	R2	1047	15.388	9.348	6.967	-0.2463	0.2264	0.2583
12STYR	R3	1048	15.212	9.337	6.762	-0.2350	0.2258	0.2486
13STYR	B	1049	15.280	8.729	7.003	-0.2182	0.2645	0.2853
13STYR	R1	1050	15.392	8.909	7.166	-0.2429	0.2198	0.2973
13STYR	R2	1051	15.528	8.919	7.399	-0.2694	0.2518	0.3115
13STYR	R3	1052	15.602	9.076	7.192	-0.2610	0.2413	0.3065
14STYR	B	1053	15.331	8.557	6.856	-0.1896	0.2182	0.2970
14STYR	R1	1054	15.223	8.771	6.749	-0.1972	0.2081	0.2780
14STYR	R2	1055	15.035	8.814	6.560	-0.1761	0.1829	0.2513
14STYR	R3	1056	15.258	8.966	6.566	-0.1831	0.1925	0.2641
15STYR	B	1057	15.459	8.374	6.763	-0.1521	0.2341	0.3146
15STYR	R1	1058	15.188	8.378	6.726	-0.1657	0.2289	0.2810
15STYR	R2	1059	14.963	8.232	6.751	-0.1492	0.2041	0.2850
15STYR	R3	1060	15.003	8.380	6.529	-0.1484	0.1725	0.2639
16STYR	B	1061	15.688	8.389	6.699	-0.1673	0.2287	0.3439
16STYR	R1	1062	15.628	8.425	6.961	-0.2065	0.2624	0.3376
16STYR	R2	1063	15.677	8.337	7.211	-0.2176	0.2668	0.3414
16STYR	R3	1064	15.678	8.602	7.158	-0.2390	0.2670	0.3418
17STYR	B	1065	15.891	8.492	6.717	-0.1663	0.2404	0.3564
17STYR	R1	1066	15.877	8.278	6.563	-0.1187	0.2455	0.3637
17STYR	R2	1067	15.929	8.190	6.313	-0.1112	0.2373	0.3682
17STYR	R3	1068	15.981	8.032	6.526	-0.1224	0.2423	0.3747
18STYR	B	1069	16.049	8.660	6.752	-0.1762	0.2635	0.3890
18STYR	R1	1070	15.807	8.740	6.696	-0.1950	0.2263	0.3282
18STYR	R2	1071	15.680	8.913	6.532	-0.1685	0.2495	0.3320
18STYR	R3	1072	15.618	8.906	6.795	-0.1970	0.2262	0.3248
19STYR	B	1073	16.280	8.678	6.812	-0.1886	0.2886	0.4006
19STYR	R1	1074	16.131	8.487	6.933	-0.1756	0.2760	0.3881
19STYR	R2	1075	16.091	8.404	7.187	-0.2287	0.2922	0.3851

19STYR	R3	1076	16.123	8.223	6.990	-0.1884	0.2796	0.4032
20STYR	B	1077	16.426	8.842	6.890	-0.2019	0.2885	0.4062
20STYR	R1	1078	16.534	8.630	6.776	-0.1920	0.3006	0.4211
20STYR	R2	1079	16.718	8.441	6.831	-0.1973	0.3050	0.4543
20STYR	R3	1080	16.695	8.538	6.580	-0.1436	0.3160	0.4535
21STYR	B	1081	16.445	9.040	7.011	-0.2368	0.2887	0.3973
21STYR	R1	1082	16.466	8.819	7.147	-0.2346	0.3091	0.4195
21STYR	R2	1083	16.365	8.692	7.363	-0.2546	0.2918	0.3999
21STYR	R3	1084	16.632	8.702	7.325	-0.2496	0.3229	0.4425
22STYR	B	1085	16.388	9.135	7.219	-0.2663	0.3108	0.3710
22STYR	R1	1086	16.200	9.017	7.089	-0.2302	0.2844	0.3669
22STYR	R2	1087	15.952	9.049	6.988	-0.2184	0.2741	0.3342
22STYR	R3	1088	16.008	8.836	7.145	-0.2352	0.2869	0.3578
23STYR	B	1089	16.208	9.172	7.369	-0.2989	0.2704	0.3580
23STYR	R1	1090	16.445	9.131	7.473	-0.2931	0.3139	0.3903
23STYR	R2	1091	16.614	9.248	7.649	-0.3345	0.3347	0.4165
23STYR	R3	1092	16.558	8.985	7.670	-0.3224	0.3325	0.4209
24STYR	B	1093	15.986	9.214	7.424	-0.2837	0.2868	0.3373
24STYR	R1	1094	16.144	9.273	7.614	-0.3014	0.3088	0.3441
24STYR	R2	1095	16.227	9.460	7.791	-0.3531	0.3206	0.3562
24STYR	R3	1096	16.174	9.209	7.875	-0.3383	0.3154	0.3500
25STYR	B	1097	15.809	9.210	7.576	-0.3152	0.2903	0.3211
25STYR	R1	1098	15.961	8.987	7.546	-0.3022	0.2774	0.3459
25STYR	R2	1099	15.932	8.720	7.524	-0.2710	0.2734	0.3540
25STYR	R3	1100	16.120	8.822	7.689	-0.3060	0.3007	0.3772
26STYR	B	1101	15.583	9.302	7.585	-0.3159	0.2609	0.2907
26STYR	R1	1102	15.726	9.316	7.811	-0.3517	0.2922	0.3102
26STYR	R2	1103	15.831	9.477	8.000	-0.3605	0.3007	0.3078
26STYR	R3	1104	15.701	9.252	8.072	-0.3742	0.3102	0.3125
27STYR	B	1105	15.507	9.498	7.694	-0.3423	0.2665	0.2787
27STYR	R1	1106	15.323	9.379	7.528	-0.3117	0.2637	0.2394
27STYR	R2	1107	15.064	9.302	7.532	-0.3040	0.2375	0.2325
27STYR	R3	1108	15.163	9.434	7.318	-0.3080	0.2352	0.2292
28STYR	B	1109	15.326	9.645	7.681	-0.3483	0.2690	0.2333
28STYR	R1	1110	15.551	9.755	7.773	-0.3686	0.2861	0.2775
28STYR	R2	1111	15.664	9.887	7.980	-0.3880	0.3043	0.2765
28STYR	R3	1112	15.699	9.976	7.727	-0.3808	0.2935	0.2737
29STYR	B	1113	15.269	9.850	7.755	-0.3821	0.2492	0.2288
29STYR	R1	1114	15.100	9.759	7.588	-0.3402	0.2343	0.2037
29STYR	R2	1115	14.840	9.690	7.563	-0.3390	0.2383	0.1792
29STYR	R3	1116	14.961	9.831	7.367	-0.3220	0.2479	0.1966
30STYR	B	1117	15.178	10.064	7.712	-0.3863	0.2718	0.1982
30STYR	R1	1118	15.209	9.971	7.975	-0.4065	0.2732	0.2155
30STYR	R2	1119	15.323	10.043	8.209	-0.4369	0.2817	0.2277
30STYR	R3	1120	15.081	9.923	8.207	-0.4323	0.2727	0.2012
31STYR	B	1121	14.957	10.141	7.774	-0.3797	0.2338	0.1747
31STYR	R1	1122	15.096	10.301	7.613	-0.3832	0.2438	0.1861
31STYR	R2	1123	15.175	10.559	7.601	-0.3945	0.2470	0.1780
31STYR	R3	1124	15.052	10.445	7.389	-0.3690	0.2283	0.1732
32STYR	B	1125	14.768	10.037	7.878	-0.3984	0.2363	0.1685
32STYR	R1	1126	14.864	10.251	8.000	-0.4219	0.2403	0.1656
32STYR	R2	1127	14.961	10.343	8.235	-0.4559	0.2650	0.1701
32STYR	R3	1128	14.785	10.492	8.094	-0.4355	0.2503	0.1288
33STYR	B	1129	14.552	10.110	7.926	-0.4191	0.2406	0.1263
33STYR	R1	1130	14.637	10.133	7.674	-0.3771	0.2065	0.1433
33STYR	R2	1131	14.655	10.325	7.485	-0.3742	0.2100	0.1471
33STYR	R3	1132	14.565	10.079	7.419	-0.3447	0.2024	0.1350
34STYR	B	1133	14.328	10.058	7.967	-0.4149	0.2343	0.1045
34STYR	R1	1134	14.401	10.274	8.080	-0.4496	0.2270	0.1085
34STYR	R2	1135	14.378	10.388	8.324	-0.4742	0.2448	0.0979
34STYR	R3	1136	14.329	10.533	8.101	-0.4458	0.2302	0.0819
35STYR	B	1137	14.211	9.889	8.064	-0.4028	0.2131	0.0877
35STYR	R1	1138	14.082	10.112	8.021	-0.4257	0.1851	0.0768
35STYR	R2	1139	13.946	10.301	8.157	-0.4405	0.1886	0.0570
35STYR	R3	1140	13.853	10.212	7.919	-0.4058	0.1946	0.0412
36STYR	B	1141	14.241	9.656	8.156	-0.4070	0.2152	0.0997
36STYR	R1	1142	14.187	9.878	8.318	-0.4212	0.2340	0.0960
36STYR	R2	1143	14.008	9.982	8.491	-0.4542	0.2339	0.0622
36STYR	R3	1144	14.273	10.001	8.542	-0.4639	0.2314	0.1138
37STYR	B	1145	14.485	9.601	8.199	-0.3976	0.2397	0.1564
37STYR	R1	1146	14.320	9.410	8.275	-0.4206	0.2297	0.1321
37STYR	R2	1147	14.280	9.258	8.495	-0.4223	0.2381	0.1375
37STYR	R3	1148	14.302	9.141	8.253	-0.3778	0.2253	0.1475

38STYR	B	1149	14.683	9.646	8.098	-0.3982	0.2371	0.1698
38STYR	R1	1150	14.631	9.801	8.296	-0.4276	0.2578	0.1367
38STYR	R2	1151	14.723	9.878	8.537	-0.4853	0.2551	0.1599
38STYR	R3	1152	14.653	10.064	8.355	-0.4564	0.2571	0.1507
39STYR	B	1153	14.879	9.605	7.978	-0.3617	0.2630	0.1889
39STYR	R1	1154	14.876	9.533	8.236	-0.4050	0.2702	0.2055
39STYR	R2	1155	15.010	9.568	8.467	-0.4437	0.2879	0.2254
39STYR	R3	1156	14.929	9.321	8.394	-0.3975	0.2766	0.2117
40STYR	B	1157	15.079	9.494	8.003	-0.4008	0.2523	0.2018
40STYR	R1	1158	14.859	9.350	7.959	-0.3599	0.2391	0.1997
40STYR	R2	1159	14.783	9.140	7.808	-0.3438	0.2357	0.1964
40STYR	R3	1160	14.703	9.157	8.066	-0.3841	0.2493	0.1830
41STYR	B	1161	15.205	9.307	7.968	-0.3680	0.2575	0.2343
41STYR	R1	1162	15.299	9.463	8.147	-0.3903	0.2646	0.2556
41STYR	R2	1163	15.400	9.413	8.392	-0.4100	0.2918	0.2694
41STYR	R3	1164	15.498	9.616	8.244	-0.4213	0.2967	0.2686
42STYR	B	1165	15.293	9.116	7.876	-0.3134	0.2611	0.2761
42STYR	R1	1166	15.171	9.069	8.089	-0.3651	0.2690	0.2563
42STYR	R2	1167	15.000	8.884	8.184	-0.3670	0.2679	0.2508
42STYR	R3	1168	15.217	8.954	8.328	-0.3768	0.2758	0.2618
43STYR	B	1169	15.219	8.934	7.745	-0.3119	0.2487	0.2547
43STYR	R1	1170	15.450	8.918	7.881	-0.3388	0.2652	0.2944
43STYR	R2	1171	15.713	8.857	7.865	-0.3317	0.2946	0.2985
43STYR	R3	1172	15.565	8.764	8.071	-0.3194	0.2963	0.3081
44STYR	B	1173	15.129	8.857	7.548	-0.2772	0.2426	0.2515
44STYR	R1	1174	15.012	8.757	7.750	-0.3059	0.2564	0.2567
44STYR	R2	1175	14.762	8.706	7.837	-0.3132	0.2359	0.2239
44STYR	R3	1176	14.953	8.516	7.857	-0.3038	0.2467	0.2360
45STYR	B	1177	15.072	8.668	7.418	-0.2699	0.2366	0.2664
45STYR	R1	1178	15.284	8.635	7.582	-0.2790	0.2475	0.2836
45STYR	R2	1179	15.370	8.476	7.782	-0.2824	0.2662	0.2998
45STYR	R3	1180	15.541	8.551	7.587	-0.2636	0.2969	0.3279
46STYR	B	1181	15.179	8.475	7.341	-0.2556	0.2155	0.2602
46STYR	R1	1182	14.925	8.449	7.394	-0.2426	0.2194	0.2401
46STYR	R2	1183	14.708	8.336	7.279	-0.2272	0.2052	0.2248
46STYR	R3	1184	14.768	8.282	7.537	-0.2541	0.2262	0.2355
47STYR	B	1185	15.165	8.308	7.181	-0.2263	0.2394	0.2748
47STYR	R1	1186	15.274	8.238	7.405	-0.2256	0.2548	0.3108
47STYR	R2	1187	15.231	8.056	7.600	-0.2504	0.2473	0.2984
47STYR	R3	1188	15.483	8.107	7.516	-0.2381	0.2634	0.3448
48STYR	B	1189	15.283	8.130	7.089	-0.2111	0.2322	0.2897
48STYR	R1	1190	15.031	8.081	7.148	-0.2001	0.2206	0.2673
48STYR	R2	1191	14.801	7.982	7.046	-0.1856	0.2009	0.2538
48STYR	R3	1192	14.880	7.912	7.295	-0.2016	0.2189	0.2639
49STYR	B	1193	15.252	7.935	6.965	-0.1744	0.2350	0.3003
49STYR	R1	1194	15.500	7.989	7.038	-0.1779	0.2460	0.3364
49STYR	R2	1195	15.745	8.002	6.925	-0.1644	0.2348	0.3645
49STYR	R3	1196	15.716	7.896	7.172	-0.1944	0.2559	0.3701
50STYR	B	1197	15.205	7.729	6.868	-0.1443	0.2196	0.3124
50STYR	R1	1198	15.301	7.920	6.708	-0.1470	0.2043	0.3115
50STYR	R2	1199	15.253	8.028	6.465	-0.1029	0.2012	0.3014
50STYR	R3	1200	15.506	7.972	6.541	-0.1144	0.2234	0.3571
1STYR	B	1201	11.708	9.627	3.884	0.0932	-0.1221	-0.1956
1STYR	R1	1202	11.883	9.773	4.009	0.0648	-0.1018	-0.1780
1STYR	R2	1203	11.956	9.851	4.257	0.0168	-0.0844	-0.1692
1STYR	R3	1204	11.935	10.033	4.059	0.0458	-0.0969	-0.1839
2STYR	B	1205	11.612	9.629	3.684	0.1189	-0.1449	-0.1834
2STYR	R1	1206	11.455	9.564	3.872	0.0953	-0.1429	-0.2124
2STYR	R2	1207	11.294	9.365	3.958	0.1003	-0.1526	-0.2252
2STYR	R3	1208	11.206	9.620	3.962	0.0884	-0.1568	-0.2226
3STYR	B	1209	11.436	9.689	3.532	0.1415	-0.1523	-0.2161
3STYR	R1	1210	11.645	9.527	3.444	0.1554	-0.1394	-0.1718
3STYR	R2	1211	11.827	9.507	3.245	0.1681	-0.1324	-0.1610
3STYR	R3	1212	11.737	9.284	3.368	0.1815	-0.1348	-0.1554
4STYR	B	1213	11.389	9.675	3.307	0.1626	-0.1659	-0.2044
4STYR	R1	1214	11.371	9.910	3.392	0.1070	-0.1574	-0.2378
4STYR	R2	1215	11.446	10.167	3.351	0.1038	-0.1571	-0.2419
4STYR	R3	1216	11.184	10.100	3.347	0.1058	-0.1641	-0.2605
5STYR	B	1217	11.290	9.536	3.139	0.2023	-0.1888	-0.2275
5STYR	R1	1218	11.126	9.637	3.322	0.1543	-0.1945	-0.2632
5STYR	R2	1219	10.886	9.759	3.335	0.1421	-0.2142	-0.3012
5STYR	R3	1220	10.946	9.561	3.508	0.1506	-0.1746	-0.2587
6STYR	B	1221	11.332	9.319	3.065	0.2004	-0.1895	-0.2068

6STYR	R1	1222	11.284	9.479	2.879	0.2297	-0.2081	-0.2147
6STYR	R2	1223	11.153	9.485	2.643	0.2655	-0.2101	-0.2347
6STYR	R3	1224	11.407	9.577	2.659	0.2581	-0.1965	-0.1938
7STYR	B	1225	11.431	9.124	3.112	0.2176	-0.1654	-0.1867
7STYR	R1	1226	11.279	9.223	3.295	0.1927	-0.1801	-0.2150
7STYR	R2	1227	11.081	9.139	3.459	0.1768	-0.1934	-0.2409
7STYR	R3	1228	11.298	9.263	3.562	0.1411	-0.1519	-0.2156
8STYR	B	1229	11.602	8.980	3.045	0.2282	-0.1759	-0.1620
8STYR	R1	1230	11.484	8.930	3.275	0.1890	-0.1418	-0.1716
8STYR	R2	1231	11.335	8.766	3.430	0.1918	-0.1705	-0.1992
8STYR	R3	1232	11.554	8.887	3.532	0.1749	-0.1634	-0.1713
9STYR	B	1233	11.593	8.760	2.949	0.2472	-0.1642	-0.1429
9STYR	R1	1234	11.790	8.919	2.880	0.2421	-0.1662	-0.1331
9STYR	R2	1235	11.885	9.024	2.650	0.2661	-0.1804	-0.1297
9STYR	R3	1236	12.056	8.930	2.837	0.2476	-0.1452	-0.0948
10STYR	B	1237	11.382	8.641	2.928	0.2401	-0.1983	-0.1912
10STYR	R1	1238	11.438	8.849	2.760	0.2859	-0.1824	-0.1644
10STYR	R2	1239	11.439	8.895	2.494	0.3080	-0.2069	-0.1687
10STYR	R3	1240	11.357	9.088	2.665	0.2814	-0.1957	-0.1940
11STYR	B	1241	11.265	8.500	3.063	0.2390	-0.1933	-0.1972
11STYR	R1	1242	11.517	8.501	3.114	0.2448	-0.1828	-0.1583
11STYR	R2	1243	11.695	8.526	3.316	0.2139	-0.1446	-0.1357
11STYR	R3	1244	11.699	8.307	3.158	0.2596	-0.1590	-0.1144
12STYR	B	1245	11.043	8.442	3.092	0.2599	-0.2030	-0.1931
12STYR	R1	1246	11.092	8.695	3.021	0.2562	-0.1821	-0.2041
12STYR	R2	1247	11.017	8.875	2.834	0.2429	-0.2102	-0.2258
12STYR	R3	1248	11.018	8.944	3.094	0.2169	-0.1852	-0.2322
13STYR	B	1249	10.945	8.219	3.104	0.2702	-0.1897	-0.1975
13STYR	R1	1250	11.087	8.353	3.313	0.2320	-0.1933	-0.2019
13STYR	R2	1251	11.059	8.452	3.563	0.1778	-0.2014	-0.2045
13STYR	R3	1252	11.290	8.334	3.490	0.2166	-0.1425	-0.1782
14STYR	B	1253	10.961	8.060	2.923	0.2975	-0.2159	-0.1836
14STYR	R1	1254	10.917	8.293	2.833	0.2871	-0.2294	-0.2190
14STYR	R2	1255	10.742	8.387	2.650	0.3083	-0.2339	-0.2415
14STYR	R3	1256	10.993	8.486	2.660	0.3030	-0.2237	-0.2057
15STYR	B	1257	11.134	8.066	2.753	0.3303	-0.2164	-0.1743
15STYR	R1	1258	11.193	8.003	3.011	0.2878	-0.2003	-0.1707
15STYR	R2	1259	11.374	8.039	3.209	0.2567	-0.1736	-0.1470
15STYR	R3	1260	11.258	7.798	3.174	0.2802	-0.1847	-0.1480
16STYR	B	1261	11.374	8.091	2.777	0.3065	-0.1846	-0.1469
16STYR	R1	1262	11.294	7.954	2.564	0.3512	-0.2204	-0.1482
16STYR	R2	1263	11.332	7.716	2.443	0.3738	-0.2180	-0.1460
16STYR	R3	1264	11.355	7.944	2.301	0.3770	-0.2162	-0.1425
17STYR	B	1265	11.507	8.272	2.723	0.3147	-0.1944	-0.1713
17STYR	R1	1266	11.631	8.046	2.724	0.3211	-0.1939	-0.1198
17STYR	R2	1267	11.757	7.870	2.563	0.3509	-0.1857	-0.1055
17STYR	R3	1268	11.858	7.926	2.807	0.3244	-0.1723	-0.0977
18STYR	B	1269	11.635	8.419	2.643	0.3194	-0.2051	-0.1175
18STYR	R1	1270	11.399	8.431	2.531	0.3311	-0.1907	-0.1518
18STYR	R2	1271	11.221	8.336	2.352	0.3504	-0.2208	-0.1552
18STYR	R3	1272	11.407	8.516	2.275	0.3471	-0.2199	-0.1610
19STYR	B	1273	11.753	8.588	2.537	0.3072	-0.2044	-0.1216
19STYR	R1	1274	11.767	8.351	2.430	0.3422	-0.1912	-0.1135
19STYR	R2	1275	11.956	8.181	2.339	0.3704	-0.1771	-0.0812
19STYR	R3	1276	11.716	8.194	2.216	0.3855	-0.2052	-0.1136
20STYR	B	1277	11.972	8.631	2.480	0.3120	-0.1894	-0.1051
20STYR	R1	1278	11.922	8.516	2.716	0.2985	-0.1793	-0.1064
20STYR	R2	1279	12.022	8.309	2.858	0.2941	-0.1579	-0.0718
20STYR	R3	1280	11.981	8.548	2.978	0.2619	-0.1482	-0.1018
21STYR	B	1281	12.125	8.770	2.372	0.3329	-0.1559	-0.0884
21STYR	R1	1282	12.216	8.606	2.558	0.3225	-0.1594	-0.0929
21STYR	R2	1283	12.365	8.605	2.783	0.2753	-0.1387	-0.0611
21STYR	R3	1284	12.392	8.406	2.602	0.3205	-0.1501	-0.0422
22STYR	B	1285	12.174	8.876	2.175	0.3339	-0.1741	-0.0872
22STYR	R1	1286	12.005	8.681	2.152	0.3655	-0.1815	-0.1021
22STYR	R2	1287	11.979	8.462	1.998	0.4056	-0.1906	-0.0961
22STYR	R3	1288	11.764	8.616	2.049	0.3812	-0.2175	-0.1164
23STYR	B	1289	12.108	8.936	1.955	0.3752	-0.1832	-0.0873
23STYR	R1	1290	12.368	8.864	2.020	0.3571	-0.1786	-0.0548
23STYR	R2	1291	12.626	8.946	2.014	0.3522	-0.1637	-0.0622
23STYR	R3	1292	12.564	8.690	1.951	0.3653	-0.1678	-0.0588
24STYR	B	1293	11.956	9.066	1.820	0.3840	-0.2143	-0.1316
24STYR	R1	1294	12.030	8.840	1.715	0.4023	-0.2050	-0.0942

24STYR	R2	1295	12.141	8.752	1.485	0.4278	-0.1900	-0.0876
24STYR	R3	1296	11.933	8.619	1.593	0.4243	-0.2048	-0.1123
25STYR	B	1297	11.784	9.212	1.787	0.3424	-0.2244	-0.1735
25STYR	R1	1298	11.836	9.136	2.034	0.3409	-0.2126	-0.1314
25STYR	R2	1299	11.912	9.215	2.281	0.2975	-0.1814	-0.1280
25STYR	R3	1300	11.734	9.014	2.253	0.3192	-0.1997	-0.1342
26STYR	B	1301	11.582	9.329	1.827	0.3583	-0.2275	-0.1709
26STYR	R1	1302	11.614	9.228	1.575	0.3770	-0.2485	-0.1659
26STYR	R2	1303	11.510	9.066	1.385	0.4230	-0.2578	-0.1832
26STYR	R3	1304	11.610	9.308	1.317	0.4064	-0.2460	-0.1656
27STYR	B	1305	11.368	9.378	1.742	0.3649	-0.2547	-0.2173
27STYR	R1	1306	11.500	9.581	1.861	0.3291	-0.2329	-0.2089
27STYR	R2	1307	11.460	9.775	2.044	0.3026	-0.2322	-0.2153
27STYR	R3	1308	11.579	9.834	1.809	0.3158	-0.2285	-0.2078
28STYR	B	1309	11.165	9.500	1.783	0.3493	-0.2665	-0.2372
28STYR	R1	1310	11.246	9.470	1.527	0.3879	-0.2921	-0.2308
28STYR	R2	1311	11.177	9.392	1.278	0.4040	-0.2788	-0.2395
28STYR	R3	1312	11.293	9.632	1.316	0.3948	-0.2778	-0.2182
29STYR	B	1313	11.054	9.580	1.991	0.3114	-0.2666	-0.2343
29STYR	R1	1314	11.100	9.763	1.793	0.3351	-0.2593	-0.2545
29STYR	R2	1315	10.971	9.945	1.641	0.3385	-0.2732	-0.2739
29STYR	R3	1316	11.199	10.013	1.769	0.3112	-0.2483	-0.2385
30STYR	B	1317	10.969	9.535	2.209	0.2885	-0.2439	-0.2704
30STYR	R1	1318	11.208	9.435	2.173	0.3040	-0.2252	-0.2196
30STYR	R2	1319	11.455	9.414	2.281	0.2996	-0.2232	-0.2088
30STYR	R3	1320	11.320	9.192	2.207	0.3026	-0.2246	-0.2103
31STYR	B	1321	10.833	9.620	2.377	0.2797	-0.2704	-0.2886
31STYR	R1	1322	10.844	9.758	2.154	0.2932	-0.2646	-0.2914
31STYR	R2	1323	10.879	10.016	2.081	0.2732	-0.2641	-0.2994
31STYR	R3	1324	10.672	9.874	1.981	0.2904	-0.2866	-0.3035
32STYR	B	1325	10.702	9.542	2.560	0.2653	-0.2419	-0.2890
32STYR	R1	1326	10.814	9.357	2.419	0.2827	-0.2565	-0.2700
32STYR	R2	1327	10.974	9.144	2.463	0.2952	-0.2468	-0.2683
32STYR	R3	1328	10.745	9.115	2.323	0.2956	-0.2618	-0.2657
33STYR	B	1329	10.646	9.638	2.765	0.2236	-0.2457	-0.3056
33STYR	R1	1330	10.647	9.797	2.549	0.2250	-0.2471	-0.3103
33STYR	R2	1331	10.497	9.954	2.387	0.2536	-0.2595	-0.3491
33STYR	R3	1332	10.729	10.047	2.489	0.2342	-0.2508	-0.3127
34STYR	B	1333	10.781	9.795	2.892	0.1948	-0.2428	-0.3127
34STYR	R1	1334	10.551	9.691	2.994	0.1893	-0.2345	-0.3154
34STYR	R2	1335	10.309	9.750	3.099	0.1647	-0.2458	-0.3650
34STYR	R3	1336	10.465	9.564	3.217	0.1571	-0.2254	-0.3224
35STYR	B	1337	11.014	9.836	2.874	0.1940	-0.2150	-0.2741
35STYR	R1	1338	10.878	10.044	2.931	0.1926	-0.2159	-0.2913
35STYR	R2	1339	10.869	10.252	3.103	0.1514	-0.2101	-0.3004
35STYR	R3	1340	10.870	10.297	2.837	0.1769	-0.2203	-0.3021
36STYR	B	1341	11.222	9.926	2.907	0.1794	-0.2047	-0.2397
36STYR	R1	1342	11.145	9.924	2.654	0.1988	-0.2118	-0.2576
36STYR	R2	1343	11.217	9.854	2.403	0.2408	-0.2247	-0.2419
36STYR	R3	1344	11.144	10.108	2.456	0.2083	-0.2277	-0.2724
37STYR	B	1345	11.450	9.941	2.953	0.1841	-0.1930	-0.2245
37STYR	R1	1346	11.318	10.166	2.918	0.1731	-0.1941	-0.2465
37STYR	R2	1347	11.283	10.409	3.031	0.1533	-0.1902	-0.2608
37STYR	R3	1348	11.329	10.388	2.765	0.1932	-0.1996	-0.2532
38STYR	B	1349	11.650	9.814	2.962	0.1704	-0.1783	-0.2254
38STYR	R1	1350	11.679	10.076	2.998	0.1456	-0.1589	-0.2049
38STYR	R2	1351	11.794	10.264	3.154	0.1411	-0.1660	-0.1931
38STYR	R3	1352	11.782	10.299	2.887	0.1750	-0.1674	-0.1948
39STYR	B	1353	11.877	9.845	2.905	0.1966	-0.1628	-0.1671
39STYR	R1	1354	11.688	9.892	2.713	0.2151	-0.1814	-0.1992
39STYR	R2	1355	11.648	9.839	2.451	0.2375	-0.1969	-0.1995
39STYR	R3	1356	11.593	10.084	2.549	0.2224	-0.1932	-0.2173
40STYR	B	1357	12.084	9.730	2.884	0.1945	-0.1533	-0.1643
40STYR	R1	1358	12.038	9.953	2.733	0.2103	-0.1609	-0.1642
40STYR	R2	1359	12.142	10.195	2.672	0.2154	-0.1615	-0.1579
40STYR	R3	1360	12.034	10.042	2.478	0.2281	-0.1617	-0.1648
41STYR	B	1361	12.218	9.533	2.857	0.2303	-0.1378	-0.1035
41STYR	R1	1362	12.342	9.767	2.842	0.2027	-0.1297	-0.0977
41STYR	R2	1363	12.484	9.931	2.682	0.1990	-0.1251	-0.0963
41STYR	R3	1364	12.560	9.894	2.939	0.2020	-0.1285	-0.0976
42STYR	B	1365	12.229	9.315	2.762	0.2432	-0.1442	-0.0884
42STYR	R1	1366	11.991	9.423	2.777	0.2368	-0.1580	-0.1504
42STYR	R2	1367	11.737	9.371	2.854	0.2302	-0.1611	-0.1738

42STYR	R3	1368	11.787	9.457	2.602	0.2530	-0.1664	-0.1711
43STYR	B	1369	12.294	9.189	2.561	0.2883	-0.1367	-0.0849
43STYR	R1	1370	12.468	9.365	2.667	0.2468	-0.1461	-0.0876
43STYR	R2	1371	12.645	9.553	2.588	0.2335	-0.1284	-0.0749
43STYR	R3	1372	12.719	9.365	2.767	0.2406	-0.1233	-0.0725
44STYR	B	1373	12.388	9.241	2.346	0.3039	-0.1499	-0.0956
44STYR	R1	1374	12.494	9.033	2.475	0.2956	-0.1470	-0.0561
44STYR	R2	1375	12.734	8.951	2.568	0.2885	-0.1288	-0.0213
44STYR	R3	1376	12.580	8.782	2.424	0.3268	-0.1370	-0.0528
45STYR	B	1377	12.330	9.296	2.122	0.3060	-0.1679	-0.0734
45STYR	R1	1378	12.571	9.356	2.182	0.3022	-0.1440	-0.0661
45STYR	R2	1379	12.827	9.326	2.101	0.3128	-0.1337	-0.0365
45STYR	R3	1380	12.730	9.572	2.154	0.2967	-0.1376	-0.0479
46STYR	B	1381	12.192	9.415	1.981	0.3318	-0.2015	-0.1167
46STYR	R1	1382	12.255	9.518	2.218	0.3064	-0.1580	-0.1189
46STYR	R2	1383	12.350	9.752	2.313	0.2757	-0.1467	-0.1155
46STYR	R3	1384	12.142	9.632	2.435	0.2806	-0.1673	-0.1274
47STYR	B	1385	12.143	9.513	1.767	0.3309	-0.2043	-0.1210
47STYR	R1	1386	12.334	9.320	1.783	0.3443	-0.1881	-0.0825
47STYR	R2	1387	12.405	9.095	1.651	0.3720	-0.1774	-0.0861
47STYR	R3	1388	12.592	9.279	1.715	0.3522	-0.1635	-0.0674
48STYR	B	1389	11.946	9.600	1.667	0.3634	-0.2208	-0.1515
48STYR	R1	1390	11.961	9.627	1.927	0.3210	-0.2036	-0.1401
48STYR	R2	1391	11.818	9.590	2.153	0.3162	-0.2010	-0.1427
48STYR	R3	1392	11.933	9.830	2.104	0.2702	-0.1845	-0.1700
49STYR	B	1393	11.821	9.641	1.476	0.3551	-0.2186	-0.1842
49STYR	R1	1394	12.009	9.442	1.471	0.3890	-0.2202	-0.1264
49STYR	R2	1395	12.034	9.183	1.399	0.4174	-0.2161	-0.1312
49STYR	R3	1396	12.228	9.359	1.335	0.4090	-0.1983	-0.1075
50STYR	B	1397	11.725	9.820	1.347	0.3830	-0.2418	-0.1923
50STYR	R1	1398	11.917	9.880	1.532	0.3585	-0.2323	-0.1694
50STYR	R2	1399	11.961	10.054	1.734	0.3167	-0.2189	-0.1718
50STYR	R3	1400	12.160	9.995	1.562	0.3394	-0.1962	-0.1533
1STYR	B	1401	14.228	8.876	3.821	0.1581	0.0251	0.1440
1STYR	R1	1402	14.407	8.702	3.912	0.1466	0.0347	0.1734
1STYR	R2	1403	14.416	8.437	3.962	0.1710	0.0376	0.1840
1STYR	R3	1404	14.494	8.613	4.151	0.1175	0.0540	0.1912
2STYR	B	1405	14.073	8.993	3.951	0.1189	0.0166	0.1227
2STYR	R1	1406	14.304	9.114	3.910	0.0939	0.0375	0.1443
2STYR	R2	1407	14.509	9.230	4.042	0.0898	0.0235	0.1628
2STYR	R3	1408	14.401	9.352	3.825	0.1258	0.0248	0.1456
3STYR	B	1409	13.851	9.047	3.998	0.1211	0.0011	0.0961
3STYR	R1	1410	13.900	8.936	3.760	0.1483	-0.0041	0.1156
3STYR	R2	1411	13.873	8.972	3.494	0.1865	-0.0037	0.1117
3STYR	R3	1412	13.802	8.737	3.607	0.1656	-0.0021	0.1020
4STYR	B	1413	13.695	9.152	4.124	0.0773	-0.0062	0.0804
4STYR	R1	1414	13.930	9.267	4.098	0.1020	0.0076	0.1012
4STYR	R2	1415	14.036	9.502	4.020	0.1003	0.0109	0.1089
4STYR	R3	1416	14.144	9.368	4.228	0.0870	0.0162	0.1192
5STYR	B	1417	13.508	9.289	4.130	0.0808	-0.0143	0.0515
5STYR	R1	1418	13.604	9.245	3.887	0.1297	-0.0202	0.0562
5STYR	R2	1419	13.528	9.181	3.636	0.1506	-0.0098	0.0473
5STYR	R3	1420	13.718	9.369	3.676	0.1371	-0.0013	0.0712
6STYR	B	1421	13.379	9.424	4.000	0.0946	-0.0343	0.0286
6STYR	R1	1422	13.255	9.309	4.196	0.0725	-0.0184	0.0148
6STYR	R2	1423	13.099	9.349	4.412	0.0487	-0.0186	-0.0024
6STYR	R3	1424	13.039	9.152	4.237	0.0685	-0.0190	-0.0087
7STYR	B	1425	13.234	9.610	4.008	0.0651	-0.0235	-0.0138
7STYR	R1	1426	13.493	9.658	3.971	0.0926	-0.0219	0.0255
7STYR	R2	1427	13.701	9.809	4.054	0.0653	0.0022	0.0502
7STYR	R3	1428	13.646	9.788	3.790	0.0937	0.0031	0.0442
8STYR	B	1429	13.235	9.804	4.159	0.0437	-0.0204	-0.0213
8STYR	R1	1430	13.129	9.823	3.900	0.0956	-0.0269	-0.0212
8STYR	R2	1431	12.910	9.908	3.765	0.0980	-0.0486	-0.0388
8STYR	R3	1432	13.157	9.960	3.669	0.1001	-0.0539	-0.0367
9STYR	B	1433	13.198	9.787	4.395	0.0318	-0.0205	-0.0382
9STYR	R1	1434	13.383	9.955	4.341	0.0293	-0.0280	0.0059
9STYR	R2	1435	13.630	10.019	4.429	0.0169	0.0095	0.0135
9STYR	R3	1436	13.443	10.211	4.399	-0.0088	-0.0176	-0.0007
10STYR	B	1437	13.046	9.775	4.560	0.0151	-0.0069	-0.0333
10STYR	R1	1438	13.003	9.958	4.379	0.0081	-0.0202	-0.0388
10STYR	R2	1439	12.820	10.069	4.213	0.0281	-0.0320	-0.0689
10STYR	R3	1440	12.977	10.227	4.366	-0.0090	-0.0220	-0.0408

11STYR	B	1441	12.979	9.725	4.784	-0.0239	0.0019	-0.0309
11STYR	R1	1442	13.159	9.930	4.740	-0.0082	-0.0057	-0.0256
11STYR	R2	1443	13.194	10.196	4.773	-0.0346	-0.0001	-0.0430
11STYR	R3	1444	13.400	10.026	4.814	-0.0321	0.0183	0.0208
12STYR	B	1445	12.780	9.622	4.872	-0.0447	-0.0315	-0.0559
12STYR	R1	1446	12.993	9.478	4.885	-0.0306	-0.0111	-0.0163
12STYR	R2	1447	13.112	9.336	5.081	-0.0330	0.0074	-0.0012
12STYR	R3	1448	13.083	9.229	4.834	0.0016	-0.0027	-0.0009
13STYR	B	1449	12.565	9.666	4.932	-0.0293	-0.0327	-0.0890
13STYR	R1	1450	12.710	9.872	4.842	-0.0447	-0.0264	-0.0755
13STYR	R2	1451	12.688	10.070	4.661	-0.0043	-0.0299	-0.0844
13STYR	R3	1452	12.805	10.118	4.899	-0.0405	-0.0302	-0.0665
14STYR	B	1453	12.373	9.781	4.886	-0.0235	-0.0272	-0.1120
14STYR	R1	1454	12.384	9.634	5.112	-0.0553	-0.0332	-0.1089
14STYR	R2	1455	12.242	9.446	5.244	-0.0507	-0.0391	-0.1124
14STYR	R3	1456	12.374	9.637	5.382	-0.0845	-0.0174	-0.1100
15STYR	B	1457	12.246	9.949	4.774	-0.0301	-0.0495	-0.1169
15STYR	R1	1458	12.357	9.773	4.610	0.0024	-0.0449	-0.1145
15STYR	R2	1459	12.299	9.623	4.393	0.0393	-0.0676	-0.1087
15STYR	R3	1460	12.481	9.821	4.375	0.0388	-0.0664	-0.0996
16STYR	B	1461	12.118	10.131	4.892	-0.0286	-0.0510	-0.1640
16STYR	R1	1462	12.217	10.163	4.627	-0.0303	-0.0663	-0.1493
16STYR	R2	1463	12.378	10.317	4.475	-0.0245	-0.0675	-0.1444
16STYR	R3	1464	12.142	10.232	4.377	-0.0120	-0.0842	-0.1598
17STYR	B	1465	12.130	10.094	5.155	-0.0789	-0.0402	-0.1467
17STYR	R1	1466	11.889	10.042	5.012	-0.0548	-0.0550	-0.1812
17STYR	R2	1467	11.693	9.861	4.969	-0.0317	-0.0755	-0.2006
17STYR	R3	1468	11.630	10.115	5.036	-0.0647	-0.0841	-0.1990
18STYR	B	1469	12.350	10.063	5.262	-0.1064	-0.0389	-0.1386
18STYR	R1	1470	12.140	10.032	5.420	-0.1051	-0.0262	-0.1459
18STYR	R2	1471	12.043	9.849	5.593	-0.1299	-0.0322	-0.1660
18STYR	R3	1472	12.048	10.110	5.662	-0.1555	-0.0325	-0.1629
19STYR	B	1473	12.581	10.016	5.250	-0.0853	-0.0121	-0.0871
19STYR	R1	1474	12.489	10.248	5.144	-0.0747	-0.0246	-0.1160
19STYR	R2	1475	12.483	10.394	4.917	-0.0839	-0.0345	-0.1221
19STYR	R3	1476	12.543	10.512	5.152	-0.0984	-0.0194	-0.1260
20STYR	B	1477	12.752	9.859	5.323	-0.0831	0.0110	-0.0655
20STYR	R1	1478	12.603	10.003	5.505	-0.1218	0.0040	-0.0886
20STYR	R2	1479	12.452	9.967	5.726	-0.1397	0.0001	-0.1014
20STYR	R3	1480	12.615	10.181	5.708	-0.1387	0.0008	-0.0847
21STYR	B	1481	12.974	9.789	5.240	-0.0844	0.0178	-0.0181
21STYR	R1	1482	12.825	9.586	5.314	-0.0443	-0.0038	-0.0588
21STYR	R2	1483	12.880	9.360	5.450	-0.0745	0.0070	-0.0285
21STYR	R3	1484	12.708	9.353	5.242	-0.0478	-0.0048	-0.0500
22STYR	B	1485	13.144	9.951	5.195	-0.1101	0.0343	-0.0344
22STYR	R1	1486	13.092	9.896	5.455	-0.0958	0.0406	-0.0353
22STYR	R2	1487	13.021	10.025	5.681	-0.1494	0.0411	-0.0520
22STYR	R3	1488	13.225	9.848	5.684	-0.1403	0.0522	-0.0068
23STYR	B	1489	13.343	10.052	5.277	-0.0882	0.0323	-0.0118
23STYR	R1	1490	13.101	10.191	5.236	-0.0764	0.0158	-0.0517
23STYR	R2	1491	12.906	10.325	5.367	-0.1112	-0.0115	-0.0754
23STYR	R3	1492	12.997	10.417	5.130	-0.0872	0.0076	-0.0587
24STYR	B	1493	13.552	9.956	5.368	-0.0947	0.0438	0.0139
24STYR	R1	1494	13.449	10.177	5.483	-0.1163	0.0496	-0.0041
24STYR	R2	1495	13.378	10.286	5.720	-0.1680	0.0586	-0.0238
24STYR	R3	1496	13.534	10.425	5.549	-0.1503	0.0615	-0.0053
25STYR	B	1497	13.773	9.861	5.351	-0.0950	0.0595	0.0712
25STYR	R1	1498	13.740	10.090	5.238	-0.1040	0.0502	0.0421
25STYR	R2	1499	13.865	10.329	5.241	-0.1122	0.0545	0.0309
25STYR	R3	1500	13.776	10.226	5.008	-0.0896	0.0307	0.0328
26STYR	B	1501	13.869	9.660	5.277	-0.0694	0.0544	0.0652
26STYR	R1	1502	13.636	9.646	5.420	-0.0765	0.0577	0.0638
26STYR	R2	1503	13.548	9.524	5.644	-0.1100	0.0534	0.0483
26STYR	R3	1504	13.392	9.531	5.423	-0.0698	0.0422	0.0195
27STYR	B	1505	13.791	9.480	5.149	-0.0484	0.0510	0.0734
27STYR	R1	1506	13.867	9.686	5.010	-0.0414	0.0562	0.0694
27STYR	R2	1507	14.022	9.752	4.799	-0.0235	0.0356	0.0762
27STYR	R3	1508	13.763	9.829	4.806	-0.0235	0.0380	0.0475
28STYR	B	1509	13.720	9.274	5.088	-0.0316	0.0649	0.0692
28STYR	R1	1510	13.891	9.304	5.296	-0.0560	0.0781	0.0918
28STYR	R2	1511	14.137	9.263	5.399	-0.0695	0.0891	0.1290
28STYR	R3	1512	13.919	9.223	5.552	-0.0912	0.0754	0.0948
29STYR	B	1513	13.511	9.179	5.030	-0.0035	0.0282	0.0384

29STYR	R1	1514	13.523	9.426	4.978	-0.0128	0.0372	0.0432
29STYR	R2	1515	13.398	9.661	5.024	-0.0469	0.0207	0.0354
29STYR	R3	1516	13.417	9.564	4.772	-0.0210	0.0217	0.0369
30STYR	B	1517	13.453	8.947	5.020	-0.0151	0.0388	0.0539
30STYR	R1	1518	13.424	9.084	5.248	-0.0273	0.0427	0.0342
30STYR	R2	1519	13.230	9.115	5.433	-0.0514	0.0157	0.0136
30STYR	R3	1520	13.489	9.107	5.509	-0.0577	0.0594	0.0404
31STYR	B	1521	13.621	8.799	4.922	0.0330	0.0151	0.0991
31STYR	R1	1522	13.465	8.979	4.760	0.0375	0.0272	0.0667
31STYR	R2	1523	13.310	8.958	4.540	0.0471	-0.0137	0.0637
31STYR	R3	1524	13.455	9.182	4.582	0.0342	0.0000	0.0358
32STYR	B	1525	13.624	8.562	4.892	0.0414	0.0287	0.0810
32STYR	R1	1526	13.504	8.647	5.116	-0.0018	0.0383	0.0759
32STYR	R2	1527	13.498	8.582	5.379	-0.0273	0.0417	0.0762
32STYR	R3	1528	13.267	8.626	5.245	-0.0159	0.0105	0.0458
33STYR	B	1529	13.606	8.476	4.649	0.0771	0.0102	0.1011
33STYR	R1	1530	13.725	8.330	4.876	0.0619	0.0316	0.1219
33STYR	R2	1531	13.717	8.110	5.032	0.0464	0.0492	0.1460
33STYR	R3	1532	13.954	8.218	4.963	0.0532	0.0443	0.1617
34STYR	B	1533	13.693	8.673	4.488	0.0918	0.0171	0.1142
34STYR	R1	1534	13.547	8.465	4.374	0.1200	0.0005	0.0734
34STYR	R2	1535	13.349	8.453	4.190	0.1348	-0.0155	0.0585
34STYR	R3	1536	13.565	8.292	4.167	0.1460	-0.0035	0.0789
35STYR	B	1537	13.819	8.885	4.480	0.0770	0.0178	0.0872
35STYR	R1	1538	13.847	8.694	4.282	0.0979	0.0138	0.1041
35STYR	R2	1539	14.051	8.583	4.145	0.1311	0.0343	0.1366
35STYR	R3	1540	13.809	8.609	4.029	0.1380	-0.0204	0.1095
36STYR	B	1541	13.870	9.128	4.527	0.0254	0.0360	0.0922
36STYR	R1	1542	14.082	8.979	4.483	0.0591	0.0297	0.1205
36STYR	R2	1543	14.304	8.963	4.330	0.0750	0.0415	0.1424
36STYR	R3	1544	14.324	8.944	4.599	0.0520	0.0635	0.1457
37STYR	B	1545	13.939	9.294	4.698	0.0117	0.0364	0.1016
37STYR	R1	1546	13.817	9.410	4.507	0.0543	0.0330	0.0753
37STYR	R2	1547	13.623	9.580	4.426	0.0390	-0.0024	0.0370
37STYR	R3	1548	13.883	9.649	4.398	0.0323	0.0340	0.0642
38STYR	B	1549	14.098	9.230	4.883	-0.0108	0.0529	0.1092
38STYR	R1	1550	14.214	9.365	4.689	0.0188	0.0521	0.1183
38STYR	R2	1551	14.438	9.342	4.540	0.0336	0.0690	0.1381
38STYR	R3	1552	14.382	9.575	4.665	0.0057	0.0644	0.1341
39STYR	B	1553	14.086	9.010	4.979	-0.0080	0.0596	0.1289
39STYR	R1	1554	14.321	9.140	4.995	0.0001	0.0718	0.1637
39STYR	R2	1555	14.513	9.233	5.161	-0.0170	0.0894	0.1737
39STYR	R3	1556	14.581	9.103	4.934	0.0065	0.0717	0.1908
40STYR	B	1557	14.052	8.785	4.919	0.0322	0.0805	0.1358
40STYR	R1	1558	14.003	8.863	5.170	0.0029	0.0604	0.1315
40STYR	R2	1559	13.806	8.863	5.354	-0.0329	0.0618	0.0934
40STYR	R3	1560	14.063	8.835	5.431	-0.0460	0.0842	0.1454
41STYR	B	1561	14.169	8.583	5.026	0.0109	0.0628	0.1312
41STYR	R1	1562	14.122	8.577	4.751	0.0629	0.0563	0.1580
41STYR	R2	1563	14.032	8.401	4.567	0.0822	0.0433	0.1611
41STYR	R3	1564	14.251	8.551	4.515	0.0697	0.0617	0.1611
42STYR	B	1565	14.366	8.492	4.933	0.0215	0.0771	0.1857
42STYR	R1	1566	14.305	8.359	5.143	0.0363	0.0850	0.1846
42STYR	R2	1567	14.411	8.249	5.366	-0.0156	0.0835	0.2087
42STYR	R3	1568	14.287	8.092	5.185	0.0299	0.0842	0.1767
43STYR	B	1569	14.496	8.327	4.827	0.0745	0.0852	0.2138
43STYR	R1	1570	14.590	8.580	4.830	0.0483	0.0912	0.2130
43STYR	R2	1571	14.810	8.720	4.900	0.0372	0.1021	0.2261
43STYR	R3	1572	14.697	8.758	4.657	0.0707	0.0702	0.2053
44STYR	B	1573	14.480	8.078	4.787	0.0760	0.0689	0.2254
44STYR	R1	1574	14.682	8.201	4.952	0.0460	0.1117	0.2288
44STYR	R2	1575	14.800	8.200	5.195	0.0182	0.1101	0.2423
44STYR	R3	1576	14.948	8.241	4.973	0.0424	0.1200	0.2603
45STYR	B	1577	14.252	7.976	4.737	0.0984	0.0689	0.2071
45STYR	R1	1578	14.415	7.822	4.881	0.0757	0.0859	0.2120
45STYR	R2	1579	14.418	7.682	5.112	0.0571	0.0839	0.2110
45STYR	R3	1580	14.497	7.565	4.882	0.0955	0.0923	0.2198
46STYR	B	1581	14.055	7.971	4.605	0.0987	0.0372	0.1673
46STYR	R1	1582	14.286	8.026	4.484	0.1328	0.0492	0.1963
46STYR	R2	1583	14.410	8.207	4.327	0.1422	0.0407	0.1939
46STYR	R3	1584	14.421	7.945	4.265	0.1416	0.0378	0.2059
47STYR	B	1585	13.816	7.967	4.620	0.1188	0.0406	0.1479
47STYR	R1	1586	13.968	7.820	4.801	0.1014	0.0483	0.1562

47STYR	R2	1587	13.991	7.781	5.067	0.0580	0.0631	0.1622
47STYR	R3	1588	14.007	7.571	4.898	0.1005	0.0556	0.1753
48STYR	B	1589	13.615	7.986	4.492	0.1002	0.0186	0.1245
48STYR	R1	1590	13.847	8.014	4.361	0.1182	0.0067	0.1386
48STYR	R2	1591	13.978	8.173	4.186	0.1420	0.0139	0.1629
48STYR	R3	1592	13.971	7.906	4.147	0.1664	0.0132	0.1629
49STYR	B	1593	13.532	7.861	4.301	0.1400	0.0040	0.0961
49STYR	R1	1594	13.674	7.716	4.469	0.1206	0.0159	0.1323
49STYR	R2	1595	13.664	7.482	4.604	0.1292	0.0203	0.1407
49STYR	R3	1596	13.886	7.548	4.464	0.1426	0.0422	0.1722
50STYR	B	1597	13.554	7.664	4.173	0.1847	0.0005	0.1379
50STYR	R1	1598	13.499	7.885	4.038	0.1858	-0.0089	0.1153
50STYR	R2	1599	13.592	8.017	3.821	0.2007	-0.0162	0.1172
50STYR	R3	1600	13.326	8.001	3.866	0.1971	-0.0239	0.0937
1STYR	B	1601	12.260	9.719	3.300	0.1431	-0.1104	-0.1084
1STYR	R1	1602	12.301	9.969	3.274	0.1467	-0.1270	-0.1198
1STYR	R2	1603	12.210	10.147	3.093	0.1634	-0.1356	-0.1366
1STYR	R3	1604	12.178	10.194	3.357	0.1218	-0.1323	-0.1422
2STYR	B	1605	12.408	9.683	3.478	0.1244	-0.1083	-0.0915
2STYR	R1	1606	12.340	9.482	3.295	0.1664	-0.1107	-0.0995
2STYR	R2	1607	12.454	9.289	3.145	0.1985	-0.1128	-0.0728
2STYR	R3	1608	12.200	9.260	3.233	0.1866	-0.1207	-0.1093
3STYR	B	1609	12.577	9.581	3.625	0.1067	-0.0871	-0.0815
3STYR	R1	1610	12.443	9.775	3.743	0.1004	-0.0959	-0.0950
3STYR	R2	1611	12.309	9.833	3.970	0.0705	-0.1031	-0.1107
3STYR	R3	1612	12.512	9.991	3.888	0.0698	-0.0905	-0.0883
4STYR	B	1613	12.803	9.495	3.643	0.1335	-0.0701	-0.0521
4STYR	R1	1614	12.741	9.619	3.421	0.1485	-0.0845	-0.0477
4STYR	R2	1615	12.768	9.583	3.155	0.1717	-0.1177	-0.0409
4STYR	R3	1616	12.841	9.817	3.267	0.1747	-0.1087	-0.0619
5STYR	B	1617	12.944	9.369	3.770	0.1150	-0.0615	-0.0160
5STYR	R1	1618	12.772	9.507	3.897	0.1135	-0.0691	-0.0586
5STYR	R2	1619	12.780	9.665	4.116	0.0709	-0.0515	-0.0694
5STYR	R3	1620	12.571	9.499	4.077	0.0858	-0.0658	-0.0892
6STYR	B	1621	13.097	9.183	3.761	0.1149	-0.0434	-0.0083
6STYR	R1	1622	13.138	9.415	3.608	0.1397	-0.0475	-0.0028
6STYR	R2	1623	13.341	9.586	3.556	0.1390	-0.0442	0.0051
6STYR	R3	1624	13.169	9.517	3.360	0.1519	-0.0524	-0.0033
7STYR	B	1625	13.042	8.958	3.742	0.1634	-0.0658	0.0122
7STYR	R1	1626	13.243	8.973	3.889	0.1216	-0.0451	0.0345
7STYR	R2	1627	13.350	8.876	4.118	0.1115	-0.0178	0.0507
7STYR	R3	1628	13.472	8.829	3.882	0.1506	-0.0006	0.0674
8STYR	B	1629	13.023	8.761	3.837	0.1627	-0.0691	0.0241
8STYR	R1	1630	13.123	8.775	3.592	0.1685	-0.0713	0.0294
8STYR	R2	1631	13.098	8.675	3.343	0.2182	-0.0701	0.0238
8STYR	R3	1632	13.342	8.737	3.439	0.1968	-0.0480	0.0639
9STYR	B	1633	12.962	8.539	3.842	0.1682	-0.0543	0.0123
9STYR	R1	1634	12.959	8.673	4.083	0.1324	-0.0469	0.0170
9STYR	R2	1635	13.037	8.673	4.341	0.0857	-0.0238	0.0314
9STYR	R3	1636	12.798	8.777	4.273	0.1012	-0.0227	-0.0224
10STYR	B	1637	12.961	8.317	3.864	0.1813	-0.0473	0.0206
10STYR	R1	1638	13.177	8.399	3.758	0.1806	-0.0622	0.0646
10STYR	R2	1639	13.328	8.321	3.548	0.1998	-0.0515	0.0744
10STYR	R3	1640	13.446	8.423	3.768	0.1764	-0.0174	0.0712
11STYR	B	1641	12.828	8.123	3.868	0.2070	-0.0530	0.0226
11STYR	R1	1642	12.978	8.183	4.083	0.1430	-0.0356	0.0416
11STYR	R2	1643	12.964	8.232	4.348	0.1051	-0.0448	0.0413
11STYR	R3	1644	13.166	8.083	4.249	0.1311	-0.0236	0.0624
12STYR	B	1645	12.848	7.910	3.947	0.1882	-0.0571	0.0490
12STYR	R1	1646	12.899	7.947	3.695	0.2210	-0.0761	0.0383
12STYR	R2	1647	13.088	7.893	3.510	0.2443	-0.0607	0.0576
12STYR	R3	1648	12.828	7.848	3.454	0.2532	-0.0771	0.0291
13STYR	B	1649	12.950	7.718	4.033	0.1881	-0.0282	0.0375
13STYR	R1	1650	12.694	7.708	3.931	0.2032	-0.0870	0.0225
13STYR	R2	1651	12.541	7.608	3.732	0.2225	-0.0749	0.0015
13STYR	R3	1652	12.446	7.615	3.984	0.2000	-0.0952	-0.0064
14STYR	B	1653	13.112	7.632	4.193	0.1826	-0.0187	0.0791
14STYR	R1	1654	13.147	7.585	3.924	0.2149	-0.0476	0.0843
14STYR	R2	1655	13.176	7.382	3.748	0.2418	-0.0571	0.0997
14STYR	R3	1656	13.339	7.597	3.733	0.2467	-0.0597	0.1154
15STYR	B	1657	13.165	7.655	4.447	0.1531	-0.0207	0.0772
15STYR	R1	1658	12.901	7.713	4.350	0.1526	-0.0336	0.0576
15STYR	R2	1659	12.645	7.633	4.382	0.1512	-0.0448	0.0179

15STYR	R3	1660	12.703	7.896	4.373	0.1396	-0.0423	0.0151
16STYR	B	1661	13.078	7.826	4.586	0.1148	-0.0442	0.0604
16STYR	R1	1662	13.271	7.714	4.706	0.1004	-0.0007	0.0869
16STYR	R2	1663	13.484	7.780	4.859	0.0763	0.0305	0.1073
16STYR	R3	1664	13.301	7.604	4.951	0.0880	0.0113	0.0938
17STYR	B	1665	13.056	7.970	4.766	0.0989	-0.0166	0.0479
17STYR	R1	1666	12.904	7.754	4.772	0.1063	-0.0160	0.0272
17STYR	R2	1667	12.651	7.670	4.814	0.1068	-0.0229	0.0162
17STYR	R3	1668	12.858	7.530	4.916	0.1053	-0.0048	0.0444
18STYR	B	1669	12.921	7.999	4.956	0.0472	-0.0215	0.0433
18STYR	R1	1670	13.168	8.055	4.997	0.0565	0.0180	0.0547
18STYR	R2	1671	13.337	8.005	5.203	0.0197	0.0230	0.0862
18STYR	R3	1672	13.333	8.249	5.086	0.0365	0.0219	0.0835
19STYR	B	1673	12.886	8.146	5.144	0.0365	-0.0139	0.0160
19STYR	R1	1674	12.821	7.889	5.178	0.0245	0.0021	0.0437
19STYR	R2	1675	12.864	7.689	5.355	0.0318	0.0114	0.0525
19STYR	R3	1676	12.618	7.725	5.249	0.0356	-0.0158	0.0344
20STYR	B	1677	12.890	8.390	5.127	0.0259	-0.0181	0.0271
20STYR	R1	1678	12.974	8.284	5.353	0.0025	0.0160	0.0340
20STYR	R2	1679	13.187	8.283	5.518	-0.0308	0.0339	0.0775
20STYR	R3	1680	12.938	8.301	5.620	-0.0528	0.0213	0.0264
21STYR	B	1681	12.761	8.520	4.975	0.0466	-0.0172	0.0080
21STYR	R1	1682	13.001	8.476	4.894	0.0303	0.0035	0.0364
21STYR	R2	1683	13.188	8.642	4.793	0.0469	-0.0075	0.0491
21STYR	R3	1684	13.155	8.396	4.687	0.0601	-0.0167	0.0662
22STYR	B	1685	12.537	8.585	5.002	0.0056	-0.0411	-0.0348
22STYR	R1	1686	12.756	8.732	5.112	-0.0032	-0.0111	-0.0243
22STYR	R2	1687	12.911	8.805	5.320	-0.0317	0.0009	-0.0073
22STYR	R3	1688	12.876	8.974	5.113	-0.0142	-0.0057	-0.0157
23STYR	B	1689	12.335	8.539	4.876	0.0380	-0.0349	-0.0604
23STYR	R1	1690	12.557	8.450	4.767	0.0317	-0.0331	-0.0321
23STYR	R2	1691	12.663	8.228	4.654	0.0811	-0.0287	0.0051
23STYR	R3	1692	12.681	8.466	4.528	0.0875	-0.0339	-0.0036
24STYR	B	1693	12.154	8.411	4.836	0.0560	-0.0619	-0.0854
24STYR	R1	1694	12.349	8.306	4.986	0.0402	-0.0487	-0.0502
24STYR	R2	1695	12.480	8.070	4.989	0.0641	-0.0352	-0.0202
24STYR	R3	1696	12.422	8.195	5.221	0.0201	-0.0289	-0.0345
25STYR	B	1697	12.047	8.192	4.868	0.0600	-0.0614	-0.0927
25STYR	R1	1698	12.133	8.276	4.615	0.0875	-0.0448	-0.0730
25STYR	R2	1699	12.045	8.288	4.360	0.1142	-0.0908	-0.0847
25STYR	R3	1700	12.306	8.240	4.410	0.1148	-0.0517	-0.0489
26STYR	B	1701	11.953	8.164	5.116	0.0411	-0.0442	-0.0836
26STYR	R1	1702	12.053	7.938	4.984	0.0707	-0.0494	-0.0812
26STYR	R2	1703	11.983	7.683	4.929	0.0942	-0.0621	-0.0523
26STYR	R3	1704	12.224	7.736	5.038	0.0689	-0.0323	-0.0106
27STYR	B	1705	11.956	8.381	5.249	0.0072	-0.0419	-0.0852
27STYR	R1	1706	11.726	8.306	5.109	0.0108	-0.0837	-0.1208
27STYR	R2	1707	11.547	8.418	4.941	0.0239	-0.0853	-0.1359
27STYR	R3	1708	11.463	8.253	5.137	0.0077	-0.0768	-0.1357
28STYR	B	1709	12.145	8.511	5.338	0.0005	-0.0423	-0.0695
28STYR	R1	1710	12.061	8.277	5.460	0.0029	-0.0446	-0.0877
28STYR	R2	1711	11.983	8.175	5.697	-0.0091	-0.0297	-0.0853
28STYR	R3	1712	12.150	8.034	5.538	0.0040	-0.0369	-0.0652
29STYR	B	1713	12.239	8.729	5.307	-0.0134	-0.0236	-0.0757
29STYR	R1	1714	12.000	8.687	5.201	0.0150	-0.0450	-0.1135
29STYR	R2	1715	11.755	8.800	5.202	0.0026	-0.0718	-0.1320
29STYR	R3	1716	11.866	8.721	4.969	0.0252	-0.0675	-0.1227
30STYR	B	1717	12.283	8.892	5.139	-0.0076	-0.0232	-0.0627
30STYR	R1	1718	12.391	8.948	5.367	-0.0318	-0.0111	-0.0662
30STYR	R2	1719	12.617	9.011	5.501	-0.0469	0.0068	-0.0492
30STYR	R3	1720	12.399	9.170	5.521	-0.0570	-0.0038	-0.0755
31STYR	B	1721	12.414	9.061	5.052	-0.0137	-0.0329	-0.0723
31STYR	R1	1722	12.134	9.076	5.071	-0.0093	-0.0314	-0.1118
31STYR	R2	1723	11.900	9.129	4.949	-0.0086	-0.0647	-0.1276
31STYR	R3	1724	11.932	9.189	5.210	-0.0297	-0.0466	-0.1291
32STYR	B	1725	12.475	9.235	4.888	0.0047	-0.0292	-0.0719
32STYR	R1	1726	12.545	9.000	4.817	0.0279	-0.0322	-0.0500
32STYR	R2	1727	12.513	8.850	4.594	0.0550	-0.0347	-0.0522
32STYR	R3	1728	12.759	8.885	4.700	0.0441	-0.0213	-0.0313
33STYR	B	1729	12.310	9.317	4.726	0.0144	-0.0456	-0.1076
33STYR	R1	1730	12.539	9.314	4.627	0.0255	-0.0373	-0.0644
33STYR	R2	1731	12.656	9.214	4.405	0.0567	-0.0448	-0.0445
33STYR	R3	1732	12.706	9.468	4.482	0.0325	-0.0367	-0.0556

34STYR	B	1733	12.154	9.195	4.606	0.0464	-0.0563	-0.1005
34STYR	R1	1734	12.085	9.431	4.714	-0.0146	-0.0902	-0.1305
34STYR	R2	1735	11.950	9.660	4.668	0.0238	-0.0708	-0.1483
34STYR	R3	1736	11.965	9.560	4.919	-0.0124	-0.0662	-0.1442
35STYR	B	1737	11.962	9.106	4.511	0.0496	-0.0859	-0.1317
35STYR	R1	1738	12.191	9.118	4.368	0.0674	-0.0837	-0.0972
35STYR	R2	1739	12.295	9.203	4.134	0.0900	-0.0798	-0.0858
35STYR	R3	1740	12.342	8.951	4.219	0.1079	-0.0713	-0.0703
36STYR	B	1741	11.747	9.009	4.552	0.0400	-0.0866	-0.1358
36STYR	R1	1742	11.954	8.867	4.443	0.0692	-0.0809	-0.1155
36STYR	R2	1743	12.144	8.677	4.470	0.0730	-0.0727	-0.0826
36STYR	R3	1744	12.003	8.697	4.240	0.0996	-0.0901	-0.1005
37STYR	B	1745	11.551	8.974	4.675	0.0472	-0.0831	-0.1555
37STYR	R1	1746	11.663	8.766	4.568	0.0579	-0.1052	-0.1418
37STYR	R2	1747	11.632	8.565	4.389	0.1077	-0.1142	-0.1405
37STYR	R3	1748	11.776	8.525	4.614	0.0773	-0.0914	-0.1169
38STYR	B	1749	11.443	8.870	4.872	0.0373	-0.1016	-0.1689
38STYR	R1	1750	11.291	9.033	4.729	0.0278	-0.1128	-0.2049
38STYR	R2	1751	11.137	9.254	4.752	0.0097	-0.1224	-0.2336
38STYR	R3	1752	11.044	9.039	4.619	0.0368	-0.1386	-0.2265
39STYR	B	1753	11.369	9.030	5.059	-0.0034	-0.0788	-0.1730
39STYR	R1	1754	11.331	8.769	5.107	0.0170	-0.0851	-0.1832
39STYR	R2	1755	11.380	8.617	5.325	-0.0070	-0.0624	-0.1619
39STYR	R3	1756	11.153	8.581	5.183	0.0094	-0.0784	-0.1843
40STYR	B	1757	11.498	9.243	5.100	-0.0139	-0.0597	-0.2071
40STYR	R1	1758	11.247	9.256	5.169	-0.0282	-0.0872	-0.2238
40STYR	R2	1759	11.089	9.324	5.376	-0.0614	-0.1025	-0.2440
40STYR	R3	1760	11.046	9.433	5.133	-0.0286	-0.0921	-0.2452
41STYR	B	1761	11.533	9.413	4.928	-0.0106	-0.0895	-0.1924
41STYR	R1	1762	11.611	9.478	5.181	-0.0574	-0.0722	-0.1778
41STYR	R2	1763	11.566	9.678	5.356	-0.0824	-0.0635	-0.1941
41STYR	R3	1764	11.814	9.576	5.329	-0.0765	-0.0582	-0.1608
42STYR	B	1765	11.570	9.441	4.684	0.0110	-0.1151	-0.1799
42STYR	R1	1766	11.373	9.601	4.798	-0.0209	-0.1109	-0.2141
42STYR	R2	1767	11.115	9.668	4.754	-0.0177	-0.1170	-0.2431
42STYR	R3	1768	11.287	9.850	4.855	-0.0284	-0.1066	-0.2435
43STYR	B	1769	11.519	9.630	4.544	0.0113	-0.0947	-0.2002
43STYR	R1	1770	11.661	9.454	4.421	0.0338	-0.1050	-0.1842
43STYR	R2	1771	11.881	9.436	4.265	0.0641	-0.0972	-0.1425
43STYR	R3	1772	11.654	9.309	4.193	0.0837	-0.1094	-0.1831
44STYR	B	1773	11.497	9.721	4.331	0.0312	-0.1241	-0.2137
44STYR	R1	1774	11.585	9.887	4.516	0.0005	-0.0951	-0.2191
44STYR	R2	1775	11.521	10.130	4.615	-0.0125	-0.0978	-0.2208
44STYR	R3	1776	11.780	10.066	4.573	-0.0080	-0.0900	-0.2052
45STYR	B	1777	11.503	9.910	4.198	0.0314	-0.1315	-0.2134
45STYR	R1	1778	11.277	9.872	4.311	0.0212	-0.1218	-0.2604
45STYR	R2	1779	11.014	9.877	4.251	0.0245	-0.1357	-0.2761
45STYR	R3	1780	11.105	10.008	4.468	0.0030	-0.1397	-0.2646
46STYR	B	1781	11.473	9.993	3.985	0.0596	-0.1531	-0.2273
46STYR	R1	1782	11.541	10.171	4.163	0.0312	-0.1214	-0.2160
46STYR	R2	1783	11.460	10.404	4.272	0.0043	-0.1190	-0.2413
46STYR	R3	1784	11.719	10.370	4.204	0.0167	-0.1122	-0.1975
47STYR	B	1785	11.554	10.090	3.789	0.0708	-0.1409	-0.2349
47STYR	R1	1786	11.286	10.034	3.825	0.0822	-0.1386	-0.2565
47STYR	R2	1787	11.071	9.921	3.707	0.0968	-0.1624	-0.2606
47STYR	R3	1788	11.039	10.136	3.867	0.0719	-0.1566	-0.2732
48STYR	B	1789	11.709	10.257	3.724	0.0761	-0.1251	-0.2014
48STYR	R1	1790	11.770	10.033	3.624	0.1113	-0.1171	-0.1963
48STYR	R2	1791	11.838	9.913	3.392	0.1343	-0.1305	-0.1826
48STYR	R3	1792	11.993	9.882	3.610	0.1059	-0.1282	-0.1620
49STYR	B	1793	11.747	10.448	3.593	0.0802	-0.1336	-0.2101
49STYR	R1	1794	11.501	10.411	3.707	0.0663	-0.1381	-0.2475
49STYR	R2	1795	11.317	10.512	3.877	0.0384	-0.1531	-0.2688
49STYR	R3	1796	11.244	10.432	3.630	0.0716	-0.1691	-0.2735
50STYR	B	1797	11.929	10.574	3.495	0.0754	-0.1323	-0.1977
50STYR	R1	1798	11.986	10.415	3.711	0.0596	-0.1316	-0.1785
50STYR	R2	1799	12.099	10.426	3.956	0.0206	-0.1092	-0.1614
50STYR	R3	1800	12.176	10.236	3.781	0.0736	-0.1036	-0.1443

18.00000 18.00000 18.00000

1.2 Polystyrene NP System Topology

```
#include "martini_v2.0_PEO_PS_CNP.itp"
#include "PS50.itp"

[ system ]
; name
 POLYSTYRENE

[ molecules ]
; name                 number
PS50                    9
```

1.3 Polystyrene NP Topology

```
[ moleculetype ]
; molname  nrexcl
PS50        3

[ atoms ]
;  nr    type        resnr  residu    atom    cgnr        charge
    1    SCY             1  STYR      B          1     0.00000E+00
    2    STY             1  STYR      R1         2     0.00000E+00
    3    STY             1  STYR      R2         3     0.00000E+00
    4    STY             1  STYR      R3         4     0.00000E+00
    5    SCY             2  STYR      B          5     0.00000E+00
    6    STY             2  STYR      R1         6     0.00000E+00
    7    STY             2  STYR      R2         7     0.00000E+00
    8    STY             2  STYR      R3         8     0.00000E+00
    9    SCY             3  STYR      B          9     0.00000E+00
   10    STY             3  STYR      R1        10     0.00000E+00
   11    STY             3  STYR      R2        11     0.00000E+00
   12    STY             3  STYR      R3        12     0.00000E+00
   13    SCY             4  STYR      B         13     0.00000E+00
   14    STY             4  STYR      R1        14     0.00000E+00
   15    STY             4  STYR      R2        15     0.00000E+00
   16    STY             4  STYR      R3        16     0.00000E+00
   17    SCY             5  STYR      B         17     0.00000E+00
   18    STY             5  STYR      R1        18     0.00000E+00
   19    STY             5  STYR      R2        19     0.00000E+00
   20    STY             5  STYR      R3        20     0.00000E+00
   21    SCY             6  STYR      B         21     0.00000E+00
   22    STY             6  STYR      R1        22     0.00000E+00
   23    STY             6  STYR      R2        23     0.00000E+00
   24    STY             6  STYR      R3        24     0.00000E+00
   25    SCY             7  STYR      B         25     0.00000E+00
   26    STY             7  STYR      R1        26     0.00000E+00
   27    STY             7  STYR      R2        27     0.00000E+00
   28    STY             7  STYR      R3        28     0.00000E+00
   29    SCY             8  STYR      B         29     0.00000E+00
   30    STY             8  STYR      R1        30     0.00000E+00
   31    STY             8  STYR      R2        31     0.00000E+00
   32    STY             8  STYR      R3        32     0.00000E+00
   33    SCY             9  STYR      B         33     0.00000E+00
   34    STY             9  STYR      R1        34     0.00000E+00
   35    STY             9  STYR      R2        35     0.00000E+00
   36    STY             9  STYR      R3        36     0.00000E+00
   37    SCY            10  STYR      B         37     0.00000E+00
   38    STY            10  STYR      R1        38     0.00000E+00
   39    STY            10  STYR      R2        39     0.00000E+00
   40    STY            10  STYR      R3        40     0.00000E+00
   41    SCY            11  STYR      B         41     0.00000E+00
   42    STY            11  STYR      R1        42     0.00000E+00
   43    STY            11  STYR      R2        43     0.00000E+00
   44    STY            11  STYR      R3        44     0.00000E+00
   45    SCY            12  STYR      B         45     0.00000E+00
   46    STY            12  STYR      R1        46     0.00000E+00
   47    STY            12  STYR      R2        47     0.00000E+00
   48    STY            12  STYR      R3        48     0.00000E+00
   49    SCY            13  STYR      B         49     0.00000E+00
```

50	STY	13	STYR	R1	50	0.00000E+00
51	STY	13	STYR	R2	51	0.00000E+00
52	STY	13	STYR	R3	52	0.00000E+00
53	SCY	14	STYR	B	53	0.00000E+00
54	STY	14	STYR	R1	54	0.00000E+00
55	STY	14	STYR	R2	55	0.00000E+00
56	STY	14	STYR	R3	56	0.00000E+00
57	SCY	15	STYR	B	57	0.00000E+00
58	STY	15	STYR	R1	58	0.00000E+00
59	STY	15	STYR	R2	59	0.00000E+00
60	STY	15	STYR	R3	60	0.00000E+00
61	SCY	16	STYR	B	61	0.00000E+00
62	STY	16	STYR	R1	62	0.00000E+00
63	STY	16	STYR	R2	63	0.00000E+00
64	STY	16	STYR	R3	64	0.00000E+00
65	SCY	17	STYR	B	65	0.00000E+00
66	STY	17	STYR	R1	66	0.00000E+00
67	STY	17	STYR	R2	67	0.00000E+00
68	STY	17	STYR	R3	68	0.00000E+00
69	SCY	18	STYR	B	69	0.00000E+00
70	STY	18	STYR	R1	70	0.00000E+00
71	STY	18	STYR	R2	71	0.00000E+00
72	STY	18	STYR	R3	72	0.00000E+00
73	SCY	19	STYR	B	73	0.00000E+00
74	STY	19	STYR	R1	74	0.00000E+00
75	STY	19	STYR	R2	75	0.00000E+00
76	STY	19	STYR	R3	76	0.00000E+00
77	SCY	20	STYR	B	77	0.00000E+00
78	STY	20	STYR	R1	78	0.00000E+00
79	STY	20	STYR	R2	79	0.00000E+00
80	STY	20	STYR	R3	80	0.00000E+00
81	SCY	21	STYR	B	81	0.00000E+00
82	STY	21	STYR	R1	82	0.00000E+00
83	STY	21	STYR	R2	83	0.00000E+00
84	STY	21	STYR	R3	84	0.00000E+00
85	SCY	22	STYR	B	85	0.00000E+00
86	STY	22	STYR	R1	86	0.00000E+00
87	STY	22	STYR	R2	87	0.00000E+00
88	STY	22	STYR	R3	88	0.00000E+00
89	SCY	23	STYR	B	89	0.00000E+00
90	STY	23	STYR	R1	90	0.00000E+00
91	STY	23	STYR	R2	91	0.00000E+00
92	STY	23	STYR	R3	92	0.00000E+00
93	SCY	24	STYR	B	93	0.00000E+00
94	STY	24	STYR	R1	94	0.00000E+00
95	STY	24	STYR	R2	95	0.00000E+00
96	STY	24	STYR	R3	96	0.00000E+00
97	SCY	25	STYR	B	97	0.00000E+00
98	STY	25	STYR	R1	98	0.00000E+00
99	STY	25	STYR	R2	99	0.00000E+00
100	STY	25	STYR	R3	100	0.00000E+00
101	SCY	26	STYR	B	101	0.00000E+00
102	STY	26	STYR	R1	102	0.00000E+00
103	STY	26	STYR	R2	103	0.00000E+00
104	STY	26	STYR	R3	104	0.00000E+00
105	SCY	27	STYR	B	105	0.00000E+00
106	STY	27	STYR	R1	106	0.00000E+00
107	STY	27	STYR	R2	107	0.00000E+00
108	STY	27	STYR	R3	108	0.00000E+00
109	SCY	28	STYR	B	109	0.00000E+00
110	STY	28	STYR	R1	110	0.00000E+00
111	STY	28	STYR	R2	111	0.00000E+00
112	STY	28	STYR	R3	112	0.00000E+00
113	SCY	29	STYR	B	113	0.00000E+00
114	STY	29	STYR	R1	114	0.00000E+00
115	STY	29	STYR	R2	115	0.00000E+00
116	STY	29	STYR	R3	116	0.00000E+00
117	SCY	30	STYR	B	117	0.00000E+00
118	STY	30	STYR	R1	118	0.00000E+00
119	STY	30	STYR	R2	119	0.00000E+00
120	STY	30	STYR	R3	120	0.00000E+00
121	SCY	31	STYR	B	121	0.00000E+00
122	STY	31	STYR	R1	122	0.00000E+00

123	STY	31	STYR	R2	123	0.00000E+00
124	STY	31	STYR	R3	124	0.00000E+00
125	SCY	32	STYR	B	125	0.00000E+00
126	STY	32	STYR	R1	126	0.00000E+00
127	STY	32	STYR	R2	127	0.00000E+00
128	STY	32	STYR	R3	128	0.00000E+00
129	SCY	33	STYR	B	129	0.00000E+00
130	STY	33	STYR	R1	130	0.00000E+00
131	STY	33	STYR	R2	131	0.00000E+00
132	STY	33	STYR	R3	132	0.00000E+00
133	SCY	34	STYR	B	133	0.00000E+00
134	STY	34	STYR	R1	134	0.00000E+00
135	STY	34	STYR	R2	135	0.00000E+00
136	STY	34	STYR	R3	136	0.00000E+00
137	SCY	35	STYR	B	137	0.00000E+00
138	STY	35	STYR	R1	138	0.00000E+00
139	STY	35	STYR	R2	139	0.00000E+00
140	STY	35	STYR	R3	140	0.00000E+00
141	SCY	36	STYR	B	141	0.00000E+00
142	STY	36	STYR	R1	142	0.00000E+00
143	STY	36	STYR	R2	143	0.00000E+00
144	STY	36	STYR	R3	144	0.00000E+00
145	SCY	37	STYR	B	145	0.00000E+00
146	STY	37	STYR	R1	146	0.00000E+00
147	STY	37	STYR	R2	147	0.00000E+00
148	STY	37	STYR	R3	148	0.00000E+00
149	SCY	38	STYR	B	149	0.00000E+00
150	STY	38	STYR	R1	150	0.00000E+00
151	STY	38	STYR	R2	151	0.00000E+00
152	STY	38	STYR	R3	152	0.00000E+00
153	SCY	39	STYR	B	153	0.00000E+00
154	STY	39	STYR	R1	154	0.00000E+00
155	STY	39	STYR	R2	155	0.00000E+00
156	STY	39	STYR	R3	156	0.00000E+00
157	SCY	40	STYR	B	157	0.00000E+00
158	STY	40	STYR	R1	158	0.00000E+00
159	STY	40	STYR	R2	159	0.00000E+00
160	STY	40	STYR	R3	160	0.00000E+00
161	SCY	41	STYR	B	161	0.00000E+00
162	STY	41	STYR	R1	162	0.00000E+00
163	STY	41	STYR	R2	163	0.00000E+00
164	STY	41	STYR	R3	164	0.00000E+00
165	SCY	42	STYR	B	165	0.00000E+00
166	STY	42	STYR	R1	166	0.00000E+00
167	STY	42	STYR	R2	167	0.00000E+00
168	STY	42	STYR	R3	168	0.00000E+00
169	SCY	43	STYR	B	169	0.00000E+00
170	STY	43	STYR	R1	170	0.00000E+00
171	STY	43	STYR	R2	171	0.00000E+00
172	STY	43	STYR	R3	172	0.00000E+00
173	SCY	44	STYR	B	173	0.00000E+00
174	STY	44	STYR	R1	174	0.00000E+00
175	STY	44	STYR	R2	175	0.00000E+00
176	STY	44	STYR	R3	176	0.00000E+00
177	SCY	45	STYR	B	177	0.00000E+00
178	STY	45	STYR	R1	178	0.00000E+00
179	STY	45	STYR	R2	179	0.00000E+00
180	STY	45	STYR	R3	180	0.00000E+00
181	SCY	46	STYR	B	181	0.00000E+00
182	STY	46	STYR	R1	182	0.00000E+00
183	STY	46	STYR	R2	183	0.00000E+00
184	STY	46	STYR	R3	184	0.00000E+00
185	SCY	47	STYR	B	185	0.00000E+00
186	STY	47	STYR	R1	186	0.00000E+00
187	STY	47	STYR	R2	187	0.00000E+00
188	STY	47	STYR	R3	188	0.00000E+00
189	SCY	48	STYR	B	189	0.00000E+00
190	STY	48	STYR	R1	190	0.00000E+00
191	STY	48	STYR	R2	191	0.00000E+00
192	STY	48	STYR	R3	192	0.00000E+00
193	SCY	49	STYR	B	193	0.00000E+00
194	STY	49	STYR	R1	194	0.00000E+00
195	STY	49	STYR	R2	195	0.00000E+00

```
 196    STY           49  STYR       R3     196     0.00000E+00
 197    SCY           50  STYR       B      197     0.00000E+00
 198    STY           50  STYR       R1     198     0.00000E+00
 199    STY           50  STYR       R2     199     0.00000E+00
 200    STY           50  STYR       R3     200     0.00000E+00


[ bonds ]
;  ai  aj   funct
    1     2    1     0.270000 8000.000000
    1     6    1     0.270000 8000.000000
    5     6    1     0.270000 8000.000000
    5    10    1     0.270000 8000.000000
    9    10    1     0.270000 8000.000000
    9    14    1     0.270000 8000.000000
   13    14    1     0.270000 8000.000000
   13    18    1     0.270000 8000.000000
   17    18    1     0.270000 8000.000000
   17    22    1     0.270000 8000.000000
   21    22    1     0.270000 8000.000000
   21    26    1     0.270000 8000.000000
   25    26    1     0.270000 8000.000000
   25    30    1     0.270000 8000.000000
   29    30    1     0.270000 8000.000000
   29    34    1     0.270000 8000.000000
   33    34    1     0.270000 8000.000000
   33    38    1     0.270000 8000.000000
   37    38    1     0.270000 8000.000000
   37    42    1     0.270000 8000.000000
   41    42    1     0.270000 8000.000000
   41    46    1     0.270000 8000.000000
   45    46    1     0.270000 8000.000000
   45    50    1     0.270000 8000.000000
   49    50    1     0.270000 8000.000000
   49    54    1     0.270000 8000.000000
   53    54    1     0.270000 8000.000000
   53    58    1     0.270000 8000.000000
   57    58    1     0.270000 8000.000000
   57    62    1     0.270000 8000.000000
   61    62    1     0.270000 8000.000000
   61    66    1     0.270000 8000.000000
   65    66    1     0.270000 8000.000000
   65    70    1     0.270000 8000.000000
   69    70    1     0.270000 8000.000000
   69    74    1     0.270000 8000.000000
   73    74    1     0.270000 8000.000000
   73    78    1     0.270000 8000.000000
   77    78    1     0.270000 8000.000000
   77    82    1     0.270000 8000.000000
   81    82    1     0.270000 8000.000000
   81    86    1     0.270000 8000.000000
   85    86    1     0.270000 8000.000000
   85    90    1     0.270000 8000.000000
   89    90    1     0.270000 8000.000000
   89    94    1     0.270000 8000.000000
   93    94    1     0.270000 8000.000000
   93    98    1     0.270000 8000.000000
   97    98    1     0.270000 8000.000000
   97   102    1     0.270000 8000.000000
  101   102    1     0.270000 8000.000000
  101   106    1     0.270000 8000.000000
  105   106    1     0.270000 8000.000000
  105   110    1     0.270000 8000.000000
  109   110    1     0.270000 8000.000000
  109   114    1     0.270000 8000.000000
  113   114    1     0.270000 8000.000000
  113   118    1     0.270000 8000.000000
  117   118    1     0.270000 8000.000000
  117   122    1     0.270000 8000.000000
  121   122    1     0.270000 8000.000000
  121   126    1     0.270000 8000.000000
  125   126    1     0.270000 8000.000000
  125   130    1     0.270000 8000.000000
```

```
 129   130    1     0.270000 8000.000000
 129   134    1     0.270000 8000.000000
 133   134    1     0.270000 8000.000000
 133   138    1     0.270000 8000.000000
 137   138    1     0.270000 8000.000000
 137   142    1     0.270000 8000.000000
 141   142    1     0.270000 8000.000000
 141   146    1     0.270000 8000.000000
 145   146    1     0.270000 8000.000000
 145   150    1     0.270000 8000.000000
 149   150    1     0.270000 8000.000000
 149   154    1     0.270000 8000.000000
 153   154    1     0.270000 8000.000000
 153   158    1     0.270000 8000.000000
 157   158    1     0.270000 8000.000000
 157   162    1     0.270000 8000.000000
 161   162    1     0.270000 8000.000000
 161   166    1     0.270000 8000.000000
 165   166    1     0.270000 8000.000000
 165   170    1     0.270000 8000.000000
 169   170    1     0.270000 8000.000000
 169   174    1     0.270000 8000.000000
 173   174    1     0.270000 8000.000000
 173   178    1     0.270000 8000.000000
 177   178    1     0.270000 8000.000000
 177   182    1     0.270000 8000.000000
 181   182    1     0.270000 8000.000000
 181   186    1     0.270000 8000.000000
 185   186    1     0.270000 8000.000000
 185   190    1     0.270000 8000.000000
 189   190    1     0.270000 8000.000000
 189   194    1     0.270000 8000.000000
 193   194    1     0.270000 8000.000000
 193   198    1     0.270000 8000.000000
 197   198    1     0.270000 8000.000000


[ constraints ]
;  ai  aj  funct length
   2     3    1     0.270000
   3     4    1     0.270000
   4     2    1     0.270000
   6     7    1     0.270000
   7     8    1     0.270000
   8     6    1     0.270000
  10    11    1     0.270000
  11    12    1     0.270000
  12    10    1     0.270000
  14    15    1     0.270000
  15    16    1     0.270000
  16    14    1     0.270000
  18    19    1     0.270000
  19    20    1     0.270000
  20    18    1     0.270000
  22    23    1     0.270000
  23    24    1     0.270000
  24    22    1     0.270000
  26    27    1     0.270000
  27    28    1     0.270000
  28    26    1     0.270000
  30    31    1     0.270000
  31    32    1     0.270000
  32    30    1     0.270000
  34    35    1     0.270000
  35    36    1     0.270000
  36    34    1     0.270000
  38    39    1     0.270000
  39    40    1     0.270000
  40    38    1     0.270000
  42    43    1     0.270000
  43    44    1     0.270000
  44    42    1     0.270000
  46    47    1     0.270000
```

47	48	1	0.270000
48	46	1	0.270000
50	51	1	0.270000
51	52	1	0.270000
52	50	1	0.270000
54	55	1	0.270000
55	56	1	0.270000
56	54	1	0.270000
58	59	1	0.270000
59	60	1	0.270000
60	58	1	0.270000
62	63	1	0.270000
63	64	1	0.270000
64	62	1	0.270000
66	67	1	0.270000
67	68	1	0.270000
68	66	1	0.270000
70	71	1	0.270000
71	72	1	0.270000
72	70	1	0.270000
74	75	1	0.270000
75	76	1	0.270000
76	74	1	0.270000
78	79	1	0.270000
79	80	1	0.270000
80	78	1	0.270000
82	83	1	0.270000
83	84	1	0.270000
84	82	1	0.270000
86	87	1	0.270000
87	88	1	0.270000
88	86	1	0.270000
90	91	1	0.270000
91	92	1	0.270000
92	90	1	0.270000
94	95	1	0.270000
95	96	1	0.270000
96	94	1	0.270000
98	99	1	0.270000
99	100	1	0.270000
100	98	1	0.270000
102	103	1	0.270000
103	104	1	0.270000
104	102	1	0.270000
106	107	1	0.270000
107	108	1	0.270000
108	106	1	0.270000
110	111	1	0.270000
111	112	1	0.270000
112	110	1	0.270000
114	115	1	0.270000
115	116	1	0.270000
116	114	1	0.270000
118	119	1	0.270000
119	120	1	0.270000
120	118	1	0.270000
122	123	1	0.270000
123	124	1	0.270000
124	122	1	0.270000
126	127	1	0.270000
127	128	1	0.270000
128	126	1	0.270000
130	131	1	0.270000
131	132	1	0.270000
132	130	1	0.270000
134	135	1	0.270000
135	136	1	0.270000
136	134	1	0.270000
138	139	1	0.270000
139	140	1	0.270000
140	138	1	0.270000
142	143	1	0.270000
143	144	1	0.270000

```
 144   142    1     0.270000
 146   147    1     0.270000
 147   148    1     0.270000
 148   146    1     0.270000
 150   151    1     0.270000
 151   152    1     0.270000
 152   150    1     0.270000
 154   155    1     0.270000
 155   156    1     0.270000
 156   154    1     0.270000
 158   159    1     0.270000
 159   160    1     0.270000
 160   158    1     0.270000
 162   163    1     0.270000
 163   164    1     0.270000
 164   162    1     0.270000
 166   167    1     0.270000
 167   168    1     0.270000
 168   166    1     0.270000
 170   171    1     0.270000
 171   172    1     0.270000
 172   170    1     0.270000
 174   175    1     0.270000
 175   176    1     0.270000
 176   174    1     0.270000
 178   179    1     0.270000
 179   180    1     0.270000
 180   178    1     0.270000
 182   183    1     0.270000
 183   184    1     0.270000
 184   182    1     0.270000
 186   187    1     0.270000
 187   188    1     0.270000
 188   186    1     0.270000
 190   191    1     0.270000
 191   192    1     0.270000
 192   190    1     0.270000
 194   195    1     0.270000
 195   196    1     0.270000
 196   194    1     0.270000
 198   199    1     0.270000
 199   200    1     0.270000
 200   198    1     0.270000


[ angles ]
;  ai  aj  ak  funct
    1     2     3    1   136.000000  100.000000
    1     2     4    1   136.000000  100.000000
    1     6     7    1   136.000000  100.000000
    1     6     8    1   136.000000  100.000000
    2     1     6    1   120.000000   25.000000
    1     6     5    1    52.000000  550.000000
    5     6     7    1   136.000000  100.000000
    5     6     8    1   136.000000  100.000000
    5    10    11    1   136.000000  100.000000
    5    10    12    1   136.000000  100.000000
    6     5    10    1   120.000000   25.000000
    5    10     9    1    52.000000  550.000000
    9    10    11    1   136.000000  100.000000
    9    10    12    1   136.000000  100.000000
    9    14    15    1   136.000000  100.000000
    9    14    16    1   136.000000  100.000000
   10     9    14    1   120.000000   25.000000
    9    14    13    1    52.000000  550.000000
   13    14    15    1   136.000000  100.000000
   13    14    16    1   136.000000  100.000000
   13    18    19    1   136.000000  100.000000
   13    18    20    1   136.000000  100.000000
   14    13    18    1   120.000000   25.000000
   13    18    17    1    52.000000  550.000000
   17    18    19    1   136.000000  100.000000
   17    18    20    1   136.000000  100.000000
```

17	22	23	1	136.000000	100.000000
17	22	24	1	136.000000	100.000000
18	17	22	1	120.000000	25.000000
17	22	21	1	52.000000	550.000000
21	22	23	1	136.000000	100.000000
21	22	24	1	136.000000	100.000000
21	26	27	1	136.000000	100.000000
21	26	28	1	136.000000	100.000000
22	21	26	1	120.000000	25.000000
21	26	25	1	52.000000	550.000000
25	26	27	1	136.000000	100.000000
25	26	28	1	136.000000	100.000000
25	30	31	1	136.000000	100.000000
25	30	32	1	136.000000	100.000000
26	25	30	1	120.000000	25.000000
25	30	29	1	52.000000	550.000000
29	30	31	1	136.000000	100.000000
29	30	32	1	136.000000	100.000000
29	34	35	1	136.000000	100.000000
29	34	36	1	136.000000	100.000000
30	29	34	1	120.000000	25.000000
29	34	33	1	52.000000	550.000000
33	34	35	1	136.000000	100.000000
33	34	36	1	136.000000	100.000000
33	38	39	1	136.000000	100.000000
33	38	40	1	136.000000	100.000000
34	33	38	1	120.000000	25.000000
33	38	37	1	52.000000	550.000000
37	38	39	1	136.000000	100.000000
37	38	40	1	136.000000	100.000000
37	42	43	1	136.000000	100.000000
37	42	44	1	136.000000	100.000000
38	37	42	1	120.000000	25.000000
37	42	41	1	52.000000	550.000000
41	42	43	1	136.000000	100.000000
41	42	44	1	136.000000	100.000000
41	46	47	1	136.000000	100.000000
41	46	48	1	136.000000	100.000000
42	41	46	1	120.000000	25.000000
41	46	45	1	52.000000	550.000000
45	46	47	1	136.000000	100.000000
45	46	48	1	136.000000	100.000000
45	50	51	1	136.000000	100.000000
45	50	52	1	136.000000	100.000000
46	45	50	1	120.000000	25.000000
45	50	49	1	52.000000	550.000000
49	50	51	1	136.000000	100.000000
49	50	52	1	136.000000	100.000000
49	54	55	1	136.000000	100.000000
49	54	56	1	136.000000	100.000000
50	49	54	1	120.000000	25.000000
49	54	53	1	52.000000	550.000000
53	54	55	1	136.000000	100.000000
53	54	56	1	136.000000	100.000000
53	58	59	1	136.000000	100.000000
53	58	60	1	136.000000	100.000000
54	53	58	1	120.000000	25.000000
53	58	57	1	52.000000	550.000000
57	58	59	1	136.000000	100.000000
57	58	60	1	136.000000	100.000000
57	62	63	1	136.000000	100.000000
57	62	64	1	136.000000	100.000000
58	57	62	1	120.000000	25.000000
57	62	61	1	52.000000	550.000000
61	62	63	1	136.000000	100.000000
61	62	64	1	136.000000	100.000000
61	66	67	1	136.000000	100.000000
61	66	68	1	136.000000	100.000000
62	61	66	1	120.000000	25.000000
61	66	65	1	52.000000	550.000000
65	66	67	1	136.000000	100.000000
65	66	68	1	136.000000	100.000000
65	70	71	1	136.000000	100.000000

65	70	72	1	136.000000	100.000000
66	65	70	1	120.000000	25.000000
65	70	69	1	52.000000	550.000000
69	70	71	1	136.000000	100.000000
69	70	72	1	136.000000	100.000000
69	74	75	1	136.000000	100.000000
69	74	76	1	136.000000	100.000000
70	69	74	1	120.000000	25.000000
69	74	73	1	52.000000	550.000000
73	74	75	1	136.000000	100.000000
73	74	76	1	136.000000	100.000000
73	78	79	1	136.000000	100.000000
73	78	80	1	136.000000	100.000000
74	73	78	1	120.000000	25.000000
73	78	77	1	52.000000	550.000000
77	78	79	1	136.000000	100.000000
77	78	80	1	136.000000	100.000000
77	82	83	1	136.000000	100.000000
77	82	84	1	136.000000	100.000000
78	77	82	1	120.000000	25.000000
77	82	81	1	52.000000	550.000000
81	82	83	1	136.000000	100.000000
81	82	84	1	136.000000	100.000000
81	86	87	1	136.000000	100.000000
81	86	88	1	136.000000	100.000000
82	81	86	1	120.000000	25.000000
81	86	85	1	52.000000	550.000000
85	86	87	1	136.000000	100.000000
85	86	88	1	136.000000	100.000000
85	90	91	1	136.000000	100.000000
85	90	92	1	136.000000	100.000000
86	85	90	1	120.000000	25.000000
85	90	89	1	52.000000	550.000000
89	90	91	1	136.000000	100.000000
89	90	92	1	136.000000	100.000000
89	94	95	1	136.000000	100.000000
89	94	96	1	136.000000	100.000000
90	89	94	1	120.000000	25.000000
89	94	93	1	52.000000	550.000000
93	94	95	1	136.000000	100.000000
93	94	96	1	136.000000	100.000000
93	98	99	1	136.000000	100.000000
93	98	100	1	136.000000	100.000000
94	93	98	1	120.000000	25.000000
93	98	97	1	52.000000	550.000000
97	98	99	1	136.000000	100.000000
97	98	100	1	136.000000	100.000000
97	102	103	1	136.000000	100.000000
97	102	104	1	136.000000	100.000000
98	97	102	1	120.000000	25.000000
97	102	101	1	52.000000	550.000000
101	102	103	1	136.000000	100.000000
101	102	104	1	136.000000	100.000000
101	106	107	1	136.000000	100.000000
101	106	108	1	136.000000	100.000000
102	101	106	1	120.000000	25.000000
101	106	105	1	52.000000	550.000000
105	106	107	1	136.000000	100.000000
105	106	108	1	136.000000	100.000000
105	110	111	1	136.000000	100.000000
105	110	112	1	136.000000	100.000000
106	105	110	1	120.000000	25.000000
105	110	109	1	52.000000	550.000000
109	110	111	1	136.000000	100.000000
109	110	112	1	136.000000	100.000000
109	114	115	1	136.000000	100.000000
109	114	116	1	136.000000	100.000000
110	109	114	1	120.000000	25.000000
109	114	113	1	52.000000	550.000000
113	114	115	1	136.000000	100.000000
113	114	116	1	136.000000	100.000000
113	118	119	1	136.000000	100.000000
113	118	120	1	136.000000	100.000000

114	113	118	1	120.000000	25.000000
113	118	117	1	52.000000	550.000000
117	118	119	1	136.000000	100.000000
117	118	120	1	136.000000	100.000000
117	122	123	1	136.000000	100.000000
117	122	124	1	136.000000	100.000000
118	117	122	1	120.000000	25.000000
117	122	121	1	52.000000	550.000000
121	122	123	1	136.000000	100.000000
121	122	124	1	136.000000	100.000000
121	126	127	1	136.000000	100.000000
121	126	128	1	136.000000	100.000000
122	121	126	1	120.000000	25.000000
121	126	125	1	52.000000	550.000000
125	126	127	1	136.000000	100.000000
125	126	128	1	136.000000	100.000000
125	130	131	1	136.000000	100.000000
125	130	132	1	136.000000	100.000000
126	125	130	1	120.000000	25.000000
125	130	129	1	52.000000	550.000000
129	130	131	1	136.000000	100.000000
129	130	132	1	136.000000	100.000000
129	134	135	1	136.000000	100.000000
129	134	136	1	136.000000	100.000000
130	129	134	1	120.000000	25.000000
129	134	133	1	52.000000	550.000000
133	134	135	1	136.000000	100.000000
133	134	136	1	136.000000	100.000000
133	138	139	1	136.000000	100.000000
133	138	140	1	136.000000	100.000000
134	133	138	1	120.000000	25.000000
133	138	137	1	52.000000	550.000000
137	138	139	1	136.000000	100.000000
137	138	140	1	136.000000	100.000000
137	142	143	1	136.000000	100.000000
137	142	144	1	136.000000	100.000000
138	137	142	1	120.000000	25.000000
137	142	141	1	52.000000	550.000000
141	142	143	1	136.000000	100.000000
141	142	144	1	136.000000	100.000000
141	146	147	1	136.000000	100.000000
141	146	148	1	136.000000	100.000000
142	141	146	1	120.000000	25.000000
141	146	145	1	52.000000	550.000000
145	146	147	1	136.000000	100.000000
145	146	148	1	136.000000	100.000000
145	150	151	1	136.000000	100.000000
145	150	152	1	136.000000	100.000000
146	145	150	1	120.000000	25.000000
145	150	149	1	52.000000	550.000000
149	150	151	1	136.000000	100.000000
149	150	152	1	136.000000	100.000000
149	154	155	1	136.000000	100.000000
149	154	156	1	136.000000	100.000000
150	149	154	1	120.000000	25.000000
149	154	153	1	52.000000	550.000000
153	154	155	1	136.000000	100.000000
153	154	156	1	136.000000	100.000000
153	158	159	1	136.000000	100.000000
153	158	160	1	136.000000	100.000000
154	153	158	1	120.000000	25.000000
153	158	157	1	52.000000	550.000000
157	158	159	1	136.000000	100.000000
157	158	160	1	136.000000	100.000000
157	162	163	1	136.000000	100.000000
157	162	164	1	136.000000	100.000000
158	157	162	1	120.000000	25.000000
157	162	161	1	52.000000	550.000000
161	162	163	1	136.000000	100.000000
161	162	164	1	136.000000	100.000000
161	166	167	1	136.000000	100.000000
161	166	168	1	136.000000	100.000000
162	161	166	1	120.000000	25.000000

161	166	165	1	52.000000	550.000000
165	166	167	1	136.000000	100.000000
165	166	168	1	136.000000	100.000000
165	170	171	1	136.000000	100.000000
165	170	172	1	136.000000	100.000000
166	165	170	1	120.000000	25.000000
165	170	169	1	52.000000	550.000000
169	170	171	1	136.000000	100.000000
169	170	172	1	136.000000	100.000000
169	174	175	1	136.000000	100.000000
169	174	176	1	136.000000	100.000000
170	169	174	1	120.000000	25.000000
169	174	173	1	52.000000	550.000000
173	174	175	1	136.000000	100.000000
173	174	176	1	136.000000	100.000000
173	178	179	1	136.000000	100.000000
173	178	180	1	136.000000	100.000000
174	173	178	1	120.000000	25.000000
173	178	177	1	52.000000	550.000000
177	178	179	1	136.000000	100.000000
177	178	180	1	136.000000	100.000000
177	182	183	1	136.000000	100.000000
177	182	184	1	136.000000	100.000000
178	177	182	1	120.000000	25.000000
177	182	181	1	52.000000	550.000000
181	182	183	1	136.000000	100.000000
181	182	184	1	136.000000	100.000000
181	186	187	1	136.000000	100.000000
181	186	188	1	136.000000	100.000000
182	181	186	1	120.000000	25.000000
181	186	185	1	52.000000	550.000000
185	186	187	1	136.000000	100.000000
185	186	188	1	136.000000	100.000000
185	190	191	1	136.000000	100.000000
185	190	192	1	136.000000	100.000000
186	185	190	1	120.000000	25.000000
185	190	189	1	52.000000	550.000000
189	190	191	1	136.000000	100.000000
189	190	192	1	136.000000	100.000000
189	194	195	1	136.000000	100.000000
189	194	196	1	136.000000	100.000000
190	189	194	1	120.000000	25.000000
189	194	193	1	52.000000	550.000000
193	194	195	1	136.000000	100.000000
193	194	196	1	136.000000	100.000000
193	198	199	1	136.000000	100.000000
193	198	200	1	136.000000	100.000000
194	193	198	1	120.000000	25.000000
193	198	197	1	52.000000	550.000000
197	198	199	1	136.000000	100.000000
197	198	200	1	136.000000	100.000000

2.1 Polypropylene NP Coordinates

```
POLYPROPYLENE
 1800
    1IPP      C1    1   2.665   5.221   6.357  0.3598 -0.3395  0.2734
    1IPP      C2    2   2.734   5.151   6.076  0.3428 -0.3337  0.2622
    1IPP      C3    3   2.733   4.862   6.007  0.3849 -0.3373  0.2714
    1IPP      C4    4   2.974   4.692   6.038  0.4110 -0.2847  0.2394
    1IPP      C5    5   3.196   4.656   5.844  0.3884 -0.2649  0.2280
    1IPP      C6    6   3.463   4.784   5.863  0.3785 -0.2218  0.1985
    1IPP      C7    7   3.547   4.998   5.676  0.3249 -0.2133  0.1942
    1IPP      C8    8   3.730   4.939   5.451  0.3144 -0.1917  0.1600
    1IPP      C9    9   4.028   4.971   5.453  0.3037 -0.1449  0.1329
    1IPP     C10   10   4.227   4.750   5.472  0.3466 -0.1083  0.1263
    1IPP     C11   11   4.380   4.678   5.716  0.3835 -0.0786  0.1093
    1IPP     C12   12   4.236   4.511   5.914  0.4270 -0.0937  0.1315
    1IPP     C13   13   4.059   4.646   6.107  0.4240 -0.1278  0.1349
    1IPP     C14   14   3.937   4.850   5.930  0.3806 -0.1452  0.1515
    1IPP     C15   15   4.164   5.037   5.886  0.3404 -0.1081  0.1230
    1IPP     C16   16   4.265   5.191   6.118  0.3430 -0.0937  0.1073
    1IPP     C17   17   4.056   5.387   6.195  0.3125 -0.1201  0.1247
    1IPP     C18   18   3.827   5.260   6.334  0.3485 -0.1472  0.1516
    1IPP     C19   19   3.641   5.100   6.165  0.3711 -0.1900  0.1677
    1IPP     C20   20   3.372   5.174   6.063  0.3411 -0.2299  0.1894
    1IPP     C21   21   3.152   5.077   6.235  0.3668 -0.2601  0.2251
    1IPP     C22   22   3.279   4.857   6.385  0.4251 -0.2368  0.2192
    1IPP     C23   23   3.348   4.588   6.282  0.4452 -0.2303  0.2085
    1IPP     C24   24   3.631   4.521   6.224  0.4659 -0.1866  0.1847
    1IPP     C25   25   3.667   4.395   5.959  0.4530 -0.1868  0.1863
    1IPP     C26   26   3.526   4.137   5.993  0.4938 -0.2092  0.2103
    1IPP     C27   27   3.409   4.101   6.266  0.5298 -0.2139  0.2186
    1IPP     C28   28   3.114   4.112   6.315  0.5344 -0.2674  0.2484
    1IPP     C29   29   2.962   4.362   6.369  0.5002 -0.2871  0.2649
    1IPP     C30   30   2.990   4.414   6.660  0.5230 -0.2812  0.2544
    1IPP     C31   31   3.247   4.548   6.726  0.4998 -0.2268  0.2238
    1IPP     C32   32   3.497   4.402   6.662  0.5201 -0.1988  0.2020
    1IPP     C33   33   3.581   4.203   6.865  0.5710 -0.1824  0.2068
    1IPP     C34   34   3.644   4.394   7.083  0.5626 -0.1676  0.1923
    1IPP     C35   35   3.819   4.616   6.991  0.5163 -0.1272  0.1648
    1IPP     C36   36   4.110   4.607   7.046  0.5298 -0.0930  0.1378
    1IPP     C37   37   4.169   4.745   7.303  0.5301 -0.0737  0.1275
    1IPP     C38   38   4.143   5.039   7.350  0.4830 -0.0909  0.1296
    1IPP     C39   39   4.326   5.248   7.243  0.4425 -0.0484  0.1004
    1IPP     C40   40   4.589   5.214   7.375  0.4614 -0.0114  0.0708
    1IPP     C41   41   4.695   5.018   7.180  0.4824 -0.0061  0.0644
    1IPP     C42   42   4.601   4.736   7.179  0.5203  0.0066  0.0786
    1IPP     C43   43   4.773   4.517   7.279  0.5672  0.0033  0.0723
    1IPP     C44   44   4.917   4.424   7.038  0.5554  0.0386  0.0666
    1IPP     C45   45   4.764   4.263   6.841  0.5512 -0.0014  0.0748
    1IPP     C46   46   4.687   4.396   6.586  0.5220 -0.0154  0.0866
    1IPP     C47   47   4.421   4.525   6.562  0.4766 -0.0504  0.1059
    1IPP     C48   48   4.405   4.817   6.601  0.4445 -0.0628  0.1072
    1IPP     C49   49   4.346   4.940   6.864  0.4579 -0.0656  0.1043
    1IPP     C50   50   4.059   5.016   6.877  0.4492 -0.1059  0.1382
    1IPP     C51   51   3.972   5.275   6.762  0.3909 -0.1214  0.1352
    1IPP     C52   52   4.032   5.483   6.964  0.3736 -0.1148  0.1262
    1IPP     C53   53   3.901   5.452   7.229  0.4091 -0.1141  0.1395
    1IPP     C54   54   3.624   5.534   7.294  0.4171 -0.1651  0.1648
    1IPP     C55   55   3.390   5.350   7.314  0.4310 -0.2088  0.1890
    1IPP     C56   56   3.213   5.307   7.078  0.4201 -0.2392  0.2117
    1IPP     C57   57   2.962   5.451   7.008  0.3884 -0.2768  0.2371
    1IPP     C58   58   2.691   5.332   7.032  0.4230 -0.3081  0.2668
    1IPP     C59   59   2.578   5.174   6.805  0.4139 -0.3355  0.2705
    1IPP     C60   60   2.461   4.905   6.858  0.4595 -0.3614  0.2949
    1IPP     C61   61   2.626   4.658   6.870  0.5016 -0.3275  0.2838
    1IPP     C62   62   2.745   4.692   7.139  0.5186 -0.3093  0.2709
    1IPP     C63   63   2.926   4.928   7.154  0.4845 -0.2644  0.2494
    1IPP     C64   64   3.199   4.853   7.063  0.4901 -0.2348  0.2221
    1IPP     C65   65   3.383   4.721   7.254  0.5257 -0.1978  0.2132
    1IPP     C66   66   3.540   4.923   7.403  0.5173 -0.1733  0.1874
    1IPP     C67   67   3.733   5.023   7.202  0.4774 -0.1528  0.1688
    1IPP     C68   68   3.602   5.128   6.958  0.4312 -0.1743  0.1723
```

1IPP	C69	69	3.537	4.909	6.770	0.4404	-0.1902	0.1893
1IPP	C70	70	3.716	4.854	6.540	0.4448	-0.1648	0.1640
1IPP	C71	71	3.969	4.699	6.553	0.4656	-0.1244	0.1433
1IPP	C72	72	3.965	4.403	6.536	0.5024	-0.1267	0.1530
1IPP	C73	73	4.021	4.242	6.780	0.5585	-0.1158	0.1557
1IPP	C74	74	4.306	4.185	6.839	0.5732	-0.0681	0.1245
1IPP	C75	75	4.389	3.969	6.652	0.5911	-0.0631	0.1198
1IPP	C76	76	4.485	4.049	6.382	0.5423	-0.0493	0.1113
1IPP	C77	77	4.292	4.233	6.252	0.5163	-0.0830	0.1262
1IPP	C78	78	4.031	4.161	6.129	0.4948	-0.1301	0.1488
1IPP	C79	79	4.015	4.078	5.843	0.4898	-0.1330	0.1644
1IPP	C80	80	4.055	4.269	5.618	0.4393	-0.1221	0.1556
1IPP	C81	81	3.877	4.505	5.581	0.4071	-0.1645	0.1634
1IPP	C82	82	3.590	4.484	5.509	0.3885	-0.2113	0.1840
1IPP	C83	83	3.489	4.373	5.252	0.3824	-0.2217	0.2087
1IPP	C84	84	3.468	4.515	4.990	0.3285	-0.2344	0.2080
1IPP	C85	85	3.262	4.716	4.916	0.3021	-0.2783	0.2203
1IPP	C86	86	3.329	5.001	4.862	0.2482	-0.2592	0.1953
1IPP	C87	87	3.433	5.087	5.124	0.2567	-0.2395	0.2017
1IPP	C88	88	3.217	5.030	5.315	0.2915	-0.2735	0.2134
1IPP	C89	89	3.240	4.749	5.406	0.3398	-0.2671	0.2162
1IPP	C90	90	3.058	4.537	5.303	0.3669	-0.3014	0.2390
1IPP	C91	91	2.777	4.528	5.400	0.3665	-0.3318	0.2640
1IPP	C92	92	2.731	4.501	5.693	0.4077	-0.3325	0.2776
1IPP	C93	93	2.783	4.243	5.829	0.4571	-0.3281	0.2754
1IPP	C94	94	3.073	4.242	5.890	0.4693	-0.2833	0.2597
1IPP	C95	95	3.218	4.245	5.630	0.4426	-0.2620	0.2385
1IPP	C96	96	3.357	4.000	5.536	0.4760	-0.2462	0.2243
1IPP	C97	97	3.652	4.023	5.564	0.4553	-0.2029	0.1983
1IPP	C98	98	3.830	4.046	5.327	0.4409	-0.1717	0.1786
1IPP	C99	99	4.054	3.851	5.326	0.4781	-0.1463	0.1652
1IPP	C100	100	4.296	3.893	5.496	0.4775	-0.1055	0.1394
1IPP	C1	101	2.158	5.092	4.695	0.2144	-0.4454	0.3248
1IPP	C2	102	2.180	5.385	4.738	0.1608	-0.4460	0.3175
1IPP	C3	103	2.249	5.495	5.003	0.1818	-0.4274	0.3072
1IPP	C4	104	2.022	5.523	5.191	0.2011	-0.4662	0.3286
1IPP	C5	105	1.890	5.779	5.263	0.1602	-0.4812	0.3296
1IPP	C6	106	1.999	5.954	5.478	0.1602	-0.4528	0.3181
1IPP	C7	107	1.935	5.901	5.763	0.1883	-0.4582	0.3319
1IPP	C8	108	1.724	6.037	5.921	0.1896	-0.4915	0.3431
1IPP	C9	109	1.784	6.305	6.034	0.1651	-0.4755	0.3385
1IPP	C10	110	1.963	6.331	6.270	0.1898	-0.4418	0.3050
1IPP	C11	111	2.231	6.453	6.232	0.1409	-0.4169	0.2896
1IPP	C12	112	2.223	6.750	6.261	0.1147	-0.4001	0.2798
1IPP	C13	113	2.158	6.922	6.027	0.0569	-0.4200	0.2781
1IPP	C14	114	2.004	6.819	5.794	0.0613	-0.4509	0.2975
1IPP	C15	115	2.126	6.640	5.590	0.0575	-0.4387	0.2968
1IPP	C16	116	2.075	6.366	5.692	0.1256	-0.4361	0.2970
1IPP	C17	117	2.269	6.215	5.857	0.1501	-0.4023	0.2865
1IPP	C18	118	2.380	5.974	5.727	0.1781	-0.3911	0.2860
1IPP	C19	119	2.547	6.075	5.507	0.1472	-0.3756	0.2587
1IPP	C20	120	2.371	6.214	5.314	0.1051	-0.4031	0.2765
1IPP	C21	121	2.286	5.997	5.132	0.1146	-0.4338	0.2807
1IPP	C22	122	2.507	5.851	4.999	0.1270	-0.3899	0.2780
1IPP	C23	123	2.582	5.903	4.717	0.0857	-0.3878	0.2658
1IPP	C24	124	2.408	5.749	4.532	0.0937	-0.4172	0.2805
1IPP	C25	125	2.166	5.916	4.557	0.0689	-0.4594	0.3119
1IPP	C26	126	2.011	5.868	4.804	0.1055	-0.4774	0.3168
1IPP	C27	127	1.810	5.652	4.790	0.1400	-0.5004	0.3458
1IPP	C28	128	1.547	5.727	4.673	0.1110	-0.5425	0.3702
1IPP	C29	129	1.601	5.819	4.396	0.0816	-0.5369	0.3621
1IPP	C30	130	1.767	6.064	4.417	0.0293	-0.5210	0.3393
1IPP	C31	131	1.677	6.348	4.399	-0.0135	-0.5325	0.3422
1IPP	C32	132	1.712	6.525	4.161	-0.0672	-0.5380	0.3322
1IPP	C33	133	1.977	6.662	4.149	-0.0821	-0.4864	0.3049
1IPP	C34	134	1.997	6.796	4.413	-0.0788	-0.4816	0.2984
1IPP	C35	135	2.020	7.085	4.480	-0.1111	-0.4781	0.2966
1IPP	C36	136	2.296	7.158	4.560	-0.1337	-0.4420	0.2567
1IPP	C37	137	2.357	7.034	4.823	-0.0716	-0.4178	0.2583
1IPP	C38	138	2.260	7.174	5.065	-0.0707	-0.4308	0.2551
1IPP	C39	139	2.465	7.323	5.219	-0.0803	-0.3885	0.2382
1IPP	C40	140	2.584	7.129	5.408	-0.0330	-0.3690	0.2367
1IPP	C41	141	2.390	7.035	5.610	-0.0014	-0.3946	0.2584

1IPP	C42	142	2.310	7.266	5.779	-0.0260	-0.3942	0.2510
1IPP	C43	143	2.531	7.327	5.966	0.0031	-0.3668	0.2386
1IPP	C44	144	2.575	7.090	6.139	0.0390	-0.3592	0.2328
1IPP	C45	145	2.678	6.837	6.019	0.0778	-0.3468	0.2269
1IPP	C46	146	2.482	6.657	5.888	0.0969	-0.3718	0.2591
1IPP	C47	147	2.564	6.501	5.652	0.0996	-0.3698	0.2436
1IPP	C48	148	2.602	6.660	5.406	0.0412	-0.3673	0.2408
1IPP	C49	149	2.394	6.740	5.210	0.0038	-0.3940	0.2560
1IPP	C50	150	2.291	6.532	5.025	0.0175	-0.4226	0.2725
1IPP	C51	151	2.502	6.403	4.860	0.0219	-0.4017	0.2590
1IPP	C52	152	2.615	6.640	4.723	-0.0271	-0.3713	0.2380
1IPP	C53	153	2.422	6.728	4.517	-0.0592	-0.4174	0.2638
1IPP	C54	154	2.445	6.711	4.223	-0.0846	-0.4185	0.2465
1IPP	C55	155	2.519	6.980	4.124	-0.1382	-0.4111	0.2473
1IPP	C56	156	2.259	7.119	4.078	-0.1675	-0.4404	0.2641
1IPP	C57	157	2.165	7.049	3.805	-0.1799	-0.4659	0.2771
1IPP	C58	158	2.251	6.766	3.773	-0.1399	-0.4606	0.2759
1IPP	C59	159	2.539	6.742	3.706	-0.1466	-0.4152	0.2399
1IPP	C60	160	2.650	6.569	3.491	-0.1407	-0.4012	0.2462
1IPP	C61	161	2.673	6.285	3.576	-0.0833	-0.3996	0.2377
1IPP	C62	162	2.901	6.174	3.731	-0.0547	-0.3574	0.2278
1IPP	C63	163	3.098	6.018	3.575	-0.0409	-0.3197	0.2097
1IPP	C64	164	3.059	5.723	3.569	-0.0045	-0.3461	0.2194
1IPP	C65	165	2.806	5.641	3.438	0.0001	-0.3792	0.2399
1IPP	C66	166	2.561	5.631	3.608	0.0114	-0.4050	0.2695
1IPP	C67	167	2.423	5.890	3.655	-0.0098	-0.4406	0.2834
1IPP	C68	168	2.493	6.042	3.901	-0.0180	-0.4178	0.2627
1IPP	C69	169	2.331	6.021	4.148	0.0125	-0.4265	0.2806
1IPP	C70	170	2.117	6.220	4.189	-0.0075	-0.4725	0.3108
1IPP	C71	171	2.187	6.371	4.433	-0.0130	-0.4524	0.2765
1IPP	C72	172	2.062	6.336	4.700	0.0195	-0.4542	0.3081
1IPP	C73	173	1.859	6.540	4.775	-0.0077	-0.5112	0.3176
1IPP	C74	174	1.927	6.812	4.876	-0.0328	-0.4785	0.3123
1IPP	C75	175	1.960	6.824	5.171	-0.0056	-0.4646	0.2994
1IPP	C76	176	1.747	6.656	5.292	0.0266	-0.4942	0.3257
1IPP	C77	177	1.837	6.374	5.320	0.0710	-0.4908	0.3260
1IPP	C78	178	1.863	6.191	5.088	0.0901	-0.4836	0.3257
1IPP	C79	179	1.619	6.059	4.980	0.0857	-0.5190	0.3523
1IPP	C80	180	1.464	5.842	5.117	0.1392	-0.5622	0.3772
1IPP	C81	181	1.526	5.550	5.122	0.1872	-0.5358	0.3805
1IPP	C82	182	1.567	5.391	5.372	0.2324	-0.5249	0.3746
1IPP	C83	183	1.808	5.362	5.545	0.2501	-0.4794	0.3469
1IPP	C84	184	2.020	5.151	5.520	0.2864	-0.4556	0.3319
1IPP	C85	185	2.274	5.191	5.369	0.2724	-0.4213	0.3086
1IPP	C86	186	2.339	5.060	5.110	0.2624	-0.4140	0.3129
1IPP	C87	187	2.532	4.834	5.108	0.2939	-0.3895	0.2965
1IPP	C88	188	2.821	4.907	5.089	0.2850	-0.3303	0.2656
1IPP	C89	189	2.874	4.930	4.797	0.2513	-0.3419	0.2474
1IPP	C90	190	2.876	5.191	4.655	0.1961	-0.3317	0.2428
1IPP	C91	191	2.612	5.324	4.623	0.1721	-0.3818	0.2753
1IPP	C92	192	2.461	5.239	4.383	0.1576	-0.4119	0.2801
1IPP	C93	193	2.560	5.348	4.125	0.1102	-0.3891	0.2675
1IPP	C94	194	2.442	5.609	4.052	0.0697	-0.4314	0.2896
1IPP	C95	195	2.165	5.625	3.944	0.0617	-0.4644	0.3084
1IPP	C96	196	1.975	5.728	4.148	0.0609	-0.4938	0.3315
1IPP	C97	197	1.992	5.539	4.375	0.1004	-0.4874	0.3249
1IPP	C98	198	1.835	5.291	4.427	0.1568	-0.5081	0.3532
1IPP	C99	199	1.650	5.262	4.659	0.1930	-0.5235	0.3850
1IPP	C100	200	1.770	5.207	4.927	0.2142	-0.5086	0.3564
1IPP	C1	201	5.242	5.864	5.488	0.1687	0.0512	0.0005
1IPP	C2	202	5.033	5.746	5.664	0.2196	0.0263	0.0103
1IPP	C3	203	5.042	5.750	5.963	0.2423	0.0287	0.0177
1IPP	C4	204	5.242	5.600	6.124	0.2777	0.0645	0.0010
1IPP	C5	205	5.260	5.311	6.184	0.3298	0.0739	0.0045
1IPP	C6	206	5.140	5.237	6.442	0.3647	0.0468	0.0044
1IPP	C7	207	5.288	5.326	6.683	0.3864	0.0776	0.0086
1IPP	C8	208	5.469	5.158	6.849	0.4174	0.1180	-0.0174
1IPP	C9	209	5.367	4.892	6.934	0.4730	0.1018	-0.0010
1IPP	C10	210	5.122	4.843	7.094	0.4993	0.0657	0.0271
1IPP	C11	211	5.101	4.846	7.390	0.5177	0.0738	0.0367
1IPP	C12	212	5.265	4.621	7.493	0.5831	0.0993	0.0266
1IPP	C13	213	5.217	4.353	7.375	0.5937	0.0897	0.0389
1IPP	C14	214	5.400	4.298	7.146	0.5946	0.1046	0.0233

1IPP	C15	215	5.343	4.417	6.879	0.5402	0.0875	0.0081
1IPP	C16	216	5.178	4.270	6.680	0.5457	0.0670	0.0357
1IPP	C17	217	5.305	4.120	6.458	0.5441	0.0801	0.0334
1IPP	C18	218	5.377	4.339	6.272	0.4954	0.0946	0.0149
1IPP	C19	219	5.626	4.479	6.348	0.4787	0.1170	-0.0243
1IPP	C20	220	5.564	4.674	6.563	0.4619	0.1185	-0.0038
1IPP	C21	221	5.461	4.937	6.475	0.4165	0.1110	-0.0059
1IPP	C22	222	5.636	5.163	6.389	0.3877	0.1400	-0.0289
1IPP	C23	223	5.796	5.338	6.567	0.3522	0.1532	-0.0478
1IPP	C24	224	5.673	5.558	6.726	0.3468	0.1385	-0.0403
1IPP	C25	225	5.543	5.793	6.598	0.2954	0.1213	-0.0352
1IPP	C26	226	5.257	5.852	6.652	0.2936	0.0733	-0.0102
1IPP	C27	227	5.033	5.691	6.541	0.3015	0.0351	0.0208
1IPP	C28	228	4.814	5.819	6.385	0.2752	-0.0018	0.0454
1IPP	C29	229	4.593	5.969	6.518	0.2593	-0.0294	0.0628
1IPP	C30	230	4.389	5.810	6.666	0.3138	-0.0519	0.0782
1IPP	C31	231	4.170	5.644	6.552	0.3160	-0.0984	0.1085
1IPP	C32	232	3.881	5.704	6.511	0.2906	-0.1503	0.1409
1IPP	C33	233	3.691	5.646	6.731	0.3331	-0.1690	0.1514
1IPP	C34	234	3.692	5.885	6.906	0.3082	-0.1662	0.1489
1IPP	C35	235	3.890	5.913	7.126	0.3441	-0.1182	0.1278
1IPP	C36	236	4.179	5.943	7.061	0.3132	-0.0869	0.1057
1IPP	C37	237	4.379	5.729	7.113	0.3634	-0.0410	0.0801
1IPP	C38	238	4.542	5.768	7.359	0.3692	-0.0206	0.0621
1IPP	C39	239	4.766	5.964	7.344	0.3591	0.0141	0.0368
1IPP	C40	240	5.059	5.910	7.343	0.3512	0.0678	0.0134
1IPP	C41	241	5.252	5.890	7.117	0.3427	0.0876	-0.0014
1IPP	C42	242	5.305	5.614	7.023	0.3671	0.0958	-0.0122
1IPP	C43	243	5.457	5.428	7.199	0.4195	0.1206	-0.0148
1IPP	C44	244	5.753	5.399	7.198	0.4242	0.1643	-0.0573
1IPP	C45	245	5.902	5.235	7.000	0.4214	0.1908	-0.0513
1IPP	C46	246	6.152	5.363	6.899	0.3997	0.2208	-0.0876
1IPP	C47	247	6.132	5.602	6.725	0.3420	0.2165	-0.0884
1IPP	C48	248	6.085	5.826	6.916	0.3323	0.2176	-0.0951
1IPP	C49	249	5.808	5.861	7.022	0.3272	0.1563	-0.0577
1IPP	C50	250	5.647	6.093	6.928	0.2828	0.1483	-0.0582
1IPP	C51	251	5.608	6.317	7.121	0.2665	0.1471	-0.0466
1IPP	C52	252	5.343	6.314	7.254	0.2835	0.1056	-0.0268
1IPP	C53	253	5.120	6.343	7.060	0.2528	0.0717	-0.0019
1IPP	C54	254	5.039	6.613	6.968	0.2060	0.0457	-0.0029
1IPP	C55	255	4.823	6.764	7.105	0.2026	0.0173	0.0221
1IPP	C56	256	4.544	6.692	7.026	0.1967	-0.0278	0.0453
1IPP	C57	257	4.379	6.839	6.827	0.1639	-0.0541	0.0648
1IPP	C58	258	4.435	6.817	6.537	0.1402	-0.0442	0.0551
1IPP	C59	259	4.334	6.612	6.346	0.1367	-0.0803	0.0580
1IPP	C60	260	4.496	6.367	6.317	0.1809	-0.0517	0.0516
1IPP	C61	261	4.747	6.441	6.177	0.1470	-0.0085	0.0294
1IPP	C62	262	4.759	6.414	5.885	0.1320	-0.0170	0.0343
1IPP	C63	263	4.835	6.130	5.864	0.1731	0.0048	0.0301
1IPP	C64	264	4.615	5.931	5.855	0.1940	-0.0454	0.0488
1IPP	C65	265	4.473	5.859	6.107	0.2306	-0.0613	0.0765
1IPP	C66	266	4.189	5.897	6.184	0.2397	-0.1007	0.1022
1IPP	C67	267	4.078	6.118	6.347	0.2166	-0.1078	0.1091
1IPP	C68	268	4.047	6.105	6.642	0.2504	-0.1100	0.1177
1IPP	C69	269	4.181	6.324	6.790	0.2314	-0.0897	0.0885
1IPP	C70	270	4.477	6.328	6.780	0.2419	-0.0500	0.0655
1IPP	C71	271	4.673	6.204	6.967	0.2686	-0.0066	0.0368
1IPP	C72	272	4.864	5.991	6.884	0.3060	0.0243	0.0276
1IPP	C73	273	4.771	5.709	6.899	0.3327	0.0072	0.0458
1IPP	C74	274	4.820	5.505	7.109	0.3879	0.0208	0.0484
1IPP	C75	275	4.997	5.291	7.004	0.4157	0.0445	0.0329
1IPP	C76	276	4.871	5.139	6.780	0.4111	0.0109	0.0406
1IPP	C77	277	4.816	4.848	6.771	0.4669	0.0164	0.0618
1IPP	C78	278	5.048	4.702	6.657	0.4646	0.0382	0.0419
1IPP	C79	279	5.043	4.624	6.371	0.4496	0.0358	0.0417
1IPP	C80	280	5.139	4.812	6.162	0.4060	0.0434	0.0250
1IPP	C81	281	4.925	5.010	6.109	0.3810	0.0117	0.0441
1IPP	C82	282	4.672	4.932	5.977	0.3713	-0.0333	0.0741
1IPP	C83	283	4.518	4.755	6.158	0.4161	-0.0568	0.0871
1IPP	C84	284	4.666	4.500	6.122	0.4410	-0.0258	0.0830
1IPP	C85	285	4.645	4.337	5.872	0.4544	-0.0444	0.0873
1IPP	C86	286	4.519	4.070	5.883	0.4830	-0.0516	0.1057
1IPP	C87	287	4.744	3.923	6.010	0.5412	-0.0181	0.0945

1IPP	C88	288	4.997	3.929	5.853	0.5092	0.0085	0.0573
1IPP	C89	289	5.194	4.147	5.902	0.4891	0.0545	0.0309
1IPP	C90	290	5.081	4.421	5.936	0.4461	0.0214	0.0451
1IPP	C91	291	4.989	4.626	5.740	0.3878	0.0133	0.0495
1IPP	C92	292	5.180	4.833	5.643	0.3499	0.0499	0.0296
1IPP	C93	293	5.256	5.069	5.804	0.3249	0.0605	0.0082
1IPP	C94	294	5.100	5.312	5.738	0.2928	0.0237	0.0221
1IPP	C95	295	4.810	5.332	5.797	0.2924	-0.0074	0.0523
1IPP	C96	296	4.682	5.483	6.017	0.2918	-0.0246	0.0534
1IPP	C97	297	4.757	5.391	6.287	0.3233	-0.0094	0.0589
1IPP	C98	298	4.619	5.158	6.407	0.3868	-0.0304	0.0690
1IPP	C99	299	4.389	5.266	6.559	0.3661	-0.0518	0.0891
1IPP	C100	300	4.464	5.373	6.825	0.3869	-0.0473	0.0848
1IPP	C1	301	5.582	5.401	5.868	0.2840	0.1010	-0.0330
1IPP	C2	302	5.823	5.229	5.865	0.3035	0.1456	-0.0491
1IPP	C3	303	5.845	4.979	5.704	0.3329	0.1450	-0.0534
1IPP	C4	304	5.996	4.953	5.450	0.3067	0.1674	-0.0653
1IPP	C5	305	5.858	4.911	5.192	0.2890	0.1376	-0.0430
1IPP	C6	306	5.715	4.653	5.220	0.3439	0.1079	-0.0298
1IPP	C7	307	5.845	4.390	5.175	0.3724	0.1382	-0.0318
1IPP	C8	308	5.840	4.294	4.895	0.3623	0.1318	-0.0295
1IPP	C9	309	5.571	4.186	4.824	0.3648	0.0781	-0.0038
1IPP	C10	310	5.494	3.936	4.968	0.4216	0.0772	0.0221
1IPP	C11	311	5.260	3.894	5.149	0.4499	0.0438	0.0402
1IPP	C12	312	5.238	3.973	5.435	0.4646	0.0455	0.0385
1IPP	C13	313	5.039	4.188	5.484	0.4308	0.0030	0.0558
1IPP	C14	314	4.771	4.061	5.482	0.4623	-0.0264	0.0810
1IPP	C15	315	4.747	3.995	5.193	0.4339	-0.0390	0.0822
1IPP	C16	316	4.788	4.230	5.016	0.3859	-0.0278	0.0823
1IPP	C17	317	5.063	4.259	4.906	0.3606	-0.0019	0.0401
1IPP	C18	318	5.264	4.349	5.105	0.3751	0.0429	0.0307
1IPP	C19	319	5.174	4.570	5.282	0.3607	0.0388	0.0342
1IPP	C20	320	5.188	4.848	5.187	0.3072	0.0275	0.0176
1IPP	C21	321	5.435	5.011	5.205	0.2752	0.0709	0.0007
1IPP	C22	322	5.507	5.165	5.447	0.2859	0.0962	-0.0219
1IPP	C23	323	5.426	5.450	5.461	0.2407	0.0664	-0.0113
1IPP	C24	324	5.651	5.619	5.370	0.1992	0.1042	-0.0491
1IPP	C25	325	5.886	5.570	5.546	0.2330	0.1494	-0.0663
1IPP	C26	326	6.121	5.394	5.491	0.2431	0.1892	-0.0808
1IPP	C27	327	6.358	5.523	5.367	0.2161	0.2262	-0.1036
1IPP	C28	328	6.372	5.478	5.074	0.2025	0.2113	-0.1163
1IPP	C29	329	6.619	5.430	4.915	0.1856	0.2459	-0.1328
1IPP	C30	330	6.706	5.662	4.752	0.1244	0.2641	-0.1529
1IPP	C31	331	6.775	5.926	4.870	0.1098	0.2853	-0.1552
1IPP	C32	332	6.509	6.044	4.922	0.0904	0.2269	-0.1360
1IPP	C33	333	6.343	6.051	4.675	0.0675	0.2057	-0.1256
1IPP	C34	334	6.168	5.811	4.649	0.0951	0.1741	-0.0957
1IPP	C35	335	6.257	5.525	4.655	0.1517	0.1783	-0.1039
1IPP	C36	336	6.387	5.371	4.436	0.1438	0.2005	-0.1108
1IPP	C37	337	6.682	5.318	4.425	0.1520	0.2447	-0.1350
1IPP	C38	338	6.874	5.533	4.349	0.1120	0.2800	-0.1692
1IPP	C39	339	6.896	5.684	4.093	0.0633	0.2662	-0.1703
1IPP	C40	340	7.074	5.620	3.864	0.0471	0.3004	-0.1853
1IPP	C41	341	6.932	5.590	3.605	0.0225	0.2687	-0.1661
1IPP	C42	342	6.765	5.348	3.566	0.0649	0.2435	-0.1426
1IPP	C43	343	6.522	5.370	3.735	0.0714	0.2073	-0.1191
1IPP	C44	344	6.560	5.253	4.006	0.1228	0.2235	-0.1412
1IPP	C45	345	6.430	4.999	4.090	0.1730	0.2017	-0.1087
1IPP	C46	346	6.144	5.018	4.172	0.1724	0.1577	-0.0782
1IPP	C47	347	6.041	5.050	4.449	0.1998	0.1530	-0.0653
1IPP	C48	348	5.992	4.808	4.618	0.2484	0.1511	-0.0598
1IPP	C49	349	5.731	4.665	4.638	0.2821	0.0973	-0.0265
1IPP	C50	350	5.521	4.751	4.832	0.2800	0.0884	-0.0019
1IPP	C51	351	5.323	4.962	4.763	0.2471	0.0430	0.0087
1IPP	C52	352	5.427	5.240	4.748	0.1949	0.0638	-0.0132
1IPP	C53	353	5.438	5.406	4.994	0.1940	0.0602	-0.0115
1IPP	C54	354	5.240	5.613	5.072	0.1745	0.0437	0.0045
1IPP	C55	355	5.311	5.881	4.970	0.1297	0.0442	-0.0021
1IPP	C56	356	5.547	6.014	5.088	0.1060	0.0802	-0.0484
1IPP	C57	357	5.513	6.201	5.316	0.1056	0.0835	-0.0379
1IPP	C58	358	5.705	6.216	5.541	0.1298	0.1224	-0.0617
1IPP	C59	359	5.692	5.950	5.669	0.1808	0.1140	-0.0592
1IPP	C60	360	5.496	5.866	5.873	0.2094	0.0950	-0.0274

1IPP	C61	361	5.593	5.866	6.153	0.2367	0.1196	-0.0406
1IPP	C62	362	5.731	5.617	6.231	0.2774	0.1383	-0.0440
1IPP	C63	363	6.026	5.604	6.215	0.2943	0.1875	-0.0790
1IPP	C64	364	6.131	5.560	5.941	0.2733	0.2005	-0.0949
1IPP	C65	365	6.283	5.751	5.771	0.2224	0.2125	-0.1061
1IPP	C66	366	6.168	5.973	5.608	0.1740	0.2005	-0.1071
1IPP	C67	367	6.015	5.954	5.354	0.1502	0.1643	-0.0979
1IPP	C68	368	6.120	5.864	5.093	0.1397	0.1846	-0.0898
1IPP	C69	369	5.898	5.698	4.986	0.1473	0.1328	-0.0735
1IPP	C70	370	5.883	5.411	5.058	0.1996	0.1436	-0.0592
1IPP	C71	371	5.957	5.202	4.863	0.2204	0.1376	-0.0661
1IPP	C72	372	6.215	5.067	4.917	0.2442	0.1931	-0.0888
1IPP	C73	373	6.452	5.062	4.738	0.2222	0.2189	-0.1132
1IPP	C74	374	6.465	4.854	4.527	0.2357	0.2149	-0.1057
1IPP	C75	375	6.551	4.604	4.662	0.2803	0.2308	-0.1060
1IPP	C76	376	6.320	4.476	4.797	0.3269	0.2057	-0.0781
1IPP	C77	377	6.188	4.637	5.008	0.3141	0.1829	-0.0788
1IPP	C78	378	6.305	4.680	5.278	0.3371	0.2137	-0.0908
1IPP	C79	379	6.252	4.461	5.471	0.3928	0.2052	-0.0728
1IPP	C80	380	5.986	4.479	5.599	0.4064	0.1632	-0.0470
1IPP	C81	381	5.953	4.530	5.888	0.4196	0.1563	-0.0318
1IPP	C82	382	5.928	4.278	6.042	0.4769	0.1751	-0.0403
1IPP	C83	383	5.662	4.157	5.990	0.4963	0.1270	-0.0118
1IPP	C84	384	5.629	3.966	5.765	0.4967	0.1193	-0.0043
1IPP	C85	385	5.684	4.121	5.518	0.4411	0.1182	-0.0044
1IPP	C86	386	5.490	4.345	5.505	0.4148	0.0760	0.0081
1IPP	C87	387	5.553	4.605	5.633	0.3930	0.1041	-0.0116
1IPP	C88	388	5.496	4.622	5.924	0.4078	0.0943	0.0009
1IPP	C89	389	5.607	4.859	6.064	0.3889	0.1182	-0.0187
1IPP	C90	390	5.890	4.867	6.156	0.4001	0.1684	-0.0514
1IPP	C91	391	5.978	4.755	6.417	0.4406	0.1727	-0.0587
1IPP	C92	392	6.023	4.956	6.630	0.4389	0.2005	-0.0574
1IPP	C93	393	6.283	5.086	6.571	0.3996	0.2292	-0.0875
1IPP	C94	394	6.305	5.289	6.355	0.3568	0.2389	-0.1108
1IPP	C95	395	6.419	5.241	6.085	0.3231	0.2396	-0.1031
1IPP	C96	396	6.280	5.138	5.841	0.3328	0.2290	-0.0957
1IPP	C97	397	6.276	4.847	5.773	0.3644	0.2129	-0.0867
1IPP	C98	398	6.549	4.737	5.752	0.3769	0.2560	-0.1124
1IPP	C99	399	6.703	4.735	5.499	0.3588	0.2784	-0.1242
1IPP	C100	400	6.709	4.469	5.365	0.3790	0.2671	-0.1178
1IPP	C1	401	6.139	9.077	5.825	-0.2846	0.1964	-0.1742
1IPP	C2	402	6.391	9.181	5.707	-0.3258	0.2343	-0.1916
1IPP	C3	403	6.468	9.050	5.452	-0.3234	0.2412	-0.2121
1IPP	C4	404	6.637	8.808	5.424	-0.2875	0.2622	-0.2189
1IPP	C5	405	6.497	8.546	5.433	-0.2419	0.2461	-0.1988
1IPP	C6	406	6.476	8.381	5.679	-0.2052	0.2369	-0.1836
1IPP	C7	407	6.720	8.270	5.803	-0.1574	0.2835	-0.2067
1IPP	C8	408	6.839	8.034	5.669	-0.1509	0.3013	-0.2216
1IPP	C9	409	7.116	8.069	5.570	-0.1582	0.3450	-0.2529
1IPP	C10	410	7.353	7.931	5.682	-0.1321	0.3973	-0.2585
1IPP	C11	411	7.463	8.030	5.938	-0.1133	0.3981	-0.2754
1IPP	C12	412	7.300	7.920	6.160	-0.0854	0.3939	-0.2601
1IPP	C13	413	7.041	8.036	6.078	-0.1007	0.3394	-0.2373
1IPP	C14	414	7.095	8.326	6.075	-0.1477	0.3488	-0.2451
1IPP	C15	415	7.147	8.452	5.813	-0.1923	0.3602	-0.2588
1IPP	C16	416	7.424	8.466	5.706	-0.2058	0.3862	-0.2784
1IPP	C17	417	7.564	8.723	5.755	-0.2360	0.4125	-0.3095
1IPP	C18	418	7.490	8.968	5.601	-0.2946	0.3975	-0.3084
1IPP	C19	419	7.303	9.175	5.708	-0.3234	0.3727	-0.2849
1IPP	C20	420	7.006	9.180	5.681	-0.3295	0.3336	-0.2570
1IPP	C21	421	6.859	9.033	5.893	-0.2811	0.3126	-0.2569
1IPP	C22	422	6.782	8.750	5.857	-0.2415	0.2972	-0.2262
1IPP	C23	423	6.487	8.765	5.848	-0.2373	0.2522	-0.2036
1IPP	C24	424	6.325	8.665	6.074	-0.2037	0.2290	-0.1781
1IPP	C25	425	6.268	8.379	6.124	-0.1599	0.2300	-0.1694
1IPP	C26	426	6.092	8.237	5.933	-0.1545	0.1881	-0.1450
1IPP	C27	427	5.799	8.272	5.920	-0.1589	0.1463	-0.1153
1IPP	C28	428	5.699	8.126	6.157	-0.1085	0.1381	-0.1034
1IPP	C29	429	5.814	7.858	6.193	-0.0819	0.1516	-0.1139
1IPP	C30	430	6.111	7.849	6.184	-0.0531	0.1996	-0.1333
1IPP	C31	431	6.260	7.868	6.438	-0.0445	0.2390	-0.1614
1IPP	C32	432	6.302	8.130	6.569	-0.0723	0.2331	-0.1701
1IPP	C33	433	6.084	8.110	6.769	-0.0521	0.2128	-0.1465

1IPP	C34	434	5.841	8.018	6.626	-0.0371	0.1626	-0.1065
1IPP	C35	435	5.772	7.729	6.655	0.0031	0.1624	-0.1049
1IPP	C36	436	5.974	7.531	6.566	0.0259	0.1841	-0.1170
1IPP	C37	437	6.135	7.376	6.761	0.0665	0.2161	-0.1294
1IPP	C38	438	6.300	7.571	6.913	0.0512	0.2466	-0.1485
1IPP	C39	439	6.516	7.698	6.755	0.0216	0.2776	-0.1831
1IPP	C40	440	6.804	7.625	6.756	0.0235	0.3165	-0.2056
1IPP	C41	441	6.911	7.859	6.905	0.0066	0.3416	-0.2128
1IPP	C42	442	6.828	8.092	6.741	-0.0474	0.3356	-0.2124
1IPP	C43	443	7.003	8.145	6.508	-0.0763	0.3393	-0.2375
1IPP	C44	444	7.219	8.347	6.520	-0.1090	0.3873	-0.2708
1IPP	C45	445	7.109	8.622	6.499	-0.1591	0.3594	-0.2541
1IPP	C46	446	7.030	8.731	6.234	-0.1956	0.3521	-0.2524
1IPP	C47	447	7.236	8.840	6.049	-0.2338	0.3625	-0.2778
1IPP	C48	448	7.312	9.098	6.174	-0.2508	0.3975	-0.2829
1IPP	C49	449	7.077	9.277	6.197	-0.2806	0.3599	-0.2786
1IPP	C50	450	6.875	9.180	6.394	-0.2657	0.3185	-0.2330
1IPP	C51	451	6.669	8.983	6.308	-0.2285	0.2949	-0.2177
1IPP	C52	452	6.427	9.088	6.172	-0.2557	0.2465	-0.1948
1IPP	C53	453	6.151	9.099	6.287	-0.2527	0.2089	-0.1685
1IPP	C54	454	5.978	8.855	6.281	-0.2166	0.1710	-0.1646
1IPP	C55	455	5.867	8.737	6.030	-0.2100	0.1597	-0.1204
1IPP	C56	456	5.594	8.778	5.919	-0.2435	0.1139	-0.1042
1IPP	C57	457	5.382	8.589	6.009	-0.2062	0.0852	-0.0852
1IPP	C58	458	5.363	8.323	5.877	-0.1725	0.0775	-0.0768
1IPP	C59	459	5.153	8.246	5.683	-0.1788	0.0406	-0.0578
1IPP	C60	460	4.932	8.081	5.794	-0.1387	0.0093	-0.0224
1IPP	C61	461	5.049	7.824	5.888	-0.0916	0.0191	-0.0401
1IPP	C62	462	5.106	7.766	6.174	-0.0566	0.0440	-0.0252
1IPP	C63	463	5.361	7.844	6.309	-0.0485	0.0921	-0.0729
1IPP	C64	464	5.399	8.057	6.515	-0.0674	0.1068	-0.0701
1IPP	C65	465	5.427	8.353	6.485	-0.1113	0.0948	-0.0771
1IPP	C66	466	5.657	8.499	6.362	-0.1523	0.1339	-0.1105
1IPP	C67	467	5.936	8.415	6.426	-0.1196	0.1769	-0.1359
1IPP	C68	468	6.120	8.552	6.615	-0.1304	0.2022	-0.1596
1IPP	C69	469	6.367	8.685	6.514	-0.1666	0.2442	-0.1819
1IPP	C70	470	6.617	8.523	6.495	-0.1301	0.2905	-0.2000
1IPP	C71	471	6.697	8.365	6.257	-0.1437	0.2957	-0.2054
1IPP	C72	472	6.601	8.085	6.219	-0.0954	0.2702	-0.1930
1IPP	C73	473	6.696	7.841	6.361	-0.0428	0.2957	-0.2074
1IPP	C74	474	6.904	7.648	6.272	-0.0237	0.3228	-0.2084
1IPP	C75	475	6.849	7.386	6.140	0.0008	0.3235	-0.2068
1IPP	C76	476	6.932	7.138	6.280	0.0527	0.3235	-0.2129
1IPP	C77	477	7.228	7.159	6.266	0.0478	0.3663	-0.2320
1IPP	C78	478	7.317	7.237	5.994	0.0119	0.3856	-0.2392
1IPP	C79	479	7.287	7.526	5.928	-0.0332	0.3716	-0.2514
1IPP	C80	480	7.033	7.652	5.836	-0.0795	0.3382	-0.2328
1IPP	C81	481	6.894	7.594	5.580	-0.0913	0.3054	-0.2185
1IPP	C82	482	7.015	7.688	5.326	-0.1330	0.3203	-0.2273
1IPP	C83	483	6.928	7.934	5.185	-0.1799	0.3006	-0.2229
1IPP	C84	484	6.751	7.935	4.947	-0.2118	0.2701	-0.2088
1IPP	C85	485	6.898	7.951	4.690	-0.2310	0.2933	-0.2220
1IPP	C86	486	6.988	8.222	4.611	-0.2800	0.3066	-0.2335
1IPP	C87	487	6.733	8.370	4.599	-0.3103	0.2576	-0.2183
1IPP	C88	488	6.504	8.216	4.490	-0.2922	0.2262	-0.1726
1IPP	C89	489	6.362	8.031	4.673	-0.2420	0.2070	-0.1668
1IPP	C90	490	6.213	8.151	4.899	-0.2307	0.1812	-0.1578
1IPP	C91	491	6.389	8.243	5.120	-0.2418	0.2188	-0.1771
1IPP	C92	492	6.503	8.085	5.344	-0.1924	0.2453	-0.1823
1IPP	C93	493	6.323	7.978	5.556	-0.1455	0.2220	-0.1703
1IPP	C94	494	6.409	7.945	5.837	-0.1068	0.2272	-0.1752
1IPP	C95	495	6.575	7.714	5.922	-0.0738	0.2714	-0.1813
1IPP	C96	496	6.420	7.471	5.997	-0.0250	0.2423	-0.1506
1IPP	C97	497	6.348	7.456	6.284	0.0072	0.2384	-0.1562
1IPP	C98	498	6.543	7.343	6.478	0.0455	0.2605	-0.1645
1IPP	C99	499	6.550	7.055	6.553	0.0930	0.2859	-0.1736
1IPP	C100	500	6.371	6.974	6.777	0.1329	0.2556	-0.1469
1IPP	C1	501	6.208	6.521	6.703	0.1862	0.2250	-0.1255
1IPP	C2	502	6.325	6.647	6.460	0.1448	0.2448	-0.1362
1IPP	C3	503	6.188	6.645	6.197	0.1316	0.2101	-0.1105
1IPP	C4	504	6.288	6.449	5.997	0.1228	0.2199	-0.1283
1IPP	C5	505	6.521	6.524	5.827	0.1065	0.2560	-0.1445
1IPP	C6	506	6.368	6.640	5.605	0.0667	0.2244	-0.1325

1IPP	C7	507	6.190	6.438	5.482	0.0849	0.2009	-0.1069
1IPP	C8	508	6.310	6.281	5.261	0.0864	0.1989	-0.1232
1IPP	C9	509	6.183	6.328	4.999	0.0569	0.1828	-0.1156
1IPP	C10	510	5.896	6.270	5.029	0.0621	0.1388	-0.0803
1IPP	C11	511	5.701	6.491	5.017	0.0277	0.1098	-0.0572
1IPP	C12	512	5.678	6.664	5.256	0.0264	0.1172	-0.0719
1IPP	C13	513	5.512	6.619	5.497	0.0548	0.0921	-0.0473
1IPP	C14	514	5.658	6.682	5.748	0.0731	0.1231	-0.0700
1IPP	C15	515	5.867	6.504	5.859	0.1128	0.1502	-0.0778
1IPP	C16	516	5.798	6.261	6.014	0.1672	0.1434	-0.0714
1IPP	C17	517	5.981	6.028	6.003	0.2021	0.1762	-0.0781
1IPP	C18	518	6.185	6.040	6.216	0.2146	0.2152	-0.0972
1IPP	C19	519	6.049	5.982	6.473	0.2552	0.2018	-0.0913
1IPP	C20	520	5.803	6.146	6.457	0.2327	0.1515	-0.0663
1IPP	C21	521	5.823	6.441	6.472	0.1841	0.1632	-0.0705
1IPP	C22	522	5.739	6.537	6.740	0.1868	0.1512	-0.0771
1IPP	C23	523	5.446	6.493	6.749	0.2006	0.1038	-0.0436
1IPP	C24	524	5.307	6.713	6.610	0.1525	0.0817	-0.0310
1IPP	C25	525	5.264	6.610	6.335	0.1385	0.0707	-0.0273
1IPP	C26	526	5.518	6.596	6.183	0.1348	0.1101	-0.0595
1IPP	C27	527	5.662	6.853	6.187	0.0916	0.1364	-0.0671
1IPP	C28	528	5.551	7.087	6.044	0.0431	0.1084	-0.0607
1IPP	C29	529	5.654	7.188	5.785	-0.0053	0.1179	-0.0805
1IPP	C30	530	5.896	7.354	5.730	-0.0230	0.1538	-0.0987
1IPP	C31	531	5.959	7.633	5.815	-0.0655	0.1681	-0.1178
1IPP	C32	532	5.780	7.848	5.716	-0.1026	0.1449	-0.1056
1IPP	C33	533	5.518	7.843	5.857	-0.0970	0.0969	-0.0705
1IPP	C34	534	5.441	7.570	5.947	-0.0456	0.0900	-0.0725
1IPP	C35	535	5.238	7.372	5.852	-0.0154	0.0552	-0.0412
1IPP	C36	536	4.950	7.390	5.924	-0.0192	0.0258	-0.0109
1IPP	C37	537	4.827	7.388	6.196	0.0143	-0.0062	0.0046
1IPP	C38	538	4.685	7.156	6.315	0.0586	-0.0215	0.0285
1IPP	C39	539	4.450	7.061	6.160	0.0464	-0.0580	0.0393
1IPP	C40	540	4.581	6.843	6.008	0.0745	-0.0507	0.0479
1IPP	C41	541	4.740	6.944	5.779	0.0347	-0.0185	0.0177
1IPP	C42	542	4.655	6.849	5.512	0.0209	-0.0440	0.0200
1IPP	C43	543	4.748	6.568	5.483	0.0626	-0.0240	0.0258
1IPP	C44	544	5.034	6.488	5.456	0.0763	0.0159	-0.0012
1IPP	C45	545	5.189	6.292	5.617	0.1187	0.0391	-0.0055
1IPP	C46	546	5.328	6.357	5.870	0.1362	0.0743	-0.0218
1IPP	C47	547	5.299	6.186	6.111	0.1840	0.0796	-0.0211
1IPP	C48	548	5.083	6.152	6.311	0.2094	0.0511	0.0058
1IPP	C49	549	5.066	6.279	6.579	0.2264	0.0465	-0.0011
1IPP	C50	550	4.843	6.473	6.603	0.1908	0.0130	0.0175
1IPP	C51	551	4.859	6.748	6.493	0.1337	0.0095	0.0110
1IPP	C52	552	4.921	6.962	6.687	0.1286	0.0273	-0.0048
1IPP	C53	553	4.747	7.172	6.806	0.1032	0.0118	0.0212
1IPP	C54	554	4.749	7.435	6.667	0.0448	-0.0013	0.0029
1IPP	C55	555	5.010	7.552	6.583	0.0187	0.0380	-0.0263
1IPP	C56	556	5.218	7.645	6.774	0.0277	0.0820	-0.0418
1IPP	C57	557	5.426	7.433	6.781	0.0759	0.1057	-0.0550
1IPP	C58	558	5.294	7.167	6.792	0.1021	0.0842	-0.0358
1IPP	C59	559	5.250	7.017	7.045	0.1520	0.0803	-0.0284
1IPP	C60	560	5.449	6.799	7.075	0.1873	0.1173	-0.0494
1IPP	C61	561	5.724	6.906	7.105	0.1750	0.1669	-0.0832
1IPP	C62	562	5.752	7.120	6.901	0.1222	0.1544	-0.0850
1IPP	C63	563	5.703	7.016	6.627	0.0971	0.1500	-0.0686
1IPP	C64	564	5.951	6.888	6.527	0.1221	0.1849	-0.0990
1IPP	C65	565	6.125	7.085	6.391	0.0829	0.2028	-0.1151
1IPP	C66	566	5.971	7.185	6.160	0.0323	0.1800	-0.1244
1IPP	C67	567	5.806	7.428	6.178	0.0011	0.1486	-0.1040
1IPP	C68	568	5.578	7.421	6.367	0.0204	0.1223	-0.0802
1IPP	C69	569	5.294	7.352	6.316	0.0204	0.0721	-0.0344
1IPP	C70	570	5.185	7.078	6.344	0.0689	0.0624	-0.0306
1IPP	C71	571	5.027	6.954	6.126	0.0649	0.0227	-0.0121
1IPP	C72	572	5.104	6.735	5.943	0.0841	0.0384	-0.0108
1IPP	C73	573	5.271	6.890	5.755	0.0451	0.0581	-0.0280
1IPP	C74	574	5.139	7.054	5.549	-0.0119	0.0356	-0.0191
1IPP	C75	575	5.056	6.894	5.317	-0.0013	0.0123	-0.0095
1IPP	C76	576	5.253	6.753	5.147	0.0070	0.0360	-0.0327
1IPP	C77	577	5.393	6.871	4.915	-0.0429	0.0576	-0.0390
1IPP	C78	578	5.652	7.015	4.937	-0.0600	0.0952	-0.0689
1IPP	C79	579	5.909	6.878	4.882	-0.0480	0.1353	-0.1012

1IPP	C80	580	6.080	6.738	5.083	-0.0031	0.1761	-0.1062
1IPP	C81	581	6.330	6.837	5.205	-0.0164	0.2065	-0.1369
1IPP	C82	582	6.615	6.751	5.177	0.0041	0.2582	-0.1707
1IPP	C83	583	6.761	6.610	5.395	0.0535	0.2784	-0.1782
1IPP	C84	584	6.760	6.314	5.434	0.0974	0.2893	-0.1650
1IPP	C85	585	6.574	6.139	5.589	0.1447	0.2546	-0.1312
1IPP	C86	586	6.564	6.094	5.884	0.1730	0.2633	-0.1508
1IPP	C87	587	6.730	5.917	6.056	0.2189	0.2877	-0.1587
1IPP	C88	588	6.613	5.674	6.181	0.2742	0.2809	-0.1378
1IPP	C89	589	6.439	5.721	6.416	0.2906	0.2552	-0.1275
1IPP	C90	590	6.544	5.903	6.626	0.2895	0.2781	-0.1351
1IPP	C91	591	6.468	6.182	6.562	0.2264	0.2656	-0.1283
1IPP	C92	592	6.552	6.279	6.295	0.1923	0.2773	-0.1568
1IPP	C93	593	6.761	6.473	6.212	0.1539	0.3071	-0.1788
1IPP	C94	594	6.692	6.763	6.215	0.1173	0.2957	-0.1645
1IPP	C95	595	6.586	6.928	5.991	0.0512	0.2722	-0.1632
1IPP	C96	596	6.310	7.029	5.953	0.0450	0.2239	-0.1426
1IPP	C97	597	6.071	6.903	5.825	0.0495	0.1846	-0.1179
1IPP	C98	598	6.008	6.884	5.534	0.0152	0.1666	-0.0991
1IPP	C99	599	5.863	7.082	5.366	-0.0286	0.1419	-0.1000
1IPP	C100	600	5.565	7.070	5.375	-0.0232	0.1067	-0.0691
1IPP	C1	601	2.929	5.303	7.451	0.4556	-0.2705	0.2421
1IPP	C2	602	3.018	5.588	7.451	0.4089	-0.2559	0.2260
1IPP	C3	603	2.837	5.799	7.344	0.3694	-0.2841	0.2360
1IPP	C4	604	2.823	5.881	7.058	0.3299	-0.2999	0.2370
1IPP	C5	605	2.966	6.109	6.930	0.2874	-0.2851	0.2169
1IPP	C6	606	2.837	6.374	6.961	0.2467	-0.2877	0.2248
1IPP	C7	607	2.566	6.417	6.845	0.2114	-0.3420	0.2484
1IPP	C8	608	2.529	6.516	6.567	0.1857	-0.3463	0.2539
1IPP	C9	609	2.591	6.806	6.539	0.1396	-0.3372	0.2381
1IPP	C10	610	2.858	6.869	6.427	0.1095	-0.2983	0.2194
1IPP	C11	611	3.101	6.792	6.576	0.1406	-0.2665	0.1886
1IPP	C12	612	3.201	7.012	6.750	0.1249	-0.2345	0.1814
1IPP	C13	613	3.335	7.224	6.590	0.0753	-0.2198	0.1570
1IPP	C14	614	3.604	7.124	6.514	0.0784	-0.1902	0.1369
1IPP	C15	615	3.556	6.856	6.396	0.1140	-0.1900	0.1281
1IPP	C16	616	3.530	6.669	6.624	0.1564	-0.1998	0.1491
1IPP	C17	617	3.803	6.606	6.718	0.1901	-0.1474	0.1314
1IPP	C18	618	3.913	6.515	6.458	0.1785	-0.1408	0.1086
1IPP	C19	619	3.694	6.354	6.343	0.1838	-0.1809	0.1376
1IPP	C20	620	3.609	6.123	6.508	0.2406	-0.1833	0.1518
1IPP	C21	621	3.358	6.172	6.659	0.2371	-0.2171	0.1695
1IPP	C22	622	3.374	6.335	6.907	0.2406	-0.2041	0.1674
1IPP	C23	623	3.465	6.197	7.155	0.3021	-0.1861	0.1657
1IPP	C24	624	3.257	6.013	7.262	0.3281	-0.2339	0.1925
1IPP	C25	625	3.282	5.756	7.117	0.3537	-0.2247	0.1813
1IPP	C26	626	3.263	5.757	6.822	0.3209	-0.2339	0.2055
1IPP	C27	627	3.014	5.761	6.663	0.3131	-0.2698	0.2264
1IPP	C28	628	2.923	5.537	6.492	0.3302	-0.2920	0.2403
1IPP	C29	629	3.009	5.508	6.209	0.3033	-0.2903	0.2196
1IPP	C30	630	2.888	5.678	5.999	0.2621	-0.3087	0.2401
1IPP	C31	631	2.650	5.600	5.838	0.2478	-0.3421	0.2580
1IPP	C32	632	2.672	5.359	5.665	0.2719	-0.3449	0.2594
1IPP	C33	633	2.809	5.329	5.402	0.2362	-0.3343	0.2513
1IPP	C34	634	2.685	5.358	5.132	0.2101	-0.3636	0.2715
1IPP	C35	635	2.841	5.532	4.947	0.1697	-0.3257	0.2343
1IPP	C36	636	3.113	5.414	4.956	0.1996	-0.2972	0.2149
1IPP	C37	637	3.228	5.491	5.217	0.2071	-0.2729	0.2073
1IPP	C38	638	3.321	5.772	5.223	0.1635	-0.2513	0.1884
1IPP	C39	639	3.606	5.851	5.217	0.1407	-0.2152	0.1614
1IPP	C40	640	3.685	5.838	5.504	0.1858	-0.1959	0.1471
1IPP	C41	641	3.481	5.954	5.681	0.1784	-0.2230	0.1717
1IPP	C42	642	3.338	6.136	5.498	0.1199	-0.2458	0.1791
1IPP	C43	643	3.394	6.426	5.501	0.0883	-0.2357	0.1751
1IPP	C44	644	3.249	6.573	5.711	0.0916	-0.2647	0.1766
1IPP	C45	645	2.989	6.700	5.653	0.0656	-0.3019	0.2073
1IPP	C46	646	2.992	6.975	5.544	-0.0026	-0.2987	0.1926
1IPP	C47	647	2.874	7.155	5.748	0.0011	-0.3209	0.1990
1IPP	C48	648	3.004	7.368	5.911	-0.0182	-0.2794	0.1813
1IPP	C49	649	3.102	7.285	6.178	0.0202	-0.2792	0.1808
1IPP	C50	650	3.375	7.184	6.125	0.0262	-0.2382	0.1358
1IPP	C51	651	3.292	6.969	5.939	0.0441	-0.2375	0.1779
1IPP	C52	652	3.148	6.760	6.093	0.0965	-0.2615	0.1745

1IPP	C53	653	3.303	6.529	6.197	0.1434	-0.2346	0.1730
1IPP	C54	654	3.372	6.292	6.031	0.1639	-0.2254	0.1722
1IPP	C55	655	3.196	6.052	6.025	0.1932	-0.2559	0.1937
1IPP	C56	656	3.184	5.921	6.290	0.2550	-0.2609	0.2022
1IPP	C57	657	3.399	5.745	6.395	0.2833	-0.2304	0.1807
1IPP	C58	658	3.410	5.448	6.401	0.3329	-0.2059	0.1971
1IPP	C59	659	3.319	5.288	6.633	0.3896	-0.2215	0.2016
1IPP	C60	660	3.049	5.171	6.686	0.3930	-0.2784	0.2293
1IPP	C61	661	2.953	4.892	6.687	0.4472	-0.2666	0.2505
1IPP	C62	662	2.813	4.797	6.445	0.4290	-0.3094	0.2666
1IPP	C63	663	2.519	4.810	6.410	0.4323	-0.3503	0.2863
1IPP	C64	664	2.379	4.558	6.340	0.4606	-0.3814	0.3105
1IPP	C65	665	2.443	4.505	6.055	0.4518	-0.3755	0.3131
1IPP	C66	666	2.300	4.642	5.831	0.3978	-0.4052	0.3125
1IPP	C67	667	2.388	4.908	5.727	0.3517	-0.3881	0.3069
1IPP	C68	668	2.607	4.940	5.529	0.3160	-0.3678	0.2834
1IPP	C69	669	2.892	4.920	5.608	0.3354	-0.3129	0.2514
1IPP	C70	670	3.058	5.112	5.762	0.3266	-0.2898	0.2325
1IPP	C71	671	3.228	5.325	5.641	0.2741	-0.2534	0.2021
1IPP	C72	672	3.201	5.619	5.669	0.2214	-0.2728	0.2015
1IPP	C73	673	2.964	5.773	5.576	0.1929	-0.3044	0.2288
1IPP	C74	674	2.849	5.781	5.304	0.1710	-0.3316	0.2372
1IPP	C75	675	2.931	5.977	5.099	0.1188	-0.3274	0.2267
1IPP	C76	676	2.794	6.230	5.167	0.0802	-0.3410	0.2369
1IPP	C77	677	2.925	6.346	5.407	0.0909	-0.3159	0.2130
1IPP	C78	678	2.957	6.216	5.673	0.1405	-0.2992	0.2095
1IPP	C79	679	2.774	6.116	5.885	0.1721	-0.3293	0.2451
1IPP	C80	680	2.654	6.302	6.081	0.1551	-0.3352	0.2496
1IPP	C81	681	2.842	6.461	6.244	0.1504	-0.3068	0.2245
1IPP	C82	682	2.971	6.290	6.447	0.2030	-0.2870	0.2173
1IPP	C83	683	2.747	6.117	6.536	0.2417	-0.3144	0.2485
1IPP	C84	684	2.667	5.883	6.371	0.2532	-0.3414	0.2524
1IPP	C85	685	2.470	5.872	6.150	0.2405	-0.3713	0.2728
1IPP	C86	686	2.180	5.929	6.175	0.2327	-0.4131	0.3050
1IPP	C87	687	2.019	5.778	6.371	0.2803	-0.4308	0.3296
1IPP	C88	688	2.024	5.989	6.579	0.2602	-0.4337	0.3078
1IPP	C89	689	2.301	6.075	6.645	0.2547	-0.3917	0.2850
1IPP	C90	690	2.420	5.895	6.850	0.3060	-0.3530	0.2762
1IPP	C91	691	2.562	5.658	6.740	0.3341	-0.3379	0.2717
1IPP	C92	692	2.390	5.546	6.526	0.3217	-0.3757	0.2903
1IPP	C93	693	2.157	5.378	6.603	0.3636	-0.4042	0.3195
1IPP	C94	694	2.167	5.083	6.571	0.4056	-0.4086	0.3195
1IPP	C95	695	2.069	5.010	6.300	0.3922	-0.4340	0.3347
1IPP	C96	696	2.283	5.091	6.112	0.3482	-0.3949	0.3142
1IPP	C97	697	2.341	5.381	6.081	0.3032	-0.3917	0.2912
1IPP	C98	698	2.192	5.565	5.902	0.2670	-0.4156	0.3067
1IPP	C99	699	2.213	5.556	5.606	0.2348	-0.4232	0.3159
1IPP	C100	700	2.424	5.648	5.418	0.1989	-0.3885	0.2859
1IPP	C1	701	6.755	6.722	4.360	-0.0683	0.2584	-0.1801
1IPP	C2	702	7.017	6.762	4.495	-0.0665	0.3011	-0.2032
1IPP	C3	703	7.125	6.615	4.730	-0.0213	0.3258	-0.2124
1IPP	C4	704	7.061	6.752	4.985	-0.0151	0.3270	-0.2098
1IPP	C5	705	7.239	6.989	4.988	-0.0457	0.3500	-0.2378
1IPP	C6	706	7.531	6.960	5.027	-0.0478	0.3970	-0.2750
1IPP	C7	707	7.650	6.897	5.292	-0.0043	0.4136	-0.2743
1IPP	C8	708	7.625	7.098	5.508	-0.0187	0.4182	-0.2647
1IPP	C9	709	7.379	6.975	5.615	0.0177	0.3852	-0.2576
1IPP	C10	710	7.154	6.944	5.425	0.0035	0.3378	-0.2154
1IPP	C11	711	6.994	7.189	5.455	-0.0326	0.3365	-0.2147
1IPP	C12	712	6.884	7.164	5.730	-0.0043	0.3161	-0.1958
1IPP	C13	713	7.016	6.938	5.870	0.0480	0.3355	-0.2120
1IPP	C14	714	7.016	6.658	5.770	0.0833	0.3315	-0.1954
1IPP	C15	715	7.249	6.523	5.636	0.0944	0.3666	-0.2234
1IPP	C16	716	7.302	6.521	5.343	0.0574	0.3703	-0.2272
1IPP	C17	717	7.120	6.356	5.173	0.0596	0.3479	-0.2091
1IPP	C18	718	7.154	6.067	5.114	0.1093	0.3305	-0.2125
1IPP	C19	719	7.269	5.972	4.856	0.0980	0.3502	-0.2054
1IPP	C20	720	7.137	5.799	4.653	0.0923	0.3332	-0.1938
1IPP	C21	721	7.205	5.510	4.656	0.1396	0.3398	-0.1929
1IPP	C22	722	7.045	5.303	4.795	0.1979	0.3070	-0.1779
1IPP	C23	723	7.162	5.199	5.047	0.2328	0.3319	-0.1950
1IPP	C24	724	7.360	4.981	5.011	0.2545	0.3609	-0.1949
1IPP	C25	725	7.632	5.096	5.000	0.2531	0.4045	-0.2317

1IPP	C26	726	7.617	5.373	4.891	0.1985	0.4079	-0.2401
1IPP	C27	727	7.669	5.473	4.616	0.1528	0.4080	-0.2389
1IPP	C28	728	7.943	5.568	4.550	0.1251	0.4500	-0.2751
1IPP	C29	729	8.046	5.826	4.658	0.0863	0.4655	-0.2892
1IPP	C30	730	8.022	6.093	4.532	0.0537	0.4558	-0.2895
1IPP	C31	731	8.233	6.247	4.392	0.0080	0.4908	-0.3272
1IPP	C32	732	8.334	6.479	4.549	-0.0222	0.5144	-0.3336
1IPP	C33	733	8.594	6.503	4.694	-0.0085	0.5564	-0.3573
1IPP	C34	734	8.642	6.434	4.980	0.0342	0.5700	-0.3584
1IPP	C35	735	8.814	6.200	5.048	0.0832	0.5984	-0.3801
1IPP	C36	736	8.707	5.923	5.077	0.1177	0.5831	-0.3512
1IPP	C37	737	8.578	5.906	5.343	0.1511	0.5576	-0.3458
1IPP	C38	738	8.305	6.018	5.376	0.1484	0.5129	-0.3190
1IPP	C39	739	8.272	6.313	5.384	0.0967	0.5233	-0.3178
1IPP	C40	740	8.325	6.479	5.625	0.0989	0.5291	-0.3356
1IPP	C41	741	8.129	6.523	5.845	0.1119	0.5005	-0.3113
1IPP	C42	742	7.950	6.759	5.815	0.0695	0.4838	-0.2937
1IPP	C43	743	7.677	6.674	5.737	0.0817	0.4271	-0.2781
1IPP	C44	744	7.723	6.446	5.555	0.0917	0.4460	-0.2677
1IPP	C45	745	7.869	6.484	5.300	0.0550	0.4581	-0.2882
1IPP	C46	746	7.711	6.531	5.055	0.0199	0.4285	-0.2753
1IPP	C47	747	7.569	6.281	4.985	0.0517	0.4061	-0.2462
1IPP	C48	748	7.705	6.046	4.862	0.0843	0.4172	-0.2588
1IPP	C49	749	7.772	5.794	5.007	0.1422	0.4319	-0.2538
1IPP	C50	750	8.039	5.763	5.136	0.1615	0.4757	-0.2986
1IPP	C51	751	8.296	5.700	5.002	0.1525	0.5058	-0.3087
1IPP	C52	752	8.406	5.433	4.934	0.1810	0.5345	-0.3191
1IPP	C53	753	8.302	5.308	4.687	0.1831	0.5071	-0.2813
1IPP	C54	754	8.047	5.181	4.766	0.2146	0.4774	-0.2708
1IPP	C55	755	8.096	4.959	4.956	0.2688	0.4766	-0.2776
1IPP	C56	756	8.152	4.697	4.830	0.2868	0.4884	-0.2746
1IPP	C57	757	7.889	4.593	4.740	0.2982	0.4338	-0.2413
1IPP	C58	758	7.784	4.736	4.502	0.2576	0.4249	-0.2337
1IPP	C59	759	7.677	5.011	4.544	0.2036	0.4154	-0.2372
1IPP	C60	760	7.393	5.092	4.583	0.2089	0.3637	-0.1974
1IPP	C61	761	7.241	5.161	4.338	0.1610	0.3337	-0.1959
1IPP	C62	762	7.373	5.382	4.189	0.1255	0.3522	-0.2142
1IPP	C63	763	7.335	5.670	4.254	0.0774	0.3456	-0.2119
1IPP	C64	764	7.519	5.839	4.415	0.0757	0.3875	-0.2332
1IPP	C65	765	7.672	6.046	4.270	0.0133	0.3972	-0.2504
1IPP	C66	766	7.880	5.948	4.082	0.0267	0.4271	-0.2831
1IPP	C67	767	8.095	5.762	4.172	0.0574	0.4603	-0.2900
1IPP	C68	768	8.371	5.821	4.266	0.0563	0.5079	-0.3198
1IPP	C69	769	8.474	5.806	4.545	0.0887	0.5239	-0.3287
1IPP	C70	770	8.493	6.053	4.709	0.0692	0.5500	-0.3353
1IPP	C71	771	8.315	6.126	4.935	0.0870	0.5091	-0.3225
1IPP	C72	772	8.087	6.313	4.933	0.0385	0.4840	-0.3025
1IPP	C73	773	8.199	6.586	4.962	0.0101	0.4931	-0.3236
1IPP	C74	774	8.295	6.723	5.210	0.0086	0.5190	-0.3305
1IPP	C75	775	8.100	6.869	5.381	0.0059	0.4990	-0.3177
1IPP	C76	776	8.031	7.153	5.324	-0.0346	0.4814	-0.3082
1IPP	C77	777	8.081	7.390	5.496	-0.0654	0.4914	-0.3361
1IPP	C78	778	7.868	7.440	5.699	-0.0436	0.4652	-0.3025
1IPP	C79	779	7.633	7.598	5.611	-0.0830	0.4218	-0.2929
1IPP	C80	780	7.379	7.485	5.503	-0.0775	0.3815	-0.2623
1IPP	C81	781	7.347	7.349	5.241	-0.0809	0.3725	-0.2421
1IPP	C82	782	7.312	7.474	4.974	-0.1134	0.3554	-0.2527
1IPP	C83	783	7.047	7.578	4.891	-0.1658	0.3211	-0.2289
1IPP	C84	784	6.818	7.425	5.001	-0.1204	0.2926	-0.2065
1IPP	C85	785	6.821	7.134	4.944	-0.0784	0.2771	-0.1918
1IPP	C86	786	6.696	6.984	4.722	-0.0762	0.2617	-0.1781
1IPP	C87	787	6.474	6.785	4.741	-0.0449	0.2301	-0.1564
1IPP	C88	788	6.543	6.505	4.811	0.0132	0.2363	-0.1513
1IPP	C89	789	6.754	6.376	4.650	0.0083	0.2790	-0.1674
1IPP	C90	790	6.716	6.263	4.377	-0.0035	0.2503	-0.1594
1IPP	C91	791	6.726	5.968	4.395	0.0482	0.2526	-0.1587
1IPP	C92	792	6.488	5.797	4.350	0.0714	0.2146	-0.1328
1IPP	C93	793	6.432	5.656	4.097	0.0699	0.2015	-0.1206
1IPP	C94	794	6.350	5.803	3.855	0.0147	0.1854	-0.1198
1IPP	C95	795	6.604	5.898	3.737	-0.0073	0.2253	-0.1416
1IPP	C96	796	6.725	6.110	3.906	-0.0157	0.2511	-0.1615
1IPP	C97	797	7.011	6.141	3.985	-0.0203	0.2833	-0.1917
1IPP	C98	798	7.132	6.053	4.241	0.0134	0.3200	-0.1950

1IPP	C99	799	7.159	6.220	4.484	0.0160	0.3250	-0.2056
1IPP	C100	800	7.436	6.294	4.557	0.0119	0.3719	-0.2349
1IPP	C1	801	3.391	8.053	4.977	-0.2214	-0.2421	0.1329
1IPP	C2	802	3.442	7.763	5.025	-0.1696	-0.2501	0.1277
1IPP	C3	803	3.453	7.645	5.298	-0.1174	-0.2354	0.1407
1IPP	C4	804	3.712	7.639	5.443	-0.1124	-0.1964	0.1026
1IPP	C5	805	3.902	7.413	5.408	-0.0770	-0.1689	0.0899
1IPP	C6	806	3.910	7.171	5.583	-0.0184	-0.1526	0.1027
1IPP	C7	807	4.115	7.201	5.794	0.0020	-0.1161	0.0737
1IPP	C8	808	4.376	7.197	5.653	-0.0230	-0.0950	0.0538
1IPP	C9	809	4.424	7.481	5.580	-0.0721	-0.0609	0.0372
1IPP	C10	810	4.329	7.609	5.331	-0.1156	-0.0939	0.0500
1IPP	C11	811	4.527	7.677	5.120	-0.1430	-0.0673	0.0170
1IPP	C12	812	4.659	7.941	5.106	-0.1903	-0.0543	0.0043
1IPP	C13	813	4.441	8.115	5.007	-0.2281	-0.0951	0.0246
1IPP	C14	814	4.200	8.020	5.153	-0.1979	-0.1203	0.0443
1IPP	C15	815	4.010	7.826	5.031	-0.1711	-0.1467	0.0770
1IPP	C16	816	4.047	7.536	4.975	-0.1364	-0.1532	0.0687
1IPP	C17	817	4.208	7.561	4.727	-0.1756	-0.1242	0.0601
1IPP	C18	818	4.113	7.751	4.523	-0.2151	-0.1510	0.0715
1IPP	C19	819	4.239	8.018	4.551	-0.2667	-0.1231	0.0482
1IPP	C20	820	4.084	8.208	4.716	-0.2629	-0.1568	0.0565
1IPP	C21	821	3.879	8.400	4.618	-0.3084	-0.1897	0.0703
1IPP	C22	822	3.595	8.380	4.697	-0.3046	-0.2312	0.1040
1IPP	C23	823	3.648	8.472	4.971	-0.2939	-0.2034	0.0922
1IPP	C24	824	3.808	8.242	5.067	-0.2362	-0.1867	0.0898
1IPP	C25	825	3.705	8.051	5.268	-0.1878	-0.2015	0.1014
1IPP	C26	826	3.838	8.081	5.528	-0.1677	-0.1657	0.0854
1IPP	C27	827	4.080	7.922	5.586	-0.1365	-0.1319	0.0555
1IPP	C28	828	4.065	7.674	5.747	-0.0826	-0.1332	0.0672
1IPP	C29	829	4.048	7.765	6.028	-0.0747	-0.1188	0.0716
1IPP	C30	830	3.776	7.863	6.093	-0.0788	-0.1717	0.0927
1IPP	C31	831	3.692	8.099	5.936	-0.1272	-0.1781	0.0995
1IPP	C32	832	3.835	8.359	5.926	-0.1688	-0.1630	0.0820
1IPP	C33	833	4.071	8.390	5.748	-0.1923	-0.1235	0.0547
1IPP	C34	834	4.346	8.375	5.857	-0.1833	-0.0909	0.0302
1IPP	C35	835	4.405	8.650	5.954	-0.2123	-0.0687	0.0158
1IPP	C36	836	4.528	8.848	5.768	-0.2663	-0.0641	0.0008
1IPP	C37	837	4.366	9.014	5.581	-0.3067	-0.0826	0.0143
1IPP	C38	838	4.304	8.880	5.324	-0.3163	-0.0966	0.0183
1IPP	C39	839	4.520	8.818	5.130	-0.3180	-0.0678	-0.0137
1IPP	C40	840	4.595	9.007	4.915	-0.3743	-0.0616	-0.0134
1IPP	C41	841	4.455	8.964	4.657	-0.3910	-0.0912	0.0003
1IPP	C42	842	4.594	8.736	4.529	-0.3643	-0.0699	-0.0082
1IPP	C43	843	4.472	8.477	4.611	-0.3255	-0.0900	0.0120
1IPP	C44	844	4.283	8.453	4.385	-0.3355	-0.1250	0.0387
1IPP	C45	845	4.104	8.691	4.371	-0.3857	-0.1590	0.0510
1IPP	C46	846	4.112	8.906	4.166	-0.4298	-0.1442	0.0367
1IPP	C47	847	4.268	9.132	4.274	-0.4670	-0.1272	0.0156
1IPP	C48	848	4.045	9.265	4.415	-0.4582	-0.1676	0.0242
1IPP	C49	849	3.967	9.081	4.634	-0.4134	-0.1560	0.0484
1IPP	C50	850	3.767	8.861	4.606	-0.3892	-0.2039	0.0734
1IPP	C51	851	3.490	8.935	4.679	-0.3808	-0.2488	0.1002
1IPP	C52	852	3.437	9.022	4.958	-0.3753	-0.2414	0.1068
1IPP	C53	853	3.413	8.805	5.158	-0.3245	-0.2471	0.1097
1IPP	C54	854	3.167	8.642	5.116	-0.2895	-0.2875	0.1394
1IPP	C55	855	3.185	8.443	4.896	-0.2861	-0.2819	0.1464
1IPP	C56	856	3.127	8.504	4.611	-0.3252	-0.3045	0.1467
1IPP	C57	857	2.857	8.435	4.504	-0.3200	-0.3451	0.1695
1IPP	C58	858	2.810	8.147	4.447	-0.2924	-0.3478	0.1832
1IPP	C59	859	2.656	7.992	4.649	-0.2419	-0.3770	0.2140
1IPP	C60	860	2.686	8.086	4.930	-0.2391	-0.3690	0.1926
1IPP	C61	861	2.956	8.073	5.053	-0.2089	-0.3158	0.1749
1IPP	C62	862	3.054	7.852	5.225	-0.1693	-0.3021	0.1744
1IPP	C63	863	3.102	7.887	5.516	-0.1347	-0.2748	0.1596
1IPP	C64	864	3.371	7.977	5.605	-0.1412	-0.2488	0.1277
1IPP	C65	865	3.399	8.272	5.636	-0.1901	-0.2262	0.1241
1IPP	C66	866	3.503	8.442	5.415	-0.2311	-0.2225	0.1124
1IPP	C67	867	3.789	8.522	5.409	-0.2494	-0.1770	0.0715
1IPP	C68	868	3.901	8.778	5.510	-0.2866	-0.1682	0.0587
1IPP	C69	869	3.856	8.988	5.303	-0.3323	-0.1702	0.0593
1IPP	C70	870	3.874	8.859	5.036	-0.3388	-0.1713	0.0658
1IPP	C71	871	4.128	8.759	4.919	-0.3302	-0.1314	0.0413

1IPP	C72	872	4.192	8.493	5.032	-0.2740	-0.1290	0.0394
1IPP	C73	873	4.246	8.445	5.321	-0.2372	-0.1057	0.0283
1IPP	C74	874	4.510	8.486	5.454	-0.2479	-0.0718	0.0034
1IPP	C75	875	4.672	8.238	5.446	-0.1930	-0.0449	0.0015
1IPP	C76	876	4.506	8.006	5.528	-0.1528	-0.0714	0.0199
1IPP	C77	877	4.476	7.934	5.813	-0.1176	-0.0595	0.0256
1IPP	C78	878	4.612	7.701	5.938	-0.0716	-0.0467	0.0100
1IPP	C79	879	4.421	7.490	6.027	-0.0247	-0.0645	0.0391
1IPP	C80	880	4.339	7.535	6.308	-0.0012	-0.0774	0.0437
1IPP	C81	881	4.330	7.337	6.531	0.0510	-0.0699	0.0551
1IPP	C82	882	4.064	7.210	6.565	0.0661	-0.1117	0.0834
1IPP	C83	883	4.023	6.944	6.442	0.1048	-0.1212	0.0834
1IPP	C84	884	4.018	6.931	6.147	0.0693	-0.1265	0.1016
1IPP	C85	885	3.759	6.948	6.004	0.0523	-0.1776	0.1164
1IPP	C86	886	3.658	6.697	5.887	0.0916	-0.1835	0.1379
1IPP	C87	887	3.700	6.747	5.597	0.0391	-0.1927	0.1330
1IPP	C88	888	3.467	6.907	5.504	0.0150	-0.2327	0.1533
1IPP	C89	889	3.467	7.204	5.488	-0.0351	-0.2297	0.1380
1IPP	C90	890	3.442	7.380	5.727	-0.0416	-0.2208	0.1470
1IPP	C91	891	3.687	7.472	5.870	-0.0420	-0.1900	0.1168
1IPP	C92	892	3.814	7.374	6.122	0.0053	-0.1605	0.0902
1IPP	C93	893	3.777	7.519	6.381	0.0069	-0.1535	0.1172
1IPP	C94	894	3.977	7.710	6.496	-0.0146	-0.1172	0.0831
1IPP	C95	895	3.985	8.006	6.460	-0.0577	-0.1349	0.0678
1IPP	C96	896	4.126	8.154	6.241	-0.1123	-0.1104	0.0618
1IPP	C97	897	4.405	8.250	6.286	-0.1081	-0.0651	0.0191
1IPP	C98	898	4.634	8.087	6.194	-0.1054	-0.0221	0.0086
1IPP	C99	899	4.726	7.876	6.381	-0.0484	-0.0105	0.0019
1IPP	C100	900	4.924	7.989	6.573	-0.0560	0.0214	-0.0210
1IPP	C1	901	3.743	4.553	4.610	0.2914	-0.2139	0.1820
1IPP	C2	902	3.565	4.786	4.551	0.2382	-0.2273	0.1902
1IPP	C3	903	3.646	5.065	4.483	0.1969	-0.2164	0.1673
1IPP	C4	904	3.731	5.257	4.694	0.1930	-0.2060	0.1615
1IPP	C5	905	3.562	5.425	4.870	0.1787	-0.2397	0.1686
1IPP	C6	906	3.516	5.698	4.772	0.1160	-0.2375	0.1674
1IPP	C7	907	3.789	5.789	4.708	0.1134	-0.1966	0.1444
1IPP	C8	908	3.963	5.937	4.897	0.1011	-0.1702	0.1195
1IPP	C9	909	3.982	6.233	4.867	0.0587	-0.1564	0.1192
1IPP	C10	910	4.194	6.328	4.682	0.0192	-0.1300	0.0825
1IPP	C11	911	4.432	6.378	4.851	0.0360	-0.0965	0.0661
1IPP	C12	912	4.578	6.121	4.881	0.0674	-0.0600	0.0522
1IPP	C13	913	4.439	5.867	4.945	0.1253	-0.0848	0.0732
1IPP	C14	914	4.376	5.811	5.230	0.1555	-0.0868	0.0902
1IPP	C15	915	4.096	5.844	5.321	0.1567	-0.1371	0.1156
1IPP	C16	916	3.971	6.113	5.301	0.1187	-0.1583	0.1181
1IPP	C17	917	3.962	6.339	5.492	0.1039	-0.1513	0.1086
1IPP	C18	918	3.765	6.304	5.710	0.1205	-0.1840	0.1322
1IPP	C19	919	3.832	6.177	5.969	0.1716	-0.1674	0.1305
1IPP	C20	920	3.675	5.958	6.092	0.2292	-0.1885	0.1450
1IPP	C21	921	3.725	5.667	6.092	0.2561	-0.1675	0.1522
1IPP	C22	922	3.627	5.483	5.883	0.2664	-0.1909	0.1625
1IPP	C23	923	3.843	5.319	5.764	0.2916	-0.1648	0.1498
1IPP	C24	924	3.915	5.460	5.515	0.2438	-0.1535	0.1325
1IPP	C25	925	3.705	5.420	5.309	0.2178	-0.1919	0.1586
1IPP	C26	926	3.863	5.240	5.138	0.2397	-0.1813	0.1433
1IPP	C27	927	4.098	5.388	5.039	0.2024	-0.1444	0.1307
1IPP	C28	928	4.144	5.504	4.769	0.1592	-0.1327	0.1201
1IPP	C29	929	4.199	5.284	4.577	0.1730	-0.1279	0.0971
1IPP	C30	930	4.430	5.102	4.630	0.2056	-0.1047	0.0899
1IPP	C31	931	4.696	5.122	4.499	0.1899	-0.0568	0.0648
1IPP	C32	932	4.773	4.948	4.272	0.1865	-0.0537	0.0611
1IPP	C33	933	4.842	4.674	4.368	0.2542	-0.0378	0.0622
1IPP	C34	934	5.061	4.649	4.568	0.2719	-0.0103	0.0353
1IPP	C35	935	4.949	4.685	4.842	0.2912	-0.0174	0.0491
1IPP	C36	936	4.847	4.960	4.886	0.2600	-0.0224	0.0531
1IPP	C37	937	5.002	5.195	4.983	0.2296	0.0026	0.0304
1IPP	C38	938	5.040	5.265	5.269	0.2405	0.0051	0.0208
1IPP	C39	939	4.829	5.452	5.361	0.2264	-0.0169	0.0510
1IPP	C40	940	4.799	5.686	5.181	0.1770	-0.0290	0.0420
1IPP	C41	941	4.920	5.956	5.186	0.1324	-0.0147	0.0196
1IPP	C42	942	4.813	6.110	5.415	0.1259	-0.0175	0.0363
1IPP	C43	943	4.526	6.170	5.456	0.1253	-0.0768	0.0666
1IPP	C44	944	4.362	6.338	5.273	0.0746	-0.0927	0.0762

1IPP	C45	945	4.337	6.634	5.269	0.0325	-0.0926	0.0640
1IPP	C46	946	4.145	6.760	5.457	0.0348	-0.1325	0.0738
1IPP	C47	947	4.214	6.751	5.747	0.0669	-0.1093	0.0869
1IPP	C48	948	4.130	6.526	5.924	0.1171	-0.1205	0.0809
1IPP	C49	949	4.284	6.272	5.915	0.1508	-0.0972	0.0753
1IPP	C50	950	4.200	6.055	5.728	0.1694	-0.1087	0.1054
1IPP	C51	951	4.038	5.811	5.777	0.2191	-0.1296	0.1136
1IPP	C52	952	4.236	5.599	5.840	0.2450	-0.0949	0.1023
1IPP	C53	953	4.432	5.572	5.620	0.2320	-0.0704	0.0800
1IPP	C54	954	4.352	5.336	5.463	0.2485	-0.0929	0.0864
1IPP	C55	955	4.484	5.101	5.587	0.3038	-0.0651	0.0800
1IPP	C56	956	4.751	4.988	5.520	0.3114	-0.0305	0.0630
1IPP	C57	957	4.759	4.775	5.314	0.3233	-0.0466	0.0772
1IPP	C58	958	4.708	4.496	5.393	0.3861	-0.0292	0.0729
1IPP	C59	959	4.441	4.364	5.389	0.3964	-0.0772	0.1081
1IPP	C60	960	4.341	4.198	5.164	0.4005	-0.1047	0.1289
1IPP	C61	961	4.191	4.315	4.936	0.3640	-0.1269	0.1356
1IPP	C62	962	3.913	4.369	5.021	0.3545	-0.1811	0.1633
1IPP	C63	963	3.884	4.634	5.150	0.3311	-0.1719	0.1721
1IPP	C64	964	3.787	4.843	4.964	0.2776	-0.1929	0.1667
1IPP	C65	965	4.013	4.942	4.802	0.2453	-0.1603	0.1367
1IPP	C66	966	4.076	4.841	4.530	0.2363	-0.1541	0.1384
1IPP	C67	967	4.239	4.594	4.560	0.2855	-0.1265	0.1280
1IPP	C68	968	4.506	4.645	4.679	0.2792	-0.0818	0.0881
1IPP	C69	969	4.485	4.652	4.975	0.3100	-0.0849	0.0956
1IPP	C70	970	4.363	4.901	5.082	0.2697	-0.0990	0.1133
1IPP	C71	971	4.543	5.130	5.137	0.2641	-0.0626	0.0872
1IPP	C72	972	4.522	5.400	5.020	0.1997	-0.0701	0.0793
1IPP	C73	973	4.636	5.473	4.759	0.1698	-0.0624	0.0656
1IPP	C74	974	4.917	5.555	4.726	0.1479	-0.0180	0.0263
1IPP	C75	975	4.885	5.848	4.714	0.1081	-0.0233	0.0215
1IPP	C76	976	4.737	5.915	4.464	0.0626	-0.0541	0.0452
1IPP	C77	977	4.465	6.034	4.455	0.0427	-0.0972	0.0710
1IPP	C78	978	4.216	5.890	4.528	0.0791	-0.1316	0.0968
1IPP	C79	979	4.058	5.711	4.352	0.0824	-0.1590	0.1219
1IPP	C80	980	3.868	5.833	4.159	0.0524	-0.1934	0.1321
1IPP	C81	981	3.908	5.830	3.866	0.0151	-0.1911	0.1252
1IPP	C82	982	3.746	5.650	3.695	0.0277	-0.2253	0.1520
1IPP	C83	983	3.487	5.791	3.722	0.0032	-0.2607	0.1697
1IPP	C84	984	3.356	5.776	3.989	0.0314	-0.2799	0.1848
1IPP	C85	985	3.117	5.606	4.026	0.0573	-0.3112	0.2094
1IPP	C86	986	2.862	5.751	3.970	0.0396	-0.3602	0.2325
1IPP	C87	987	2.796	5.889	4.223	0.0498	-0.3564	0.2419
1IPP	C88	988	2.832	5.673	4.423	0.0974	-0.3519	0.2445
1IPP	C89	989	3.092	5.637	4.561	0.1139	-0.3100	0.2216
1IPP	C90	990	3.295	5.432	4.486	0.1444	-0.2743	0.1980
1IPP	C91	991	3.547	5.526	4.358	0.1111	-0.2373	0.1769
1IPP	C92	992	3.637	5.444	4.087	0.0961	-0.2338	0.1648
1IPP	C93	993	3.845	5.235	4.051	0.1275	-0.1989	0.1454
1IPP	C94	994	4.119	5.332	4.117	0.1130	-0.1611	0.1253
1IPP	C95	995	4.247	5.544	3.952	0.0731	-0.1459	0.0966
1IPP	C96	996	4.441	5.496	3.732	0.0554	-0.1153	0.0879
1IPP	C97	997	4.714	5.432	3.830	0.0741	-0.0706	0.0567
1IPP	C98	998	4.845	5.628	4.010	0.0585	-0.0459	0.0443
1IPP	C99	999	4.778	5.519	4.277	0.1148	-0.0585	0.0440
1IPP	C100	1000	4.490	5.551	4.342	0.0992	-0.0974	0.0771
1IPP	C1	1001	5.718	9.224	5.711	-0.3274	0.1242	-0.1275
1IPP	C2	1002	5.527	9.059	5.555	-0.3203	0.0931	-0.1056
1IPP	C3	1003	5.536	9.070	5.259	-0.3519	0.0897	-0.1143
1IPP	C4	1004	5.392	9.265	5.085	-0.3995	0.0595	-0.0913
1IPP	C5	1005	5.106	9.264	4.999	-0.4046	0.0165	-0.0682
1IPP	C6	1006	5.013	9.128	4.752	-0.4157	-0.0019	-0.0573
1IPP	C7	1007	5.032	9.284	4.500	-0.4598	-0.0041	-0.0648
1IPP	C8	1008	4.841	9.492	4.404	-0.5073	-0.0468	-0.0554
1IPP	C9	1009	4.623	9.436	4.210	-0.5054	-0.0824	-0.0284
1IPP	C10	1010	4.710	9.507	3.935	-0.5514	-0.0633	-0.0299
1IPP	C11	1011	4.880	9.274	3.868	-0.5319	-0.0443	-0.0534
1IPP	C12	1012	5.120	9.306	4.039	-0.5074	-0.0112	-0.0702
1IPP	C13	1013	5.354	9.439	3.914	-0.5370	0.0316	-0.1027
1IPP	C14	1014	5.535	9.261	3.760	-0.5306	0.0572	-0.1072
1IPP	C15	1015	5.688	9.066	3.923	-0.4835	0.0845	-0.1192
1IPP	C16	1016	5.910	9.160	4.098	-0.4830	0.1228	-0.1497
1IPP	C17	1017	5.931	9.104	4.390	-0.4478	0.1389	-0.1513

1IPP	C18	1018	5.812	9.292	4.584	-0.4514	0.1148	-0.1393
1IPP	C19	1019	5.522	9.256	4.628	-0.4360	0.0666	-0.1083
1IPP	C20	1020	5.443	9.356	4.360	-0.4864	0.0551	-0.1145
1IPP	C21	1021	5.327	9.629	4.338	-0.5387	0.0328	-0.1076
1IPP	C22	1022	5.506	9.867	4.356	-0.5579	0.0661	-0.1191
1IPP	C23	1023	5.560	9.966	4.631	-0.5488	0.0783	-0.1277
1IPP	C24	1024	5.818	9.923	4.773	-0.5321	0.1315	-0.1603
1IPP	C25	1025	5.920	9.648	4.825	-0.4764	0.1491	-0.1595
1IPP	C26	1026	5.786	9.453	5.006	-0.4383	0.1218	-0.1434
1IPP	C27	1027	5.836	9.433	5.298	-0.4044	0.1522	-0.1543
1IPP	C28	1028	6.070	9.257	5.349	-0.3626	0.1647	-0.1598
1IPP	C29	1029	6.007	8.970	5.306	-0.3273	0.1618	-0.1541
1IPP	C30	1030	5.911	8.821	5.544	-0.2742	0.1550	-0.1434
1IPP	C31	1031	6.088	8.618	5.667	-0.2427	0.1877	-0.1550
1IPP	C32	1032	6.052	8.367	5.514	-0.2141	0.1653	-0.1439
1IPP	C33	1033	6.061	8.487	5.246	-0.2491	0.1778	-0.1477
1IPP	C34	1034	5.823	8.574	5.095	-0.2920	0.1218	-0.1245
1IPP	C35	1035	5.757	8.397	4.867	-0.2801	0.1253	-0.1103
1IPP	C36	1036	5.922	8.382	4.620	-0.3042	0.1374	-0.1347
1IPP	C37	1037	5.948	8.624	4.451	-0.3674	0.1377	-0.1444
1IPP	C38	1038	5.735	8.698	4.258	-0.3868	0.1005	-0.1139
1IPP	C39	1039	5.531	8.909	4.303	-0.4192	0.0663	-0.1016
1IPP	C40	1040	5.295	8.897	4.481	-0.3918	0.0380	-0.0732
1IPP	C41	1041	5.034	8.790	4.393	-0.4031	-0.0052	-0.0589
1IPP	C42	1042	4.869	8.948	4.205	-0.4339	-0.0454	-0.0366
1IPP	C43	1043	4.959	8.818	3.953	-0.4437	-0.0227	-0.0391
1IPP	C44	1044	5.190	8.939	3.813	-0.4702	0.0007	-0.0663
1IPP	C45	1045	5.177	9.062	3.544	-0.5206	0.0003	-0.0808
1IPP	C46	1046	5.257	8.882	3.322	-0.5144	0.0046	-0.0769
1IPP	C47	1047	5.550	8.878	3.264	-0.5229	0.0579	-0.1068
1IPP	C48	1048	5.750	8.682	3.368	-0.4781	0.0718	-0.1264
1IPP	C49	1049	5.837	8.729	3.648	-0.4607	0.1063	-0.1191
1IPP	C50	1050	6.062	8.917	3.700	-0.4853	0.1273	-0.1688
1IPP	C51	1051	6.288	8.809	3.857	-0.4452	0.1808	-0.1560
1IPP	C52	1052	6.198	8.833	4.137	-0.4127	0.1528	-0.1856
1IPP	C53	1053	6.355	8.943	4.363	-0.4157	0.1994	-0.1910
1IPP	C54	1054	6.465	8.715	4.518	-0.3650	0.2169	-0.1973
1IPP	C55	1055	6.313	8.578	4.733	-0.3241	0.2022	-0.1672
1IPP	C56	1056	6.372	8.693	4.998	-0.3156	0.2134	-0.1792
1IPP	C57	1057	6.662	8.632	4.996	-0.3008	0.2509	-0.2069
1IPP	C58	1058	6.800	8.375	5.047	-0.2616	0.2827	-0.2204
1IPP	C59	1059	6.877	8.357	5.331	-0.2295	0.2981	-0.2222
1IPP	C60	1060	7.125	8.510	5.377	-0.2504	0.3385	-0.2594
1IPP	C61	1061	7.101	8.791	5.466	-0.2901	0.3434	-0.2608
1IPP	C62	1062	7.102	9.005	5.259	-0.3378	0.3247	-0.2615
1IPP	C63	1063	6.864	9.177	5.205	-0.3686	0.2974	-0.2467
1IPP	C64	1064	6.641	9.068	5.041	-0.3687	0.2593	-0.2214
1IPP	C65	1065	6.573	9.088	4.753	-0.4104	0.2352	-0.2117
1IPP	C66	1066	6.329	9.253	4.711	-0.4366	0.2044	-0.1880
1IPP	C67	1067	6.119	9.143	4.890	-0.3990	0.1677	-0.1739
1IPP	C68	1068	5.975	8.890	4.832	-0.3635	0.1611	-0.1502
1IPP	C69	1069	5.697	8.848	4.733	-0.3650	0.0982	-0.1137
1IPP	C70	1070	5.454	8.846	4.906	-0.3492	0.0724	-0.0934
1IPP	C71	1071	5.381	8.636	5.106	-0.3021	0.0555	-0.0895
1IPP	C72	1072	5.381	8.640	5.403	-0.2684	0.0704	-0.0862
1IPP	C73	1073	5.612	8.511	5.537	-0.2366	0.0940	-0.1118
1IPP	C74	1074	5.612	8.220	5.488	-0.1952	0.1076	-0.1021
1IPP	C75	1075	5.557	8.198	5.197	-0.2214	0.0888	-0.0829
1IPP	C76	1076	5.276	8.173	5.105	-0.2219	0.0488	-0.0599
1IPP	C77	1077	5.061	8.322	5.244	-0.2283	0.0054	-0.0423
1IPP	C78	1078	4.862	8.502	5.115	-0.2831	-0.0177	-0.0333
1IPP	C79	1079	4.964	8.764	5.024	-0.3272	-0.0002	-0.0442
1IPP	C80	1080	5.026	8.923	5.266	-0.3195	0.0094	-0.0527
1IPP	C81	1081	4.789	9.045	5.396	-0.3338	-0.0164	-0.0383
1IPP	C82	1082	4.670	9.290	5.276	-0.3719	-0.0512	-0.0256
1IPP	C83	1083	4.375	9.319	5.241	-0.3959	-0.0842	0.0050
1IPP	C84	1084	4.209	9.222	5.014	-0.3979	-0.1272	0.0190
1IPP	C85	1085	4.213	9.439	4.810	-0.4531	-0.1170	0.0060
1IPP	C86	1086	4.459	9.408	4.647	-0.4651	-0.0904	-0.0128
1IPP	C87	1087	4.692	9.438	4.830	-0.4434	-0.0381	-0.0330
1IPP	C88	1088	4.840	9.691	4.874	-0.4889	-0.0325	-0.0500
1IPP	C89	1089	5.079	9.749	4.707	-0.5086	0.0092	-0.0847
1IPP	C90	1090	5.325	9.620	4.809	-0.4829	0.0477	-0.0999

1IPP	C91	1091	5.460	9.757	5.036	-0.4756	0.0691	-0.1111
1IPP	C92	1092	5.359	9.740	5.315	-0.4479	0.0605	-0.1072
1IPP	C93	1093	5.395	9.491	5.472	-0.3936	0.0748	-0.1130
1IPP	C94	1094	5.155	9.314	5.474	-0.3704	0.0425	-0.0762
1IPP	C95	1095	5.068	9.169	5.718	-0.3217	0.0298	-0.0630
1IPP	C96	1096	5.145	8.881	5.731	-0.2692	0.0384	-0.0716
1IPP	C97	1097	4.986	8.650	5.632	-0.2406	0.0246	-0.0450
1IPP	C98	1098	4.814	8.514	5.834	-0.2071	-0.0187	-0.0233
1IPP	C99	1099	4.920	8.457	6.107	-0.1622	-0.0005	-0.0242
1IPP	C100	1100	5.077	8.222	6.199	-0.1237	0.0436	-0.0416
1IPP	C1	1101	5.970	7.992	5.265	-0.1852	0.1654	-0.1332
1IPP	C2	1102	6.008	7.712	5.359	-0.1253	0.1583	-0.1218
1IPP	C3	1103	5.969	7.501	5.157	-0.1207	0.1518	-0.1145
1IPP	C4	1104	5.687	7.420	5.111	-0.0936	0.1035	-0.0839
1IPP	C5	1105	5.502	7.576	4.940	-0.1460	0.0820	-0.0741
1IPP	C6	1106	5.262	7.734	5.011	-0.1565	0.0379	-0.0523
1IPP	C7	1107	4.983	7.631	5.019	-0.1645	-0.0013	-0.0294
1IPP	C8	1108	4.814	7.613	4.775	-0.1646	-0.0280	0.0049
1IPP	C9	1109	4.636	7.822	4.660	-0.2268	-0.0607	0.0094
1IPP	C10	1110	4.704	7.947	4.398	-0.2655	-0.0645	0.0040
1IPP	C11	1111	4.866	8.186	4.332	-0.3010	-0.0367	-0.0187
1IPP	C12	1112	4.772	8.441	4.215	-0.3581	-0.0587	-0.0216
1IPP	C13	1113	4.767	8.388	3.923	-0.3835	-0.0549	-0.0100
1IPP	C14	1114	5.033	8.274	3.856	-0.3724	-0.0242	-0.0367
1IPP	C15	1115	5.082	8.013	3.986	-0.3095	-0.0037	-0.0329
1IPP	C16	1116	5.254	7.958	4.223	-0.2873	0.0181	-0.0477
1IPP	C17	1117	5.151	7.855	4.484	-0.2351	0.0141	-0.0509
1IPP	C18	1118	5.088	8.021	4.723	-0.2321	0.0112	-0.0391
1IPP	C19	1119	4.856	8.195	4.785	-0.2574	-0.0360	-0.0268
1IPP	C20	1120	4.924	8.457	4.666	-0.3167	-0.0151	-0.0294
1IPP	C21	1121	5.205	8.513	4.735	-0.3077	0.0344	-0.0678
1IPP	C22	1122	5.444	8.469	4.565	-0.3298	0.0542	-0.0893
1IPP	C23	1123	5.486	8.176	4.542	-0.2856	0.0682	-0.0939
1IPP	C24	1124	5.553	7.991	4.764	-0.2307	0.0752	-0.0823
1IPP	C25	1125	5.826	7.898	4.836	-0.2037	0.1242	-0.1100
1IPP	C26	1126	5.932	7.666	4.684	-0.1841	0.1505	-0.1213
1IPP	C27	1127	5.886	7.374	4.729	-0.1464	0.1314	-0.0992
1IPP	C28	1128	5.889	7.158	4.525	-0.1110	0.1267	-0.1081
1IPP	C29	1129	5.663	7.042	4.376	-0.1193	0.0823	-0.0814
1IPP	C30	1130	5.597	6.804	4.538	-0.0757	0.0875	-0.0635
1IPP	C31	1131	5.753	6.550	4.543	-0.0231	0.1028	-0.0813
1IPP	C32	1132	6.037	6.542	4.633	-0.0193	0.1491	-0.1071
1IPP	C33	1133	6.245	6.547	4.421	-0.0359	0.1789	-0.1197
1IPP	C34	1134	6.312	6.816	4.314	-0.0951	0.1922	-0.1327
1IPP	C35	1135	6.118	6.955	4.137	-0.1264	0.1519	-0.1134
1IPP	C36	1136	6.110	6.874	3.851	-0.1520	0.1424	-0.1179
1IPP	C37	1137	6.271	7.011	3.642	-0.1905	0.1650	-0.1392
1IPP	C38	1138	6.201	7.291	3.576	-0.2374	0.1592	-0.1343
1IPP	C39	1139	6.012	7.408	3.378	-0.2690	0.1176	-0.1185
1IPP	C40	1140	6.124	7.536	3.135	-0.3153	0.1366	-0.1378
1IPP	C41	1141	6.210	7.822	3.141	-0.3665	0.1444	-0.1481
1IPP	C42	1142	6.027	8.052	3.084	-0.4088	0.1127	-0.1475
1IPP	C43	1143	5.963	8.263	3.285	-0.4231	0.1195	-0.1308
1IPP	C44	1144	5.763	8.239	3.503	-0.4069	0.0891	-0.1054
1IPP	C45	1145	5.478	8.318	3.515	-0.4005	0.0555	-0.0858
1IPP	C46	1146	5.400	8.577	3.634	-0.4330	0.0249	-0.0800
1IPP	C47	1147	5.458	8.622	3.920	-0.4172	0.0525	-0.0988
1IPP	C48	1148	5.333	8.452	4.128	-0.3696	0.0304	-0.0655
1IPP	C49	1149	5.584	8.295	4.133	-0.3388	0.0712	-0.0891
1IPP	C50	1150	5.655	8.100	3.923	-0.3291	0.0859	-0.0961
1IPP	C51	1151	5.669	7.825	4.035	-0.2753	0.0802	-0.0941
1IPP	C52	1152	5.746	7.860	4.319	-0.2580	0.1010	-0.1032
1IPP	C53	1153	5.996	8.018	4.342	-0.2761	0.1384	-0.1332
1IPP	C54	1154	6.224	7.960	4.159	-0.2880	0.1732	-0.1459
1IPP	C55	1155	6.417	7.738	4.194	-0.2487	0.2006	-0.1734
1IPP	C56	1156	6.610	7.751	4.420	-0.2308	0.2338	-0.1767
1IPP	C57	1157	6.576	7.581	4.660	-0.1640	0.2392	-0.1899
1IPP	C58	1158	6.341	7.608	4.838	-0.1726	0.2033	-0.1499
1IPP	C59	1159	6.369	7.762	5.088	-0.1578	0.2187	-0.1583
1IPP	C60	1160	6.528	7.647	5.311	-0.1319	0.2438	-0.1765
1IPP	C61	1161	6.393	7.537	5.552	-0.0745	0.2343	-0.1603
1IPP	C62	1162	6.280	7.263	5.558	-0.0361	0.2128	-0.1374
1IPP	C63	1163	6.495	7.060	5.549	-0.0012	0.2344	-0.1590

1IPP	C64	1164	6.587	7.189	5.301	-0.0488	0.2535	-0.1811
1IPP	C65	1165	6.375	7.277	5.112	-0.0824	0.2139	-0.1502
1IPP	C66	1166	6.251	7.151	4.873	-0.0915	0.1965	-0.1404
1IPP	C67	1167	6.324	7.195	4.588	-0.1249	0.1981	-0.1418
1IPP	C68	1168	6.233	7.434	4.435	-0.1798	0.1760	-0.1491
1IPP	C69	1169	5.993	7.492	4.268	-0.1968	0.1388	-0.1133
1IPP	C70	1170	5.992	7.444	3.977	-0.2208	0.1419	-0.1191
1IPP	C71	1171	6.137	7.644	3.812	-0.2713	0.1411	-0.1322
1IPP	C72	1172	6.005	7.893	3.720	-0.3228	0.1319	-0.1273
1IPP	C73	1173	5.766	7.808	3.567	-0.3220	0.0842	-0.1053
1IPP	C74	1174	5.731	7.777	3.275	-0.3510	0.0729	-0.0966
1IPP	C75	1175	5.540	7.984	3.182	-0.3943	0.0350	-0.0863
1IPP	C76	1176	5.287	7.926	3.326	-0.3652	0.0059	-0.0597
1IPP	C77	1177	5.302	7.924	3.623	-0.3394	0.0188	-0.0626
1IPP	C78	1178	5.322	7.684	3.799	-0.2829	0.0143	-0.0548
1IPP	C79	1179	5.076	7.566	3.917	-0.2467	-0.0084	-0.0353
1IPP	C80	1180	4.926	7.614	4.168	-0.2307	-0.0258	-0.0140
1IPP	C81	1181	4.981	7.446	4.408	-0.1839	-0.0185	-0.0172
1IPP	C82	1182	4.811	7.216	4.486	-0.1429	-0.0423	0.0088
1IPP	C83	1183	4.548	7.263	4.617	-0.1237	-0.0717	0.0351
1IPP	C84	1184	4.509	7.304	4.909	-0.1105	-0.0796	0.0291
1IPP	C85	1185	4.539	7.122	5.140	-0.0614	-0.0649	0.0376
1IPP	C86	1186	4.795	7.233	5.238	-0.0648	-0.0231	0.0031
1IPP	C87	1187	4.843	7.391	5.485	-0.0645	-0.0055	-0.0065
1IPP	C88	1188	4.823	7.687	5.506	-0.1023	-0.0054	0.0048
1IPP	C89	1189	5.018	7.874	5.383	-0.1454	0.0066	-0.0279
1IPP	C90	1190	5.298	7.902	5.477	-0.1486	0.0699	-0.0560
1IPP	C91	1191	5.539	7.777	5.357	-0.1373	0.0854	-0.0804
1IPP	C92	1192	5.508	7.517	5.491	-0.0895	0.0883	-0.0705
1IPP	C93	1193	5.260	7.431	5.352	-0.0773	0.0466	-0.0423
1IPP	C94	1194	5.234	7.242	5.122	-0.0802	0.0429	-0.0275
1IPP	C95	1195	5.165	7.274	4.834	-0.1177	0.0285	-0.0347
1IPP	C96	1196	5.394	7.266	4.646	-0.1322	0.0538	-0.0559
1IPP	C97	1197	5.510	7.502	4.508	-0.1707	0.0745	-0.0662
1IPP	C98	1198	5.401	7.528	4.235	-0.2166	0.0503	-0.0597
1IPP	C99	1199	5.510	7.326	4.048	-0.2023	0.0587	-0.0664
1IPP	C100	1200	5.320	7.188	3.866	-0.1953	0.0235	-0.0354
1IPP	C1	1201	7.035	7.394	3.243	-0.2940	0.2757	-0.2189
1IPP	C2	1202	7.294	7.539	3.270	-0.3151	0.3242	-0.2655
1IPP	C3	1203	7.438	7.498	3.526	-0.2682	0.3452	-0.2624
1IPP	C4	1204	7.297	7.657	3.732	-0.2917	0.3341	-0.2434
1IPP	C5	1205	7.354	7.946	3.772	-0.3242	0.3357	-0.2756
1IPP	C6	1206	7.575	8.019	3.957	-0.3152	0.3734	-0.2812
1IPP	C7	1207	7.485	8.000	4.239	-0.2866	0.3833	-0.2873
1IPP	C8	1208	7.372	8.242	4.367	-0.2982	0.3595	-0.2744
1IPP	C9	1209	7.532	8.406	4.555	-0.3141	0.3842	-0.3025
1IPP	C10	1210	7.520	8.324	4.841	-0.2761	0.3917	-0.2905
1IPP	C11	1211	7.247	8.372	4.948	-0.2648	0.3500	-0.2658
1IPP	C12	1212	7.181	8.663	4.954	-0.3128	0.3341	-0.2727
1IPP	C13	1213	6.991	8.800	4.771	-0.3494	0.3137	-0.2536
1IPP	C14	1214	7.040	8.858	4.484	-0.3959	0.3097	-0.2463
1IPP	C15	1215	6.890	8.697	4.283	-0.3796	0.2788	-0.2402
1IPP	C16	1216	6.996	8.432	4.203	-0.3530	0.2864	-0.2518
1IPP	C17	1217	7.166	8.371	3.966	-0.3710	0.3153	-0.2500
1IPP	C18	1218	7.017	8.318	3.713	-0.3869	0.2894	-0.2607
1IPP	C19	1219	6.914	8.040	3.698	-0.3509	0.2643	-0.2233
1IPP	C20	1220	6.962	7.833	3.491	-0.3334	0.2785	-0.2208
1IPP	C21	1221	6.738	7.772	3.306	-0.3491	0.2284	-0.1954
1IPP	C22	1222	6.529	7.577	3.390	-0.3044	0.2041	-0.1815
1IPP	C23	1223	6.604	7.295	3.333	-0.2701	0.2181	-0.1788
1IPP	C24	1224	6.709	7.111	3.542	-0.2129	0.2384	-0.1721
1IPP	C25	1225	6.974	7.179	3.659	-0.2110	0.2741	-0.2093
1IPP	C26	1226	7.239	7.078	3.565	-0.2145	0.3281	-0.2377
1IPP	C27	1227	7.358	6.833	3.688	-0.1574	0.3347	-0.2400
1IPP	C28	1228	7.491	6.852	3.954	-0.1226	0.3709	-0.2489
1IPP	C29	1229	7.776	6.876	4.033	-0.1359	0.4090	-0.2743
1IPP	C30	1230	7.922	6.625	4.099	-0.0879	0.4370	-0.2925
1IPP	C31	1231	7.840	6.470	4.340	-0.0324	0.4271	-0.2871
1IPP	C32	1232	7.869	6.593	4.609	-0.0221	0.4346	-0.2914
1IPP	C33	1233	7.590	6.685	4.653	-0.0347	0.4007	-0.2537
1IPP	C34	1234	7.468	6.811	4.413	-0.0812	0.3583	-0.2539
1IPP	C35	1235	7.517	7.100	4.370	-0.1400	0.3813	-0.2505
1IPP	C36	1236	7.393	7.210	4.615	-0.1152	0.3633	-0.2602

1IPP	C37	1237	7.098	7.196	4.593	-0.1212	0.3216	-0.2228
1IPP	C38	1238	6.931	7.410	4.474	-0.1661	0.2848	-0.2104
1IPP	C39	1239	6.917	7.495	4.190	-0.2146	0.2819	-0.2078
1IPP	C40	1240	7.097	7.720	4.113	-0.2546	0.3153	-0.2433
1IPP	C41	1241	7.003	7.977	4.225	-0.2751	0.2886	-0.2355
1IPP	C42	1242	6.734	8.060	4.131	-0.3096	0.2606	-0.2096
1IPP	C43	1243	6.638	8.286	3.965	-0.3529	0.2317	-0.2003
1IPP	C44	1244	6.505	8.498	4.122	-0.3824	0.2175	-0.1931
1IPP	C45	1245	6.234	8.402	4.197	-0.3515	0.1847	-0.1634
1IPP	C46	1246	6.017	8.433	3.998	-0.3758	0.1340	-0.1427
1IPP	C47	1247	6.120	8.329	3.741	-0.3835	0.1470	-0.1538
1IPP	C48	1248	6.258	8.500	3.541	-0.4303	0.1694	-0.1652
1IPP	C49	1249	6.552	8.467	3.558	-0.4307	0.2127	-0.1947
1IPP	C50	1250	6.599	8.220	3.400	-0.3884	0.2237	-0.2014
1IPP	C51	1251	6.411	8.017	3.510	-0.3632	0.1796	-0.1639
1IPP	C52	1252	6.486	7.912	3.776	-0.3170	0.2023	-0.1814
1IPP	C53	1253	6.678	7.691	3.830	-0.2796	0.2401	-0.1936
1IPP	C54	1254	6.554	7.424	3.824	-0.2375	0.2191	-0.1696
1IPP	C55	1255	6.413	7.302	4.055	-0.1860	0.2103	-0.1580
1IPP	C56	1256	6.605	7.167	4.235	-0.1546	0.2342	-0.1875
1IPP	C57	1257	6.689	7.020	3.994	-0.1566	0.2352	-0.1749
1IPP	C58	1258	6.557	6.775	3.896	-0.1227	0.2219	-0.1612
1IPP	C59	1259	6.730	6.539	3.932	-0.0819	0.2481	-0.1726
1IPP	C60	1260	6.939	6.509	3.722	-0.1040	0.2729	-0.1946
1IPP	C61	1261	6.912	6.320	3.494	-0.0919	0.2666	-0.1867
1IPP	C62	1262	6.997	6.039	3.550	-0.0576	0.2742	-0.1965
1IPP	C63	1263	7.283	5.964	3.593	-0.0279	0.3317	-0.2138
1IPP	C64	1264	7.426	5.994	3.852	-0.0119	0.3647	-0.2302
1IPP	C65	1265	7.525	6.272	3.893	-0.0457	0.3681	-0.2375
1IPP	C66	1266	7.433	6.430	4.127	-0.0522	0.3610	-0.2480
1IPP	C67	1267	7.159	6.542	4.146	-0.0597	0.3142	-0.2134
1IPP	C68	1268	7.068	6.805	4.039	-0.1227	0.2904	-0.2164
1IPP	C69	1269	7.115	7.072	4.160	-0.1456	0.3162	-0.2198
1IPP	C70	1270	7.254	7.265	3.983	-0.1946	0.3325	-0.2343
1IPP	C71	1271	7.544	7.314	3.956	-0.2082	0.3763	-0.2697
1IPP	C72	1272	7.577	7.578	4.085	-0.2363	0.3816	-0.2823
1IPP	C73	1273	7.539	7.561	4.378	-0.2050	0.3828	-0.2837
1IPP	C74	1274	7.302	7.660	4.527	-0.2082	0.3566	-0.2534
1IPP	C75	1275	7.350	7.914	4.673	-0.2316	0.3543	-0.2651
1IPP	C76	1276	7.508	7.883	4.922	-0.1907	0.3882	-0.2837
1IPP	C77	1277	7.399	7.921	5.195	-0.1782	0.3744	-0.2712
1IPP	C78	1278	7.457	8.189	5.312	-0.2102	0.3944	-0.2858
1IPP	C79	1279	7.728	8.235	5.427	-0.1957	0.4388	-0.3034
1IPP	C80	1280	7.814	8.040	5.635	-0.1525	0.4483	-0.3058
1IPP	C81	1281	8.020	7.864	5.513	-0.1377	0.4767	-0.3442
1IPP	C82	1282	7.916	7.810	5.241	-0.1374	0.4669	-0.3047
1IPP	C83	1283	7.718	7.595	5.188	-0.1271	0.4162	-0.3033
1IPP	C84	1284	7.755	7.350	5.030	-0.0976	0.4375	-0.2841
1IPP	C85	1285	7.756	7.474	4.762	-0.1456	0.4205	-0.2930
1IPP	C86	1286	8.048	7.456	4.718	-0.1501	0.4780	-0.3239
1IPP	C87	1287	8.184	7.592	4.943	-0.1529	0.4961	-0.3424
1IPP	C88	1288	8.197	7.875	4.855	-0.2048	0.4952	-0.3439
1IPP	C89	1289	7.934	8.003	4.798	-0.2296	0.4570	-0.3298
1IPP	C90	1290	7.805	7.876	4.562	-0.2268	0.4307	-0.3129
1IPP	C91	1291	7.972	7.772	4.339	-0.2315	0.4452	-0.3285
1IPP	C92	1292	7.986	7.492	4.231	-0.2043	0.4560	-0.3219
1IPP	C93	1293	8.129	7.251	4.332	-0.1606	0.4667	-0.3250
1IPP	C94	1294	7.962	7.022	4.422	-0.1099	0.4478	-0.3077
1IPP	C95	1295	7.828	7.050	4.685	-0.0920	0.4310	-0.3012
1IPP	C96	1296	7.972	6.955	4.924	-0.0523	0.4527	-0.3060
1IPP	C97	1297	8.239	7.084	4.919	-0.0736	0.5077	-0.3413
1IPP	C98	1298	8.435	6.926	4.761	-0.0590	0.5313	-0.3624
1IPP	C99	1299	8.478	6.963	4.469	-0.0962	0.5306	-0.3552
1IPP	C100	1300	8.328	6.819	4.255	-0.0917	0.5089	-0.3427
1IPP	C1	1301	6.327	4.656	6.880	0.5000	0.2509	-0.0962
1IPP	C2	1302	6.453	4.683	6.612	0.4743	0.2675	-0.0946
1IPP	C3	1303	6.657	4.502	6.492	0.4842	0.2867	-0.1186
1IPP	C4	1304	6.939	4.519	6.583	0.4939	0.3357	-0.1478
1IPP	C5	1305	7.094	4.767	6.529	0.4515	0.3562	-0.1626
1IPP	C6	1306	7.124	4.851	6.246	0.4125	0.3614	-0.1736
1IPP	C7	1307	7.316	4.701	6.077	0.4226	0.3864	-0.1909
1IPP	C8	1308	7.606	4.735	6.128	0.4191	0.4365	-0.2219
1IPP	C9	1309	7.679	5.017	6.075	0.3620	0.4410	-0.2274

1IPP	C10	1310	7.667	5.113	5.795	0.3185	0.4281	-0.2248
1IPP	C11	1311	7.915	5.146	5.635	0.3029	0.4741	-0.2623
1IPP	C12	1312	8.005	4.892	5.509	0.3358	0.4907	-0.2597
1IPP	C13	1313	7.834	4.797	5.285	0.3130	0.4417	-0.2457
1IPP	C14	1314	7.575	4.830	5.424	0.3329	0.4144	-0.2220
1IPP	C15	1315	7.516	4.667	5.665	0.3764	0.4018	-0.2115
1IPP	C16	1316	7.309	4.453	5.667	0.4085	0.3795	-0.1834
1IPP	C17	1317	7.030	4.540	5.724	0.4128	0.3379	-0.1510
1IPP	C18	1318	6.893	4.534	5.988	0.4294	0.3159	-0.1401
1IPP	C19	1319	6.694	4.319	6.038	0.4734	0.2958	-0.1192
1IPP	C20	1320	6.406	4.368	5.976	0.4564	0.2420	-0.0915
1IPP	C21	1321	6.295	4.515	6.208	0.4492	0.2340	-0.0799
1IPP	C22	1322	6.361	4.804	6.188	0.4154	0.2426	-0.0901
1IPP	C23	1323	6.649	4.870	6.210	0.4032	0.2859	-0.1193
1IPP	C24	1324	6.748	5.034	6.436	0.3954	0.3018	-0.1463
1IPP	C25	1325	6.813	5.308	6.348	0.3410	0.3109	-0.1421
1IPP	C26	1326	6.996	5.293	6.115	0.3340	0.3372	-0.1783
1IPP	C27	1327	7.290	5.244	6.094	0.3267	0.3824	-0.1981
1IPP	C28	1328	7.481	5.467	6.045	0.2998	0.4036	-0.2294
1IPP	C29	1329	7.574	5.552	5.774	0.2655	0.4179	-0.2354
1IPP	C30	1330	7.850	5.637	5.706	0.2362	0.4588	-0.2588
1IPP	C31	1331	7.893	5.930	5.672	0.1848	0.4683	-0.2860
1IPP	C32	1332	7.824	6.036	5.404	0.1413	0.4480	-0.2637
1IPP	C33	1333	7.529	6.059	5.376	0.1387	0.4081	-0.2361
1IPP	C34	1334	7.414	5.785	5.382	0.1770	0.3892	-0.2214
1IPP	C35	1335	7.335	5.633	5.138	0.1736	0.3680	-0.2101
1IPP	C36	1336	7.041	5.629	5.091	0.1776	0.3116	-0.1856
1IPP	C37	1337	6.853	5.487	5.274	0.2124	0.3037	-0.1581
1IPP	C38	1338	6.793	5.200	5.318	0.2542	0.2817	-0.1492
1IPP	C39	1339	6.578	5.051	5.180	0.2653	0.2680	-0.1220
1IPP	C40	1340	6.685	4.806	5.049	0.2932	0.2571	-0.1329
1IPP	C41	1341	6.921	4.864	4.880	0.2746	0.2952	-0.1614
1IPP	C42	1342	6.916	4.928	4.590	0.2373	0.2923	-0.1584
1IPP	C43	1343	7.020	4.688	4.451	0.2543	0.3021	-0.1554
1IPP	C44	1344	7.315	4.655	4.483	0.2644	0.3368	-0.1820
1IPP	C45	1345	7.432	4.482	4.696	0.3041	0.3788	-0.1949
1IPP	C46	1346	7.508	4.570	4.970	0.3278	0.3861	-0.2027
1IPP	C47	1347	7.342	4.503	5.207	0.3592	0.3707	-0.1937
1IPP	C48	1348	7.108	4.671	5.277	0.3459	0.3346	-0.1733
1IPP	C49	1349	7.143	4.931	5.416	0.3132	0.3457	-0.1689
1IPP	C50	1350	7.189	4.981	5.705	0.3392	0.3545	-0.1857
1IPP	C51	1351	6.921	4.980	5.832	0.3511	0.3152	-0.1479
1IPP	C52	1352	6.718	5.180	5.744	0.3062	0.2840	-0.1331
1IPP	C53	1353	6.704	5.471	5.804	0.2686	0.2832	-0.1476
1IPP	C54	1354	6.705	5.709	5.624	0.2149	0.2747	-0.1472
1IPP	C55	1355	6.919	5.860	5.479	0.1748	0.3116	-0.1751
1IPP	C56	1356	7.098	6.066	5.595	0.1595	0.3370	-0.1968
1IPP	C57	1357	6.987	6.221	5.824	0.1525	0.3286	-0.1787
1IPP	C58	1358	7.147	6.266	6.069	0.1674	0.3516	-0.2056
1IPP	C59	1359	7.129	6.089	6.306	0.2222	0.3636	-0.2113
1IPP	C60	1360	6.948	6.192	6.517	0.2220	0.3311	-0.1907
1IPP	C61	1361	7.093	6.431	6.621	0.2095	0.3732	-0.2112
1IPP	C62	1362	7.062	6.696	6.488	0.1473	0.3509	-0.2146
1IPP	C63	1363	7.166	6.693	6.209	0.1155	0.3599	-0.2125
1IPP	C64	1364	7.456	6.699	6.143	0.1124	0.4070	-0.2455
1IPP	C65	1365	7.594	6.437	6.177	0.1559	0.4277	-0.2630
1IPP	C66	1366	7.687	6.254	5.961	0.1579	0.4529	-0.2625
1IPP	C67	1367	7.483	6.091	5.819	0.1684	0.3983	-0.2363
1IPP	C68	1368	7.335	5.875	5.961	0.2166	0.3917	-0.2270
1IPP	C69	1369	7.108	5.712	5.859	0.2492	0.3363	-0.1837
1IPP	C70	1370	7.137	5.446	5.730	0.2601	0.3544	-0.1883
1IPP	C71	1371	7.234	5.369	5.462	0.2473	0.3581	-0.1970
1IPP	C72	1372	7.509	5.272	5.407	0.2676	0.4033	-0.2177
1IPP	C73	1373	7.735	5.446	5.325	0.2213	0.4300	-0.2511
1IPP	C74	1374	7.971	5.335	5.183	0.2248	0.4690	-0.2623
1IPP	C75	1375	8.220	5.260	5.326	0.2550	0.5125	-0.3013
1IPP	C76	1376	8.395	5.489	5.401	0.2251	0.5430	-0.3221
1IPP	C77	1377	8.289	5.652	5.625	0.2234	0.5294	-0.3068
1IPP	C78	1378	8.360	5.592	5.906	0.2581	0.5451	-0.3031
1IPP	C79	1379	8.212	5.351	5.997	0.3089	0.5247	-0.2934
1IPP	C80	1380	7.927	5.388	6.071	0.3099	0.4813	-0.2734
1IPP	C81	1381	7.822	5.441	6.344	0.3223	0.4682	-0.2557
1IPP	C82	1382	7.735	5.217	6.521	0.3792	0.4543	-0.2398

1IPP	C83	1383	7.475	5.078	6.475	0.3995	0.4278	-0.2130
1IPP	C84	1384	7.219	5.211	6.543	0.3893	0.3710	-0.1934
1IPP	C85	1385	7.133	5.216	6.827	0.4066	0.3711	-0.1757
1IPP	C86	1386	7.029	4.967	6.952	0.4692	0.3581	-0.1651
1IPP	C87	1387	6.754	4.911	6.858	0.4608	0.3068	-0.1356
1IPP	C88	1388	6.584	5.149	6.913	0.4314	0.2960	-0.1199
1IPP	C89	1389	6.593	5.386	6.733	0.3819	0.2844	-0.1308
1IPP	C90	1390	6.825	5.574	6.746	0.3483	0.3211	-0.1563
1IPP	C91	1391	6.938	5.719	6.511	0.2996	0.3409	-0.1689
1IPP	C92	1392	7.171	5.622	6.353	0.3200	0.3701	-0.1877
1IPP	C93	1393	7.440	5.686	6.458	0.2884	0.4167	-0.2312
1IPP	C94	1394	7.582	5.916	6.334	0.2536	0.4288	-0.2453
1IPP	C95	1395	7.763	5.835	6.113	0.2338	0.4531	-0.2547
1IPP	C96	1396	8.053	5.829	6.173	0.2546	0.4972	-0.2869
1IPP	C97	1397	8.173	6.091	6.102	0.2003	0.5151	-0.3085
1IPP	C98	1398	8.282	6.075	5.827	0.1781	0.5329	-0.3119
1IPP	C99	1399	8.569	6.018	5.773	0.1884	0.5798	-0.3467
1IPP	C100	1400	8.730	6.240	5.660	0.1310	0.5965	-0.3563
1IPP	C1	1401	4.175	6.776	3.141	-0.2087	-0.1713	0.0855
1IPP	C2	1402	4.083	7.016	3.292	-0.2327	-0.1834	0.0885
1IPP	C3	1403	3.902	6.951	3.518	-0.1897	-0.2078	0.0953
1IPP	C4	1404	3.606	6.920	3.497	-0.1878	-0.2503	0.1329
1IPP	C5	1405	3.422	6.952	3.727	-0.1739	-0.2638	0.1581
1IPP	C6	1406	3.342	6.711	3.885	-0.1208	-0.2803	0.1689
1IPP	C7	1407	3.081	6.576	3.846	-0.1077	-0.3338	0.1901
1IPP	C8	1408	2.829	6.610	3.998	-0.0988	-0.3539	0.2261
1IPP	C9	1409	2.704	6.365	4.110	-0.0427	-0.3843	0.2422
1IPP	C10	1410	2.641	6.308	4.394	-0.0031	-0.3823	0.2362
1IPP	C11	1411	2.861	6.208	4.563	0.0235	-0.3438	0.2297
1IPP	C12	1412	2.973	6.377	4.779	0.0240	-0.3192	0.2180
1IPP	C13	1413	3.162	6.601	4.733	-0.0216	-0.2908	0.1825
1IPP	C14	1414	3.431	6.497	4.671	0.0002	-0.2519	0.1570
1IPP	C15	1415	3.396	6.541	4.380	-0.0454	-0.2659	0.1712
1IPP	C16	1416	3.143	6.434	4.269	-0.0383	-0.3081	0.1924
1IPP	C17	1417	3.146	6.172	4.131	-0.0075	-0.3025	0.2013
1IPP	C18	1418	3.330	6.203	3.901	-0.0505	-0.2865	0.1849
1IPP	C19	1419	3.616	6.181	3.972	-0.0197	-0.2363	0.1534
1IPP	C20	1420	3.679	6.463	4.027	-0.0661	-0.2329	0.1342
1IPP	C21	1421	3.794	6.604	3.795	-0.1186	-0.2192	0.1330
1IPP	C22	1422	4.085	6.654	3.827	-0.1126	-0.1677	0.0826
1IPP	C23	1423	4.113	6.933	3.916	-0.1483	-0.1579	0.0760
1IPP	C24	1424	3.926	6.948	4.147	-0.1274	-0.1868	0.1047
1IPP	C25	1425	3.630	6.949	4.133	-0.1323	-0.2273	0.1353
1IPP	C26	1426	3.493	7.213	4.120	-0.1734	-0.2489	0.1398
1IPP	C27	1427	3.420	7.349	4.374	-0.1767	-0.2575	0.1441
1IPP	C28	1428	3.159	7.349	4.516	-0.1460	-0.3041	0.1714
1IPP	C29	1429	2.997	7.599	4.513	-0.2068	-0.3233	0.1822
1IPP	C30	1430	3.089	7.825	4.683	-0.2146	-0.3001	0.1622
1IPP	C31	1431	3.232	8.057	4.565	-0.2583	-0.2742	0.1405
1IPP	C32	1432	3.513	7.999	4.491	-0.2735	-0.2404	0.1103
1IPP	C33	1433	3.715	7.912	4.689	-0.2180	-0.2128	0.1083
1IPP	C34	1434	3.721	7.616	4.666	-0.1798	-0.1986	0.1064
1IPP	C35	1435	3.552	7.408	4.794	-0.1338	-0.2299	0.1336
1IPP	C36	1436	3.611	7.302	5.065	-0.0926	-0.2186	0.1186
1IPP	C37	1437	3.773	7.062	5.132	-0.0476	-0.1845	0.1199
1IPP	C38	1438	4.070	7.087	5.141	-0.0474	-0.1379	0.0814
1IPP	C39	1439	4.161	7.021	4.868	-0.0749	-0.1294	0.0790
1IPP	C40	1440	4.108	6.730	4.843	-0.0238	-0.1378	0.0858
1IPP	C41	1441	3.818	6.670	4.866	-0.0206	-0.1929	0.1254
1IPP	C42	1442	3.687	6.589	5.120	0.0280	-0.1940	0.1287
1IPP	C43	1443	3.600	6.305	5.132	0.0717	-0.2158	0.1563
1IPP	C44	1444	3.320	6.241	5.051	0.0694	-0.2644	0.1874
1IPP	C45	1445	3.272	6.041	4.837	0.0705	-0.2702	0.1826
1IPP	C46	1446	3.288	6.089	4.545	0.0431	-0.2739	0.1875
1IPP	C47	1447	3.530	6.026	4.387	0.0521	-0.2384	0.1578
1IPP	C48	1448	3.767	6.176	4.487	0.0175	-0.1896	0.1353
1IPP	C49	1449	3.829	6.465	4.452	-0.0184	-0.2033	0.1273
1IPP	C50	1450	4.046	6.545	4.268	-0.0446	-0.1612	0.1058
1IPP	C51	1451	4.299	6.658	4.367	-0.0711	-0.1209	0.0632
1IPP	C52	1452	4.281	6.947	4.437	-0.1000	-0.1315	0.0659
1IPP	C53	1453	4.305	7.162	4.232	-0.1487	-0.1223	0.0535
1IPP	C54	1454	4.523	7.349	4.165	-0.1941	-0.0933	0.0350
1IPP	C55	1455	4.451	7.614	4.277	-0.2138	-0.1076	0.0302

1IPP	C56	1456	4.306	7.834	4.136	-0.2777	-0.1310	0.0468
1IPP	C57	1457	4.457	8.071	4.035	-0.3132	-0.1051	0.0189
1IPP	C58	1458	4.530	8.017	3.753	-0.3373	-0.1032	0.0170
1IPP	C59	1459	4.674	7.759	3.763	-0.2914	-0.0869	0.0093
1IPP	C60	1460	4.446	7.569	3.791	-0.2648	-0.1215	0.0305
1IPP	C61	1461	4.348	7.365	3.600	-0.2478	-0.1258	0.0573
1IPP	C62	1462	4.237	7.466	3.344	-0.2834	-0.1475	0.0541
1IPP	C63	1463	3.972	7.595	3.310	-0.3112	-0.1941	0.0825
1IPP	C64	1464	3.768	7.383	3.341	-0.2785	-0.2276	0.1104
1IPP	C65	1465	3.659	7.371	3.615	-0.2589	-0.2405	0.1106
1IPP	C66	1466	3.839	7.265	3.824	-0.2071	-0.2105	0.1146
1IPP	C67	1467	4.006	7.418	4.017	-0.2207	-0.1767	0.0797
1IPP	C68	1468	3.886	7.421	4.289	-0.1903	-0.1827	0.0935
1IPP	C69	1469	3.930	7.248	4.527	-0.1394	-0.1757	0.0984
1IPP	C70	1470	3.790	6.988	4.566	-0.0894	-0.2060	0.1128
1IPP	C71	1471	3.496	6.943	4.580	-0.0914	-0.2470	0.1465
1IPP	C72	1472	3.325	7.024	4.808	-0.0730	-0.2636	0.1582
1IPP	C73	1473	3.334	6.908	5.081	-0.0292	-0.2630	0.1513
1IPP	C74	1474	3.181	6.676	5.187	0.0140	-0.2893	0.1874
1IPP	C75	1475	2.903	6.690	5.087	0.0067	-0.3294	0.2202
1IPP	C76	1476	2.774	6.955	5.054	-0.0443	-0.3439	0.2242
1IPP	C77	1477	2.866	7.103	4.815	-0.0795	-0.3305	0.2095
1IPP	C78	1478	2.751	7.057	4.546	-0.1105	-0.3580	0.2184
1IPP	C79	1479	2.882	6.835	4.398	-0.0862	-0.3437	0.2096
1IPP	C80	1480	3.131	6.896	4.247	-0.1051	-0.3109	0.1782
1IPP	C81	1481	3.092	7.069	4.009	-0.1646	-0.3219	0.1870
1IPP	C82	1482	2.908	7.021	3.782	-0.1734	-0.3422	0.2087
1IPP	C83	1483	2.981	6.868	3.539	-0.1764	-0.3586	0.1894
1IPP	C84	1484	3.165	6.965	3.329	-0.2136	-0.3296	0.1838
1IPP	C85	1485	3.076	7.097	3.079	-0.2601	-0.3371	0.1867
1IPP	C86	1486	3.139	7.387	3.094	-0.3080	-0.3421	0.1640
1IPP	C87	1487	3.297	7.430	3.343	-0.2795	-0.3046	0.1542
1IPP	C88	1488	3.181	7.342	3.603	-0.2479	-0.3139	0.1708
1IPP	C89	1489	2.995	7.511	3.763	-0.2579	-0.3385	0.1922
1IPP	C90	1490	3.011	7.515	4.059	-0.2270	-0.3262	0.1712
1IPP	C91	1491	2.818	7.337	4.199	-0.1879	-0.3627	0.2074
1IPP	C92	1492	2.546	7.421	4.280	-0.1897	-0.4023	0.2267
1IPP	C93	1493	2.524	7.579	4.530	-0.1889	-0.3948	0.2297
1IPP	C94	1494	2.583	7.465	4.798	-0.1514	-0.3783	0.2272
1IPP	C95	1495	2.816	7.571	4.951	-0.1364	-0.3461	0.2074
1IPP	C96	1496	3.092	7.460	4.967	-0.1346	-0.3008	0.1686
1IPP	C97	1497	3.095	7.275	5.198	-0.0816	-0.2781	0.1827
1IPP	C98	1498	3.061	7.441	5.441	-0.0911	-0.2901	0.1716
1IPP	C99	1499	2.783	7.527	5.506	-0.0722	-0.3404	0.2031
1IPP	C100	1500	2.639	7.740	5.356	-0.1329	-0.3629	0.2070
1IPP	C1	1501	3.348	5.859	3.240	-0.0510	-0.2938	0.1844
1IPP	C2	1502	3.315	6.141	3.152	-0.1041	-0.3114	0.1722
1IPP	C3	1503	3.190	6.197	2.889	-0.1423	-0.3364	0.2006
1IPP	C4	1504	3.341	6.276	2.646	-0.1689	-0.3250	0.1698
1IPP	C5	1505	3.264	6.514	2.484	-0.2276	-0.3298	0.1746
1IPP	C6	1506	3.396	6.779	2.486	-0.2656	-0.2961	0.1654
1IPP	C7	1507	3.301	6.983	2.681	-0.2838	-0.3222	0.1554
1IPP	C8	1508	3.082	7.177	2.629	-0.3235	-0.3507	0.1809
1IPP	C9	1509	3.140	7.450	2.529	-0.3677	-0.3433	0.1748
1IPP	C10	1510	3.125	7.575	2.259	-0.4127	-0.3561	0.1627
1IPP	C11	1511	3.330	7.550	2.045	-0.4321	-0.3205	0.1517
1IPP	C12	1512	3.604	7.665	2.054	-0.4621	-0.2918	0.1112
1IPP	C13	1513	3.824	7.465	2.030	-0.4245	-0.2485	0.1002
1IPP	C14	1514	3.946	7.338	2.269	-0.3897	-0.2261	0.0888
1IPP	C15	1515	3.772	7.163	2.433	-0.3379	-0.2466	0.1127
1IPP	C16	1516	3.594	7.313	2.614	-0.3427	-0.2691	0.1301
1IPP	C17	1517	3.684	7.290	2.894	-0.3092	-0.2521	0.1156
1IPP	C18	1518	3.517	7.130	3.078	-0.2746	-0.2706	0.1361
1IPP	C19	1519	3.504	6.835	3.079	-0.2168	-0.2762	0.1558
1IPP	C20	1520	3.293	6.649	2.982	-0.2072	-0.3174	0.1705
1IPP	C21	1521	3.087	6.555	3.175	-0.1607	-0.3431	0.1953
1IPP	C22	1522	3.175	6.456	3.441	-0.1216	-0.3151	0.1826
1IPP	C23	1523	3.458	6.415	3.523	-0.1084	-0.2726	0.1606
1IPP	C24	1524	3.622	6.164	3.515	-0.0713	-0.2353	0.1602
1IPP	C25	1525	3.762	6.033	3.284	-0.0764	-0.2360	0.1308
1IPP	C26	1526	4.038	6.071	3.177	-0.0918	-0.2007	0.1192
1IPP	C27	1527	4.080	6.325	3.028	-0.1480	-0.1884	0.0928
1IPP	C28	1528	4.137	6.393	2.747	-0.1967	-0.1875	0.0993

1IPP	C29	1529	4.387	6.538	2.682	-0.2129	-0.1422	0.0680
1IPP	C30	1530	4.653	6.410	2.644	-0.1927	-0.1062	0.0408
1IPP	C31	1531	4.863	6.399	2.856	-0.1581	-0.0610	0.0177
1IPP	C32	1532	5.075	6.607	2.878	-0.2143	-0.0441	-0.0059
1IPP	C33	1533	5.055	6.845	3.056	-0.2229	-0.0332	-0.0090
1IPP	C34	1534	4.962	7.110	2.960	-0.2802	-0.0644	-0.0006
1IPP	C35	1535	5.137	7.346	2.918	-0.3029	-0.0141	-0.0215
1IPP	C36	1536	5.141	7.560	3.124	-0.3380	-0.0190	-0.0347
1IPP	C37	1537	4.892	7.719	3.148	-0.3499	-0.0537	-0.0186
1IPP	C38	1538	4.704	7.593	3.341	-0.3207	-0.0857	0.0029
1IPP	C39	1539	4.662	7.315	3.245	-0.2856	-0.0866	0.0191
1IPP	C40	1540	4.501	7.323	2.997	-0.2983	-0.1222	0.0409
1IPP	C41	1541	4.632	7.317	2.731	-0.3283	-0.1087	0.0201
1IPP	C42	1542	4.812	7.551	2.698	-0.3737	-0.0795	-0.0049
1IPP	C43	1543	4.640	7.788	2.735	-0.3968	-0.1054	0.0183
1IPP	C44	1544	4.422	7.768	2.535	-0.4235	-0.1434	0.0346
1IPP	C45	1545	4.273	7.512	2.515	-0.3772	-0.1684	0.0466
1IPP	C46	1546	4.073	7.391	2.699	-0.3346	-0.1896	0.0783
1IPP	C47	1547	4.121	7.142	2.854	-0.2905	-0.1792	0.0785
1IPP	C48	1548	4.043	6.890	2.716	-0.2721	-0.2056	0.0844
1IPP	C49	1549	3.767	6.782	2.726	-0.2500	-0.2398	0.1184
1IPP	C50	1550	3.720	6.511	2.838	-0.1951	-0.2536	0.1312
1IPP	C51	1551	3.660	6.439	3.120	-0.1517	-0.2606	0.1362
1IPP	C52	1552	3.831	6.549	3.337	-0.1502	-0.2237	0.1153
1IPP	C53	1553	4.068	6.416	3.455	-0.1122	-0.1717	0.1020
1IPP	C54	1554	4.049	6.208	3.666	-0.0691	-0.1682	0.1132
1IPP	C55	1555	4.093	6.230	3.959	-0.0394	-0.1728	0.0927
1IPP	C56	1556	4.375	6.224	4.052	-0.0285	-0.1150	0.0695
1IPP	C57	1557	4.474	5.949	3.997	0.0140	-0.1018	0.0669
1IPP	C58	1558	4.611	5.920	3.734	-0.0199	-0.0886	0.0482
1IPP	C59	1559	4.422	5.976	3.511	-0.0509	-0.1261	0.0802
1IPP	C60	1560	4.177	5.809	3.512	-0.0014	-0.1529	0.0975
1IPP	C61	1561	4.130	5.560	3.358	0.0045	-0.1832	0.1092
1IPP	C62	1562	4.005	5.581	3.089	-0.0267	-0.1873	0.1241
1IPP	C63	1563	3.711	5.626	3.085	-0.0274	-0.2387	0.1492
1IPP	C64	1564	3.595	5.791	2.866	-0.0766	-0.2790	0.1666
1IPP	C65	1565	3.706	6.064	2.835	-0.1168	-0.2440	0.1435
1IPP	C66	1566	3.937	6.008	2.658	-0.1309	-0.2165	0.1211
1IPP	C67	1567	4.188	5.896	2.771	-0.0976	-0.1808	0.0996
1IPP	C68	1568	4.438	6.050	2.824	-0.1290	-0.1369	0.0760
1IPP	C69	1569	4.533	6.147	3.089	-0.1159	-0.1184	0.0524
1IPP	C70	1570	4.474	6.421	3.187	-0.1500	-0.1201	0.0571
1IPP	C71	1571	4.640	6.662	3.139	-0.1887	-0.0888	0.0343
1IPP	C72	1572	4.580	6.879	2.945	-0.2458	-0.1086	0.0380
1IPP	C73	1573	4.674	6.863	2.664	-0.2558	-0.1040	0.0201
1IPP	C74	1574	4.941	6.929	2.550	-0.2895	-0.0676	0.0002
1IPP	C75	1575	4.970	7.204	2.445	-0.3372	-0.0681	-0.0007
1IPP	C76	1576	4.791	7.189	2.208	-0.3650	-0.0875	0.0070
1IPP	C77	1577	4.502	7.243	2.255	-0.3612	-0.1359	0.0368
1IPP	C78	1578	4.338	7.051	2.413	-0.3201	-0.1598	0.0634
1IPP	C79	1579	4.203	6.814	2.292	-0.2971	-0.1836	0.0715
1IPP	C80	1580	3.907	6.777	2.281	-0.2911	-0.2384	0.1084
1IPP	C81	1581	3.711	6.835	2.064	-0.3283	-0.2630	0.1259
1IPP	C82	1582	3.572	7.097	2.032	-0.3745	-0.2844	0.1292
1IPP	C83	1583	3.371	7.153	2.243	-0.3518	-0.3260	0.1570
1IPP	C84	1584	3.102	7.040	2.197	-0.3453	-0.3643	0.1788
1IPP	C85	1585	2.855	7.201	2.171	-0.3628	-0.3933	0.2034
1IPP	C86	1586	2.651	7.177	2.387	-0.3412	-0.4156	0.2289
1IPP	C87	1587	2.624	7.359	2.621	-0.3471	-0.4159	0.2246
1IPP	C88	1588	2.715	7.285	2.894	-0.3154	-0.3995	0.2061
1IPP	C89	1589	2.554	7.101	3.063	-0.2684	-0.4261	0.2343
1IPP	C90	1590	2.692	6.852	3.148	-0.2143	-0.3979	0.2254
1IPP	C91	1591	2.673	6.622	2.960	-0.1950	-0.4088	0.2309
1IPP	C92	1592	2.895	6.597	2.765	-0.2122	-0.3734	0.2128
1IPP	C93	1593	2.897	6.786	2.537	-0.2740	-0.3850	0.2181
1IPP	C94	1594	2.796	6.718	2.266	-0.2851	-0.4101	0.2147
1IPP	C95	1595	2.985	6.564	2.096	-0.2741	-0.3816	0.2072
1IPP	C96	1596	3.263	6.655	2.038	-0.2970	-0.3460	0.1752
1IPP	C97	1597	3.466	6.439	2.011	-0.2800	-0.2926	0.1616
1IPP	C98	1598	3.562	6.273	2.239	-0.2116	-0.2821	0.1601
1IPP	C99	1599	3.777	6.373	2.417	-0.2090	-0.2499	0.1257
1IPP	C100	1600	4.053	6.337	2.312	-0.2252	-0.2065	0.0999
1IPP	C1	1601	4.866	8.672	3.084	-0.5150	-0.0573	-0.0274

1IPP	C2	1602	4.569	8.664	3.107	-0.4974	-0.1111	0.0044
1IPP	C3	1603	4.387	8.893	3.162	-0.5292	-0.1329	0.0064
1IPP	C4	1604	4.327	8.968	3.444	-0.5110	-0.1383	0.0157
1IPP	C5	1605	4.166	8.811	3.639	-0.4854	-0.1639	0.0437
1IPP	C6	1606	3.869	8.801	3.636	-0.4631	-0.2037	0.0617
1IPP	C7	1607	3.699	9.007	3.768	-0.4842	-0.2353	0.0782
1IPP	C8	1608	3.681	9.010	4.066	-0.4686	-0.2364	0.0836
1IPP	C9	1609	3.466	8.881	4.225	-0.4210	-0.2490	0.1070
1IPP	C10	1610	3.485	8.604	4.335	-0.3627	-0.2428	0.1143
1IPP	C11	1611	3.355	8.356	4.232	-0.3490	-0.2792	0.1239
1IPP	C12	1612	3.491	8.147	4.072	-0.3288	-0.2544	0.1069
1IPP	C13	1613	3.515	8.163	3.778	-0.3497	-0.2584	0.1189
1IPP	C14	1614	3.686	8.396	3.727	-0.3978	-0.2334	0.0929
1IPP	C15	1615	3.546	8.603	3.883	-0.4170	-0.2467	0.1079
1IPP	C16	1616	3.269	8.700	3.839	-0.4301	-0.2996	0.1241
1IPP	C17	1617	3.067	8.643	4.051	-0.3983	-0.3304	0.1460
1IPP	C18	1618	2.930	8.380	4.053	-0.3732	-0.3382	0.1670
1IPP	C19	1619	2.674	8.361	3.904	-0.3634	-0.3876	0.2055
1IPP	C20	1620	2.669	8.268	3.621	-0.3869	-0.3934	0.1901
1IPP	C21	1621	2.801	8.451	3.427	-0.4399	-0.3828	0.1846
1IPP	C22	1622	3.067	8.390	3.306	-0.4422	-0.3395	0.1569
1IPP	C23	1623	3.324	8.438	3.449	-0.4306	-0.2964	0.1363
1IPP	C24	1624	3.445	8.709	3.420	-0.4775	-0.2775	0.1144
1IPP	C25	1625	3.603	8.774	3.177	-0.5113	-0.2650	0.0969
1IPP	C26	1626	3.899	8.806	3.179	-0.5119	-0.2075	0.0636
1IPP	C27	1627	4.084	8.590	3.263	-0.4720	-0.1876	0.0506
1IPP	C28	1628	4.219	8.385	3.098	-0.4635	-0.1623	0.0358
1IPP	C29	1629	4.123	8.114	3.161	-0.4128	-0.1671	0.0483
1IPP	C30	1630	4.278	7.944	3.347	-0.3671	-0.1550	0.0475
1IPP	C31	1631	4.444	7.873	3.115	-0.3874	-0.1248	0.0302
1IPP	C32	1632	4.256	7.697	2.967	-0.3579	-0.1667	0.0555
1IPP	C33	1633	4.008	7.806	2.846	-0.4026	-0.1949	0.0748
1IPP	C34	1634	3.766	7.896	2.993	-0.3862	-0.2401	0.0920
1IPP	C35	1635	3.565	7.685	3.048	-0.3633	-0.2688	0.1273
1IPP	C36	1636	3.380	7.662	2.820	-0.3738	-0.3038	0.1402
1IPP	C37	1637	3.129	7.819	2.789	-0.4089	-0.3460	0.1646
1IPP	C38	1638	2.869	7.695	2.857	-0.3779	-0.3735	0.1883
1IPP	C39	1639	2.729	7.720	3.119	-0.3499	-0.3933	0.2019
1IPP	C40	1640	2.825	7.567	3.354	-0.3064	-0.3653	0.2053
1IPP	C41	1641	2.792	7.274	3.395	-0.2504	-0.3878	0.2048
1IPP	C42	1642	2.544	7.192	3.533	-0.2400	-0.4121	0.2390
1IPP	C43	1643	2.570	7.304	3.806	-0.2233	-0.4073	0.2275
1IPP	C44	1644	2.468	7.578	3.858	-0.2655	-0.4249	0.2361
1IPP	C45	1645	2.634	7.822	3.821	-0.2939	-0.3853	0.2130
1IPP	C46	1646	2.803	7.927	4.042	-0.2969	-0.3653	0.1888
1IPP	C47	1647	3.088	7.948	3.962	-0.3039	-0.3236	0.1586
1IPP	C48	1648	3.078	8.108	3.712	-0.3555	-0.3298	0.1696
1IPP	C49	1649	2.987	7.976	3.460	-0.3604	-0.3497	0.1808
1IPP	C50	1650	3.180	7.861	3.266	-0.3653	-0.3305	0.1476
1IPP	C51	1651	3.355	8.071	3.150	-0.4050	-0.2904	0.1191
1IPP	C52	1652	3.310	8.200	2.886	-0.4459	-0.3231	0.1388
1IPP	C53	1653	3.497	8.146	2.660	-0.4650	-0.2817	0.1274
1IPP	C54	1654	3.501	7.918	2.467	-0.4501	-0.2865	0.1179
1IPP	C55	1655	3.727	7.726	2.500	-0.4198	-0.2621	0.0970
1IPP	C56	1656	3.982	7.847	2.404	-0.4348	-0.2138	0.0790
1IPP	C57	1657	4.089	7.822	2.128	-0.4705	-0.2115	0.0618
1IPP	C58	1658	4.278	7.603	2.057	-0.4525	-0.1762	0.0542
1IPP	C59	1659	4.572	7.644	2.026	-0.4507	-0.1421	0.0133
1IPP	C60	1660	4.754	7.647	2.261	-0.4252	-0.0999	0.0052
1IPP	C61	1661	4.849	7.909	2.366	-0.4629	-0.0886	-0.0131
1IPP	C62	1662	5.124	7.915	2.480	-0.4534	-0.0371	-0.0495
1IPP	C63	1663	5.147	7.864	2.772	-0.4111	-0.0387	-0.0403
1IPP	C64	1664	5.108	8.101	2.947	-0.4305	-0.0224	-0.0390
1IPP	C65	1665	4.821	8.159	2.991	-0.4382	-0.0773	-0.0207
1IPP	C66	1666	4.674	8.342	2.809	-0.4839	-0.1129	-0.0098
1IPP	C67	1667	4.513	8.220	2.593	-0.4918	-0.1291	0.0199
1IPP	C68	1668	4.252	8.133	2.704	-0.4541	-0.1675	0.0386
1IPP	C69	1669	3.987	8.255	2.645	-0.4797	-0.2057	0.0663
1IPP	C70	1670	3.877	8.464	2.825	-0.4951	-0.2272	0.0688
1IPP	C71	1671	3.692	8.361	3.032	-0.4646	-0.2467	0.0938
1IPP	C72	1672	3.735	8.290	3.316	-0.4336	-0.2208	0.0902
1IPP	C73	1673	3.786	8.020	3.433	-0.3666	-0.2263	0.0938
1IPP	C74	1674	3.585	7.804	3.465	-0.3378	-0.2588	0.1160

1IPP	C75	1675	3.376	7.762	3.672	-0.3036	-0.2808	0.1380
1IPP	C76	1676	3.445	7.606	3.914	-0.2622	-0.2780	0.1310
1IPP	C77	1677	3.568	7.720	4.158	-0.2490	-0.2397	0.1351
1IPP	C78	1678	3.838	7.845	4.137	-0.2724	-0.1987	0.0906
1IPP	C79	1679	3.912	8.106	4.260	-0.3057	-0.1790	0.0711
1IPP	C80	1680	3.863	8.383	4.165	-0.3536	-0.2043	0.0807
1IPP	C81	1681	4.038	8.506	3.958	-0.3911	-0.1673	0.0506
1IPP	C82	1682	4.335	8.521	3.958	-0.3891	-0.1345	0.0226
1IPP	C83	1683	4.497	8.752	4.051	-0.4189	-0.0910	0.0008
1IPP	C84	1684	4.534	8.970	3.854	-0.4769	-0.1132	-0.0086
1IPP	C85	1685	4.376	9.221	3.858	-0.5123	-0.1166	0.0125
1IPP	C86	1686	4.082	9.238	3.821	-0.5249	-0.1685	0.0248
1IPP	C87	1687	4.006	9.258	3.535	-0.5575	-0.1923	0.0424
1IPP	C88	1688	4.188	9.473	3.439	-0.5934	-0.1540	0.0248
1IPP	C89	1689	4.448	9.397	3.313	-0.5943	-0.1237	-0.0097
1IPP	C90	1690	4.685	9.289	3.458	-0.5643	-0.0815	-0.0310
1IPP	C91	1691	4.770	9.018	3.371	-0.5391	-0.0656	-0.0286
1IPP	C92	1692	4.728	8.762	3.517	-0.4870	-0.0709	-0.0155
1IPP	C93	1693	4.966	8.592	3.560	-0.4494	-0.0363	-0.0401
1IPP	C94	1694	5.033	8.361	3.386	-0.4261	-0.0315	-0.0374
1IPP	C95	1695	4.848	8.137	3.446	-0.3760	-0.0587	-0.0161
1IPP	C96	1696	4.594	8.264	3.355	-0.4295	-0.0971	0.0123
1IPP	C97	1697	4.405	8.405	3.534	-0.4179	-0.1198	0.0128
1IPP	C98	1698	4.136	8.288	3.591	-0.3951	-0.1648	0.0411
1IPP	C99	1699	4.062	8.083	3.795	-0.3436	-0.1703	0.0588
1IPP	C100	1700	4.055	7.792	3.729	-0.2997	-0.1770	0.0711
1IPP	C1	1701	5.202	7.062	4.280	-0.1366	0.0134	-0.0350
1IPP	C2	1702	5.151	6.909	4.529	-0.0859	0.0327	-0.0127
1IPP	C3	1703	5.155	6.621	4.462	-0.0550	0.0028	-0.0147
1IPP	C4	1704	5.368	6.519	4.284	-0.0460	0.0381	-0.0303
1IPP	C5	1705	5.295	6.593	4.006	-0.0938	0.0245	-0.0309
1IPP	C6	1706	5.193	6.407	3.800	-0.0818	0.0099	-0.0165
1IPP	C7	1707	5.431	6.293	3.666	-0.0809	0.0388	-0.0353
1IPP	C8	1708	5.545	6.521	3.515	-0.1179	0.0535	-0.0526
1IPP	C9	1709	5.391	6.575	3.267	-0.1622	0.0249	-0.0265
1IPP	C10	1710	5.497	6.488	3.004	-0.1606	0.0419	-0.0547
1IPP	C11	1711	5.675	6.714	2.934	-0.2219	0.0584	-0.0694
1IPP	C12	1712	5.500	6.954	2.937	-0.2472	0.0282	-0.0586
1IPP	C13	1713	5.354	7.024	2.688	-0.2909	-0.0060	-0.0408
1IPP	C14	1714	5.454	7.239	2.507	-0.3440	0.0254	-0.0676
1IPP	C15	1715	5.372	7.514	2.584	-0.3748	0.0041	-0.0541
1IPP	C16	1716	5.562	7.648	2.770	-0.3790	0.0426	-0.0761
1IPP	C17	1717	5.697	7.458	2.955	-0.3300	0.0675	-0.0869
1IPP	C18	1718	5.541	7.337	3.175	-0.2865	0.0410	-0.0743
1IPP	C19	1719	5.476	7.450	3.442	-0.2804	0.0416	-0.0704
1IPP	C20	1720	5.672	7.399	3.658	-0.2460	0.0828	-0.0888
1IPP	C21	1721	5.785	7.127	3.693	-0.2050	0.0853	-0.0888
1IPP	C22	1722	5.652	6.881	3.791	-0.1546	0.0835	-0.0816
1IPP	C23	1723	5.698	6.757	4.057	-0.0998	0.0840	-0.0628
1IPP	C24	1724	5.884	6.530	4.099	-0.0710	0.1331	-0.0881
1IPP	C25	1725	5.771	6.287	3.972	-0.0363	0.0915	-0.0811
1IPP	C26	1726	5.555	6.145	4.120	-0.0103	0.0628	-0.0341
1IPP	C27	1727	5.608	5.891	4.264	0.0479	0.0741	-0.0482
1IPP	C28	1728	5.537	5.625	4.154	0.0793	0.0676	-0.0219
1IPP	C29	1729	5.272	5.491	4.133	0.1006	0.0225	0.0041
1IPP	C30	1730	5.173	5.314	4.352	0.1421	0.0138	0.0095
1IPP	C31	1731	5.221	5.024	4.317	0.1885	0.0184	0.0190
1IPP	C32	1732	5.502	4.940	4.362	0.2112	0.0645	-0.0111
1IPP	C33	1733	5.686	4.947	4.131	0.1742	0.0909	-0.0252
1IPP	C34	1734	5.746	5.228	4.058	0.1235	0.1007	-0.0428
1IPP	C35	1735	5.640	5.367	3.817	0.0882	0.0673	-0.0345
1IPP	C36	1736	5.830	5.348	3.589	0.0670	0.0989	-0.0526
1IPP	C37	1737	6.088	5.498	3.583	0.0394	0.1432	-0.0788
1IPP	C38	1738	6.135	5.748	3.429	-0.0182	0.1361	-0.0904
1IPP	C39	1739	6.116	6.023	3.541	-0.0564	0.1436	-0.1041
1IPP	C40	1740	5.850	6.153	3.528	-0.0610	0.1003	-0.0766
1IPP	C41	1741	5.820	6.281	3.262	-0.1017	0.0944	-0.0753
1IPP	C42	1742	6.009	6.509	3.267	-0.1585	0.1104	-0.0964
1IPP	C43	1743	5.948	6.748	3.433	-0.1759	0.1084	-0.0915
1IPP	C44	1744	5.800	6.963	3.293	-0.2138	0.0845	-0.0887
1IPP	C45	1745	5.979	7.043	3.072	-0.2519	0.1026	-0.1100
1IPP	C46	1746	6.257	7.073	3.173	-0.2484	0.1517	-0.1443
1IPP	C47	1747	6.452	6.848	3.197	-0.2109	0.1890	-0.1446

```
  1IPP    C48 1748   6.536   6.694   3.439 -0.1601  0.2071 -0.1605
  1IPP    C49 1749   6.418   6.433   3.520 -0.1141  0.1866 -0.1403
  1IPP    C50 1750   6.197   6.438   3.718 -0.0958  0.1573 -0.1115
  1IPP    C51 1751   6.296   6.419   3.997 -0.0674  0.1849 -0.1302
  1IPP    C52 1752   6.339   6.161   4.137 -0.0076  0.1845 -0.1149
  1IPP    C53 1753   6.087   6.020   4.204  0.0259  0.1493 -0.1005
  1IPP    C54 1754   5.947   6.169   4.418  0.0249  0.1404 -0.0902
  1IPP    C55 1755   5.800   6.068   4.655  0.0552  0.1175 -0.0594
  1IPP    C56 1756   5.509   6.123   4.640  0.0507  0.0743 -0.0380
  1IPP    C57 1757   5.372   6.357   4.765  0.0176  0.0544 -0.0294
  1IPP    C58 1758   5.210   6.337   5.014  0.0528  0.0341 -0.0206
  1IPP    C59 1759   4.916   6.381   5.017  0.0372 -0.0079  0.0148
  1IPP    C60 1760   4.793   6.648   5.060  0.0086 -0.0312  0.0197
  1IPP    C61 1761   4.852   6.885   4.893 -0.0554 -0.0235  0.0031
  1IPP    C62 1762   4.634   6.844   4.699 -0.0529 -0.0608  0.0300
  1IPP    C63 1763   4.721   6.703   4.455 -0.0545 -0.0509  0.0317
  1IPP    C64 1764   4.702   6.412   4.511 -0.0105 -0.0505  0.0230
  1IPP    C65 1765   4.949   6.270   4.598  0.0319 -0.0205  0.0176
  1IPP    C66 1766   5.144   6.145   4.411  0.0241  0.0080 -0.0015
  1IPP    C67 1767   5.177   5.853   4.371  0.0609  0.0142  0.0044
  1IPP    C68 1768   5.314   5.682   4.575  0.1203  0.0293 -0.0072
  1IPP    C69 1769   5.604   5.652   4.639  0.1165  0.0871 -0.0339
  1IPP    C70 1770   5.791   5.466   4.500  0.1390  0.1079 -0.0522
  1IPP    C71 1771   5.990   5.576   4.307  0.1036  0.1351 -0.0741
  1IPP    C72 1772   5.977   5.600   4.012  0.0771  0.1313 -0.0739
  1IPP    C73 1773   5.869   5.833   3.865  0.0056  0.1072 -0.0615
  1IPP    C74 1774   5.585   5.855   3.780  0.0078  0.0630 -0.0488
  1IPP    C75 1775   5.509   5.731   3.523 -0.0025  0.0500 -0.0256
  1IPP    C76 1776   5.528   5.896   3.279 -0.0536  0.0423 -0.0347
  1IPP    C77 1777   5.338   6.122   3.243 -0.0854  0.0098 -0.0140
  1IPP    C78 1778   5.044   6.099   3.198 -0.1065 -0.0407  0.0121
  1IPP    C79 1779   4.906   6.322   3.337 -0.1100 -0.0585  0.0140
  1IPP    C80 1780   4.784   6.334   3.608 -0.0880 -0.0679  0.0302
  1IPP    C81 1781   4.497   6.404   3.647 -0.0891 -0.1070  0.0516
  1IPP    C82 1782   4.420   6.676   3.552 -0.1483 -0.1325  0.0639
  1IPP    C83 1783   4.540   6.919   3.669 -0.1761 -0.0990  0.0424
  1IPP    C84 1784   4.761   6.930   3.471 -0.1810 -0.0780  0.0115
  1IPP    C85 1785   4.982   6.733   3.489 -0.1609 -0.0231 -0.0002
  1IPP    C86 1786   5.232   6.823   3.619 -0.1621  0.0073 -0.0345
  1IPP    C87 1787   5.373   7.001   3.432 -0.2111  0.0253 -0.0414
  1IPP    C88 1788   5.143   7.169   3.355 -0.2468 -0.0099 -0.0277
  1IPP    C89 1789   5.027   7.368   3.540 -0.2560 -0.0301 -0.0147
  1IPP    C90 1790   4.802   7.290   3.720 -0.2237 -0.0590  0.0143
  1IPP    C91 1791   4.869   7.150   3.974 -0.1824 -0.0361 -0.0096
  1IPP    C92 1792   4.935   6.863   3.936 -0.1364 -0.0373  0.0059
  1IPP    C93 1793   4.755   6.629   3.961 -0.0969 -0.0659  0.0263
  1IPP    C94 1794   4.858   6.398   4.117 -0.0489 -0.0368  0.0230
  1IPP    C95 1795   4.892   6.112   4.043 -0.0189 -0.0343  0.0161
  1IPP    C96 1796   5.141   5.989   3.936  0.0020 -0.0104  0.0045
  1IPP    C97 1797   5.132   5.958   3.641 -0.0187 -0.0074  0.0012
  1IPP    C98 1798   5.003   5.700   3.575  0.0032 -0.0268  0.0088
  1IPP    C99 1799   5.153   5.458   3.664  0.0585 -0.0174  0.0152
  1IPP   C100 1800   5.332   5.313   3.476  0.0580  0.0173 -0.0009
10.00000  10.00000  10.00000
```

2.2 Polypropylene NP System Topology

```
#include "martini_v2.0_PEO_PS_CNP.itp"
#include "PP100.itp"

[ system ]
; name
 POLYPROPYLENE

[ molecules ]
; name                 number
IPP                     18
```

2.3 Polypropylene NP Topology

```
[moleculetype]
 ; molname     nrexcl
   IPP       2

 [atoms]
 ; id type   resnr residu  atom  cgnr charge
   1  SC1      1     IPP   C1      1   0
   2  SC1      1     IPP   C2      2   0
   3  SC1      1     IPP   C3      3   0
   4  SC1      1     IPP   C4      4   0
   5  SC1      1     IPP   C5      5   0
   6  SC1      1     IPP   C6      6   0
   7  SC1      1     IPP   C7      7   0
   8  SC1      1     IPP   C8      8   0
   9  SC1      1     IPP   C9      9   0
  10  SC1      1     IPP   C10    10   0
  11  SC1      1     IPP   C11    11   0
  12  SC1      1     IPP   C12    12   0
  13  SC1      1     IPP   C13    13   0
  14  SC1      1     IPP   C14    14   0
  15  SC1      1     IPP   C15    15   0
  16  SC1      1     IPP   C16    16   0
  17  SC1      1     IPP   C17    17   0
  18  SC1      1     IPP   C18    18   0
  19  SC1      1     IPP   C19    19   0
  20  SC1      1     IPP   C20    20   0
  21  SC1      1     IPP   C21    21   0
  22  SC1      1     IPP   C22    22   0
  23  SC1      1     IPP   C23    23   0
  24  SC1      1     IPP   C24    24   0
  25  SC1      1     IPP   C25    25   0
  26  SC1      1     IPP   C26    26   0
  27  SC1      1     IPP   C27    27   0
  28  SC1      1     IPP   C28    28   0
  29  SC1      1     IPP   C29    29   0
  30  SC1      1     IPP   C30    30   0
  31  SC1      1     IPP   C31    31   0
  32  SC1      1     IPP   C32    32   0
  33  SC1      1     IPP   C33    33   0
  34  SC1      1     IPP   C34    34   0
  35  SC1      1     IPP   C35    35   0
  36  SC1      1     IPP   C36    36   0
  37  SC1      1     IPP   C37    37   0
  38  SC1      1     IPP   C38    38   0
  39  SC1      1     IPP   C39    39   0
  40  SC1      1     IPP   C40    40   0
  41  SC1      1     IPP   C41    41   0
  42  SC1      1     IPP   C42    42   0
  43  SC1      1     IPP   C43    43   0
  44  SC1      1     IPP   C44    44   0
  45  SC1      1     IPP   C45    45   0
  46  SC1      1     IPP   C46    46   0
  47  SC1      1     IPP   C47    47   0
  48  SC1      1     IPP   C48    48   0
  49  SC1      1     IPP   C49    49   0
  50  SC1      1     IPP   C50    50   0
  51  SC1      1     IPP   C51    51   0
```

```
 52   SC1       1      IPP   C52    52    0
 53   SC1       1      IPP   C53    53    0
 54   SC1       1      IPP   C54    54    0
 55   SC1       1      IPP   C55    55    0
 56   SC1       1      IPP   C56    56    0
 57   SC1       1      IPP   C57    57    0
 58   SC1       1      IPP   C58    58    0
 59   SC1       1      IPP   C59    59    0
 60   SC1       1      IPP   C60    60    0
 61   SC1       1      IPP   C61    61    0
 62   SC1       1      IPP   C62    62    0
 63   SC1       1      IPP   C63    63    0
 64   SC1       1      IPP   C64    64    0
 65   SC1       1      IPP   C65    65    0
 66   SC1       1      IPP   C66    66    0
 67   SC1       1      IPP   C67    67    0
 68   SC1       1      IPP   C68    68    0
 69   SC1       1      IPP   C69    69    0
 70   SC1       1      IPP   C70    70    0
 71   SC1       1      IPP   C71    71    0
 72   SC1       1      IPP   C72    72    0
 73   SC1       1      IPP   C73    73    0
 74   SC1       1      IPP   C74    74    0
 75   SC1       1      IPP   C75    75    0
 76   SC1       1      IPP   C76    76    0
 77   SC1       1      IPP   C77    77    0
 78   SC1       1      IPP   C78    78    0
 79   SC1       1      IPP   C79    79    0
 80   SC1       1      IPP   C80    80    0
 81   SC1       1      IPP   C81    81    0
 82   SC1       1      IPP   C82    82    0
 83   SC1       1      IPP   C83    83    0
 84   SC1       1      IPP   C84    84    0
 85   SC1       1      IPP   C85    85    0
 86   SC1       1      IPP   C86    86    0
 87   SC1       1      IPP   C87    87    0
 88   SC1       1      IPP   C88    88    0
 89   SC1       1      IPP   C89    89    0
 90   SC1       1      IPP   C90    90    0
 91   SC1       1      IPP   C91    91    0
 92   SC1       1      IPP   C92    92    0
 93   SC1       1      IPP   C93    93    0
 94   SC1       1      IPP   C94    94    0
 95   SC1       1      IPP   C95    95    0
 96   SC1       1      IPP   C96    96    0
 97   SC1       1      IPP   C97    97    0
 98   SC1       1      IPP   C98    98    0
 99   SC1       1      IPP   C99    99    0
100   SC1       1      IPP   C100  100    0

[bonds]
;  i    j   funct   length  force.c.
   1    2      1     0.298  48000
   2    3      1     0.298  48000
   3    4      1     0.298  48000
   4    5      1     0.298  48000
   5    6      1     0.298  48000
   6    7      1     0.298  48000
   7    8      1     0.298  48000
   8    9      1     0.298  48000
   9   10      1     0.298  48000
  10   11      1     0.298  48000
  11   12      1     0.298  48000
  12   13      1     0.298  48000
  13   14      1     0.298  48000
  14   15      1     0.298  48000
  15   16      1     0.298  48000
  16   17      1     0.298  48000
  17   18      1     0.298  48000
  18   19      1     0.298  48000
  19   20      1     0.298  48000
  20   21      1     0.298  48000
  21   22      1     0.298  48000
```

22	23	1	0.298	48000
23	24	1	0.298	48000
24	25	1	0.298	48000
25	26	1	0.298	48000
26	27	1	0.298	48000
27	28	1	0.298	48000
28	29	1	0.298	48000
29	30	1	0.298	48000
30	31	1	0.298	48000
31	32	1	0.298	48000
32	33	1	0.298	48000
33	34	1	0.298	48000
34	35	1	0.298	48000
35	36	1	0.298	48000
36	37	1	0.298	48000
37	38	1	0.298	48000
38	39	1	0.298	48000
39	40	1	0.298	48000
40	41	1	0.298	48000
41	42	1	0.298	48000
42	43	1	0.298	48000
43	44	1	0.298	48000
44	45	1	0.298	48000
45	46	1	0.298	48000
46	47	1	0.298	48000
47	48	1	0.298	48000
48	49	1	0.298	48000
49	50	1	0.298	48000
50	51	1	0.298	48000
51	52	1	0.298	48000
52	53	1	0.298	48000
53	54	1	0.298	48000
54	55	1	0.298	48000
55	56	1	0.298	48000
56	57	1	0.298	48000
57	58	1	0.298	48000
58	59	1	0.298	48000
59	60	1	0.298	48000
60	61	1	0.298	48000
61	62	1	0.298	48000
62	63	1	0.298	48000
63	64	1	0.298	48000
64	65	1	0.298	48000
65	66	1	0.298	48000
66	67	1	0.298	48000
67	68	1	0.298	48000
68	69	1	0.298	48000
69	70	1	0.298	48000
70	71	1	0.298	48000
71	72	1	0.298	48000
72	73	1	0.298	48000
73	74	1	0.298	48000
74	75	1	0.298	48000
75	76	1	0.298	48000
76	77	1	0.298	48000
77	78	1	0.298	48000
78	79	1	0.298	48000
79	80	1	0.298	48000
80	81	1	0.298	48000
81	82	1	0.298	48000
82	83	1	0.298	48000
83	84	1	0.298	48000
84	85	1	0.298	48000
85	86	1	0.298	48000
86	87	1	0.298	48000
87	88	1	0.298	48000
88	89	1	0.298	48000
89	90	1	0.298	48000
90	91	1	0.298	48000
91	92	1	0.298	48000
92	93	1	0.298	48000
93	94	1	0.298	48000
94	95	1	0.298	48000

```
   95   96      1     0.298  48000
   96   97      1     0.298  48000
   97   98      1     0.298  48000
   98   99      1     0.298  48000
   99  100      1     0.298  48000

[angles]
;  i    j    k    funct   angle   force.c.
    1    2    3     10       119     78
    2    3    4     10       119     78
    3    4    5     10       119     78
    4    5    6     10       119     78
    5    6    7     10       119     78
    6    7    8     10       119     78
    7    8    9     10       119     78
    8    9   10     10       119     78
    9   10   11     10       119     78
   10   11   12     10       119     78
   11   12   13     10       119     78
   12   13   14     10       119     78
   13   14   15     10       119     78
   14   15   16     10       119     78
   15   16   17     10       119     78
   16   17   18     10       119     78
   17   18   19     10       119     78
   18   19   20     10       119     78
   19   20   21     10       119     78
   20   21   22     10       119     78
   21   22   23     10       119     78
   22   23   24     10       119     78
   23   24   25     10       119     78
   24   25   26     10       119     78
   25   26   27     10       119     78
   26   27   28     10       119     78
   27   28   29     10       119     78
   28   29   30     10       119     78
   29   30   31     10       119     78
   30   31   32     10       119     78
   31   32   33     10       119     78
   32   33   34     10       119     78
   33   34   35     10       119     78
   34   35   36     10       119     78
   35   36   37     10       119     78
   36   37   38     10       119     78
   37   38   39     10       119     78
   38   39   40     10       119     78
   39   40   41     10       119     78
   40   41   42     10       119     78
   41   42   43     10       119     78
   42   43   44     10       119     78
   43   44   45     10       119     78
   44   45   46     10       119     78
   45   46   47     10       119     78
   46   47   48     10       119     78
   47   48   49     10       119     78
   48   49   50     10       119     78
   49   50   51     10       119     78
   50   51   52     10       119     78
   51   52   53     10       119     78
   52   53   54     10       119     78
   53   54   55     10       119     78
   54   55   56     10       119     78
   55   56   57     10       119     78
   56   57   58     10       119     78
   57   58   59     10       119     78
   58   59   60     10       119     78
   59   60   61     10       119     78
   60   61   62     10       119     78
   61   62   63     10       119     78
   62   63   64     10       119     78
   63   64   65     10       119     78
   64   65   66     10       119     78
   65   66   67     10       119     78
```

```
   66   67   68     10       119     78
   67   68   69     10       119     78
   68   69   70     10       119     78
   69   70   71     10       119     78
   70   71   72     10       119     78
   71   72   73     10       119     78
   72   73   74     10       119     78
   73   74   75     10       119     78
   74   75   76     10       119     78
   75   76   77     10       119     78
   76   77   78     10       119     78
   77   78   79     10       119     78
   78   79   80     10       119     78
   79   80   81     10       119     78
   80   81   82     10       119     78
   81   82   83     10       119     78
   82   83   84     10       119     78
   83   84   85     10       119     78
   84   85   86     10       119     78
   85   86   87     10       119     78
   86   87   88     10       119     78
   87   88   89     10       119     78
   88   89   90     10       119     78
   89   90   91     10       119     78
   90   91   92     10       119     78
   91   92   93     10       119     78
   92   93   94     10       119     78
   93   94   95     10       119     78
   94   95   96     10       119     78
   95   96   97     10       119     78
   96   97   98     10       119     78
   97   98   99     10       119     78
   98   99  100     10       119     78

[ dihedrals ]
;  ai  aj  ak  al       funct  phi     k       n
    1    2    3    4      9   100.00  3.10      1
    1    2    3    4      9   190.00 -5.90      2
    2    3    4    5      9   100.00  3.10      1
    2    3    4    5      9   190.00 -5.90      2
    3    4    5    6      9   100.00  3.10      1
    3    4    5    6      9   190.00 -5.90      2
    4    5    6    7      9   100.00  3.10      1
    4    5    6    7      9   190.00 -5.90      2
    5    6    7    8      9   100.00  3.10      1
    5    6    7    8      9   190.00 -5.90      2
    6    7    8    9      9   100.00  3.10      1
    6    7    8    9      9   190.00 -5.90      2
    7    8    9   10      9   100.00  3.10      1
    7    8    9   10      9   190.00 -5.90      2
    8    9   10   11      9   100.00  3.10      1
    8    9   10   11      9   190.00 -5.90      2
    9   10   11   12      9   100.00  3.10      1
    9   10   11   12      9   190.00 -5.90      2
   10   11   12   13      9   100.00  3.10      1
   10   11   12   13      9   190.00 -5.90      2
   11   12   13   14      9   100.00  3.10      1
   11   12   13   14      9   190.00 -5.90      2
   12   13   14   15      9   100.00  3.10      1
   12   13   14   15      9   190.00 -5.90      2
   13   14   15   16      9   100.00  3.10      1
   13   14   15   16      9   190.00 -5.90      2
   14   15   16   17      9   100.00  3.10      1
   14   15   16   17      9   190.00 -5.90      2
   15   16   17   18      9   100.00  3.10      1
   15   16   17   18      9   190.00 -5.90      2
   16   17   18   19      9   100.00  3.10      1
   16   17   18   19      9   190.00 -5.90      2
   17   18   19   20      9   100.00  3.10      1
   17   18   19   20      9   190.00 -5.90      2
   18   19   20   21      9   100.00  3.10      1
   18   19   20   21      9   190.00 -5.90      2
   19   20   21   22      9   100.00  3.10      1
```

19	20	21	22	9	190.00	-5.90	2
20	21	22	23	9	100.00	3.10	1
20	21	22	23	9	190.00	-5.90	2
21	22	23	24	9	100.00	3.10	1
21	22	23	24	9	190.00	-5.90	2
22	23	24	25	9	100.00	3.10	1
22	23	24	25	9	190.00	-5.90	2
23	24	25	26	9	100.00	3.10	1
23	24	25	26	9	190.00	-5.90	2
24	25	26	27	9	100.00	3.10	1
24	25	26	27	9	190.00	-5.90	2
25	26	27	28	9	100.00	3.10	1
25	26	27	28	9	190.00	-5.90	2
26	27	28	29	9	100.00	3.10	1
26	27	28	29	9	190.00	-5.90	2
27	28	29	30	9	100.00	3.10	1
27	28	29	30	9	190.00	-5.90	2
28	29	30	31	9	100.00	3.10	1
28	29	30	31	9	190.00	-5.90	2
29	30	31	32	9	100.00	3.10	1
29	30	31	32	9	190.00	-5.90	2
30	31	32	33	9	100.00	3.10	1
30	31	32	33	9	190.00	-5.90	2
31	32	33	34	9	100.00	3.10	1
31	32	33	34	9	190.00	-5.90	2
32	33	34	35	9	100.00	3.10	1
32	33	34	35	9	190.00	-5.90	2
33	34	35	36	9	100.00	3.10	1
33	34	35	36	9	190.00	-5.90	2
34	35	36	37	9	100.00	3.10	1
34	35	36	37	9	190.00	-5.90	2
35	36	37	38	9	100.00	3.10	1
35	36	37	38	9	190.00	-5.90	2
36	37	38	39	9	100.00	3.10	1
36	37	38	39	9	190.00	-5.90	2
37	38	39	40	9	100.00	3.10	1
37	38	39	40	9	190.00	-5.90	2
38	39	40	41	9	100.00	3.10	1
38	39	40	41	9	190.00	-5.90	2
39	40	41	42	9	100.00	3.10	1
39	40	41	42	9	190.00	-5.90	2
40	41	42	43	9	100.00	3.10	1
40	41	42	43	9	190.00	-5.90	2
41	42	43	44	9	100.00	3.10	1
41	42	43	44	9	190.00	-5.90	2
42	43	44	45	9	100.00	3.10	1
42	43	44	45	9	190.00	-5.90	2
43	44	45	46	9	100.00	3.10	1
43	44	45	46	9	190.00	-5.90	2
44	45	46	47	9	100.00	3.10	1
44	45	46	47	9	190.00	-5.90	2
45	46	47	48	9	100.00	3.10	1
45	46	47	48	9	190.00	-5.90	2
46	47	48	49	9	100.00	3.10	1
46	47	48	49	9	190.00	-5.90	2
47	48	49	50	9	100.00	3.10	1
47	48	49	50	9	190.00	-5.90	2
48	49	50	51	9	100.00	3.10	1
48	49	50	51	9	190.00	-5.90	2
49	50	51	52	9	100.00	3.10	1
49	50	51	52	9	190.00	-5.90	2
50	51	52	53	9	100.00	3.10	1
50	51	52	53	9	190.00	-5.90	2
51	52	53	54	9	100.00	3.10	1
51	52	53	54	9	190.00	-5.90	2
52	53	54	55	9	100.00	3.10	1
52	53	54	55	9	190.00	-5.90	2
53	54	55	56	9	100.00	3.10	1
53	54	55	56	9	190.00	-5.90	2
54	55	56	57	9	100.00	3.10	1
54	55	56	57	9	190.00	-5.90	2
55	56	57	58	9	100.00	3.10	1
55	56	57	58	9	190.00	-5.90	2

56	57	58	59	9	100.00	3.10	1
56	57	58	59	9	190.00	-5.90	2
57	58	59	60	9	100.00	3.10	1
57	58	59	60	9	190.00	-5.90	2
58	59	60	61	9	100.00	3.10	1
58	59	60	61	9	190.00	-5.90	2
59	60	61	62	9	100.00	3.10	1
59	60	61	62	9	190.00	-5.90	2
60	61	62	63	9	100.00	3.10	1
60	61	62	63	9	190.00	-5.90	2
61	62	63	64	9	100.00	3.10	1
61	62	63	64	9	190.00	-5.90	2
62	63	64	65	9	100.00	3.10	1
62	63	64	65	9	190.00	-5.90	2
63	64	65	66	9	100.00	3.10	1
63	64	65	66	9	190.00	-5.90	2
64	65	66	67	9	100.00	3.10	1
64	65	66	67	9	190.00	-5.90	2
65	66	67	68	9	100.00	3.10	1
65	66	67	68	9	190.00	-5.90	2
66	67	68	69	9	100.00	3.10	1
66	67	68	69	9	190.00	-5.90	2
67	68	69	70	9	100.00	3.10	1
67	68	69	70	9	190.00	-5.90	2
68	69	70	71	9	100.00	3.10	1
68	69	70	71	9	190.00	-5.90	2
69	70	71	72	9	100.00	3.10	1
69	70	71	72	9	190.00	-5.90	2
70	71	72	73	9	100.00	3.10	1
70	71	72	73	9	190.00	-5.90	2
71	72	73	74	9	100.00	3.10	1
71	72	73	74	9	190.00	-5.90	2
72	73	74	75	9	100.00	3.10	1
72	73	74	75	9	190.00	-5.90	2
73	74	75	76	9	100.00	3.10	1
73	74	75	76	9	190.00	-5.90	2
74	75	76	77	9	100.00	3.10	1
74	75	76	77	9	190.00	-5.90	2
75	76	77	78	9	100.00	3.10	1
75	76	77	78	9	190.00	-5.90	2
76	77	78	79	9	100.00	3.10	1
76	77	78	79	9	190.00	-5.90	2
77	78	79	80	9	100.00	3.10	1
77	78	79	80	9	190.00	-5.90	2
78	79	80	81	9	100.00	3.10	1
78	79	80	81	9	190.00	-5.90	2
79	80	81	82	9	100.00	3.10	1
79	80	81	82	9	190.00	-5.90	2
80	81	82	83	9	100.00	3.10	1
80	81	82	83	9	190.00	-5.90	2
81	82	83	84	9	100.00	3.10	1
81	82	83	84	9	190.00	-5.90	2
82	83	84	85	9	100.00	3.10	1
82	83	84	85	9	190.00	-5.90	2
83	84	85	86	9	100.00	3.10	1
83	84	85	86	9	190.00	-5.90	2
84	85	86	87	9	100.00	3.10	1
84	85	86	87	9	190.00	-5.90	2
85	86	87	88	9	100.00	3.10	1
85	86	87	88	9	190.00	-5.90	2
86	87	88	89	9	100.00	3.10	1
86	87	88	89	9	190.00	-5.90	2
87	88	89	90	9	100.00	3.10	1
87	88	89	90	9	190.00	-5.90	2
88	89	90	91	9	100.00	3.10	1
88	89	90	91	9	190.00	-5.90	2
89	90	91	92	9	100.00	3.10	1
89	90	91	92	9	190.00	-5.90	2
90	91	92	93	9	100.00	3.10	1
90	91	92	93	9	190.00	-5.90	2
91	92	93	94	9	100.00	3.10	1
91	92	93	94	9	190.00	-5.90	2
92	93	94	95	9	100.00	3.10	1

92	93	94	95	9	190.00	-5.90	2
93	94	95	96	9	100.00	3.10	1
93	94	95	96	9	190.00	-5.90	2
94	95	96	97	9	100.00	3.10	1
94	95	96	97	9	190.00	-5.90	2
95	96	97	98	9	100.00	3.10	1
95	96	97	98	9	190.00	-5.90	2
96	97	98	99	9	100.00	3.10	1
96	97	98	99	9	190.00	-5.90	2
97	98	99	100	9	100.00	3.10	1
97	98	99	100	9	190.00	-5.90	2

3.1 Polyethylene NP Coordinates

```
POLYETHYLENE
 1120
    1PE      C1    1  11.451  17.082  11.380  0.2420 -0.2185  0.0258
    1PE      C2    2  11.751  16.942  11.101  0.2223 -0.1860 -0.0309
    1PE      C3    3  12.118  17.068  10.967  0.2001 -0.1806 -0.0543
    1PE      C4    4  12.506  17.056  11.107  0.2139 -0.2076 -0.0937
    1PE      C5    5  12.630  16.877  11.491  0.2467 -0.2154 -0.1175
    1PE      C6    6  12.421  16.937  11.882  0.2995 -0.2677 -0.1069
    1PE      C7    7  12.052  17.150  12.014  0.3056 -0.2735 -0.0295
    1PE      C8    8  11.659  17.207  12.231  0.3238 -0.3044  0.0089
    1PE      C9    9  11.453  17.509  12.470  0.3362 -0.3343  0.0532
    1PE     C10   10  11.274  17.916  12.383  0.3234 -0.3278  0.1042
    1PE     C11   11  10.996  18.103  12.065  0.3031 -0.2969  0.1543
    1PE     C12   12  10.679  18.096  11.733  0.2723 -0.2867  0.2003
    1PE     C13   13  10.365  18.083  11.423  0.2281 -0.2379  0.2079
    1PE     C14   14   9.939  18.190  11.366  0.2265 -0.2499  0.2689
    1PE     C15   15   9.593  18.511  11.279  0.2237 -0.2352  0.3364
    1PE     C16   16   9.623  18.960  11.240  0.2110 -0.2440  0.3891
    1PE     C17   17  10.011  19.191  11.172  0.1958 -0.2174  0.3574
    1PE     C18   18  10.394  19.000  11.044  0.1861 -0.2100  0.2924
    1PE     C19   19  10.581  18.596  11.128  0.1953 -0.2302  0.2421
    1PE     C20   20  10.830  18.239  11.231  0.2146 -0.2034  0.1922
    1PE     C21   21  11.150  17.951  11.085  0.2003 -0.2046  0.1350
    1PE     C22   22  11.519  17.713  10.974  0.2040 -0.1923  0.0666
    1PE     C23   23  11.775  17.436  11.195  0.2004 -0.2131  0.0139
    1PE     C24   24  11.758  17.378  11.649  0.2589 -0.2585  0.0234
    1PE     C25   25  11.597  17.646  11.970  0.3009 -0.2887  0.0483
    1PE     C26   26  11.517  18.073  11.971  0.2989 -0.2699  0.1092
    1PE     C27   27  11.362  18.483  12.032  0.2799 -0.2937  0.1534
    1PE     C28   28  11.056  18.812  12.043  0.2944 -0.2907  0.2212
    1PE     C29   29  10.626  18.907  12.112  0.2947 -0.3045  0.2694
    1PE     C30   30  10.285  18.723  11.916  0.2885 -0.2966  0.2838
    1PE     C31   31  10.263  18.576  11.515  0.2427 -0.2470  0.2822
    1PE     C32   32  10.066  18.644  11.161  0.2151 -0.2159  0.3097
    1PE     C33   33   9.894  18.844  10.824  0.1567 -0.1807  0.3343
    1PE     C34   34  10.014  19.235  10.648  0.1537 -0.1643  0.3558
    1PE     C35   35  10.318  19.499  10.855  0.1543 -0.1944  0.3578
    1PE     C36   36  10.446  19.439  11.298  0.2115 -0.2324  0.3431
    1PE     C37   37  10.453  19.069  11.560  0.2382 -0.2629  0.3076
    1PE     C38   38  10.723  18.714  11.639  0.2568 -0.2534  0.2468
    1PE     C39   39  11.032  18.430  11.661  0.2644 -0.2592  0.1925
    1PE     C40   40  11.235  18.073  11.552  0.2458 -0.2462  0.1367
    1PE     C41   41  11.669  17.889  11.526  0.2593 -0.2393  0.0697
    1PE     C42   42  12.016  17.864  11.263  0.2136 -0.2034  0.0294
    1PE     C43   43  12.381  18.027  11.056  0.2002 -0.1820  0.0178
    1PE     C44   44  12.417  18.327  10.740  0.1677 -0.1655  0.0484
    1PE     C45   45  12.059  18.456  10.521  0.1424 -0.1514  0.0862
    1PE     C46   46  11.666  18.476  10.726  0.1620 -0.1711  0.1299
    1PE     C47   47  11.565  18.689  11.108  0.2068 -0.1934  0.1670
    1PE     C48   48  11.847  18.838  11.411  0.2268 -0.2364  0.1388
    1PE     C49   49  12.284  18.748  11.435  0.2260 -0.2313  0.0931
    1PE     C50   50  12.507  18.354  11.420  0.2265 -0.2240  0.0288
    1PE     C51   51  12.409  17.929  11.566  0.2483 -0.2435 -0.0045
    1PE     C52   52  12.109  17.694  11.820  0.2830 -0.2521  0.0137
    1PE     C53   53  11.997  17.608  12.259  0.3128 -0.3116  0.0072
    1PE     C54   54  11.779  17.906  12.518  0.3553 -0.3322  0.0715
    1PE     C55   55  11.724  18.325  12.364  0.3285 -0.3210  0.1114
    1PE     C56   56  11.873  18.459  11.942  0.2927 -0.2774  0.1072
    1PE     C57   57  12.013  18.290  11.542  0.2389 -0.2351  0.0741
    1PE     C58   58  12.048  18.419  11.111  0.2050 -0.1882  0.0859
    1PE     C59   59  12.015  18.820  10.903  0.1738 -0.1727  0.1270
    1PE     C60   60  11.709  19.162  10.984  0.1705 -0.1796  0.1872
    1PE     C61   61  11.364  19.140  11.289  0.2204 -0.2299  0.2196
    1PE     C62   62  11.363  18.831  11.615  0.2528 -0.2515  0.1812
    1PE     C63   63  11.544  18.438  11.542  0.2475 -0.2614  0.1461
    1PE     C64   64  11.596  18.194  11.148  0.2024 -0.1939  0.1048
    1PE     C65   65  11.874  18.037  10.815  0.1732 -0.1825  0.0585
    1PE     C66   66  12.182  17.896  10.548  0.1518 -0.1461  0.0163
    1PE     C67   67  12.429  17.616  10.292  0.1330 -0.1204 -0.0261
    1PE     C68   68  12.760  17.354  10.158  0.0910 -0.1010 -0.0797
```

1PE	C69	69	12.919	16.952	10.274	0.1330	-0.1068	-0.1369
1PE	C70	70	12.750	16.643	10.558	0.1501	-0.1321	-0.1547
1PE	C71	71	12.381	16.655	10.802	0.1826	-0.1606	-0.1127
1PE	C72	72	11.961	16.551	10.863	0.1882	-0.1760	-0.0938
1PE	C73	73	11.737	16.294	11.119	0.2381	-0.1956	-0.0900
1PE	C74	74	11.638	15.873	11.123	0.2165	-0.2018	-0.1279
1PE	C75	75	11.428	15.606	10.828	0.1940	-0.1741	-0.1236
1PE	C76	76	11.184	15.395	10.497	0.1620	-0.1545	-0.1066
1PE	C77	77	10.888	15.368	10.133	0.1388	-0.0974	-0.0775
1PE	C78	78	10.754	15.338	9.681	0.1010	-0.0714	-0.0796
1PE	C79	79	10.680	15.338	9.222	0.0287	-0.0328	-0.0814
1PE	C80	80	10.435	15.313	8.830	0.0164	0.0017	-0.0432
1PE	C1	81	11.324	18.517	10.374	0.1132	-0.1294	0.1531
1PE	C2	82	10.959	18.333	10.441	0.1376	-0.1490	0.1904
1PE	C3	83	10.637	18.265	10.728	0.1617	-0.1753	0.2097
1PE	C4	84	10.248	18.219	10.935	0.1675	-0.1936	0.2466
1PE	C5	85	9.820	18.336	10.848	0.1701	-0.1898	0.2947
1PE	C6	86	9.616	18.227	10.464	0.1376	-0.1685	0.3075
1PE	C7	87	9.886	18.020	10.113	0.1071	-0.1343	0.2572
1PE	C8	88	10.345	18.035	10.037	0.0990	-0.1144	0.2242
1PE	C9	89	10.754	17.933	10.227	0.1243	-0.1323	0.1793
1PE	C10	90	10.892	17.851	10.628	0.1468	-0.1560	0.1449
1PE	C11	91	10.678	17.819	11.015	0.1952	-0.2019	0.1669
1PE	C12	92	10.783	17.727	11.434	0.2329	-0.2544	0.1579
1PE	C13	93	10.688	17.496	11.812	0.2839	-0.2781	0.1305
1PE	C14	94	10.810	17.092	11.975	0.3139	-0.2776	0.0767
1PE	C15	95	10.834	16.645	12.042	0.3168	-0.3000	0.0419
1PE	C16	96	10.887	16.311	11.742	0.2763	-0.2880	0.0009
1PE	C17	97	10.950	16.398	11.288	0.2222	-0.2106	0.0024
1PE	C18	98	11.345	16.614	11.172	0.2332	-0.1988	-0.0034
1PE	C19	99	11.691	16.651	11.476	0.2654	-0.2307	-0.0573
1PE	C20	100	11.665	16.917	11.810	0.2858	-0.2685	-0.0169
1PE	C21	101	11.308	17.216	11.852	0.2819	-0.2604	0.0424
1PE	C22	102	10.991	17.227	11.529	0.2592	-0.2497	0.0753
1PE	C23	103	11.023	16.992	11.170	0.2123	-0.2090	0.0576
1PE	C24	104	11.320	17.016	10.847	0.1892	-0.1626	0.0076
1PE	C25	105	11.675	17.243	10.719	0.1781	-0.1538	0.0180
1PE	C26	106	12.067	17.414	10.621	0.1598	-0.1596	-0.0165
1PE	C27	107	12.335	17.547	10.945	0.1914	-0.1738	-0.0196
1PE	C28	108	12.250	17.427	11.391	0.2219	-0.2268	-0.0213
1PE	C29	109	12.084	17.025	11.495	0.2506	-0.2343	-0.0320
1PE	C30	110	12.182	16.653	11.308	0.2352	-0.2162	-0.0923
1PE	C31	111	12.290	16.264	11.149	0.2055	-0.1994	-0.1542
1PE	C32	112	12.170	15.850	11.299	0.2445	-0.2078	-0.1767
1PE	C33	113	12.120	15.785	11.776	0.2781	-0.2493	-0.1526
1PE	C34	114	11.910	16.118	12.062	0.3380	-0.2864	-0.1283
1PE	C35	115	11.578	16.390	11.896	0.3070	-0.2779	-0.0389
1PE	C36	116	11.375	16.273	11.518	0.2576	-0.2516	-0.0553
1PE	C37	117	11.114	15.942	11.419	0.2536	-0.2395	-0.0608
1PE	C38	118	10.654	15.948	11.484	0.2605	-0.2521	-0.0047
1PE	C39	119	10.379	16.313	11.606	0.2747	-0.2602	0.0632
1PE	C40	120	10.293	16.679	11.362	0.2413	-0.2319	0.0965
1PE	C41	121	10.587	16.834	11.060	0.2171	-0.1973	0.0866
1PE	C42	122	10.954	16.621	10.842	0.1982	-0.1754	0.0164
1PE	C43	123	10.890	16.264	10.603	0.1685	-0.1666	-0.0046
1PE	C44	124	10.471	16.120	10.533	0.1751	-0.1620	0.0171
1PE	C45	125	10.114	16.371	10.642	0.1729	-0.1762	0.0989
1PE	C46	126	10.102	16.760	10.889	0.1836	-0.1991	0.1233
1PE	C47	127	10.236	17.170	11.057	0.2184	-0.2140	0.1576
1PE	C48	128	10.642	17.336	11.120	0.2138	-0.2106	0.1259
1PE	C49	129	11.061	17.460	11.056	0.1964	-0.2024	0.1006
1PE	C50	130	11.311	17.577	11.409	0.2204	-0.2350	0.0692
1PE	C51	131	11.140	17.685	11.812	0.2932	-0.2658	0.1085
1PE	C52	132	11.031	17.517	12.183	0.3187	-0.3160	0.0876
1PE	C53	133	11.130	17.124	12.360	0.3490	-0.3220	0.0481
1PE	C54	134	11.311	16.788	12.121	0.3113	-0.3022	0.0094
1PE	C55	135	11.195	16.721	11.684	0.2764	-0.2680	0.0312
1PE	C56	136	10.772	16.779	11.549	0.2681	-0.2535	0.0657
1PE	C57	137	10.500	17.105	11.545	0.2598	-0.2473	0.1173
1PE	C58	138	10.080	17.143	11.548	0.2663	-0.2610	0.1598
1PE	C59	139	9.811	16.960	11.256	0.2264	-0.2288	0.1626
1PE	C60	140	9.781	16.507	11.162	0.2125	-0.2217	0.1241
1PE	C61	141	10.152	16.230	11.149	0.2294	-0.2195	0.0653

1PE	C62	142	10.530	16.354	10.971	0.2109	-0.1967	0.0376
1PE	C63	143	10.527	16.653	10.594	0.1711	-0.1561	0.0667
1PE	C64	144	10.166	16.818	10.392	0.1225	-0.1478	0.1292
1PE	C65	145	9.845	16.643	10.192	0.1240	-0.1333	0.1407
1PE	C66	146	9.803	16.215	10.168	0.1311	-0.1292	0.1021
1PE	C67	147	10.012	15.910	10.388	0.1578	-0.1512	0.0404
1PE	C68	148	10.148	15.845	10.801	0.1825	-0.1711	0.0170
1PE	C69	149	10.531	15.845	11.004	0.2109	-0.1996	-0.0102
1PE	C70	150	10.962	15.881	10.943	0.1959	-0.1963	-0.0317
1PE	C71	151	11.288	16.161	10.905	0.1967	-0.1808	-0.0483
1PE	C72	152	11.428	16.510	10.692	0.1834	-0.1688	-0.0370
1PE	C73	153	11.681	16.769	10.491	0.1506	-0.1312	-0.0283
1PE	C74	154	12.080	16.912	10.501	0.1483	-0.1365	-0.0586
1PE	C75	155	12.486	17.098	10.548	0.1618	-0.1446	-0.0848
1PE	C76	156	12.741	17.436	10.672	0.1744	-0.1505	-0.0967
1PE	C77	157	12.702	17.893	10.640	0.1524	-0.1514	-0.0377
1PE	C78	158	12.540	18.144	10.275	0.1299	-0.1219	0.0070
1PE	C79	159	12.191	18.016	10.036	0.0917	-0.0922	0.0443
1PE	C80	160	11.962	17.649	10.088	0.1016	-0.0929	0.0205
1PE	C1	161	9.508	17.962	8.819	-0.0405	-0.0101	0.2870
1PE	C2	162	9.421	17.863	9.252	0.0135	-0.0366	0.2947
1PE	C3	163	9.376	17.569	9.607	0.0450	-0.0819	0.2787
1PE	C4	164	9.362	17.342	10.020	0.1059	-0.1266	0.2554
1PE	C5	165	9.449	17.020	10.346	0.1349	-0.1517	0.2178
1PE	C6	166	9.773	17.028	10.631	0.1670	-0.1767	0.1889
1PE	C7	167	10.145	17.281	10.579	0.1621	-0.1663	0.1671
1PE	C8	168	10.485	17.156	10.253	0.1333	-0.1202	0.1345
1PE	C9	169	10.378	16.846	9.919	0.0978	-0.1065	0.1014
1PE	C10	170	9.948	16.839	9.716	0.0709	-0.0699	0.1451
1PE	C11	171	9.725	17.216	9.626	0.0710	-0.0802	0.1896
1PE	C12	172	9.785	17.585	9.903	0.0836	-0.1083	0.2357
1PE	C13	173	10.162	17.598	10.175	0.1174	-0.1414	0.1976
1PE	C14	174	10.568	17.555	9.984	0.0855	-0.1259	0.1368
1PE	C15	175	10.688	17.316	9.638	0.0685	-0.0571	0.1236
1PE	C16	176	10.777	16.877	9.538	0.0526	-0.0561	0.0652
1PE	C17	177	10.652	16.480	9.686	0.0886	-0.0699	0.0239
1PE	C18	178	10.535	16.096	9.724	0.0860	-0.0830	0.0052
1PE	C19	179	10.472	15.724	9.509	0.0680	-0.0660	-0.0146
1PE	C20	180	10.133	15.619	9.217	0.0343	-0.0245	0.0281
1PE	C21	181	9.763	15.874	9.095	0.0117	-0.0263	0.0678
1PE	C22	182	9.699	16.278	8.873	-0.0011	-0.0164	0.1163
1PE	C23	183	10.014	16.579	8.796	-0.0121	0.0124	0.1151
1PE	C24	184	10.437	16.490	8.890	0.0043	-0.0080	0.0572
1PE	C25	185	10.532	16.229	9.217	0.0306	-0.0249	0.0337
1PE	C26	186	10.149	16.107	9.382	0.0562	-0.0540	0.0609
1PE	C27	187	9.808	16.441	9.342	0.0352	-0.0427	0.1145
1PE	C28	188	9.897	16.895	9.183	0.0233	-0.0259	0.1582
1PE	C29	189	10.287	17.016	8.968	-0.0031	-0.0030	0.1397
1PE	C30	190	10.716	16.861	9.012	-0.0015	-0.0141	0.0732
1PE	C31	191	10.933	16.463	8.899	-0.0019	0.0081	0.0114
1PE	C32	192	10.997	16.029	9.012	0.0006	0.0002	-0.0236
1PE	C33	193	10.984	15.751	9.385	0.0399	-0.0503	-0.0692
1PE	C34	194	10.891	15.788	9.841	0.1009	-0.0883	-0.0339
1PE	C35	195	10.884	16.125	10.113	0.1037	-0.1201	-0.0097
1PE	C36	196	11.240	16.366	10.243	0.1387	-0.1233	-0.0218
1PE	C37	197	11.629	16.441	10.051	0.1225	-0.1145	-0.0592
1PE	C38	198	11.981	16.716	10.038	0.1078	-0.0888	-0.0764
1PE	C39	199	12.409	16.724	10.185	0.1248	-0.1077	-0.1169
1PE	C40	200	12.778	16.542	10.047	0.1139	-0.0892	-0.1733
1PE	C41	201	13.026	16.623	9.664	0.0687	-0.0571	-0.1830
1PE	C42	202	12.896	16.907	9.326	0.0263	-0.0186	-0.1495
1PE	C43	203	12.469	17.048	9.281	0.0355	-0.0131	-0.0857
1PE	C44	204	12.117	17.135	9.539	0.0625	-0.0332	-0.0439
1PE	C45	205	11.736	17.079	9.764	0.0716	-0.0916	-0.0024
1PE	C46	206	11.431	16.912	10.064	0.0991	-0.1123	0.0046
1PE	C47	207	11.143	16.861	10.411	0.1331	-0.1388	0.0185
1PE	C48	208	10.834	17.087	10.672	0.1448	-0.1691	0.0890
1PE	C49	209	10.594	17.463	10.649	0.1729	-0.1690	0.1447
1PE	C50	210	10.366	17.838	10.584	0.1573	-0.1592	0.2069
1PE	C51	211	9.927	17.842	10.575	0.1536	-0.1698	0.2443
1PE	C52	212	9.697	17.469	10.407	0.1368	-0.1560	0.2245
1PE	C53	213	9.905	17.166	10.129	0.1013	-0.1176	0.1688
1PE	C54	214	10.191	17.279	9.788	0.0729	-0.0895	0.1628

1PE	C55	215	10.330	17.046	9.446	0.0514	-0.0627	0.1313
1PE	C56	216	10.270	16.618	9.403	0.0339	-0.0554	0.0914
1PE	C57	217	10.124	16.382	9.789	0.0865	-0.0811	0.0726
1PE	C58	218	10.356	16.407	10.155	0.1197	-0.1300	0.0723
1PE	C59	219	10.774	16.617	10.157	0.1112	-0.1294	0.0410
1PE	C60	220	11.104	16.622	9.835	0.0971	-0.0760	0.0028
1PE	C61	221	11.483	16.643	9.602	0.0731	-0.0571	-0.0401
1PE	C62	222	11.919	16.668	9.544	0.0498	-0.0454	-0.0660
1PE	C63	223	12.353	16.729	9.684	0.0750	-0.0520	-0.1247
1PE	C64	224	12.681	16.978	9.814	0.0846	-0.0740	-0.1133
1PE	C65	225	12.861	17.306	9.620	0.0635	-0.0480	-0.1094
1PE	C66	226	12.666	17.509	9.303	0.0231	-0.0254	-0.0559
1PE	C67	227	12.246	17.532	9.169	0.0145	-0.0192	-0.0425
1PE	C68	228	11.847	17.326	9.174	0.0187	-0.0247	-0.0013
1PE	C69	229	11.514	17.035	9.209	0.0167	-0.0275	0.0129
1PE	C70	230	11.174	16.758	9.223	0.0149	-0.0328	0.0120
1PE	C71	231	11.057	16.375	9.388	0.0445	-0.0428	-0.0078
1PE	C72	232	11.231	16.107	9.671	0.0837	-0.0531	-0.0547
1PE	C73	233	11.404	15.938	10.029	0.1181	-0.1104	-0.0697
1PE	C74	234	11.208	15.888	10.442	0.1580	-0.1469	-0.0816
1PE	C75	235	10.789	15.751	10.469	0.1528	-0.1565	-0.0397
1PE	C76	236	10.469	15.789	10.140	0.1312	-0.1180	-0.0084
1PE	C77	237	10.106	15.834	9.857	0.1027	-0.0903	0.0368
1PE	C78	238	9.752	16.051	9.695	0.0850	-0.0724	0.0803
1PE	C79	239	9.553	16.446	9.777	0.0865	-0.1010	0.1387
1PE	C80	240	9.465	16.864	9.873	0.0898	-0.0948	0.2045
1PE	C1	241	11.307	17.128	12.840	0.3751	-0.3729	0.0444
1PE	C2	242	11.537	16.842	12.573	0.3621	-0.3511	-0.0070
1PE	C3	243	11.830	16.698	12.240	0.3269	-0.3006	-0.0500
1PE	C4	244	12.047	16.587	11.842	0.2986	-0.2760	-0.0772
1PE	C5	245	11.969	16.232	11.562	0.2621	-0.2452	-0.1232
1PE	C6	246	11.651	15.898	11.648	0.2600	-0.2432	-0.1058
1PE	C7	247	11.281	15.973	11.926	0.3124	-0.2733	-0.0712
1PE	C8	248	11.175	16.313	12.227	0.3285	-0.3092	-0.0283
1PE	C9	249	11.034	16.657	12.531	0.3549	-0.3448	0.0127
1PE	C10	250	10.806	16.997	12.759	0.3737	-0.3488	0.0734
1PE	C11	251	10.362	17.116	12.778	0.3702	-0.3729	0.1258
1PE	C12	252	10.041	17.102	12.459	0.3297	-0.3442	0.1600
1PE	C13	253	9.891	16.934	12.066	0.3187	-0.2993	0.1602
1PE	C14	254	9.884	16.678	11.697	0.2775	-0.2865	0.1401
1PE	C15	255	9.854	16.251	11.589	0.2688	-0.2676	0.1028
1PE	C16	256	9.723	15.985	11.233	0.2426	-0.2222	0.0828
1PE	C17	257	9.708	16.113	10.783	0.1881	-0.1890	0.0862
1PE	C18	258	9.626	16.547	10.657	0.1818	-0.1813	0.1599
1PE	C19	259	9.428	16.832	10.963	0.1927	-0.2020	0.1872
1PE	C20	260	9.380	16.742	11.424	0.2366	-0.2606	0.1972
1PE	C21	261	9.405	16.699	11.885	0.2960	-0.2909	0.1865
1PE	C22	262	9.690	16.478	12.165	0.3134	-0.3254	0.1232
1PE	C23	263	10.138	16.445	12.077	0.3185	-0.3034	0.0952
1PE	C24	264	10.356	16.783	11.894	0.2927	-0.2832	0.0906
1PE	C25	265	10.306	17.232	12.004	0.2867	-0.2948	0.1318
1PE	C26	266	10.152	17.558	12.285	0.3127	-0.3189	0.2071
1PE	C27	267	10.092	17.975	12.457	0.3311	-0.3411	0.2473
1PE	C28	268	9.804	18.248	12.682	0.3399	-0.3720	0.2974
1PE	C29	269	9.381	18.350	12.524	0.3431	-0.3637	0.3536
1PE	C30	270	9.221	18.082	12.176	0.3093	-0.3303	0.3334
1PE	C31	271	9.364	17.659	12.145	0.3084	-0.3275	0.2947
1PE	C32	272	9.662	17.456	12.431	0.3331	-0.3526	0.2174
1PE	C33	273	9.955	17.567	12.765	0.3706	-0.3720	0.2257
1PE	C34	274	10.402	17.618	12.714	0.3734	-0.3595	0.1718
1PE	C35	275	10.613	17.356	12.399	0.3352	-0.3383	0.1347
1PE	C36	276	10.537	16.916	12.345	0.3329	-0.3278	0.0952
1PE	C37	277	10.291	16.632	12.553	0.3649	-0.3630	0.1016
1PE	C38	278	9.855	16.651	12.629	0.3624	-0.3649	0.1391
1PE	C39	279	9.546	16.926	12.465	0.3519	-0.3514	0.2067
1PE	C40	280	9.391	17.152	12.125	0.3046	-0.3281	0.2350
1PE	C41	281	9.115	17.311	11.806	0.2814	-0.2909	0.2733
1PE	C42	282	8.910	17.443	11.420	0.2350	-0.2635	0.2999
1PE	C43	283	8.949	17.328	10.976	0.1904	-0.2223	0.2806
1PE	C44	284	9.351	17.306	10.760	0.1792	-0.1914	0.2506
1PE	C45	285	9.761	17.396	10.961	0.2006	-0.2032	0.2227
1PE	C46	286	9.801	17.543	11.392	0.2375	-0.2550	0.2232
1PE	C47	287	9.476	17.621	11.661	0.2619	-0.2819	0.2770

1PE	C48	288	9.108	17.852	11.691	0.2656	-0.2816	0.3261
1PE	C49	289	9.091	18.308	11.709	0.2528	-0.2702	0.3782
1PE	C50	290	9.375	18.522	11.988	0.2792	-0.3205	0.3556
1PE	C51	291	9.744	18.341	12.184	0.3059	-0.3183	0.3022
1PE	C52	292	10.113	18.486	12.331	0.3356	-0.3388	0.2773
1PE	C53	293	10.202	18.938	12.373	0.3214	-0.3294	0.3210
1PE	C54	294	10.144	19.200	11.992	0.2680	-0.2914	0.3658
1PE	C55	295	9.952	18.975	11.639	0.2358	-0.2611	0.3419
1PE	C56	296	9.826	18.540	11.727	0.2534	-0.2581	0.3413
1PE	C57	297	10.164	18.232	11.894	0.2780	-0.2790	0.2399
1PE	C58	298	10.567	18.383	12.136	0.2963	-0.2972	0.2350
1PE	C59	299	10.556	18.592	12.561	0.3451	-0.3484	0.2468
1PE	C60	300	10.147	18.654	12.804	0.3637	-0.3784	0.3003
1PE	C61	301	9.759	18.750	12.564	0.3476	-0.3575	0.3449
1PE	C62	302	9.735	18.865	12.123	0.3048	-0.3085	0.3584
1PE	C63	303	9.472	18.954	11.750	0.2574	-0.2750	0.3883
1PE	C64	304	9.183	18.793	11.450	0.2238	-0.2484	0.4149
1PE	C65	305	9.033	18.451	11.215	0.1980	-0.2302	0.3869
1PE	C66	306	8.993	18.007	11.220	0.2259	-0.2484	0.3568
1PE	C67	307	9.279	17.656	11.143	0.2079	-0.2256	0.2954
1PE	C68	308	9.384	17.252	11.290	0.2354	-0.2198	0.2403
1PE	C69	309	9.594	17.134	11.672	0.2703	-0.2663	0.2230
1PE	C70	310	9.835	17.406	11.941	0.2969	-0.2964	0.2261
1PE	C71	311	9.810	17.849	12.021	0.2976	-0.3085	0.2494
1PE	C72	312	9.570	18.108	11.778	0.2720	-0.2808	0.2974
1PE	C73	313	9.467	18.042	11.381	0.2363	-0.2351	0.3078
1PE	C74	314	9.733	17.886	11.031	0.2074	-0.2108	0.2679
1PE	C75	315	10.157	17.714	11.043	0.2029	-0.2070	0.1971
1PE	C76	316	10.310	17.571	11.415	0.2238	-0.2429	0.1811
1PE	C77	317	10.251	17.726	11.807	0.2811	-0.2965	0.2063
1PE	C78	318	10.491	17.888	12.145	0.2969	-0.3061	0.2040
1PE	C79	319	10.775	17.847	12.468	0.3356	-0.3357	0.1601
1PE	C80	320	10.990	17.526	12.707	0.3800	-0.3562	0.0963
1PE	C1	321	13.161	14.418	7.291	-0.1688	0.1915	-0.4191
1PE	C2	322	13.535	14.692	7.240	-0.1486	0.1882	-0.4279
1PE	C3	323	13.680	15.107	7.093	-0.1516	0.2051	-0.3888
1PE	C4	324	13.504	15.466	6.915	-0.1934	0.2010	-0.3399
1PE	C5	325	13.210	15.776	7.064	-0.1768	0.2002	-0.2811
1PE	C6	326	13.040	15.763	7.460	-0.1312	0.1468	-0.2893
1PE	C7	327	13.157	15.747	7.856	-0.0938	0.1325	-0.2747
1PE	C8	328	13.068	15.615	8.251	-0.0607	0.0840	-0.2906
1PE	C9	329	12.873	15.258	8.414	-0.0427	0.0743	-0.2935
1PE	C10	330	12.774	14.805	8.485	-0.0208	0.0574	-0.3391
1PE	C11	331	12.851	14.366	8.596	-0.0084	0.0550	-0.3768
1PE	C12	332	12.874	14.184	9.019	0.0376	0.0103	-0.3917
1PE	C13	333	12.946	14.452	9.386	0.0486	-0.0197	-0.3807
1PE	C14	334	13.049	14.888	9.367	0.0604	-0.0355	-0.3432
1PE	C15	335	12.915	15.298	9.316	0.0651	-0.0134	-0.3147
1PE	C16	336	12.648	15.562	9.071	0.0347	-0.0034	-0.2503
1PE	C17	337	12.225	15.668	9.004	0.0264	0.0014	-0.1982
1PE	C18	338	11.810	15.652	9.179	0.0371	-0.0133	-0.1473
1PE	C19	339	11.518	15.675	9.523	0.0749	-0.0582	-0.1177
1PE	C20	340	11.262	15.461	9.800	0.0964	-0.0913	-0.1247
1PE	C21	341	11.355	15.137	10.100	0.1313	-0.1207	-0.1626
1PE	C22	342	11.695	15.138	10.410	0.1639	-0.1330	-0.1795
1PE	C23	343	12.099	15.336	10.341	0.1537	-0.1317	-0.2164
1PE	C24	344	12.216	15.485	9.952	0.1134	-0.0932	-0.2070
1PE	C25	345	12.024	15.291	9.558	0.0768	-0.0562	-0.2080
1PE	C26	346	12.018	14.855	9.463	0.0571	-0.0545	-0.2517
1PE	C27	347	12.184	14.536	9.753	0.0951	-0.0692	-0.3011
1PE	C28	348	12.623	14.569	9.922	0.1169	-0.0729	-0.3387
1PE	C29	349	12.846	14.956	9.846	0.1098	-0.0804	-0.3375
1PE	C30	350	12.635	15.328	9.721	0.0937	-0.0720	-0.2770
1PE	C31	351	12.385	15.590	9.483	0.0654	-0.0300	-0.2137
1PE	C32	352	12.023	15.797	9.596	0.0842	-0.0521	-0.1608
1PE	C33	353	11.916	15.960	10.002	0.1068	-0.0973	-0.1319
1PE	C34	354	12.142	15.840	10.356	0.1414	-0.1239	-0.1564
1PE	C35	355	12.561	15.706	10.351	0.1385	-0.1209	-0.2069
1PE	C36	356	12.905	15.639	10.048	0.1154	-0.0954	-0.2712
1PE	C37	357	13.138	15.374	9.765	0.0998	-0.0480	-0.2984
1PE	C38	358	13.418	15.237	9.455	0.0584	-0.0295	-0.3586
1PE	C39	359	13.626	14.942	9.183	0.0434	-0.0089	-0.4262
1PE	C40	360	13.623	14.671	8.812	0.0066	0.0422	-0.4262

1PE	C41	361	13.454	14.451	8.442	-0.0377	0.0714	-0.4403
1PE	C42	362	13.110	14.539	8.170	-0.0663	0.0848	-0.4101
1PE	C43	363	12.985	14.949	8.048	-0.0720	0.1010	-0.3374
1PE	C44	364	13.208	15.218	7.782	-0.0905	0.1317	-0.3442
1PE	C45	365	13.589	15.080	7.580	-0.1359	0.1526	-0.3892
1PE	C46	366	13.821	14.702	7.684	-0.1133	0.1515	-0.4406
1PE	C47	367	13.625	14.348	7.892	-0.0930	0.1205	-0.4625
1PE	C48	368	13.219	14.191	7.772	-0.0973	0.1287	-0.4311
1PE	C49	369	12.846	14.428	7.722	-0.0818	0.1300	-0.3654
1PE	C50	370	12.601	14.597	8.051	-0.0824	0.0956	-0.3499
1PE	C51	371	12.385	14.431	8.427	-0.0334	0.0574	-0.3217
1PE	C52	372	12.370	14.275	8.863	0.0084	0.0148	-0.3287
1PE	C53	373	12.423	14.382	9.319	0.0496	-0.0366	-0.3281
1PE	C54	374	12.537	14.796	9.476	0.0891	-0.0343	-0.3103
1PE	C55	375	12.439	15.140	9.246	0.0476	-0.0241	-0.2789
1PE	C56	376	12.066	15.206	9.036	0.0125	-0.0056	-0.2266
1PE	C57	377	11.661	15.168	9.202	0.0279	-0.0193	-0.1937
1PE	C58	378	11.572	15.092	9.632	0.0760	-0.0655	-0.1796
1PE	C59	379	11.871	14.968	9.941	0.1149	-0.0843	-0.2209
1PE	C60	380	12.306	14.997	9.975	0.1145	-0.0949	-0.2716
1PE	C61	381	12.617	15.208	10.220	0.1413	-0.1043	-0.2883
1PE	C62	382	13.050	15.240	10.309	0.1537	-0.0997	-0.3120
1PE	C63	383	13.426	15.435	10.174	0.1452	-0.0899	-0.3383
1PE	C64	384	13.697	15.467	9.827	0.0920	-0.0603	-0.3576
1PE	C65	385	13.920	15.353	9.443	0.0529	-0.0406	-0.3992
1PE	C66	386	14.118	15.070	9.135	0.0275	0.0126	-0.4567
1PE	C67	387	14.067	14.884	8.725	-0.0008	0.0557	-0.4421
1PE	C68	388	13.801	14.874	8.362	-0.0503	0.0861	-0.4289
1PE	C69	389	13.502	14.831	8.044	-0.0690	0.0915	-0.3943
1PE	C70	390	13.275	14.702	7.700	-0.1094	0.1299	-0.3838
1PE	C71	391	13.101	14.920	7.364	-0.1449	0.1693	-0.3409
1PE	C72	392	13.187	15.308	7.257	-0.1571	0.1781	-0.3318
1PE	C73	393	13.508	15.548	7.441	-0.1419	0.1685	-0.3475
1PE	C74	394	13.612	15.509	7.864	-0.1096	0.1229	-0.3445
1PE	C75	395	13.442	15.279	8.245	-0.0443	0.0957	-0.3538
1PE	C76	396	13.278	14.932	8.494	-0.0176	0.0664	-0.3647
1PE	C77	397	13.118	14.694	8.862	0.0092	0.0269	-0.3797
1PE	C78	398	12.692	14.661	9.005	0.0291	0.0104	-0.3355
1PE	C79	399	12.343	14.798	8.773	-0.0092	0.0318	-0.2829
1PE	C80	400	12.301	14.915	8.317	-0.0610	0.0573	-0.2720
1PE	C1	401	11.412	17.499	8.940	-0.0093	0.0029	0.0739
1PE	C2	402	11.539	17.561	8.487	-0.0479	0.0514	0.0374
1PE	C3	403	11.893	17.761	8.263	-0.0987	0.0744	0.0437
1PE	C4	404	12.156	17.935	7.962	-0.1164	0.1027	0.0289
1PE	C5	405	12.196	18.348	7.820	-0.1404	0.1216	0.0708
1PE	C6	406	12.009	18.701	8.032	-0.1074	0.0935	0.1003
1PE	C7	407	11.710	18.738	8.378	-0.0709	0.0588	0.1558
1PE	C8	408	11.588	18.898	8.805	-0.0326	0.0077	0.1891
1PE	C9	409	11.634	18.858	9.245	0.0086	-0.0080	0.1675
1PE	C10	410	11.827	18.627	9.594	0.0438	-0.0658	0.1306
1PE	C11	411	12.204	18.431	9.734	0.0704	-0.0747	0.0697
1PE	C12	412	12.600	18.214	9.743	0.0666	-0.0527	0.0194
1PE	C13	413	12.701	17.780	9.772	0.0794	-0.0572	-0.0450
1PE	C14	414	12.415	17.441	9.794	0.0798	-0.0534	-0.0360
1PE	C15	415	12.206	17.182	10.072	0.1081	-0.1093	-0.0610
1PE	C16	416	11.790	17.205	10.240	0.1323	-0.1128	-0.0076
1PE	C17	417	11.473	17.445	10.068	0.0894	-0.1029	0.0609
1PE	C18	418	11.538	17.830	9.840	0.0843	-0.0744	0.0928
1PE	C19	419	11.807	18.115	9.610	0.0536	-0.0497	0.0890
1PE	C20	420	12.091	18.229	9.270	0.0157	-0.0241	0.0605
1PE	C21	421	12.118	18.053	8.864	-0.0221	0.0243	0.0431
1PE	C22	422	11.692	18.016	8.633	-0.0581	0.0439	0.0838
1PE	C23	423	11.324	18.236	8.734	-0.0397	0.0280	0.1394
1PE	C24	424	11.131	18.162	9.133	0.0194	-0.0263	0.1516
1PE	C25	425	11.388	17.935	9.372	0.0185	-0.0425	0.1037
1PE	C26	426	11.771	17.820	9.169	0.0133	-0.0233	0.0550
1PE	C27	427	11.928	17.604	8.783	-0.0228	0.0256	0.0207
1PE	C28	428	12.333	17.600	8.636	-0.0384	0.0263	-0.0284
1PE	C29	429	12.634	17.798	8.873	-0.0134	0.0178	-0.0428
1PE	C30	430	12.550	17.984	9.271	0.0177	-0.0209	0.0109
1PE	C31	431	12.248	17.862	9.564	0.0363	-0.0521	0.0166
1PE	C32	432	11.918	17.592	9.597	0.0638	-0.0687	0.0200
1PE	C33	433	11.514	17.442	9.519	0.0428	-0.0383	0.0545

1PE	C34	434	11.219	17.115	9.635	0.0518	-0.0572	0.0633
1PE	C35	435	10.948	17.099	9.993	0.0961	-0.1140	0.0682
1PE	C36	436	10.951	17.405	10.304	0.1281	-0.1307	0.0915
1PE	C37	437	11.270	17.500	10.577	0.1700	-0.1478	0.0649
1PE	C38	438	11.671	17.698	10.492	0.1385	-0.1412	0.0577
1PE	C39	439	11.762	18.102	10.304	0.1158	-0.1247	0.0912
1PE	C40	440	11.531	18.298	9.987	0.0838	-0.0980	0.1286
1PE	C41	441	11.121	18.168	9.860	0.0764	-0.0946	0.1560
1PE	C42	442	10.735	18.357	9.966	0.0934	-0.0948	0.2130
1PE	C43	443	10.665	18.697	10.267	0.1046	-0.1229	0.2459
1PE	C44	444	10.844	18.774	10.699	0.1618	-0.1694	0.2483
1PE	C45	445	11.239	18.936	10.806	0.1683	-0.1688	0.2122
1PE	C46	446	11.605	18.929	10.538	0.1283	-0.1333	0.1628
1PE	C47	447	11.648	18.819	10.102	0.0950	-0.1032	0.1718
1PE	C48	448	11.475	18.979	9.727	0.0636	-0.0663	0.1908
1PE	C49	449	11.168	18.872	9.432	0.0436	-0.0445	0.2235
1PE	C50	450	11.179	18.661	9.037	-0.0232	-0.0127	0.1894
1PE	C51	451	11.216	18.684	8.570	-0.0628	0.0334	0.1872
1PE	C52	452	11.312	18.494	8.144	-0.1056	0.0793	0.1690
1PE	C53	453	11.686	18.266	8.016	-0.1084	0.0930	0.0947
1PE	C54	454	11.988	18.277	8.334	-0.0757	0.0673	0.0597
1PE	C55	455	11.870	18.461	8.770	-0.0479	0.0155	0.0986
1PE	C56	456	11.615	18.359	9.140	-0.0149	-0.0193	0.1155
1PE	C57	457	11.371	18.430	9.520	0.0303	-0.0575	0.1447
1PE	C58	458	11.143	18.656	9.894	0.0760	-0.0984	0.2086
1PE	C59	459	11.136	18.946	10.234	0.1130	-0.1216	0.2247
1PE	C60	460	10.963	19.259	10.489	0.1382	-0.1412	0.2864
1PE	C61	461	10.794	19.307	10.907	0.1806	-0.1807	0.3004
1PE	C62	462	10.859	19.040	11.251	0.1853	-0.2159	0.2512
1PE	C63	463	11.079	18.672	11.225	0.2031	-0.2174	0.2037
1PE	C64	464	11.189	18.414	10.892	0.1824	-0.1839	0.1946
1PE	C65	465	11.353	18.078	10.637	0.1630	-0.1585	0.1229
1PE	C66	466	11.258	17.881	10.251	0.1140	-0.1113	0.1033
1PE	C67	467	11.057	17.675	9.879	0.0868	-0.0953	0.1180
1PE	C68	468	11.052	17.571	9.456	0.0398	-0.0450	0.0916
1PE	C69	469	11.014	17.244	9.167	0.0160	-0.0304	0.0843
1PE	C70	470	11.156	17.032	8.803	-0.0139	0.0007	0.0452
1PE	C71	471	11.369	17.072	8.409	-0.0566	0.0526	0.0177
1PE	C72	472	11.605	17.231	8.082	-0.0790	0.0789	0.0115
1PE	C73	473	11.920	17.423	7.889	-0.1075	0.1108	0.0032
1PE	C74	474	12.180	17.497	7.556	-0.1456	0.1434	-0.0189
1PE	C75	475	12.050	17.654	7.142	-0.1958	0.1835	0.0122
1PE	C76	476	11.660	17.864	7.088	-0.2085	0.1785	0.0736
1PE	C77	477	11.378	17.952	7.421	-0.1605	0.1479	0.1031
1PE	C78	478	11.212	18.156	7.765	-0.1311	0.1086	0.1607
1PE	C79	479	10.806	18.206	7.906	-0.1170	0.1040	0.1791
1PE	C80	480	10.404	18.034	8.054	-0.0924	0.0821	0.2183
1PE	C1	481	11.087	14.966	9.682	0.0906	-0.0688	-0.1434
1PE	C2	482	11.176	15.270	9.340	0.0426	-0.0499	-0.1342
1PE	C3	483	11.323	15.569	9.032	0.0335	-0.0074	-0.1136
1PE	C4	484	11.296	15.812	8.658	-0.0105	0.0290	-0.0941
1PE	C5	485	11.099	16.198	8.474	-0.0445	0.0544	-0.0340
1PE	C6	486	11.131	16.645	8.474	-0.0396	0.0515	0.0132
1PE	C7	487	10.875	17.002	8.309	-0.0694	0.0584	0.0621
1PE	C8	488	10.527	16.900	8.039	-0.0961	0.0763	0.1015
1PE	C9	489	10.451	16.449	7.951	-0.0986	0.0870	0.0662
1PE	C10	490	10.697	16.097	8.119	-0.0821	0.0895	0.0233
1PE	C11	491	10.909	15.739	8.313	-0.0614	0.0505	-0.0592
1PE	C12	492	10.863	15.580	8.741	-0.0188	0.0153	-0.0650
1PE	C13	493	10.561	15.780	9.015	0.0255	-0.0091	-0.0119
1PE	C14	494	10.195	16.040	8.871	-0.0014	-0.0025	0.0479
1PE	C15	495	9.908	15.945	8.539	-0.0509	0.0269	0.0637
1PE	C16	496	9.887	15.645	8.204	-0.0618	0.0554	0.0394
1PE	C17	497	9.993	15.569	7.768	-0.1021	0.0901	0.0241
1PE	C18	498	10.100	15.757	7.378	-0.1496	0.1359	0.0161
1PE	C19	499	10.076	16.156	7.184	-0.1613	0.1683	0.0691
1PE	C20	500	9.960	16.582	7.156	-0.1851	0.1544	0.1238
1PE	C21	501	9.860	16.976	7.319	-0.1594	0.1468	0.1707
1PE	C22	502	9.850	17.288	7.603	-0.1367	0.1153	0.1950
1PE	C23	503	9.686	17.645	7.784	-0.1283	0.0934	0.2319
1PE	C24	504	9.611	18.015	7.585	-0.1527	0.1210	0.2771
1PE	C25	505	9.567	18.069	7.145	-0.1950	0.1470	0.2990
1PE	C26	506	9.603	17.731	6.860	-0.2304	0.1926	0.2700

1PE	C27	507	9.750	17.319	6.935	-0.2235	0.1825	0.2002
1PE	C28	508	9.999	16.971	6.821	-0.1995	0.1966	0.1496
1PE	C29	509	10.043	16.569	6.640	-0.2324	0.2072	0.1098
1PE	C30	510	9.786	16.233	6.780	-0.2277	0.1998	0.0957
1PE	C31	511	9.546	16.295	7.171	-0.1682	0.1568	0.1279
1PE	C32	512	9.437	16.695	7.367	-0.1691	0.1263	0.1763
1PE	C33	513	9.350	17.137	7.309	-0.1860	0.1273	0.2278
1PE	C34	514	9.475	17.563	7.335	-0.1595	0.1268	0.2748
1PE	C35	515	9.894	17.676	7.284	-0.1804	0.1512	0.2414
1PE	C36	516	10.198	17.368	7.199	-0.1770	0.1677	0.1737
1PE	C37	517	10.348	16.926	7.175	-0.1748	0.1587	0.1231
1PE	C38	518	10.459	16.548	6.934	-0.2100	0.1963	0.0657
1PE	C39	519	10.348	16.141	6.746	-0.2162	0.1993	0.0271
1PE	C40	520	10.044	15.800	6.817	-0.1894	0.2037	0.0383
1PE	C41	521	9.686	15.806	7.108	-0.1756	0.1460	0.0710
1PE	C42	522	9.652	15.955	7.555	-0.1262	0.1220	0.0875
1PE	C43	523	9.703	16.088	8.007	-0.0805	0.0734	0.1053
1PE	C44	524	9.665	16.383	8.350	-0.0617	0.0396	0.1229
1PE	C45	525	9.649	16.819	8.471	-0.0460	0.0338	0.1749
1PE	C46	526	10.014	17.045	8.542	-0.0508	0.0197	0.1611
1PE	C47	527	10.433	16.827	8.528	-0.0538	0.0206	0.0949
1PE	C48	528	10.659	16.486	8.442	-0.0550	0.0390	0.0330
1PE	C49	529	10.642	16.087	8.626	-0.0222	0.0217	0.0029
1PE	C50	530	10.414	15.728	8.519	-0.0179	0.0498	-0.0103
1PE	C51	531	10.391	15.570	8.117	-0.0771	0.0637	-0.0299
1PE	C52	532	10.455	15.771	7.730	-0.1269	0.1094	-0.0003
1PE	C53	533	10.507	16.074	7.442	-0.1489	0.1362	0.0188
1PE	C54	534	10.379	16.484	7.422	-0.1532	0.1536	0.0687
1PE	C55	535	10.164	16.780	7.695	-0.1343	0.1059	0.1025
1PE	C56	536	10.050	16.713	8.159	-0.0805	0.0771	0.1184
1PE	C57	537	10.183	16.344	8.386	-0.0512	0.0417	0.0754
1PE	C58	538	10.209	16.027	8.120	-0.0768	0.0619	0.0325
1PE	C59	539	10.097	16.145	7.709	-0.1148	0.1244	0.0597
1PE	C60	540	9.796	16.461	7.632	-0.1212	0.1214	0.1182
1PE	C61	541	9.653	16.873	7.815	-0.1175	0.0908	0.1819
1PE	C62	542	9.370	17.255	7.820	-0.1238	0.0846	0.2496
1PE	C63	543	9.226	17.687	7.961	-0.1028	0.0633	0.2906
1PE	C64	544	9.335	18.143	7.991	-0.1028	0.0676	0.3210
1PE	C65	545	9.624	18.458	7.798	-0.1314	0.0996	0.3365
1PE	C66	546	9.891	18.418	7.407	-0.1774	0.1231	0.3009
1PE	C67	547	10.071	18.134	7.094	-0.1981	0.1587	0.2720
1PE	C68	548	10.118	17.773	6.834	-0.2199	0.1886	0.2152
1PE	C69	549	10.249	17.385	6.685	-0.2434	0.2059	0.1768
1PE	C70	550	10.465	16.992	6.629	-0.2430	0.2042	0.1185
1PE	C71	551	10.643	16.683	6.361	-0.2591	0.2553	0.0580
1PE	C72	552	10.863	16.432	6.062	-0.2849	0.2720	0.0212
1PE	C73	553	11.238	16.242	5.893	-0.2916	0.2783	-0.0419
1PE	C74	554	11.612	16.097	5.685	-0.3403	0.3075	-0.0839
1PE	C75	555	12.046	15.981	5.537	-0.3415	0.3403	-0.1510
1PE	C76	556	12.440	15.726	5.484	-0.3292	0.3414	-0.2037
1PE	C77	557	12.772	15.509	5.731	-0.3168	0.3215	-0.2735
1PE	C78	558	12.954	15.216	6.042	-0.2783	0.2868	-0.3093
1PE	C79	559	13.079	15.018	6.441	-0.2337	0.2644	-0.3308
1PE	C80	560	13.233	14.960	6.870	-0.1897	0.2144	-0.3733
1PE	C1	561	11.728	15.473	9.965	0.0902	-0.0957	-0.1621
1PE	C2	562	11.657	15.652	10.362	0.1629	-0.1328	-0.1571
1PE	C3	563	11.661	16.094	10.491	0.1533	-0.1335	-0.0953
1PE	C4	564	12.005	16.359	10.384	0.1483	-0.1264	-0.1085
1PE	C5	565	12.359	16.225	10.111	0.1072	-0.0811	-0.1467
1PE	C6	566	12.510	15.876	9.865	0.0953	-0.0864	-0.2106
1PE	C7	567	12.844	15.766	9.529	0.0633	-0.0364	-0.2610
1PE	C8	568	13.209	15.684	9.257	0.0374	-0.0288	-0.3062
1PE	C9	569	13.584	15.534	9.082	0.0135	0.0104	-0.3468
1PE	C10	570	13.991	15.511	8.944	0.0031	0.0367	-0.3814
1PE	C11	571	14.313	15.314	8.702	-0.0217	0.0388	-0.4351
1PE	C12	572	14.271	15.109	8.285	-0.0546	0.0888	-0.4541
1PE	C13	573	13.943	15.131	7.943	-0.0775	0.1198	-0.4226
1PE	C14	574	14.014	15.413	7.571	-0.1097	0.1538	-0.4055
1PE	C15	575	14.410	15.623	7.640	-0.1206	0.1629	-0.4128
1PE	C16	576	14.569	15.572	8.063	-0.0691	0.1262	-0.4315
1PE	C17	577	14.374	15.729	8.442	-0.0446	0.0820	-0.3994
1PE	C18	578	14.366	15.840	8.882	0.0088	0.0295	-0.3783
1PE	C19	579	14.273	15.708	9.315	0.0351	-0.0164	-0.3958

1PE	C20	580	14.081	15.815	9.718	0.0747	-0.0517	-0.3712
1PE	C21	581	13.729	15.961	9.979	0.1233	-0.0789	-0.3192
1PE	C22	582	13.327	15.930	10.229	0.1376	-0.1227	-0.2758
1PE	C23	583	13.048	15.722	10.533	0.1743	-0.1219	-0.2770
1PE	C24	584	12.773	15.393	10.704	0.1875	-0.1412	-0.2735
1PE	C25	585	12.331	15.293	10.779	0.2001	-0.1693	-0.2402
1PE	C26	586	11.967	15.591	10.804	0.1889	-0.1763	-0.1869
1PE	C27	587	12.043	16.028	10.800	0.1708	-0.1715	-0.1427
1PE	C28	588	12.424	16.203	10.607	0.1892	-0.1362	-0.1637
1PE	C29	589	12.824	16.159	10.382	0.1440	-0.1185	-0.2134
1PE	C30	590	13.199	16.414	10.271	0.1363	-0.1029	-0.2218
1PE	C31	591	13.343	16.779	10.038	0.1184	-0.0748	-0.2122
1PE	C32	592	13.293	17.056	9.687	0.0777	-0.0548	-0.1724
1PE	C33	593	13.309	17.241	9.279	0.0319	-0.0174	-0.1506
1PE	C34	594	13.529	17.303	8.882	-0.0119	0.0230	-0.1707
1PE	C35	595	13.758	17.355	8.491	-0.0414	0.0573	-0.1858
1PE	C36	596	13.986	17.268	8.109	-0.0816	0.0946	-0.2163
1PE	C37	597	14.098	16.924	7.838	-0.1105	0.1304	-0.2564
1PE	C38	598	14.229	16.626	7.538	-0.1331	0.1710	-0.3170
1PE	C39	599	14.364	16.204	7.624	-0.1144	0.1529	-0.3489
1PE	C40	600	14.459	16.060	8.052	-0.0902	0.1259	-0.3810
1PE	C41	601	14.355	16.238	8.484	-0.0482	0.0745	-0.3676
1PE	C42	602	13.970	16.405	8.650	-0.0266	0.0593	-0.2989
1PE	C43	603	13.707	16.482	9.013	0.0171	0.0291	-0.2764
1PE	C44	604	13.370	16.747	9.135	0.0179	0.0118	-0.2134
1PE	C45	605	13.129	16.997	8.862	-0.0019	0.0331	-0.1656
1PE	C46	606	13.247	17.144	8.449	-0.0557	0.0726	-0.1551
1PE	C47	607	13.598	16.962	8.207	-0.0733	0.1062	-0.2086
1PE	C48	608	13.672	16.507	8.216	-0.0658	0.1007	-0.2753
1PE	C49	609	13.528	16.192	8.522	-0.0295	0.0712	-0.2738
1PE	C50	610	13.429	16.062	8.958	-0.0033	0.0104	-0.2855
1PE	C51	611	13.283	16.206	9.365	0.0300	-0.0152	-0.2549
1PE	C52	612	13.410	16.334	9.791	0.0897	-0.0557	-0.2481
1PE	C53	613	13.783	16.465	9.978	0.1038	-0.0765	-0.2899
1PE	C54	614	14.190	16.327	9.846	0.0939	-0.0652	-0.3474
1PE	C55	615	14.328	16.187	9.433	0.0575	-0.0030	-0.3565
1PE	C56	616	14.299	16.368	9.018	-0.0064	0.0216	-0.3390
1PE	C57	617	14.143	16.799	8.925	-0.0057	0.0275	-0.2887
1PE	C58	618	13.813	16.983	9.194	0.0194	-0.0065	-0.2214
1PE	C59	619	13.675	16.698	9.527	0.0433	-0.0317	-0.2480
1PE	C60	620	13.816	16.269	9.478	0.0656	-0.0161	-0.2957
1PE	C61	621	13.923	16.016	9.092	0.0106	0.0180	-0.3349
1PE	C62	622	13.916	15.900	8.637	-0.0207	0.0431	-0.3360
1PE	C63	623	13.972	16.085	8.215	-0.0537	0.1117	-0.3420
1PE	C64	624	14.154	16.495	8.107	-0.0723	0.1018	-0.3097
1PE	C65	625	14.079	16.855	8.396	-0.0541	0.0856	-0.2663
1PE	C66	626	13.709	16.865	8.682	-0.0375	0.0472	-0.2226
1PE	C67	627	13.353	16.658	8.566	-0.0330	0.0588	-0.2097
1PE	C68	628	13.175	16.699	8.150	-0.0872	0.1001	-0.1787
1PE	C69	629	13.218	17.097	7.910	-0.1186	0.1275	-0.1475
1PE	C70	630	13.386	17.479	8.091	-0.0998	0.0942	-0.1499
1PE	C71	631	13.337	17.642	8.531	-0.0457	0.0486	-0.1168
1PE	C72	632	13.114	17.612	8.938	-0.0130	0.0321	-0.0878
1PE	C73	633	13.108	17.714	9.381	0.0324	-0.0278	-0.0958
1PE	C74	634	13.219	17.595	9.805	0.0713	-0.0695	-0.1022
1PE	C75	635	13.274	17.311	10.149	0.1153	-0.0916	-0.1356
1PE	C76	636	13.208	17.208	10.585	0.1521	-0.1338	-0.1414
1PE	C77	637	12.947	16.989	10.882	0.1974	-0.1727	-0.1316
1PE	C78	638	12.804	16.589	11.068	0.2213	-0.1951	-0.1660
1PE	C79	639	12.755	16.139	10.976	0.2032	-0.1813	-0.2085
1PE	C80	640	12.486	15.781	10.872	0.2089	-0.1688	-0.2094
1PE	C1	641	13.642	15.814	9.473	0.0744	-0.0236	-0.3281
1PE	C2	642	13.303	15.840	9.738	0.0873	-0.0496	-0.2760
1PE	C3	643	12.969	16.116	9.857	0.1087	-0.0843	-0.2149
1PE	C4	644	12.603	16.302	9.665	0.0695	-0.0580	-0.1718
1PE	C5	645	12.173	16.261	9.639	0.0701	-0.0496	-0.1289
1PE	C6	646	11.722	16.188	9.564	0.0625	-0.0536	-0.0953
1PE	C7	647	11.471	16.065	9.200	0.0182	-0.0098	-0.0973
1PE	C8	648	11.423	16.305	8.839	-0.0064	0.0135	-0.0601
1PE	C9	649	11.549	16.727	8.779	-0.0036	0.0197	-0.0292
1PE	C10	650	11.745	17.121	8.715	-0.0223	0.0164	-0.0138
1PE	C11	651	12.005	17.291	8.391	-0.0680	0.0543	-0.0172
1PE	C12	652	12.346	17.490	8.139	-0.0923	0.1053	-0.0385

1PE	C13	653	12.571	17.853	8.243	-0.0934	0.0888	-0.0231
1PE	C14	654	12.470	18.238	8.465	-0.0640	0.0511	0.0313
1PE	C15	655	12.273	18.665	8.478	-0.0687	0.0622	0.0789
1PE	C16	656	12.090	18.909	8.815	-0.0373	0.0212	0.1093
1PE	C17	657	12.159	18.754	9.234	0.0157	-0.0178	0.1164
1PE	C18	658	12.521	18.486	9.276	0.0097	-0.0199	0.0271
1PE	C19	659	12.741	18.300	8.909	-0.0258	0.0188	0.0039
1PE	C20	660	12.950	18.046	8.574	-0.0627	0.0671	-0.0418
1PE	C21	661	13.081	17.896	8.178	-0.0876	0.0935	-0.0720
1PE	C22	662	13.105	17.840	7.743	-0.1369	0.1300	-0.0644
1PE	C23	663	12.976	18.025	7.357	-0.1784	0.1496	-0.0379
1PE	C24	664	12.620	18.212	7.169	-0.1847	0.1835	0.0028
1PE	C25	665	12.304	18.515	7.318	-0.1760	0.1705	0.0561
1PE	C26	666	12.354	18.816	7.669	-0.1451	0.1255	0.0890
1PE	C27	667	12.555	18.667	8.048	-0.1184	0.0928	0.0539
1PE	C28	668	12.750	18.265	8.022	-0.0945	0.1094	0.0045
1PE	C29	669	12.606	17.966	7.716	-0.1252	0.1226	-0.0189
1PE	C30	670	12.257	17.987	7.444	-0.1705	0.1467	0.0204
1PE	C31	671	12.118	18.141	7.035	-0.2051	0.1849	0.0409
1PE	C32	672	12.390	18.284	6.715	-0.2267	0.2022	0.0274
1PE	C33	673	12.826	18.140	6.653	-0.2559	0.2425	-0.0201
1PE	C34	674	13.045	17.812	6.885	-0.2289	0.1998	-0.0821
1PE	C35	675	12.910	17.554	7.207	-0.1898	0.1791	-0.0877
1PE	C36	676	12.746	17.490	7.611	-0.1537	0.1426	-0.0767
1PE	C37	677	12.853	17.463	8.043	-0.1097	0.0862	-0.0811
1PE	C38	678	12.845	17.517	8.501	-0.0627	0.0503	-0.0857
1PE	C39	679	12.723	17.265	8.867	-0.0153	0.0266	-0.1100
1PE	C40	680	12.675	16.791	8.875	-0.0104	0.0071	-0.1232
1PE	C41	681	12.589	16.318	8.823	-0.0058	0.0360	-0.1667
1PE	C42	682	12.293	16.069	8.696	-0.0136	0.0433	-0.1565
1PE	C43	683	12.166	16.016	8.280	-0.0658	0.0580	-0.1535
1PE	C44	684	12.395	16.149	7.887	-0.0990	0.1046	-0.1717
1PE	C45	685	12.855	16.176	7.845	-0.1075	0.1195	-0.2134
1PE	C46	686	13.131	16.131	8.205	-0.0691	0.0894	-0.2446
1PE	C47	687	13.065	16.030	8.616	-0.0273	0.0404	-0.2538
1PE	C48	688	12.895	15.989	9.025	0.0114	0.0161	-0.2288
1PE	C49	689	12.493	16.027	9.250	0.0470	-0.0103	-0.1985
1PE	C50	690	12.056	16.103	9.168	0.0335	-0.0038	-0.1373
1PE	C51	691	11.835	16.453	9.103	0.0103	-0.0125	-0.0778
1PE	C52	692	11.981	16.869	9.096	0.0100	0.0021	-0.0443
1PE	C53	693	12.237	17.130	8.828	-0.0214	0.0173	-0.0396
1PE	C54	694	12.503	17.138	8.452	-0.0550	0.0406	-0.0851
1PE	C55	695	12.750	16.982	8.115	-0.0845	0.0990	-0.1149
1PE	C56	696	12.633	16.606	8.005	-0.1107	0.1110	-0.1371
1PE	C57	697	12.314	16.480	8.301	-0.0564	0.0804	-0.1333
1PE	C58	698	12.224	16.630	8.749	-0.0072	0.0382	-0.1150
1PE	C59	699	12.368	16.539	9.202	0.0194	-0.0048	-0.1308
1PE	C60	700	12.820	16.425	9.254	0.0364	-0.0126	-0.1806
1PE	C61	701	13.068	16.452	8.897	0.0018	0.0320	-0.2027
1PE	C62	702	12.875	16.484	8.473	-0.0377	0.0600	-0.1750
1PE	C63	703	12.650	16.116	8.324	-0.0602	0.0589	-0.1946
1PE	C64	704	12.662	15.723	8.564	-0.0201	0.0347	-0.2390
1PE	C65	705	13.041	15.567	8.775	-0.0123	0.0322	-0.2889
1PE	C66	706	13.440	15.651	8.634	-0.0133	0.0389	-0.3120
1PE	C67	707	13.580	15.769	8.270	-0.0478	0.0635	-0.3110
1PE	C68	708	13.549	16.033	7.961	-0.0957	0.1252	-0.2919
1PE	C69	709	13.351	16.357	7.824	-0.1057	0.1414	-0.2363
1PE	C70	710	13.124	16.671	7.660	-0.1382	0.1395	-0.2004
1PE	C71	711	12.858	17.002	7.566	-0.1512	0.1419	-0.1374
1PE	C72	712	12.440	17.072	7.687	-0.1331	0.1291	-0.0869
1PE	C73	713	12.204	16.979	8.061	-0.0950	0.0909	-0.0757
1PE	C74	714	11.931	16.779	8.389	-0.0775	0.0659	-0.0571
1PE	C75	715	11.862	16.353	8.584	-0.0399	0.0344	-0.0943
1PE	C76	716	11.803	15.927	8.744	-0.0194	0.0404	-0.1180
1PE	C77	717	11.723	15.479	8.719	-0.0207	0.0268	-0.1659
1PE	C78	718	11.431	15.134	8.756	0.0096	0.0082	-0.1707
1PE	C79	719	11.055	15.035	8.537	-0.0218	0.0369	-0.1273
1PE	C80	720	10.726	15.261	8.334	-0.0528	0.0514	-0.0731
1PE	C1	721	10.273	18.541	9.987	0.0825	-0.1136	0.2569
1PE	C2	722	10.333	18.322	9.617	0.0625	-0.0683	0.2403
1PE	C3	723	10.131	18.262	9.255	0.0128	-0.0289	0.2746
1PE	C4	724	10.233	18.350	8.830	-0.0189	0.0124	0.2601
1PE	C5	725	10.486	18.135	8.556	-0.0504	0.0311	0.2127

1PE	C6	726	10.499	17.713	8.597	-0.0450	0.0280	0.1788
1PE	C7	727	10.172	17.477	8.779	-0.0395	0.0130	0.1934
1PE	C8	728	9.748	17.532	8.657	-0.0300	0.0149	0.2255
1PE	C9	729	9.622	17.787	8.282	-0.0681	0.0536	0.2459
1PE	C10	730	9.883	18.061	8.032	-0.0915	0.0685	0.2838
1PE	C11	731	10.121	18.414	7.861	-0.1285	0.1087	0.2719
1PE	C12	732	10.148	18.788	7.642	-0.1507	0.1159	0.3006
1PE	C13	733	10.415	18.904	7.303	-0.1882	0.1471	0.2867
1PE	C14	734	10.683	18.614	7.079	-0.2010	0.1734	0.2545
1PE	C15	735	10.621	18.171	6.963	-0.2138	0.1851	0.2007
1PE	C16	736	10.461	17.805	7.202	-0.1939	0.1596	0.1854
1PE	C17	737	10.583	17.703	7.609	-0.1358	0.1081	0.1664
1PE	C18	738	10.973	17.691	7.806	-0.1260	0.1083	0.1095
1PE	C19	739	11.153	17.468	8.152	-0.0879	0.0769	0.0822
1PE	C20	740	11.051	17.457	8.588	-0.0452	0.0356	0.0999
1PE	C21	741	10.683	17.272	8.717	-0.0410	0.0098	0.1092
1PE	C22	742	10.427	17.273	8.376	-0.0616	0.0417	0.1365
1PE	C23	743	10.586	17.511	8.090	-0.0939	0.0880	0.1556
1PE	C24	744	10.830	17.848	8.251	-0.0920	0.0684	0.1452
1PE	C25	745	10.936	17.930	8.696	-0.0361	0.0192	0.1396
1PE	C26	746	10.837	17.704	9.035	-0.0037	-0.0256	0.1471
1PE	C27	747	10.519	17.429	9.177	0.0158	-0.0209	0.1556
1PE	C28	748	10.072	17.432	9.285	0.0193	-0.0471	0.1881
1PE	C29	749	9.635	17.415	9.163	0.0118	-0.0363	0.2466
1PE	C30	750	9.288	17.505	8.865	-0.0324	-0.0073	0.2736
1PE	C31	751	9.312	17.414	8.413	-0.0695	0.0327	0.2669
1PE	C32	752	9.699	17.280	8.195	-0.0769	0.0579	0.2061
1PE	C33	753	10.091	17.185	8.020	-0.1073	0.0731	0.1494
1PE	C34	754	10.388	17.230	7.679	-0.1276	0.1148	0.1403
1PE	C35	755	10.657	17.277	7.340	-0.1683	0.1414	0.1280
1PE	C36	756	10.691	17.421	6.928	-0.1938	0.1860	0.1132
1PE	C37	757	10.885	17.767	6.782	-0.2295	0.1895	0.1448
1PE	C38	758	11.167	18.025	6.977	-0.2093	0.1747	0.1512
1PE	C39	759	11.504	18.317	6.935	-0.2218	0.1933	0.1351
1PE	C40	760	11.831	18.586	7.082	-0.2013	0.1721	0.1208
1PE	C41	761	11.885	18.746	7.503	-0.1619	0.1414	0.1303
1PE	C42	762	11.563	18.751	7.816	-0.1313	0.1257	0.1610
1PE	C43	763	11.117	18.726	7.738	-0.1527	0.1185	0.2062
1PE	C44	764	10.917	18.966	7.420	-0.1723	0.1337	0.2453
1PE	C45	765	11.121	19.338	7.237	-0.1967	0.1564	0.2652
1PE	C46	766	11.575	19.441	7.314	-0.1881	0.1614	0.2343
1PE	C47	767	11.841	19.240	7.646	-0.1496	0.1299	0.1778
1PE	C48	768	11.700	19.150	8.070	-0.1169	0.0791	0.1728
1PE	C49	769	11.283	19.015	8.202	-0.0988	0.0644	0.2150
1PE	C50	770	10.882	18.785	8.201	-0.0895	0.0697	0.2325
1PE	C51	771	10.512	18.529	8.166	-0.0799	0.0823	0.2652
1PE	C52	772	10.101	18.401	8.357	-0.0779	0.0525	0.2580
1PE	C53	773	10.001	17.993	8.543	-0.0544	0.0374	0.2435
1PE	C54	774	10.097	17.629	8.293	-0.0674	0.0686	0.1930
1PE	C55	775	10.158	17.647	7.855	-0.1166	0.1137	0.1983
1PE	C56	776	10.142	17.978	7.583	-0.1539	0.1306	0.2467
1PE	C57	777	10.417	18.277	7.444	-0.1577	0.1309	0.2402
1PE	C58	778	10.632	18.574	7.621	-0.1453	0.1156	0.2281
1PE	C59	779	10.598	18.942	7.825	-0.1375	0.1009	0.2795
1PE	C60	780	10.910	19.232	7.913	-0.1312	0.0927	0.2742
1PE	C61	781	11.323	19.209	7.720	-0.1465	0.1224	0.2361
1PE	C62	782	11.436	18.961	7.364	-0.1863	0.1722	0.1925
1PE	C63	783	11.201	18.761	7.039	-0.1976	0.1905	0.2023
1PE	C64	784	11.050	18.457	6.739	-0.2446	0.2182	0.1936
1PE	C65	785	11.234	18.115	6.494	-0.2503	0.2454	0.1392
1PE	C66	786	11.398	17.705	6.625	-0.2485	0.2188	0.1028
1PE	C67	787	11.237	17.449	6.975	-0.2040	0.1832	0.0713
1PE	C68	788	11.014	17.612	7.311	-0.1825	0.1506	0.1131
1PE	C69	789	10.866	18.034	7.395	-0.1792	0.1535	0.1659
1PE	C70	790	11.045	18.391	7.344	-0.1774	0.1553	0.1743
1PE	C71	791	11.455	18.476	7.414	-0.1666	0.1480	0.1576
1PE	C72	792	11.813	18.230	7.452	-0.1761	0.1452	0.1007
1PE	C73	793	11.804	17.842	7.629	-0.1482	0.1250	0.0648
1PE	C74	794	11.508	17.749	7.942	-0.1078	0.0945	0.0791
1PE	C75	795	11.293	18.002	8.260	-0.0786	0.0608	0.1240
1PE	C76	796	10.937	18.315	8.389	-0.0605	0.0601	0.1880
1PE	C77	797	10.797	18.456	8.790	-0.0322	0.0106	0.2240
1PE	C78	798	10.633	18.192	9.091	-0.0050	-0.0207	0.2044

1PE	C79	799	10.347	17.878	9.049	-0.0026	-0.0274	0.2068
1PE	C80	800	9.917	17.846	9.046	0.0010	-0.0281	0.2472
1PE	C1	801	11.505	14.637	8.615	-0.0108	0.0379	-0.2310
1PE	C2	802	11.887	14.578	8.365	-0.0266	0.0568	-0.2780
1PE	C3	803	12.121	14.503	7.983	-0.0733	0.0989	-0.2901
1PE	C4	804	12.241	14.634	7.564	-0.1170	0.1411	-0.2924
1PE	C5	805	12.220	14.704	7.096	-0.1688	0.1805	-0.2858
1PE	C6	806	12.113	14.887	6.690	-0.1845	0.2069	-0.2716
1PE	C7	807	12.259	14.943	6.272	-0.2456	0.2703	-0.2602
1PE	C8	808	12.412	15.288	6.031	-0.2739	0.2818	-0.2471
1PE	C9	809	12.153	15.614	5.894	-0.2985	0.3079	-0.1875
1PE	C10	810	11.721	15.656	5.898	-0.2992	0.2880	-0.1583
1PE	C11	811	11.275	15.723	5.957	-0.2840	0.2723	-0.0984
1PE	C12	812	10.907	15.923	6.133	-0.2837	0.2636	-0.0366
1PE	C13	813	10.794	16.169	6.499	-0.2427	0.2391	0.0043
1PE	C14	814	10.926	16.524	6.736	-0.2161	0.2035	0.0010
1PE	C15	815	11.312	16.710	6.778	-0.2208	0.2064	-0.0121
1PE	C16	816	11.657	16.670	6.470	-0.2372	0.2399	-0.0400
1PE	C17	817	11.651	16.502	6.046	-0.2821	0.2918	-0.0477
1PE	C18	818	11.954	16.468	5.705	-0.3369	0.3170	-0.0949
1PE	C19	819	12.325	16.671	5.781	-0.3206	0.3244	-0.1160
1PE	C20	820	12.321	16.892	6.144	-0.2828	0.2841	-0.0811
1PE	C21	821	11.951	17.078	6.384	-0.2733	0.2398	-0.0367
1PE	C22	822	11.728	17.451	6.291	-0.2619	0.2570	0.0160
1PE	C23	823	11.727	17.604	5.869	-0.3112	0.3023	0.0275
1PE	C24	824	11.732	17.304	5.526	-0.3517	0.3337	0.0146
1PE	C25	825	11.531	16.861	5.537	-0.3498	0.3298	-0.0166
1PE	C26	826	11.163	16.749	5.786	-0.2962	0.3144	0.0142
1PE	C27	827	10.934	17.049	6.064	-0.2865	0.2818	0.0562
1PE	C28	828	11.110	17.420	6.254	-0.2866	0.2619	0.0931
1PE	C29	829	11.335	17.778	6.122	-0.2844	0.2571	0.0960
1PE	C30	830	11.708	17.973	6.285	-0.2802	0.2665	0.0665
1PE	C31	831	11.949	17.837	6.647	-0.2428	0.2284	0.0471
1PE	C32	832	12.172	17.457	6.691	-0.2185	0.2253	-0.0171
1PE	C33	833	12.414	17.293	6.392	-0.2496	0.2588	-0.0706
1PE	C34	834	12.599	17.506	6.086	-0.2895	0.2892	-0.0575
1PE	C35	835	12.436	17.676	5.703	-0.3247	0.3218	-0.0384
1PE	C36	836	12.254	17.416	5.366	-0.3688	0.3398	-0.0319
1PE	C37	837	12.459	17.026	5.221	-0.3789	0.3721	-0.0825
1PE	C38	838	12.718	16.736	5.451	-0.3626	0.3566	-0.1387
1PE	C39	839	13.130	16.641	5.622	-0.3338	0.3297	-0.2035
1PE	C40	840	13.559	16.709	5.728	-0.3194	0.3254	-0.2343
1PE	C41	841	13.886	16.830	6.015	-0.2966	0.3042	-0.2532
1PE	C42	842	13.926	17.163	6.314	-0.2704	0.2893	-0.2151
1PE	C43	843	13.592	17.302	6.571	-0.2462	0.2428	-0.1764
1PE	C44	844	13.179	17.218	6.542	-0.2306	0.2486	-0.1406
1PE	C45	845	12.900	17.131	6.239	-0.2580	0.2777	-0.1220
1PE	C46	846	12.814	16.837	5.959	-0.3117	0.2976	-0.1519
1PE	C47	847	12.747	16.417	5.874	-0.3067	0.3135	-0.1832
1PE	C48	848	12.492	16.251	5.532	-0.3468	0.3352	-0.1634
1PE	C49	849	12.245	16.543	5.277	-0.3499	0.3493	-0.1064
1PE	C50	850	12.044	16.913	5.467	-0.3604	0.3553	-0.0537
1PE	C51	851	11.869	16.939	5.888	-0.3056	0.2953	-0.0278
1PE	C52	852	11.485	17.005	6.099	-0.3028	0.2644	0.0025
1PE	C53	853	11.198	16.715	6.298	-0.2698	0.2544	0.0166
1PE	C54	854	11.289	16.304	6.409	-0.2335	0.2329	-0.0442
1PE	C55	855	11.588	16.028	6.218	-0.2785	0.2664	-0.0955
1PE	C56	856	11.995	16.088	6.041	-0.2849	0.2787	-0.1218
1PE	C57	857	12.340	16.344	6.153	-0.2774	0.2651	-0.1535
1PE	C58	858	12.655	16.609	6.372	-0.2552	0.2552	-0.1567
1PE	C59	859	12.684	16.948	6.665	-0.2202	0.2262	-0.1264
1PE	C60	860	12.752	17.404	6.721	-0.2283	0.2176	-0.0877
1PE	C61	861	12.951	17.671	6.407	-0.2653	0.2564	-0.0747
1PE	C62	862	13.271	17.502	6.121	-0.2895	0.2928	-0.1320
1PE	C63	863	13.414	17.073	6.094	-0.2913	0.2870	-0.1880
1PE	C64	864	13.258	16.664	6.145	-0.2818	0.2869	-0.2166
1PE	C65	865	13.107	16.273	6.191	-0.2664	0.2866	-0.2240
1PE	C66	866	12.756	16.094	6.322	-0.2570	0.2529	-0.2156
1PE	C67	867	12.464	16.228	6.639	-0.2145	0.2275	-0.1644
1PE	C68	868	12.535	16.557	6.951	-0.2058	0.1900	-0.1430
1PE	C69	869	12.548	16.949	7.172	-0.1808	0.1821	-0.1131
1PE	C70	870	12.466	17.371	7.168	-0.1803	0.1818	-0.0223
1PE	C71	871	12.500	17.771	6.938	-0.2147	0.2035	-0.0319

1PE	C72	872	12.455	17.828	6.501	-0.2689	0.2293	-0.0132
1PE	C73	873	12.156	17.697	6.187	-0.2876	0.2742	-0.0014
1PE	C74	874	12.145	17.333	5.931	-0.3048	0.2948	-0.0237
1PE	C75	875	12.499	17.145	5.725	-0.3305	0.3292	-0.0826
1PE	C76	876	12.919	17.343	5.740	-0.3195	0.3225	-0.1007
1PE	C77	877	12.933	17.799	5.871	-0.3359	0.3145	-0.0602
1PE	C78	878	12.573	18.033	6.055	-0.3073	0.2865	-0.0045
1PE	C79	879	12.181	18.197	6.262	-0.2954	0.2787	0.0580
1PE	C80	880	11.889	18.344	6.597	-0.2521	0.2288	0.0898
1PE	C1	881	11.873	14.736	8.966	0.0062	-0.0000	-0.2380
1PE	C2	882	11.908	15.020	8.608	-0.0126	0.0256	-0.2235
1PE	C3	883	11.933	15.261	8.242	-0.0567	0.0674	-0.2145
1PE	C4	884	11.718	15.387	7.870	-0.1009	0.1116	-0.1692
1PE	C5	885	11.830	15.494	7.449	-0.1470	0.1512	-0.1756
1PE	C6	886	12.232	15.581	7.303	-0.1537	0.1678	-0.2092
1PE	C7	887	12.554	15.810	7.547	-0.1303	0.1431	-0.2191
1PE	C8	888	12.643	15.721	7.990	-0.0932	0.1086	-0.2327
1PE	C9	889	12.449	15.368	8.142	-0.0600	0.0847	-0.2415
1PE	C10	890	12.255	15.113	7.826	-0.0856	0.1046	-0.2571
1PE	C11	891	12.348	15.100	7.377	-0.1467	0.1596	-0.2531
1PE	C12	892	12.365	15.244	6.960	-0.1877	0.2074	-0.2540
1PE	C13	893	12.356	15.602	6.707	-0.2056	0.2192	-0.2151
1PE	C14	894	12.340	15.837	6.314	-0.2517	0.2652	-0.1985
1PE	C15	895	12.543	15.929	5.919	-0.3048	0.2883	-0.2012
1PE	C16	896	12.949	16.007	5.696	-0.3191	0.3250	-0.2481
1PE	C17	897	13.365	16.211	5.764	-0.3104	0.3233	-0.2654
1PE	C18	898	13.691	16.355	6.073	-0.3014	0.3080	-0.2781
1PE	C19	899	13.939	16.403	6.463	-0.2460	0.2646	-0.3115
1PE	C20	900	13.834	16.458	6.899	-0.2084	0.2288	-0.2832
1PE	C21	901	13.978	16.264	7.268	-0.1529	0.1883	-0.3121
1PE	C22	902	14.176	15.847	7.253	-0.1635	0.1913	-0.3701
1PE	C23	903	14.044	15.502	6.947	-0.2033	0.2240	-0.3951
1PE	C24	904	13.812	15.477	6.530	-0.2266	0.2501	-0.3640
1PE	C25	905	13.340	15.466	6.449	-0.2453	0.2698	-0.3274
1PE	C26	906	12.997	15.406	6.768	-0.2185	0.2204	-0.3016
1PE	C27	907	12.742	15.515	7.148	-0.1624	0.1807	-0.2653
1PE	C28	908	12.742	15.347	7.595	-0.1347	0.1602	-0.2873
1PE	C29	909	12.699	14.904	7.656	-0.1130	0.1326	-0.3217
1PE	C30	910	12.684	14.669	7.275	-0.1410	0.1604	-0.3359
1PE	C31	911	12.707	14.867	6.851	-0.1954	0.2096	-0.3161
1PE	C32	912	12.607	15.191	6.529	-0.2454	0.2375	-0.2836
1PE	C33	913	12.743	15.560	6.333	-0.2491	0.2523	-0.2510
1PE	C34	914	13.062	15.744	6.111	-0.2742	0.2861	-0.2754
1PE	C35	915	13.473	15.894	6.146	-0.2728	0.2943	-0.3051
1PE	C36	916	13.884	15.926	6.308	-0.2574	0.2721	-0.3466
1PE	C37	917	14.095	15.970	6.715	-0.2224	0.2383	-0.3570
1PE	C38	918	14.343	16.275	6.941	-0.2123	0.2297	-0.3564
1PE	C39	919	14.247	16.720	7.023	-0.1882	0.2234	-0.2833
1PE	C40	920	13.924	16.962	6.793	-0.2205	0.2426	-0.2343
1PE	C41	921	13.628	16.787	6.476	-0.2542	0.2688	-0.2337
1PE	C42	922	13.452	16.369	6.544	-0.2226	0.2469	-0.2621
1PE	C43	923	13.364	16.272	6.978	-0.2010	0.2079	-0.2480
1PE	C44	924	13.535	16.526	7.324	-0.1607	0.1821	-0.2479
1PE	C45	925	13.839	16.873	7.306	-0.1552	0.1883	-0.2384
1PE	C46	926	13.724	17.293	7.153	-0.1927	0.1887	-0.1863
1PE	C47	927	13.323	17.375	6.996	-0.2115	0.2142	-0.1562
1PE	C48	928	13.011	17.089	7.011	-0.2013	0.2039	-0.1371
1PE	C49	929	12.988	16.693	7.172	-0.1646	0.1780	-0.1653
1PE	C50	930	12.691	16.522	7.465	-0.1466	0.1434	-0.1463
1PE	C51	931	12.414	16.250	7.375	-0.1457	0.1472	-0.1567
1PE	C52	932	12.446	15.949	7.054	-0.1785	0.1827	-0.2056
1PE	C53	933	12.777	15.849	6.757	-0.2105	0.2264	-0.2460
1PE	C54	934	13.161	15.930	6.575	-0.2363	0.2545	-0.2630
1PE	C55	935	13.566	15.904	6.686	-0.2183	0.2271	-0.3058
1PE	C56	936	13.718	15.881	7.088	-0.1659	0.1999	-0.3345
1PE	C57	937	13.518	16.045	7.449	-0.1461	0.1637	-0.2930
1PE	C58	938	13.115	16.240	7.414	-0.1464	0.1760	-0.2463
1PE	C59	939	12.869	16.197	7.096	-0.1841	0.1813	-0.2010
1PE	C60	940	12.941	16.372	6.701	-0.2183	0.2332	-0.1822
1PE	C61	941	13.151	16.737	6.648	-0.2388	0.2327	-0.1956
1PE	C62	942	13.449	16.867	6.953	-0.1975	0.2083	-0.1942
1PE	C63	943	13.356	17.005	7.376	-0.1682	0.1755	-0.1763
1PE	C64	944	13.275	17.413	7.518	-0.1408	0.1609	-0.1262

1PE	C65	945	13.420	17.774	7.277	-0.1928	0.1642	-0.1177
1PE	C66	946	13.649	17.738	6.857	-0.2190	0.2154	-0.1451
1PE	C67	947	14.025	17.460	6.768	-0.2337	0.2276	-0.1922
1PE	C68	948	14.231	17.246	7.122	-0.1806	0.2025	-0.2472
1PE	C69	949	14.078	17.338	7.538	-0.1422	0.1507	-0.2258
1PE	C70	950	13.692	17.197	7.706	-0.1255	0.1367	-0.1990
1PE	C71	951	13.613	16.767	7.758	-0.1216	0.1347	-0.2427
1PE	C72	952	13.842	16.370	7.729	-0.1226	0.1530	-0.2929
1PE	C73	953	13.967	15.917	7.729	-0.1247	0.1444	-0.3495
1PE	C74	954	14.063	15.613	8.052	-0.0668	0.1098	-0.3857
1PE	C75	955	13.900	15.415	8.427	-0.0432	0.0810	-0.3883
1PE	C76	956	13.651	15.190	8.725	0.0030	0.0533	-0.3898
1PE	C77	957	13.254	15.164	8.947	0.0186	0.0296	-0.3509
1PE	C78	958	12.809	15.118	8.878	0.0125	0.0254	-0.3115
1PE	C79	959	12.435	15.275	8.683	-0.0217	0.0372	-0.2423
1PE	C80	960	12.180	15.585	8.524	-0.0211	0.0599	-0.1949
1PE	C1	961	9.249	18.422	8.829	-0.0349	-0.0076	0.3567
1PE	C2	962	9.429	18.717	8.543	-0.0693	0.0210	0.3757
1PE	C3	963	9.736	18.749	8.220	-0.1037	0.0498	0.3412
1PE	C4	964	10.161	18.879	8.146	-0.0885	0.0723	0.3174
1PE	C5	965	10.506	19.139	8.290	-0.0866	0.0546	0.3059
1PE	C6	966	10.554	19.484	8.581	-0.0573	0.0329	0.3351
1PE	C7	967	10.236	19.641	8.907	-0.0228	-0.0021	0.3819
1PE	C8	968	9.795	19.561	8.825	-0.0463	-0.0005	0.4136
1PE	C9	969	9.702	19.154	8.648	-0.0469	0.0258	0.3954
1PE	C10	970	9.996	18.808	8.652	-0.0510	0.0128	0.3327
1PE	C11	971	10.440	18.753	8.606	-0.0515	0.0266	0.2675
1PE	C12	972	10.823	18.988	8.693	-0.0403	0.0259	0.2724
1PE	C13	973	11.150	19.167	8.984	-0.0115	0.0033	0.2368
1PE	C14	974	11.258	19.358	9.404	0.0325	-0.0272	0.2485
1PE	C15	975	11.105	19.302	9.849	0.0538	-0.0849	0.2657
1PE	C16	976	10.719	19.158	10.037	0.0882	-0.1081	0.2882
1PE	C17	977	10.299	19.023	10.065	0.0896	-0.1173	0.3232
1PE	C18	978	9.999	18.851	10.315	0.1123	-0.1329	0.3460
1PE	C19	979	9.595	18.740	10.451	0.1324	-0.1605	0.3706
1PE	C20	980	9.383	18.589	10.802	0.1680	-0.2019	0.3696
1PE	C21	981	9.345	18.166	10.914	0.1793	-0.1995	0.3266
1PE	C22	982	9.416	17.798	10.644	0.1613	-0.1838	0.2951
1PE	C23	983	9.410	17.853	10.178	0.1095	-0.1304	0.2785
1PE	C24	984	9.307	18.276	10.069	0.1001	-0.1284	0.3601
1PE	C25	985	9.119	18.500	10.394	0.1276	-0.1656	0.3901
1PE	C26	986	8.897	18.269	10.728	0.1552	-0.2139	0.3788
1PE	C27	987	8.934	17.803	10.760	0.1669	-0.1967	0.3349
1PE	C28	988	9.046	17.543	10.394	0.1319	-0.1520	0.2884
1PE	C29	989	8.968	17.670	9.951	0.0877	-0.0985	0.3204
1PE	C30	990	9.159	17.995	9.704	0.0673	-0.1015	0.3361
1PE	C31	991	9.606	18.078	9.684	0.0640	-0.0880	0.2881
1PE	C32	992	9.984	17.889	9.563	0.0357	-0.0740	0.2528
1PE	C33	993	10.401	17.772	9.557	0.0551	-0.0586	0.1831
1PE	C34	994	10.785	17.991	9.524	0.0370	-0.0491	0.1713
1PE	C35	995	10.867	18.445	9.485	0.0394	-0.0447	0.2057
1PE	C36	996	10.645	18.777	9.677	0.0506	-0.0703	0.2623
1PE	C37	997	10.231	18.869	9.576	0.0474	-0.0691	0.3205
1PE	C38	998	9.895	18.592	9.634	0.0450	-0.0863	0.3048
1PE	C39	999	9.774	18.481	10.042	0.0856	-0.1164	0.3237
1PE	C40	1000	10.087	18.371	10.420	0.1257	-0.1477	0.2650
1PE	C41	1001	10.337	18.663	10.658	0.1513	-0.1732	0.2702
1PE	C42	1002	10.482	19.091	10.548	0.1480	-0.1589	0.3024
1PE	C43	1003	10.342	19.450	10.313	0.1074	-0.1353	0.3582
1PE	C44	1004	10.029	19.544	9.994	0.0905	-0.1159	0.3806
1PE	C45	1005	9.625	19.496	9.817	0.0705	-0.1034	0.4449
1PE	C46	1006	9.270	19.246	9.850	0.0545	-0.1121	0.4461
1PE	C47	1007	9.063	18.934	9.604	0.0488	-0.0844	0.4289
1PE	C48	1008	9.185	18.776	9.206	-0.0062	-0.0314	0.3898
1PE	C49	1009	9.580	18.822	9.021	-0.0236	-0.0173	0.3623
1PE	C50	1010	9.984	18.714	9.149	0.0050	-0.0192	0.3303
1PE	C51	1011	10.427	18.660	9.154	0.0041	-0.0288	0.2684
1PE	C52	1012	10.744	18.900	9.185	0.0014	-0.0329	0.2741
1PE	C53	1013	10.767	19.222	9.496	0.0340	-0.0509	0.2876
1PE	C54	1014	10.396	19.364	9.703	0.0594	-0.0830	0.3475
1PE	C55	1015	9.986	19.307	9.528	0.0356	-0.0686	0.3767
1PE	C56	1016	9.959	19.194	9.111	-0.0046	-0.0350	0.3647
1PE	C57	1017	10.340	19.129	8.907	-0.0157	-0.0156	0.3297

1PE	C58	1018	10.680	19.388	9.043	-0.0048	-0.0053	0.3086
1PE	C59	1019	10.577	19.700	9.355	0.0077	-0.0430	0.3459
1PE	C60	1020	10.144	19.785	9.501	0.0253	-0.0565	0.4021
1PE	C61	1021	9.727	19.706	9.317	0.0201	-0.0441	0.4472
1PE	C62	1022	9.490	19.316	9.261	0.0117	-0.0506	0.4228
1PE	C63	1023	9.597	18.973	9.502	0.0330	-0.0616	0.3901
1PE	C64	1024	9.785	19.013	9.897	0.0699	-0.0938	0.3750
1PE	C65	1025	9.647	19.222	10.273	0.1058	-0.1492	0.4090
1PE	C66	1026	9.515	19.189	10.722	0.1446	-0.1899	0.4037
1PE	C67	1027	9.193	19.019	10.977	0.1742	-0.2023	0.4374
1PE	C68	1028	8.856	18.774	10.846	0.1649	-0.1862	0.4484
1PE	C69	1029	8.686	18.757	10.436	0.1326	-0.1698	0.4604
1PE	C70	1030	8.840	18.705	10.017	0.0897	-0.1261	0.4302
1PE	C71	1031	8.913	18.446	9.656	0.0478	-0.0871	0.3945
1PE	C72	1032	9.072	18.249	9.272	0.0103	-0.0586	0.3659
1PE	C73	1033	9.009	17.969	8.899	-0.0217	-0.0196	0.3464
1PE	C74	1034	9.178	18.009	8.456	-0.0611	0.0355	0.3305
1PE	C75	1035	9.577	18.263	8.416	-0.0652	0.0392	0.3043
1PE	C76	1036	9.755	18.394	8.805	-0.0400	-0.0084	0.3045
1PE	C77	1037	9.597	18.356	9.237	0.0068	-0.0359	0.3385
1PE	C78	1038	9.399	18.528	9.617	0.0376	-0.0927	0.3537
1PE	C79	1039	9.349	18.774	10.001	0.0652	-0.1215	0.3966
1PE	C80	1040	9.166	19.005	10.357	0.1244	-0.1487	0.4329
1PE	C1	1041	11.327	15.546	7.561	-0.1390	0.1313	-0.1200
1PE	C2	1042	11.032	15.249	7.421	-0.1458	0.1485	-0.1237
1PE	C3	1043	10.886	14.889	7.644	-0.1172	0.1027	-0.1282
1PE	C4	1044	11.077	14.746	8.039	-0.0699	0.0751	-0.1542
1PE	C5	1045	11.480	14.534	8.031	-0.0698	0.0852	-0.2285
1PE	C6	1046	11.739	14.481	7.641	-0.1120	0.1319	-0.2524
1PE	C7	1047	11.741	14.590	7.198	-0.1591	0.1743	-0.2472
1PE	C8	1048	11.573	14.853	6.858	-0.1789	0.2002	-0.2087
1PE	C9	1049	11.654	15.122	6.509	-0.2159	0.2503	-0.1904
1PE	C10	1050	11.568	15.540	6.376	-0.2398	0.2449	-0.1404
1PE	C11	1051	11.218	15.811	6.494	-0.2499	0.2351	-0.0842
1PE	C12	1052	10.832	15.681	6.671	-0.2242	0.2090	-0.0554
1PE	C13	1053	10.786	15.315	6.953	-0.1844	0.1929	-0.0902
1PE	C14	1054	11.059	14.945	7.027	-0.1890	0.1801	-0.1564
1PE	C15	1055	11.348	14.842	7.361	-0.1390	0.1533	-0.1774
1PE	C16	1056	11.383	15.046	7.753	-0.1106	0.1031	-0.1650
1PE	C17	1057	11.063	15.281	7.941	-0.0941	0.0875	-0.1313
1PE	C18	1058	10.660	15.323	7.777	-0.1189	0.1291	-0.0761
1PE	C19	1059	10.472	15.392	7.364	-0.1444	0.1412	-0.0465
1PE	C20	1060	10.495	15.741	7.057	-0.1826	0.1737	-0.0116
1PE	C21	1061	10.785	16.084	7.013	-0.1765	0.1819	-0.0095
1PE	C22	1062	11.212	16.152	6.901	-0.2011	0.1874	-0.0502
1PE	C23	1063	11.610	16.293	6.865	-0.2117	0.2103	-0.0601
1PE	C24	1064	11.778	16.706	6.959	-0.1972	0.2020	-0.0517
1PE	C25	1065	11.529	17.032	7.112	-0.1830	0.1831	-0.0028
1PE	C26	1066	11.104	17.030	7.278	-0.1820	0.1554	0.0468
1PE	C27	1067	10.819	16.949	6.989	-0.2113	0.1782	0.0530
1PE	C28	1068	10.981	17.056	6.599	-0.2372	0.2298	0.0622
1PE	C29	1069	11.440	17.161	6.574	-0.2417	0.2299	0.0375
1PE	C30	1070	11.716	17.398	6.868	-0.2182	0.1989	0.0116
1PE	C31	1071	11.644	17.442	7.358	-0.1576	0.1678	0.0350
1PE	C32	1072	11.327	17.351	7.664	-0.1509	0.1288	0.0622
1PE	C33	1073	10.912	17.176	7.765	-0.1268	0.1148	0.0744
1PE	C34	1074	10.699	16.819	7.567	-0.1361	0.1230	0.0614
1PE	C35	1075	10.898	16.533	7.248	-0.1731	0.1727	0.0223
1PE	C36	1076	11.339	16.564	7.252	-0.1795	0.1599	-0.0255
1PE	C37	1077	11.476	16.870	7.562	-0.1269	0.1259	-0.0126
1PE	C38	1078	11.257	16.876	7.960	-0.0926	0.0986	0.0101
1PE	C39	1079	10.933	16.588	8.007	-0.1006	0.0703	0.0235
1PE	C40	1080	10.858	16.312	7.678	-0.1166	0.1218	-0.0020
1PE	C41	1081	11.163	16.108	7.414	-0.1364	0.1393	-0.0397
1PE	C42	1082	11.557	15.911	7.318	-0.1600	0.1606	-0.0984
1PE	C43	1083	11.927	15.946	7.067	-0.1715	0.1904	-0.1395
1PE	C44	1084	12.103	16.325	6.983	-0.1945	0.1943	-0.1141
1PE	C45	1085	12.158	16.663	7.294	-0.1735	0.1517	-0.0914
1PE	C46	1086	12.183	16.580	7.728	-0.1325	0.1229	-0.0982
1PE	C47	1087	11.910	16.278	7.946	-0.1096	0.1018	-0.1035
1PE	C48	1088	11.632	15.947	7.829	-0.1005	0.1138	-0.0998
1PE	C49	1089	11.349	15.685	8.043	-0.0725	0.0928	-0.0901
1PE	C50	1090	11.365	15.398	8.334	-0.0468	0.0644	-0.1385

1PE	C51	1091	11.513	14.994	8.264	-0.0483	0.0538	-0.1898
1PE	C52	1092	11.815	14.886	7.949	-0.0847	0.0839	-0.2280
1PE	C53	1093	11.847	14.984	7.526	-0.1211	0.1493	-0.2313
1PE	C54	1094	11.916	15.101	7.124	-0.1683	0.1737	-0.2138
1PE	C55	1095	11.879	15.447	6.870	-0.2004	0.2072	-0.1754
1PE	C56	1096	11.529	15.729	6.861	-0.2045	0.2175	-0.1301
1PE	C57	1097	11.131	15.692	7.072	-0.1840	0.1856	-0.0841
1PE	C58	1098	10.862	15.713	7.403	-0.1484	0.1393	-0.0605
1PE	C59	1099	10.937	15.791	7.809	-0.1283	0.1169	-0.0734
1PE	C60	1100	11.188	16.140	7.984	-0.0895	0.1006	-0.0353
1PE	C61	1101	11.408	16.420	7.741	-0.1216	0.1250	-0.0545
1PE	C62	1102	11.768	16.343	7.468	-0.1487	0.1521	-0.0758
1PE	C63	1103	12.051	15.978	7.540	-0.1453	0.1393	-0.1523
1PE	C64	1104	12.097	15.700	7.900	-0.1011	0.1062	-0.1761
1PE	C65	1105	11.785	15.726	8.256	-0.0601	0.0698	-0.1474
1PE	C66	1106	11.549	16.074	8.326	-0.0496	0.0652	-0.0796
1PE	C67	1107	11.536	16.501	8.217	-0.0639	0.0637	-0.0406
1PE	C68	1108	11.763	16.767	7.933	-0.1085	0.1093	-0.0364
1PE	C69	1109	11.940	17.021	7.599	-0.1435	0.1262	-0.0316
1PE	C70	1110	12.028	17.137	7.186	-0.1786	0.1734	-0.0169
1PE	C71	1111	12.196	16.979	6.820	-0.2159	0.2094	-0.0777
1PE	C72	1112	12.171	16.641	6.563	-0.2469	0.2194	-0.0906
1PE	C73	1113	11.960	16.265	6.500	-0.2437	0.2302	-0.1082
1PE	C74	1114	11.921	15.839	6.559	-0.2289	0.2311	-0.1531
1PE	C75	1115	12.051	15.417	6.374	-0.2348	0.2487	-0.2174
1PE	C76	1116	11.868	15.189	6.037	-0.2802	0.2695	-0.2093
1PE	C77	1117	11.415	15.228	5.953	-0.2817	0.2953	-0.1543
1PE	C78	1118	11.125	15.333	6.297	-0.2525	0.2485	-0.1271
1PE	C79	1119	11.243	15.331	6.730	-0.2046	0.2064	-0.1217
1PE	C80	1120	11.455	15.292	7.130	-0.1670	0.1755	-0.1604

20.31205 20.31205 20.31205

3.2 Polyethylene NP System Topology

```
#include "martini_v2.0_PEO_PS_CNP.itp"

#include "PE160.itp"

[ system ]
; name
 POLYETHYLENE

[ molecules ]
; name                 number
PE                     14
```

3.3 Polyethylene NP Topology

```
[moleculetype]
 ; molname     nrexcl
 PE                 3

 [atoms]
 ; id   type    resnr   residu  atom    cgnr    charge
  1  C1           1  PE  C1              1           0
  2  C1           1  PE  C2              2           0
  3  C1           1  PE  C3              3           0
  4  C1           1  PE  C4              4           0
  5  C1           1  PE  C5              5           0
  6  C1           1  PE  C6              6           0
  7  C1           1  PE  C7              7           0
  8  C1           1  PE  C8              8           0
  9  C1           1  PE  C9              9           0
  10  C1           1  PE  C10            10           0
  11  C1           1  PE  C11            11           0
  12  C1           1  PE  C12            12           0
  13  C1           1  PE  C13            13           0
  14  C1           1  PE  C14            14           0
  15  C1           1  PE  C15            15           0
  16  C1           1  PE  C16            16           0
  17  C1           1  PE  C17            17           0
  18  C1           1  PE  C18            18           0
  19  C1           1  PE  C19            19           0
  20  C1           1  PE  C20            20           0
  21  C1           1  PE  C21            21           0
  22  C1           1  PE  C22            22           0
  23  C1           1  PE  C23            23           0
  24  C1           1  PE  C24            24           0
  25  C1           1  PE  C25            25           0
  26  C1           1  PE  C26            26           0
  27  C1           1  PE  C27            27           0
  28  C1           1  PE  C28            28           0
  29  C1           1  PE  C29            29           0
  30  C1           1  PE  C30            30           0
  31  C1           1  PE  C31            31           0
  32  C1           1  PE  C32            32           0
  33  C1           1  PE  C33            33           0
  34  C1           1  PE  C34            34           0
  35  C1           1  PE  C35            35           0
  36  C1           1  PE  C36            36           0
  37  C1           1  PE  C37            37           0
  38  C1           1  PE  C38            38           0
  39  C1           1  PE  C39            39           0
  40  C1           1  PE  C40            40           0
  41  C1           1  PE  C41            41           0
  42  C1           1  PE  C42            42           0
  43  C1           1  PE  C43            43           0
  44  C1           1  PE  C44            44           0
  45  C1           1  PE  C45            45           0
  46  C1           1  PE  C46            46           0
  47  C1           1  PE  C47            47           0
```

```
 48 C1           1 PE C48             48           0
 49 C1           1 PE C49             49           0
 50 C1           1 PE C50             50           0
 51 C1           1 PE C51             51           0
 52 C1           1 PE C52             52           0
 53 C1           1 PE C53             53           0
 54 C1           1 PE C54             54           0
 55 C1           1 PE C55             55           0
 56 C1           1 PE C56             56           0
 57 C1           1 PE C57             57           0
 58 C1           1 PE C58             58           0
 59 C1           1 PE C59             59           0
 60 C1           1 PE C60             60           0
 61 C1           1 PE C61             61           0
 62 C1           1 PE C62             62           0
 63 C1           1 PE C63             63           0
 64 C1           1 PE C64             64           0
 65 C1           1 PE C65             65           0
 66 C1           1 PE C66             66           0
 67 C1           1 PE C67             67           0
 68 C1           1 PE C68             68           0
 69 C1           1 PE C69             69           0
 70 C1           1 PE C70             70           0
 71 C1           1 PE C71             71           0
 72 C1           1 PE C72             72           0
 73 C1           1 PE C73             73           0
 74 C1           1 PE C74             74           0
 75 C1           1 PE C75             75           0
 76 C1           1 PE C76             76           0
 77 C1           1 PE C77             77           0
 78 C1           1 PE C78             78           0
 79 C1           1 PE C79             79           0
 80 C1           1 PE C80             80           0

[bonds]
; i j  funct   length  force.c.
          1           2           1  0.46000001           2000
          2           3           1  0.46000001           2000
          3           4           1  0.46000001           2000
          4           5           1  0.46000001           2000
          5           6           1  0.46000001           2000
          6           7           1  0.46000001           2000
          7           8           1  0.46000001           2000
          8           9           1  0.46000001           2000
          9          10           1  0.46000001           2000
         10          11           1  0.46000001           2000
         11          12           1  0.46000001           2000
         12          13           1  0.46000001           2000
         13          14           1  0.46000001           2000
         14          15           1  0.46000001           2000
         15          16           1  0.46000001           2000
         16          17           1  0.46000001           2000
         17          18           1  0.46000001           2000
         18          19           1  0.46000001           2000
         19          20           1  0.46000001           2000
         20          21           1  0.46000001           2000
         21          22           1  0.46000001           2000
         22          23           1  0.46000001           2000
         23          24           1  0.46000001           2000
         24          25           1  0.46000001           2000
         25          26           1  0.46000001           2000
         26          27           1  0.46000001           2000
         27          28           1  0.46000001           2000
         28          29           1  0.46000001           2000
         29          30           1  0.46000001           2000
         30          31           1  0.46000001           2000
         31          32           1  0.46000001           2000
         32          33           1  0.46000001           2000
         33          34           1  0.46000001           2000
         34          35           1  0.46000001           2000
         35          36           1  0.46000001           2000
         36          37           1  0.46000001           2000
         37          38           1  0.46000001           2000
```

```
        38          39           1  0.46000001            2000
        39          40           1  0.46000001            2000
        40          41           1  0.46000001            2000
        41          42           1  0.46000001            2000
        42          43           1  0.46000001            2000
        43          44           1  0.46000001            2000
        44          45           1  0.46000001            2000
        45          46           1  0.46000001            2000
        46          47           1  0.46000001            2000
        47          48           1  0.46000001            2000
        48          49           1  0.46000001            2000
        49          50           1  0.46000001            2000
        50          51           1  0.46000001            2000
        51          52           1  0.46000001            2000
        52          53           1  0.46000001            2000
        53          54           1  0.46000001            2000
        54          55           1  0.46000001            2000
        55          56           1  0.46000001            2000
        56          57           1  0.46000001            2000
        57          58           1  0.46000001            2000
        58          59           1  0.46000001            2000
        59          60           1  0.46000001            2000
        60          61           1  0.46000001            2000
        61          62           1  0.46000001            2000
        62          63           1  0.46000001            2000
        63          64           1  0.46000001            2000
        64          65           1  0.46000001            2000
        65          66           1  0.46000001            2000
        66          67           1  0.46000001            2000
        67          68           1  0.46000001            2000
        68          69           1  0.46000001            2000
        69          70           1  0.46000001            2000
        70          71           1  0.46000001            2000
        71          72           1  0.46000001            2000
        72          73           1  0.46000001            2000
        73          74           1  0.46000001            2000
        74          75           1  0.46000001            2000
        75          76           1  0.46000001            2000
        76          77           1  0.46000001            2000
        77          78           1  0.46000001            2000
        78          79           1  0.46000001            2000
        79          80           1  0.46000001            2000

[angles]
; i j k       funct   angle   force.c.
         1           2           3           2   180.00000       82.000000
         2           3           4           2   180.00000       82.000000
         3           4           5           2   180.00000       82.000000
         4           5           6           2   180.00000       82.000000
         5           6           7           2   180.00000       82.000000
         6           7           8           2   180.00000       82.000000
         7           8           9           2   180.00000       82.000000
         8           9          10           2   180.00000       82.000000
         9          10          11           2   180.00000       82.000000
        10          11          12           2   180.00000       82.000000
        11          12          13           2   180.00000       82.000000
        12          13          14           2   180.00000       82.000000
        13          14          15           2   180.00000       82.000000
        14          15          16           2   180.00000       82.000000
        15          16          17           2   180.00000       82.000000
        16          17          18           2   180.00000       82.000000
        17          18          19           2   180.00000       82.000000
        18          19          20           2   180.00000       82.000000
        19          20          21           2   180.00000       82.000000
        20          21          22           2   180.00000       82.000000
        21          22          23           2   180.00000       82.000000
        22          23          24           2   180.00000       82.000000
        23          24          25           2   180.00000       82.000000
        24          25          26           2   180.00000       82.000000
        25          26          27           2   180.00000       82.000000
        26          27          28           2   180.00000       82.000000
        27          28          29           2   180.00000       82.000000
        28          29          30           2   180.00000       82.000000
```

```
        29          30          31           2   180.00000       82.000000
        30          31          32           2   180.00000       82.000000
        31          32          33           2   180.00000       82.000000
        32          33          34           2   180.00000       82.000000
        33          34          35           2   180.00000       82.000000
        34          35          36           2   180.00000       82.000000
        35          36          37           2   180.00000       82.000000
        36          37          38           2   180.00000       82.000000
        37          38          39           2   180.00000       82.000000
        38          39          40           2   180.00000       82.000000
        39          40          41           2   180.00000       82.000000
        40          41          42           2   180.00000       82.000000
        41          42          43           2   180.00000       82.000000
        42          43          44           2   180.00000       82.000000
        43          44          45           2   180.00000       82.000000
        44          45          46           2   180.00000       82.000000
        45          46          47           2   180.00000       82.000000
        46          47          48           2   180.00000       82.000000
        47          48          49           2   180.00000       82.000000
        48          49          50           2   180.00000       82.000000
        49          50          51           2   180.00000       82.000000
        50          51          52           2   180.00000       82.000000
        51          52          53           2   180.00000       82.000000
        52          53          54           2   180.00000       82.000000
        53          54          55           2   180.00000       82.000000
        54          55          56           2   180.00000       82.000000
        55          56          57           2   180.00000       82.000000
        56          57          58           2   180.00000       82.000000
        57          58          59           2   180.00000       82.000000
        58          59          60           2   180.00000       82.000000
        59          60          61           2   180.00000       82.000000
        60          61          62           2   180.00000       82.000000
        61          62          63           2   180.00000       82.000000
        62          63          64           2   180.00000       82.000000
        63          64          65           2   180.00000       82.000000
        64          65          66           2   180.00000       82.000000
        65          66          67           2   180.00000       82.000000
        66          67          68           2   180.00000       82.000000
        67          68          69           2   180.00000       82.000000
        68          69          70           2   180.00000       82.000000
        69          70          71           2   180.00000       82.000000
        70          71          72           2   180.00000       82.000000
        71          72          73           2   180.00000       82.000000
        72          73          74           2   180.00000       82.000000
        73          74          75           2   180.00000       82.000000
        74          75          76           2   180.00000       82.000000
        75          76          77           2   180.00000       82.000000
        76          77          78           2   180.00000       82.000000
        77          78          79           2   180.00000       82.000000
        78          79          80           2   180.00000       82.000000

 [dihedrals]
 ; i j k h     funct   c0 c1 c2 c3 c4 c5
    1    2     3      4     11  -19.665600       0.5092800      1.12000000     -4.40827012E-02 -
2.01571006E-02   0.0000000
    2    3     4      5     11  -19.665600       0.5092800      1.12000000     -4.40827012E-02 -
2.01571006E-02   0.0000000
    3    4     5      6     11  -19.665600       0.5092800      1.12000000     -4.40827012E-02 -
2.01571006E-02   0.0000000
    4    5     6      7     11  -19.665600       0.5092800      1.12000000     -4.40827012E-02 -
2.01571006E-02   0.0000000
    5    6     7      8     11  -19.665600       0.5092800      1.12000000     -4.40827012E-02 -
2.01571006E-02   0.0000000
    6    7     8      9     11  -19.665600       0.5092800      1.12000000     -4.40827012E-02 -
2.01571006E-02   0.0000000
    7    8     9     10     11  -19.665600       0.5092800      1.12000000     -4.40827012E-02 -
2.01571006E-02   0.0000000
    8    9    10     11     11  -19.665600       0.5092800      1.12000000     -4.40827012E-02 -
2.01571006E-02   0.0000000
    9   10    11     12     11  -19.665600       0.5092800      1.12000000     -4.40827012E-02 -
2.01571006E-02   0.0000000
   10   11    12     13     11  -19.665600       0.5092800      1.12000000     -4.40827012E-02 -
2.01571006E-02   0.0000000
```

```
  11   12   13    14     11  -19.665600       0.5092800      1.12000000     -4.40827012E-02 -
2.01571006E-02   0.0000000
  12   13   14    15     11  -19.665600       0.5092800      1.12000000     -4.40827012E-02 -
2.01571006E-02   0.0000000
  13   14   15    16     11  -19.665600       0.5092800      1.12000000     -4.40827012E-02 -
2.01571006E-02   0.0000000
  14   15   16    17     11  -19.665600       0.5092800      1.12000000     -4.40827012E-02 -
2.01571006E-02   0.0000000
  15   16   17    18     11  -19.665600       0.5092800      1.12000000     -4.40827012E-02 -
2.01571006E-02   0.0000000
  16   17   18    19     11  -19.665600       0.5092800      1.12000000     -4.40827012E-02 -
2.01571006E-02   0.0000000
  17   18   19    20     11  -19.665600       0.5092800      1.12000000     -4.40827012E-02 -
2.01571006E-02   0.0000000
  18   19   20    21     11  -19.665600       0.5092800      1.12000000     -4.40827012E-02 -
2.01571006E-02   0.0000000
  19   20   21    22     11  -19.665600       0.5092800      1.12000000     -4.40827012E-02 -
2.01571006E-02   0.0000000
  20   21   22    23     11  -19.665600       0.5092800      1.12000000     -4.40827012E-02 -
2.01571006E-02   0.0000000
  21   22   23    24     11  -19.665600       0.5092800      1.12000000     -4.40827012E-02 -
2.01571006E-02   0.0000000
  22   23   24    25     11  -19.665600       0.5092800      1.12000000     -4.40827012E-02 -
2.01571006E-02   0.0000000
  23   24   25    26     11  -19.665600       0.5092800      1.12000000     -4.40827012E-02 -
2.01571006E-02   0.0000000
  24   25   26    27     11  -19.665600       0.5092800      1.12000000     -4.40827012E-02 -
2.01571006E-02   0.0000000
  25   26   27    28     11  -19.665600       0.5092800      1.12000000     -4.40827012E-02 -
2.01571006E-02   0.0000000
  26   27   28    29     11  -19.665600       0.5092800      1.12000000     -4.40827012E-02 -
2.01571006E-02   0.0000000
  27   28   29    30     11  -19.665600       0.5092800      1.12000000     -4.40827012E-02 -
2.01571006E-02   0.0000000
  28   29   30    31     11  -19.665600       0.5092800      1.12000000     -4.40827012E-02 -
2.01571006E-02   0.0000000
  29   30   31    32     11  -19.665600       0.5092800      1.12000000     -4.40827012E-02 -
2.01571006E-02   0.0000000
  30   31   32    33     11  -19.665600       0.5092800      1.12000000     -4.40827012E-02 -
2.01571006E-02   0.0000000
  31   32   33    34     11  -19.665600       0.5092800      1.12000000     -4.40827012E-02 -
2.01571006E-02   0.0000000
  32   33   34    35     11  -19.665600       0.5092800      1.12000000     -4.40827012E-02 -
2.01571006E-02   0.0000000
  33   34   35    36     11  -19.665600       0.5092800      1.12000000     -4.40827012E-02 -
2.01571006E-02   0.0000000
  34   35   36    37     11  -19.665600       0.5092800      1.12000000     -4.40827012E-02 -
2.01571006E-02   0.0000000
  35   36   37    38     11  -19.665600       0.5092800      1.12000000     -4.40827012E-02 -
2.01571006E-02   0.0000000
  36   37   38    39     11  -19.665600       0.5092800      1.12000000     -4.40827012E-02 -
2.01571006E-02   0.0000000
  37   38   39    40     11  -19.665600       0.5092800      1.12000000     -4.40827012E-02 -
2.01571006E-02   0.0000000
  38   39   40    41     11  -19.665600       0.5092800      1.12000000     -4.40827012E-02 -
2.01571006E-02   0.0000000
  39   40   41    42     11  -19.665600       0.5092800      1.12000000     -4.40827012E-02 -
2.01571006E-02   0.0000000
  40   41   42    43     11  -19.665600       0.5092800      1.12000000     -4.40827012E-02 -
2.01571006E-02   0.0000000
  41   42   43    44     11  -19.665600       0.5092800      1.12000000     -4.40827012E-02 -
2.01571006E-02   0.0000000
  42   43   44    45     11  -19.665600       0.5092800      1.12000000     -4.40827012E-02 -
2.01571006E-02   0.0000000
  43   44   45    46     11  -19.665600       0.5092800      1.12000000     -4.40827012E-02 -
2.01571006E-02   0.0000000
  44   45   46    47     11  -19.665600       0.5092800      1.12000000     -4.40827012E-02 -
2.01571006E-02   0.0000000
  45   46   47    48     11  -19.665600       0.5092800      1.12000000     -4.40827012E-02 -
2.01571006E-02   0.0000000
  46   47   48    49     11  -19.665600       0.5092800      1.12000000     -4.40827012E-02 -
2.01571006E-02   0.0000000
```

47 48 49 50 11 -19.665600 0.5092800 1.12000000 -4.40827012E-02 -
2.01571006E-02 0.0000000
48 49 50 51 11 -19.665600 0.5092800 1.12000000 -4.40827012E-02 -
2.01571006E-02 0.0000000
49 50 51 52 11 -19.665600 0.5092800 1.12000000 -4.40827012E-02 -
2.01571006E-02 0.0000000
50 51 52 53 11 -19.665600 0.5092800 1.12000000 -4.40827012E-02 -
2.01571006E-02 0.0000000
51 52 53 54 11 -19.665600 0.5092800 1.12000000 -4.40827012E-02 -
2.01571006E-02 0.0000000
52 53 54 55 11 -19.665600 0.5092800 1.12000000 -4.40827012E-02 -
2.01571006E-02 0.0000000
53 54 55 56 11 -19.665600 0.5092800 1.12000000 -4.40827012E-02 -
2.01571006E-02 0.0000000
54 55 56 57 11 -19.665600 0.5092800 1.12000000 -4.40827012E-02 -
2.01571006E-02 0.0000000
55 56 57 58 11 -19.665600 0.5092800 1.12000000 -4.40827012E-02 -
2.01571006E-02 0.0000000
56 57 58 59 11 -19.665600 0.5092800 1.12000000 -4.40827012E-02 -
2.01571006E-02 0.0000000
57 58 59 60 11 -19.665600 0.5092800 1.12000000 -4.40827012E-02 -
2.01571006E-02 0.0000000
58 59 60 61 11 -19.665600 0.5092800 1.12000000 -4.40827012E-02 -
2.01571006E-02 0.0000000
59 60 61 62 11 -19.665600 0.5092800 1.12000000 -4.40827012E-02 -
2.01571006E-02 0.0000000
60 61 62 63 11 -19.665600 0.5092800 1.12000000 -4.40827012E-02 -
2.01571006E-02 0.0000000
61 62 63 64 11 -19.665600 0.5092800 1.12000000 -4.40827012E-02 -
2.01571006E-02 0.0000000
62 63 64 65 11 -19.665600 0.5092800 1.12000000 -4.40827012E-02 -
2.01571006E-02 0.0000000
63 64 65 66 11 -19.665600 0.5092800 1.12000000 -4.40827012E-02 -
2.01571006E-02 0.0000000
64 65 66 67 11 -19.665600 0.5092800 1.12000000 -4.40827012E-02 -
2.01571006E-02 0.0000000
65 66 67 68 11 -19.665600 0.5092800 1.12000000 -4.40827012E-02 -
2.01571006E-02 0.0000000
66 67 68 69 11 -19.665600 0.5092800 1.12000000 -4.40827012E-02 -
2.01571006E-02 0.0000000
67 68 69 70 11 -19.665600 0.5092800 1.12000000 -4.40827012E-02 -
2.01571006E-02 0.0000000
68 69 70 71 11 -19.665600 0.5092800 1.12000000 -4.40827012E-02 -
2.01571006E-02 0.0000000
69 70 71 72 11 -19.665600 0.5092800 1.12000000 -4.40827012E-02 -
2.01571006E-02 0.0000000
70 71 72 73 11 -19.665600 0.5092800 1.12000000 -4.40827012E-02 -
2.01571006E-02 0.0000000
71 72 73 74 11 -19.665600 0.5092800 1.12000000 -4.40827012E-02 -
2.01571006E-02 0.0000000
72 73 74 75 11 -19.665600 0.5092800 1.12000000 -4.40827012E-02 -
2.01571006E-02 0.0000000
73 74 75 76 11 -19.665600 0.5092800 1.12000000 -4.40827012E-02 -
2.01571006E-02 0.0000000
74 75 76 77 11 -19.665600 0.5092800 1.12000000 -4.40827012E-02 -
2.01571006E-02 0.0000000
75 76 77 78 11 -19.665600 0.5092800 1.12000000 -4.40827012E-02 -
2.01571006E-02 0.0000000
76 77 78 79 11 -19.665600 0.5092800 1.12000000 -4.40827012E-02 -
2.01571006E-02 0.0000000
77 78 79 80 11 -19.665600 0.5092800 1.12000000 -4.40827012E-02 -
2.01571006E-02 0.0000000
